# Unique Reactivity of Triazolyl Diazoacetates under Photochemical Conditions

**DOI:** 10.1021/acsorginorgau.4c00019

**Published:** 2024-06-12

**Authors:** Marzena Wosińska-Hrydczuk, Mohadese Yaghoobi Anzabi, Jakub Przeździecki, Oksana Danylyuk, Wojciech Chaładaj, Dorota Gryko

**Affiliations:** † Institute of Organic Chemistry, Polish Academy of Sciences, Kasprzaka 44/52, 01-224 Warsaw, Poland; ‡ Department of Chemistry, Warsaw University of Technology, Noakowskiego 3, 00-664 Warsaw, Poland; § Institute of Physical Chemistry, Polish Academy of Sciences, Kasprzaka 44/52, 01-224 Warsaw, Poland

**Keywords:** photochemistry, diazo compounds, radicals, carbenes, triazole

## Abstract

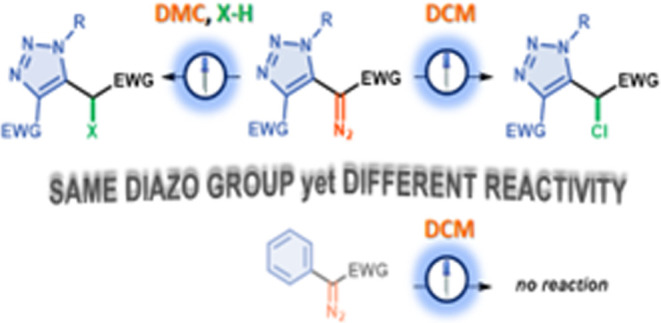

Under light irradiation, aryldiazo acetates can generate either singlet or triplet carbenes depending on the reaction conditions, but heteroaryl diazo compounds have remained underexplored in this context. Herein, we found that triazolyl diazoacetates exhibit higher reactivity than their aryl counterparts. They even react with dichloromethane (DCM), a common, inert solvent, for photoreactions involving diazo reagents, giving halogenated products. Theoretical studies show that all reactions involve carbenes but progress via different pathways depending on the solvent used.

Diazo compounds are neutral divalent species, which are versatile precursors of carbenes, the crucial intermediates in organic chemistry.^1,2^ They are a powerful tool for the formation of various C–C and C–X bonds,^2−4^ with notable applications in the synthesis of pharmaceuticals.^5−7^ Distinguished by their electronic spins, carbenes are classified as singlet or triplet and the substituents on the carbene carbon atom define their spin state. Singlet carbenes typically engage in cheletropic reactions, either as electrophiles or nucleophiles, while triplet carbenes participate in radical addition reactions.^2,8^ Of particular interest, are recent contributions to the library of reactions involving the photochemical generation of these reactive species.^9−13^ Under light irradiation, singlet carbenes are mainly generated via direct photolysis of diazo compounds, while photosensitization gives access to triplet carbenes. Along this line, the reactivity of aryl diazoacetates has been broadly studied, but the influence of heteroaryl moieties in these reactions remains underexplored. In 2019, Gevorgyan et al. reported that under light irradiation, pyridotriazoles undergo a ring chain tautomerization to obtain a diazo tautomer, which after generates diazopyridyl carbenes (Figure 1, Ia).^14^ The resulting pyridyl carbenes exhibit versatile reactivity, participating in arylation, X–H insertion, or cyclopropanation reactions. An analogous tautomerization was proposed for triazolyl diazoacetates by L’abbé and Dehaen in 1987, but under thermal conditions, only one of the diazo moieties forms a carbene, while the other recombines to the triazole (Figure 1, Ib).^15^ This was consistent with previous studies showing that 5-diazomethyl-1,4-diphenyl triazole (**1**) reacts with benzene releasing N_2_ without any stimuli to give cycloheptatriene derivative **2** in 69% yield (Figure 1, IIa).^16^ The introduction of an aryl substituent at the diazo carbon leads to a mixture of triplet-derived products, whereas a stabilizing ester substituent allows the synthesis of product **4** after 4 days (Figure 1, IIb).^15^

**Figure 1:**
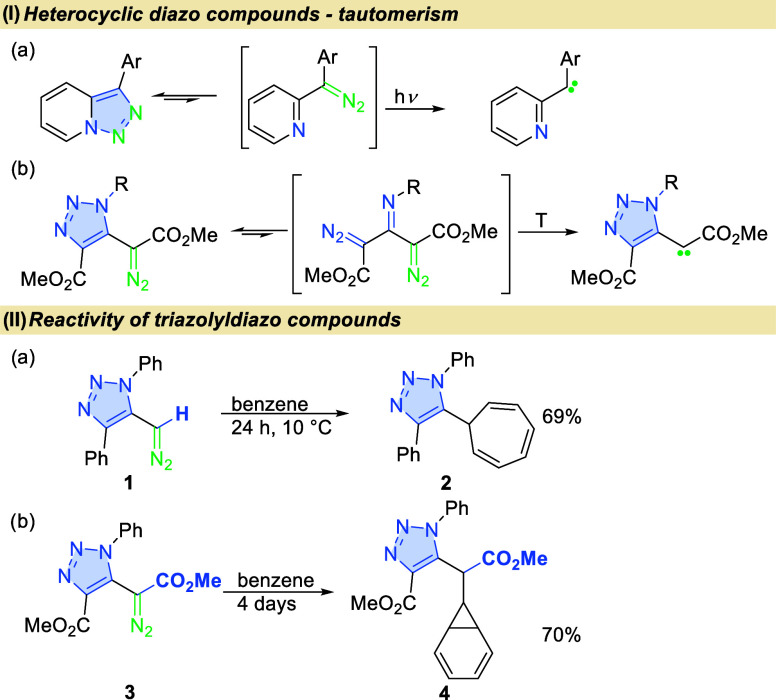
Reactivity of triazolyl diazo compounds.

The Koenigs group found that for diaryl reagents, their singlet/triplet splitting depends on the electronic properties of aryl rings.^17^ Under light irradiation, electron-deficient aryl/aryl diazoalkanes generate singlet carbenes, while for electron-rich aryl/aryl reagents triplet reactivity was observed. Along this line, Tamioka et al. compared the photoreactivity of diphenyl carbenes with aryl(triazolyl) analogues and showed a unique role for the triazolyl moiety that elongates the lifetime of the triplet carbene by 2 orders of magnitude with respect to the phenyl-substituted analogue.^18,19^

Our long-standing interest in the photochemistry of diazo compounds led us to examine the influence of the triazolyl moiety on the photoreactivity of aryldiazo acetates. *We wondered whether under light irradiation these compounds would behave similarly to those of aryl diazoacetates or would induce ring–chain tautomerization with concomitant loss of dinitrogen*.

Previously, triazolyl diazo acetates were synthesized by L’abbé via the condensation of dimethyl acetonedicarboxylate (**5**) with aryl azides yielding a triazole derivative, followed by diazo transfer furnishing the desired compound.^15^ Our revised method, utilizes enamines **6a**–**e**, prepared in one-step from **5**, followed by diazo transfer with 4-acetamidobenzenesulfonyl azide (*p*-ABSA) in the presence of 1,8-diazabicyclo(5.4.0)undec-7-ene (DBU) to obtain diazo derivatives **7a**–**d** (for optimization studies, see SI, Scheme 1).^20−23^ Overall, the condensation of dicarboxylate **5** with amines was performed in the presence of 4 Å molecular sieves in toluene; the subsequent diazo transfer requires 1.5 equiv of *p*-ABSA and 1.25 equiv of DBU in dry acetonitrile. X-ray analysis of compound **7b** unambiguously confirmed its structure.^24^ The optimized methodology enabled the synthesis of *N*-substituted triazolyl diazoacetates **7**, including benzyl, cyclopropyl, and *n*-propyl. However, condensation with *t*-BuNH_2_ did not lead to desired product **6e**.

**Scheme 1:**
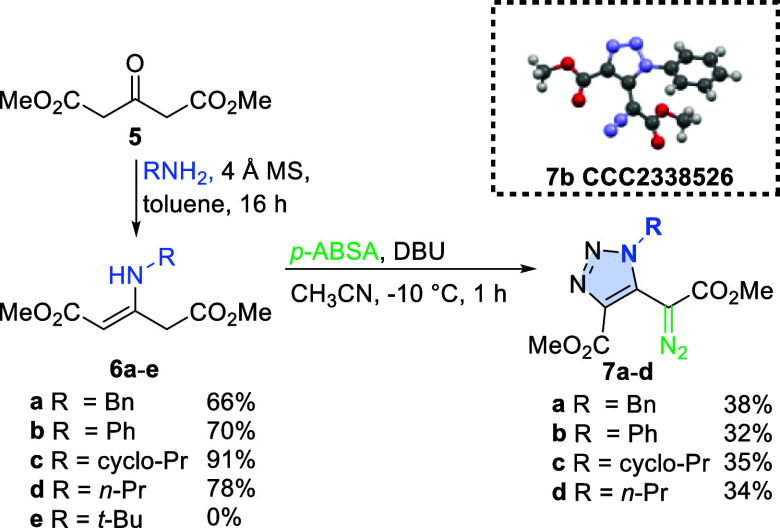
Synthesis of Triazolyl Diazo Compounds Conditions for diazo transfer: *p*-ABSA (1.5 equiv, 0.15 mmol), DBU (1.25 equiv, 0.125 mmol), **6** (1 equiv, 0.1 mmol) dry acetonitrile (0.25 mL), −10 °C, Ar atmosphere, 1 h. (For details, see SI).

The UV/vis spectra of triazolyl diazoacetates **7** show that the λ_max_ is slightly hypsochromically shifted compared to the model phenyl diazoacetate (**8**, Figure 2I).^9^ However, all synthesized reagents **7** absorb in the visible region and thus can be activated by blue light irradiation. Replacement of the phenyl moiety with the triazolyl one has a strong impact on the S_0_–S_1_ gap. For phenyl diazo acetate (**8**), it is equal to 3.5 eV, while for model triazolyl derivative **7e**, the gap decreases to 3.35 eV (Figure 2, II).

**Figure 2:**
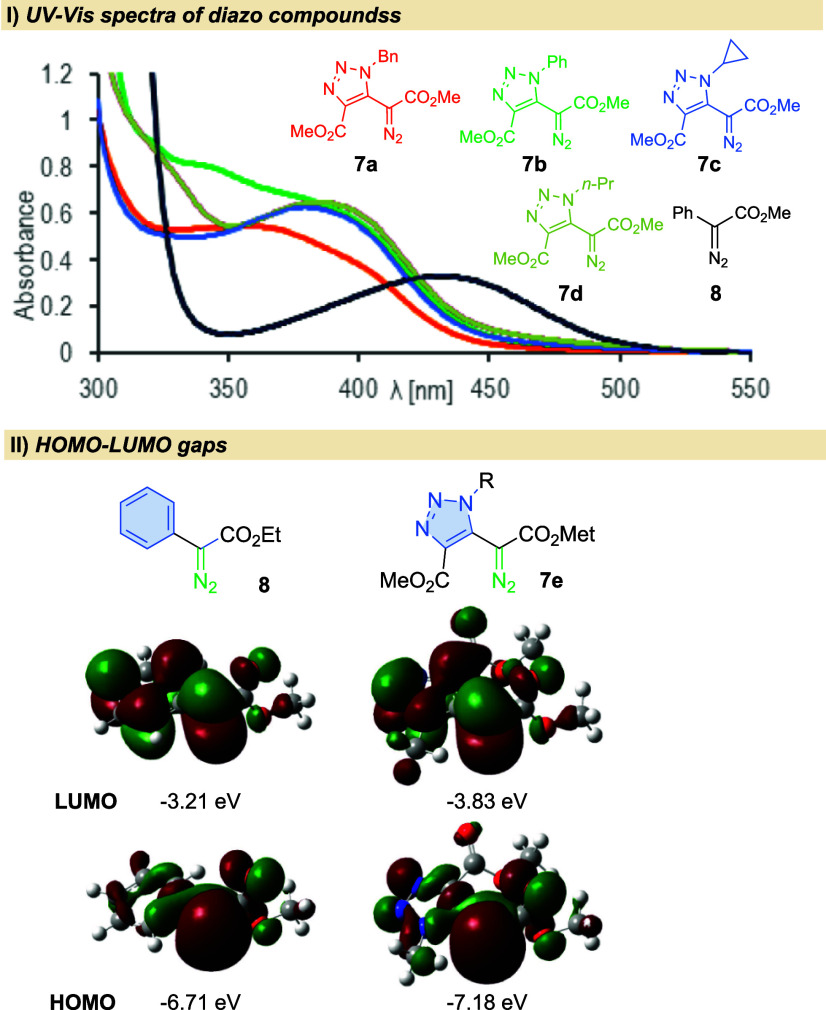
(I) UV–vis spectra of *N*-substituted triazolyl diazoacetates. (II) Highest occupied molecular orbital (HOMO) and lowest occupied molecular orbital (LUMO) energies for phenyl and triazolyl diazoacetates.

## Photochemical Reactivity of Diazo Compounds in a Halogenated Solvent

**Scheme 2:**
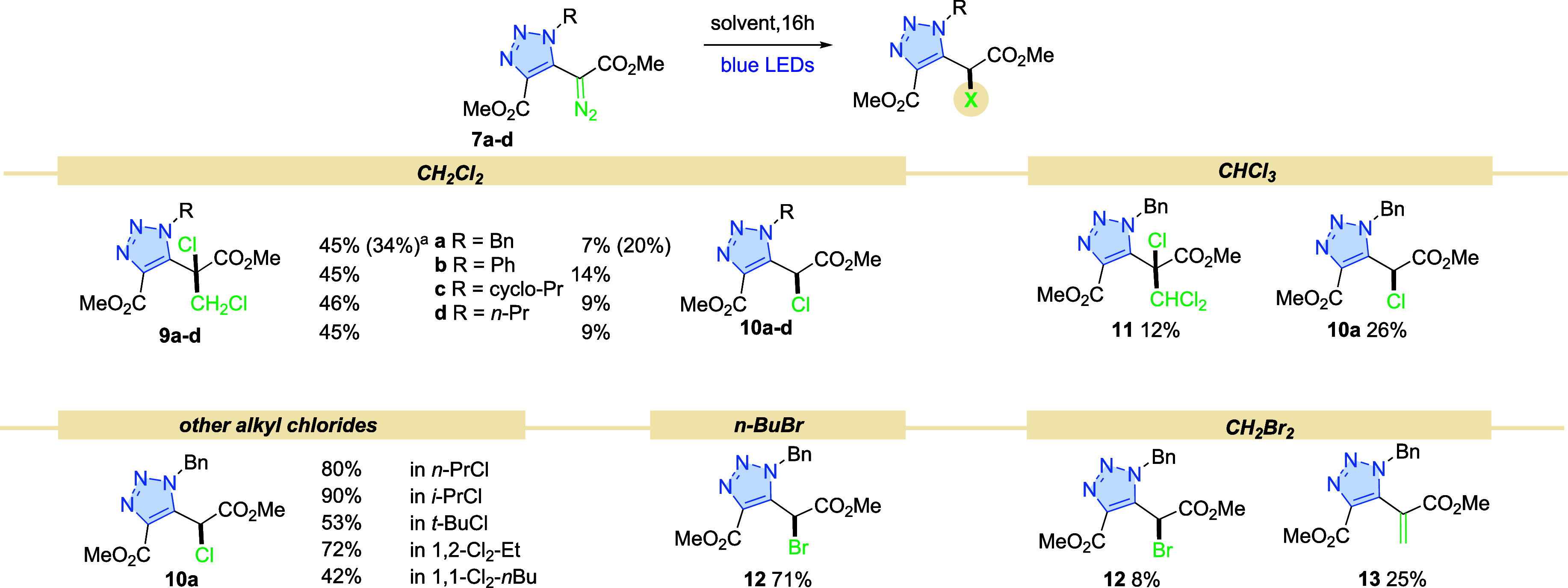
Reaction of Diazo Compounds **7** with Halogenated Solvents^,^ Conditions: diazo compound **7** (0.16 mmol), solvent (4.3 mL, *c* = 0.04 M), blue LEDs (450 nm, 7 W), 16 h, isolated yield (for details, see Supporting Information). 1 mmol scale.

Photochemical transformations of aryl diazo compounds are usually performed in dichloromethane (DCM), as other common solvents are reactive to carbenes.^11^ Intriguingly, in our case, under blue light irradiation, triazolyl diazoacetate **7a** in DCM was completely consumed after 16 h. Two distinct products formed; the primary product exhibited characteristics indicative of the addition of one molecule of DCM to carbene **9a**, while the second compound demonstrated only Cl–H insertion **10a** (Scheme 2). This unusual reactivity is consistent across diazo compounds **7a**–**d**, all predominantly gave product **9** in similar yields.

On the contrary, in the benchmark Rh-catalyzed reaction in DCM, either at room temperature or under reflux, diazo compound **7a** remained almost intact.

Furthermore, other primary, secondary, or tertiary alkyl chlorides, including *n*-propyl-, *i*-propyl-, and *t*-butyl chlorides were evaluated. In these cases, however, chlorinated products **10a** were formed exclusively as confirmed by the ^1^H NMR analysis of the crude reaction mixtures. Similarly, dichloroalkanes, regardless of the substitution pattern, 1,2- or 1,1- provided the corresponding product **10** in 72 and 42%, respectively.

To determine the impact of the halide moiety, *n*-butyl bromide was reacted with diazo compound **7a** under blue light irradiation. The reaction afforded the corresponding brominated compound **12**, with a yield of 71%. In contrast to DCM, dibromomethane predominantly formed olefin **13**. The higher reactivity of bromoalkanes in these transformations is reflected in a complex mixture of products for the reaction with bromoform. Unlike the recent report by Wu et al., which showed that aryl diazoacetates react with bromoform to give brominated derivatives.^25^ Wu’s and our data underline the higher reactivity of triazolyl diazo acetates **7** over the phenyl analogue **8**.

## Photochemical Reactivity of Diazo Compounds **7** in Nonhalogenated Solvents

**Scheme 3:**
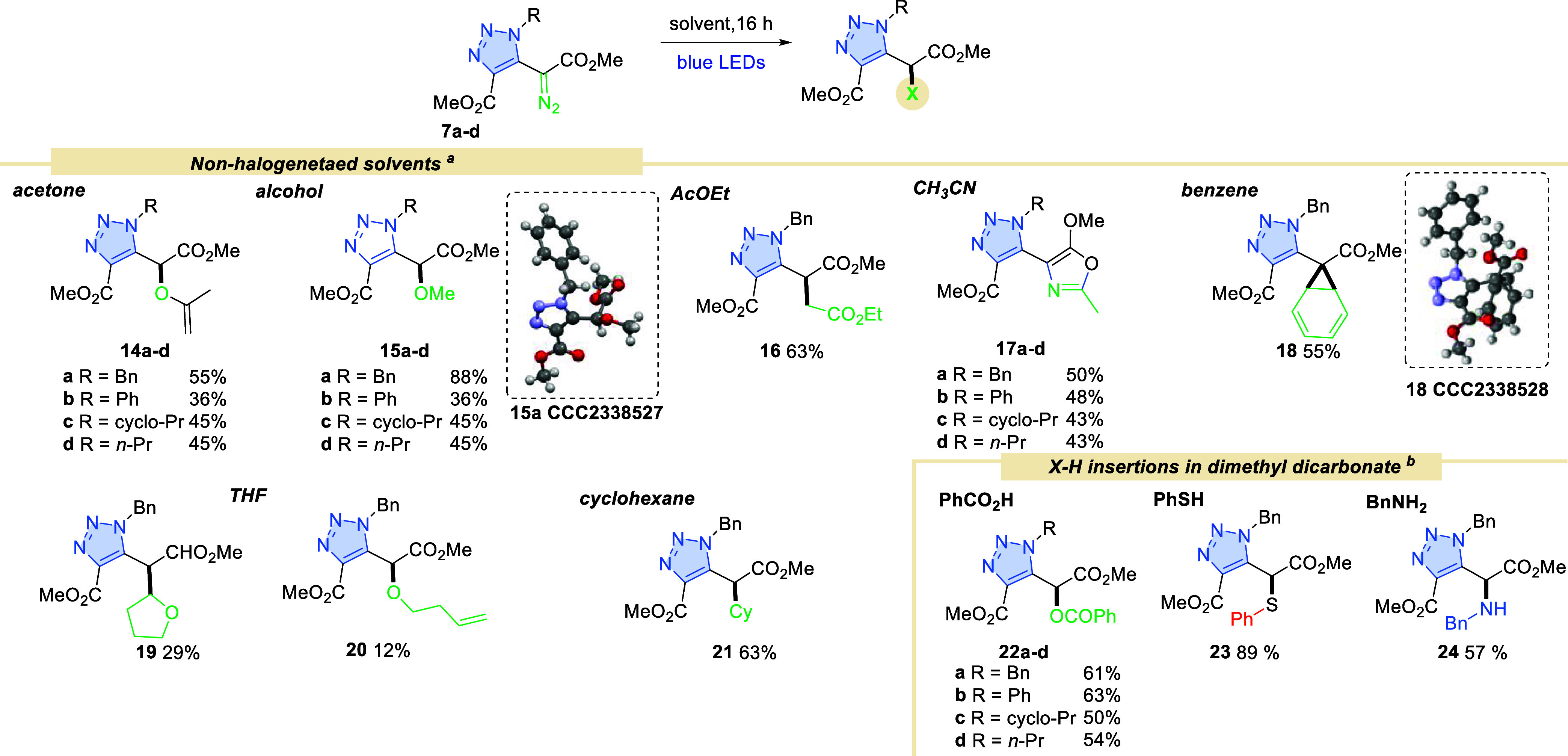
C–H and X–H Insertions of Diazo Compounds with Nonhalogenated Solvents Conditions a: diazo compound **7a**–**d** (0.16 mmol), solvent (4.3 mL, *c* = 0.04 M), blue LEDs (450 nm, 7 W), 16 h, isolated yield; Conditions b: diazo compound (0.1 mmol), X–H reagent (10 equiv, 1 mmol) 2.0 mL of dimethyl carbonate (DMC) (*c* = 0.05 M), blue LEDs (450 nm, 7 W), 16 h, isolated yield (for details, see Supporting Information).

Furthermore, as expected, other solvents, acetone, acetonitrile, benzene, methanol, ethyl acetate, cyclohexane, and tetrahydrofuran reacted with diazo compounds **7** under blue light irradiation similarly to phenyl diazoacetate **8** (Scheme 3). The reaction of compound **7a** with acetone led to enol adduct **14a** in 55% yield. A similar reaction was reported by Alt and Maas in 1994, in the presence of a rhodium catalyst^26^ and by Crocker et al. under photochemical conditions.^27^

In acetonitrile, oxazole **17** formed upon photolysis of the diazo reagent. This reactivity for aryl diazoacetates under photochemical conditions (34 W, 440 nm) has just been recently described by Maiti and co-workers.^28^ It was noted that the photochemical energy of 5 W was not sufficient to drive this reaction, in contrast to triazolyl diazoacetate **7**, when under 7 W LEDs irradiation, it furnishes the desired product. This result further corroborates the greater influence of the triazolyl moiety on the reactivity of the diazo group. This reaction is believed to involve the generation of singlet carbenes, which is trapped by acetonitrile in a [3 + 2] cycloaddition.

Previously, Buu et al. reported the formation of oxazole also from ethyl diazoacetate.^29^ Furthermore, the reaction with benzene afforded cycloaddition product **18** in a moderate yield, under thermal conditions the reaction requires 4 days to complete.^15^ In the cases of cyclohexane, ethyl acetate, and methanol, C–H and O–H insertion occurred, respectively, while the reaction with THF leads to a mixture of two products **19** and **20**, as previously reported by Jurberg and Davies for phenyl diazoacetate **8**.^9^

Following an exhaustive examination of conventional organic solvents, dimethyl carbonate (DMC) emerged as a promising medium for certain photochemical insertions.^30^ DMC is known as a green solvent and is a good substitute for many halogenated solvent.^31,32^ When substrate **7a** was irradiated in DMC under blue LEDs, product **15a**, the same as in MeOH, formed in 7% yield (Scheme 3). In the presence of other reagents, however, OH, SH, and NH insertions occurred, furnishing products **22**, **23**, and **24** respectively.

## Mechanistic Considerations

**Scheme 4:**
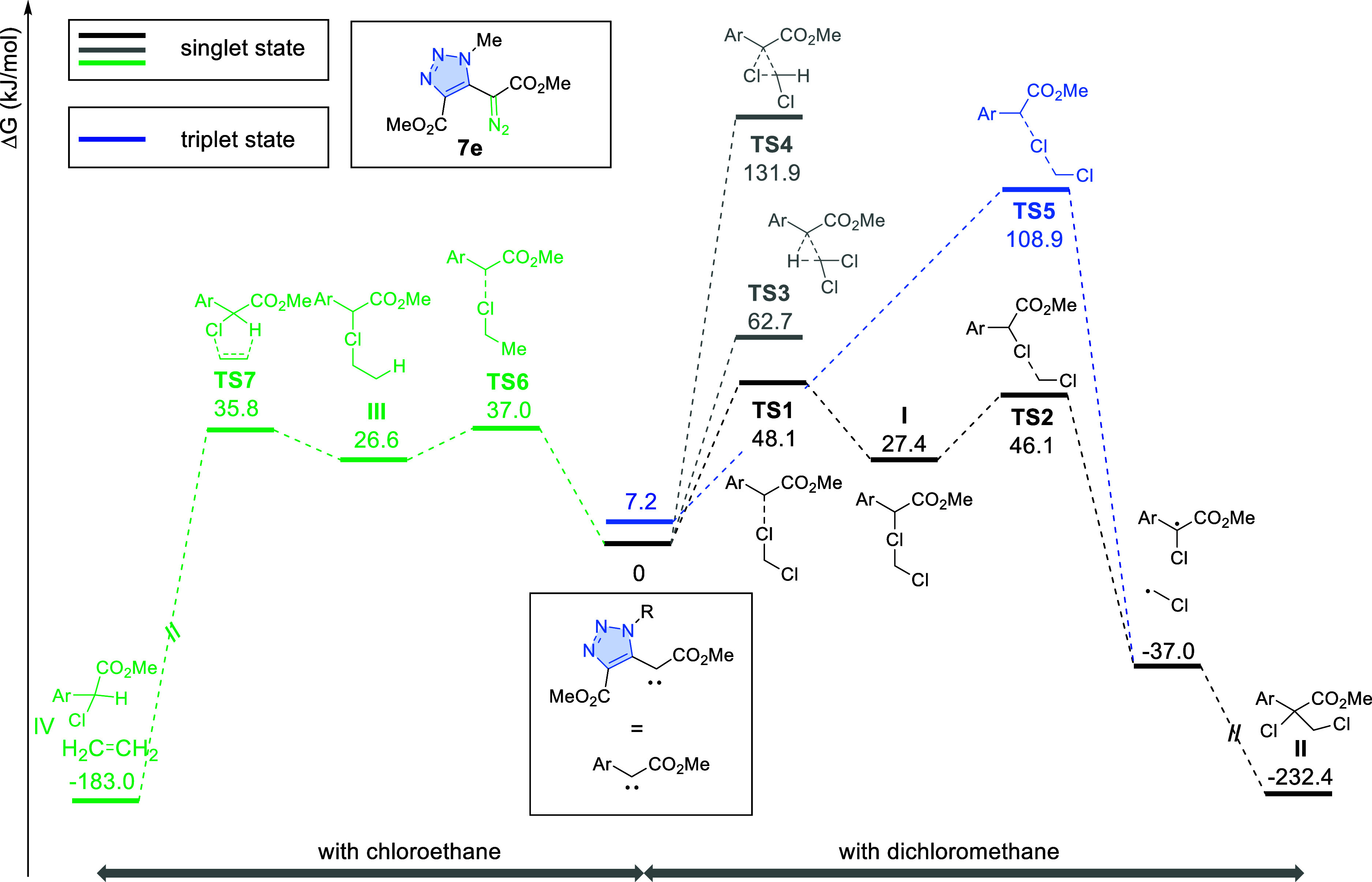
Reaction Profile Calculated at M06/6-311++G(d,p)/SMD(DCM)//B3LYP-D3/6-31G(d)

To gain a better understanding of the underlying reaction mechanism involving triazolyl diazoacetates **7**, the reaction of a model diazo compound **7e** (R = Me) with DCM and chloroethane was studied computationally. All the calculations were performed with Gaussian 16 package^33^ at M06/6-311++G(d,p)/SMD(DCM)//B3LYP-D3/6-31G(d) level of theory. The carbene, derived from diazo reagent **7e**, has a singlet ground state with a singlet–triplet gap of 7.2 kJ/mol. It can easily react with DCM (**TS1**, Δ*G*^‡^ = 48.1 kJ/mol) giving rise to chloronium ylide **I**, which is suspectable to facile homolytic cleavage of the C–Cl bond (**TS2**, Δ*G*^‡^ = 18.7 kJ/mol, Scheme 4). The resulting pair of radicals can recombine to form adduct **II**. It could be argued that a similar pair of radicals can be accessed via halogen atom transfer to a triplet carbene via **TS5**; however, this path is associated with a barrier of 102.7 kJ/mol. Furthermore, direct insertion of a singlet carbene into C–Cl or C–H bonds through **TS3** and **TS4** is also less preferred (Δ*G*^‡^ = 131.9 and 32.7 kJ/mol, respectively).

**Figure 3:**
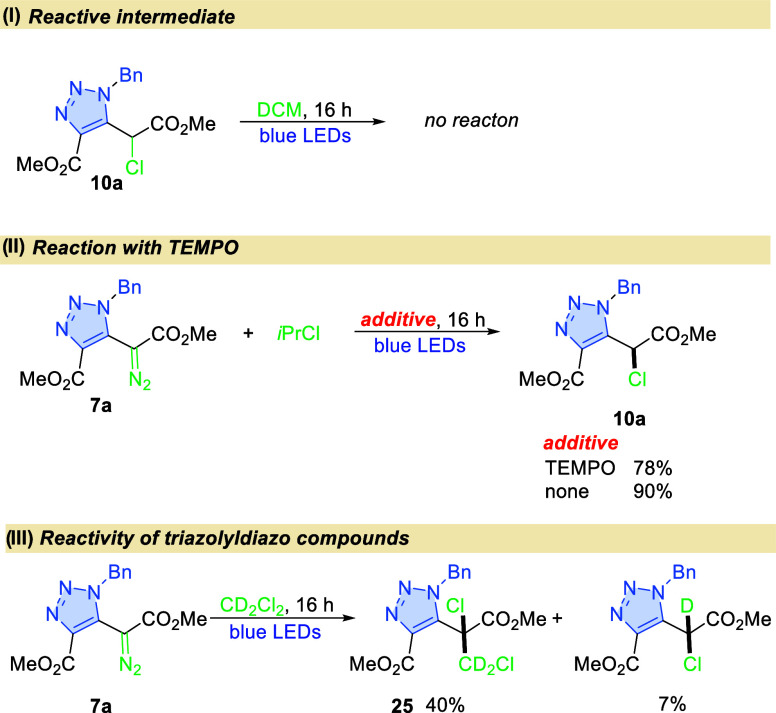
Mechanistic experiments^a^.

Experimentally, we have proved that chloro-derivative **10a** is not an intermediate product as it does not transform into final compound **9a** (Figure 3I). The experiment with 2,2,6,6-tetramethyl-1-piperidinyloxy (TEMPO) addition corroborates the radical character of this transformation (the reaction is halted completely). For 2-chloropropane, the addition of TEMPO does not alter the reaction course—product **10a** formed in a slightly diminished yield (Figure 3II). Therefore, a different mechanism was considered. Longer chloroalkanes, which contain a hydrogen atom at the β-position, on the other hand undergo a facile transfer of HCl to the carbene center. Thus, the formation of halonium ylide **III** from chloroethane (**TS6**, Δ*G*^‡^ = 37.0 kJ/mol) is followed by an intramolecular hydrogen-shift (**TS7**, Δ*G*^‡^ = 9.2 kJ/mol). A similar process was postulated for the light-induced bromination of α-diazo compounds with bromoform in THF.^23^ In our case, however, the reaction with bromoform leads to a complex mixture of products.

In conclusion, the difference in the HOMO–LUMO gap for phenyl and triazolyl diazoacetates is reflected in their reactivity under light irradiation. While photochemical reactions for phenyl diazoacetates can be performed in DCM, triazolyl diazoacetates react with the solvent and become chlorinated. All alkyl halides studied behave in the same way. With other commonly used solvents, C–H or X–H insertions occur. The solvent that allows reactions with other reagents is dimethyl decarbonate.

## Experimental Section

### General Procedures

Photochemical reactions were conducted in 10 mL glass vials equipped with an aluminum cap and sealed with a rubber septum. Reactions were monitored by thin layer chromatography (TLC), using 0.20 mm Merck silica plates (60F-254), and visualized using UV-light, cerium molybdate with heat applied as a developing agent. Colum chromatography was performed on Merck silica gel 60 (230–400 mesh). All reported yields, when determined by ^1^H NMR analysis, were normalized using bromoform as an internal standard. Isolated yields refer to homogeneous materials spectroscopically (^1^H NMR).

NMR spectra were recorded at ambient temperature (unless otherwise stated) on Bruker 400 MHz or Varian 500, 600 MHz. Chemical shifts are reported in ppm relative to the tetramethyl silane signal or a residual undeuterated solvent peak (TMS 0 ppm for ^1^H and ^13^C, CHCl_3_–7.26 ppm for ^1^H, and 77.16 ppm for ^13^C). Multiplicities are given as singlet (s), doublet (d), triplet (t), quartet (q), multiplet (m) broad singlet (br), triplet of quartets (tq), triplet of doublet (td), triplet of triplets (tt), and doublet of doublets of doublets (ddd). LR and HRMS. Low-resolution mass spectra (LRMS) were recorded on an Applied Biosystems API 365 mass spectrometer using the electrospray ionization (ESI) technique. High-resolution mass spectra (HRMS) were recorded on the Waters SYNAPT G2-S HDMS instrument using electrospray ionization (ESI) or atmospheric pressure chemical ionization (APCI) with the time-of-flight detector (TOF). Melting points were recorded on a Marienfeld MPM-H2 melting point apparatus and are uncorrected. UV–vis absorption spectra were recorded on 60 UV–vis Agilent Spectrophotometer.

### General Procedure for the Synthesis of Diazo Compounds

To a solution of *p*-ABSA (1.5 equiv, 5.7 mmol, 1.37 g) in dry MeCN (5.0 mL, *c* = 1.1 M) at −10 °C, DBU (1.25 equiv, 4.75 mmol, 0.71 mL) and a solution of enamine **6a**–**d** (1.0 equiv, 3.8 mmol) in dry MeCN (5.0 mL, *c* = 0.76 M) were added. The reaction mixture was stirred at −10 °C for 1 h, then MeCN was evaporated *in vacuo*. To the reaction mixture, saturated NaHCO_3_ and DCM were added, layers were separated, and the aqueous layer was washed with DCM. The combined organic layers were dried over sodium sulfate, filtrated, concentrated *in vacuo*, and purified by flash column chromatography using hexanes/AcOEt to afford the final diazo product.

### General Procedure for Photochemical Reactions in DCM

A glass vial equipped with a stirring bar and sealed with an aluminum cap with a rubber septum was charged with a diazo compound **7a**–**d** (0.16 mmol) in DCM (4.3 mL, *c* = 0.04 M). The reaction mixture was placed in a photoreactor and irradiated with blue LED (450 nm, 7 W) for 16 h. After that time, the crude reaction mixture was concentrated *in vacuo* and purified by flash column chromatography using hexanes/AcOEt to afford the corresponding product.

### General Procedure for Photochemical Reactions in Various Solvents

A glass vial equipped with a stirring bar and sealed with an aluminum cap with a rubber septum was charged with a diazo compound **7a**–**d** (0.16 mmol) in a solvent (4.3 mL, *c* = 0.04 M). The reaction mixture was placed in a photoreactor and irradiated with blue LED (450 nm, 7 W) for 16 h. After that time, the crude reaction mixture was concentrated *in vacuo* and purified by flash column chromatography using hexanes/AcOEt to afford the corresponding product.

## Data Availability

The data underlying this study are available in the published article and its Supporting Information.

## Supporting Information Available

Full description of optimization and mechanistic studies, general procedures, compound characterization (NMR, HRMS), NMR spectra (PDF)
