# Supplementary material for: Unique Reactivity of Triazolyl Diazoacetates under Photochemical Conditions
Source: ACS Org Inorg Au. 2024 Jun 12;4(4):418–23. doi: 10.1021/acsorginorgau.4c00019 (PMC11311451; doi:10.1021/acsorginorgau.4c00019)
Supplement: Supplementary file 1 — gg4c00019_si_001.pdf [file gg4c00019_si_001.pdf]

Supporting Information

Unique Reactivity of Triazolyl Diazoacetates under Photochemical Conditions

Marzena Wosińska-Hrydczuk,‡[a] Mohadese Yaghoobi Anzabi,‡[a] Jakub Przeździecki,[a,b] Oksana Danylyuk,[c] Wojciech Chaładaj,[a]* Dorota Gryko[a]*

[a]Institute of Organic Chemistry, Polish Academy of Sciences, Kasprzaka 44/52, 01-224 Warsaw, Poland

[b]Department of Chemistry, Warsaw University of Technology, Noakowskiego 3, 00-664 Warsaw, Poland

[c]Institute of Physical Chemistry, Polish Academy of Sciences, Kasprzaka 44/52, 01-224 Warsaw, Poland

email: dorota.gryko@icho.edu.pl

TABLE OF CONTENT

1. General Information **6**
2. Photoreactor setups **7**
3. Synthesis of enamine derivatives **9**
3.1 General procedure for the synthesis of enamine derivatives 9
3.2 Characterization of enamines 9
4. Synthesis of diazo compounds **12**
4.1 Diazo transfer reactions – optimization studies 12
4.1.1 Model reaction 12
4.1.2 Control experiments 12
4.1.3 Screening of solvents 12
4.1.4 Screening of amines 13
4.1.5 The influence of the amount of an amine 13
4.1.6 Selection of diazo transfer reagents 13
4.1.7 Influence of the amount of a p-ABSA 13
4.1.8 Optimization of the reaction temperature 14
4.1.9 Optimization of the concentration of diazo reagent 14
4.1.10 Screening of other different reaction conditions 14
4.2 General procedure for the synthesis of diazo compounds 15
4.3 Characterization of diazo compounds 15
5. Photochemical reactions **17**
5.1 Optimization studies for the model reaction of diazo reagent **7a** in DCM 17
5.1.1 Background experiments 17
5.1.2 Optimization of the reaction time 17
5.1.3 The influence of light on the model photoreaction 17
5.2 Reactions of diazo compounds **7a** with different alkyl chloride solvents 18
5.3 Reaction of diazo compound **7a** with DCM on a 1 mmol scale 18
5.3 General procedure for photochemical reactions in various solvents 19
5.2.1 Characterization of products generated in halogenated solvents 20
methyl 1-benzyl-5-(3-methoxy-3-oxoprop-1-en-2-yl)-1H-1,2,3-triazole-4-carboxylate (**13**) 25
5.2.2 Characterization of products generated in non-halogenated solvents 25
5.3 Insertion reactions 35
5.3.1 General procedure for photochemical – NH, OH or SH insertions 35

5.3.2 Characterization of insertion products 36
6. Thermal reactions 41
6.1 General procedure for thermal reactions involving diazo compounds 41
7. Mechanistic considerations 42
7.1 Experiments with the TEMPO radical trap 42
7.2 Experiments in CD_2Cl_2 43
7.2.1 Characterization of the deuterated products 43
8. Crystallographic Data 44
9. Computational studies 47
General 47
Optimized geometries, energies and corrections to thermodynamic functions. 47
10. NMR and IR spectra 63
dimethyl 3-(benzylamino)pent-2-enedioate (**6a**) 63
dimethyl 3-(phenylamino)pent-2-enedioate (**6b**) 64
dimethyl 3-(propylamino)pent-2-enedioate (**6c**) 65
dimethyl 3-(cyclopropylamino)pent-2-enedioate (**6d**) 66
methyl 1-benzyl-5-(1-diazo-2-methoxy-2-oxoethyl)-1H-1,2,3-triazole-4-carboxylate (**7a**) 67
methyl 5-(1-diazo-2-methoxy-2-oxoethyl)-1-phenyl-1H-1,2,3-triazole-4-carboxylate (**7b**) 69
methyl 5-(1-diazo-2-methoxy-2-oxoethyl)-1-propyl-1H-1,2,3-triazole-4-carboxylate (**7d**) 73
methyl 1-benzyl-5-(2,3-dichloro-1-methoxy-1-oxopropan-2-yl)-1H-1,2,3-triazole-4-carboxylate (**9a**) 75
methyl 1-benzyl-5-(1-chloro-2-methoxy-2-oxoethyl)-1H-1,2,3-triazole-4-carboxylate (**10a**) 76
methyl 5-(2,3-dichloro-1-methoxy-1-oxopropan-2-yl)-1-phenyl-1H-1,2,3-triazole-4-carboxylate (**9b**) 77
methyl 5-(1-chloro-2-methoxy-2-oxoethyl)-1-phenyl-1H-1,2,3-triazole-4-carboxylate (**10b**) 78
methyl 1-cyclopropyl-5-(2,3-dichloro-1-methoxy-1-oxopropan-2-yl)-1H-1,2,3-triazole-4-carboxylate (**9c**) 79
methyl 5-(1-chloro-2-methoxy-2-oxoethyl)-1-cyclopropyl-1H-1,2,3-triazole-4-carboxylate (**10c**) 80
methyl 5-(2,3-dichloro-1-methoxy-1-oxopropan-2-yl)-1-propyl-1H-1,2,3-triazole-4-carboxylate (**9d**) 81

methyl 5-(1-chloro-2-methoxy-2-oxoethyl)-1-propyl-1H-1,2,3-triazole-4-carboxylate (**10d**)........ 82
methyl 1-benzyl-5-(1,1,2-trichloro-3-methoxy-3-oxopropan-2-yl)-1H-1,2,3-triazole-4-carboxylate (**11**) 83
methyl 1-benzyl-5-(1-bromo-2-methoxy-2-oxoethyl)-1H-1,2,3-triazole-4-carboxylate (**12**)........ 84
methyl 1-benzyl-5-(2-methoxy-2-oxo-1-(prop-1-en-2-yloxy)ethyl)-1H-1,2,3-triazole-4-carboxylate (**14a**) 86
methyl 5-(2-methoxy-2-oxo-1-(prop-1-en-2-yloxy)ethyl)-1-phenyl-1H-1,2,3-triazole-4-carboxylate (**14b**) 87
methyl 1-cyclopropyl-5-(2-methoxy-2-oxo-1-(prop-1-en-2-yloxy)ethyl)-1H-1,2,3-triazole-4-carboxylate (**14c**)........ 88
methyl 5-(2-methoxy-2-oxo-1-(prop-1-en-2-yloxy)ethyl)-1-propyl-1H-1,2,3-triazole-4-carboxylate (**14d**) 89
methyl 1-benzyl-5-(1,2-dimethoxy-2-oxoethyl)-1H-1,2,3-triazole-4-carboxylate (**15a**) . 90
methyl 5-(1,2-dimethoxy-2-oxoethyl)-1-phenyl-1H-1,2,3-triazole-4-carboxylate (**15b**). 91
methyl 1-cyclopropyl-5-(1,2-dimethoxy-2-oxoethyl)-1H-1,2,3-triazole-4-carboxylate (**15c**) 92
methyl 5-(1,2-dimethoxy-2-oxoethyl)-1-propyl-1H-1,2,3-triazole-4-carboxylate (**15d**) . 93
4-ethyl 1-methyl 2-(1-benzyl-4-(methoxycarbonyl)-1H-1,2,3-triazol-5-yl)succinate (**16**) 94
methyl 1-benzyl-5-(5-methoxy-2-methyloxazol-4-yl)-1H-1,2,3-triazole-4-carboxylate (**17a**)........ 95
methyl 5-(5-methoxy-2-methyloxazol-4-yl)-1-phenyl-1H-1,2,3-triazole-4-carboxylate (**17b**)........ 96
methyl 1-cyclopropyl-5-(5-methoxy-2-methyloxazol-4-yl)-1H-1,2,3-triazole-4-carboxylate (**17c**)........ 97
methyl 5-(5-methoxy-2-methyloxazol-4-yl)-1-propyl-1H-1,2,3-triazole-4-carboxylate (**17d**)........ 98
methyl 1-benzyl-5-(7-(methoxycarbonyl)bicyclo[4.1.0]hepta-2,4-dien-7-yl)-1H-1,2,3-triazole-4-carboxylate (**18**)........ 99
methyl 1-benzyl-5-(2-methoxy-2-oxo-1-(tetrahydrofuran-2-yl)ethyl)-1H-1,2,3-triazole-4-carboxylate (**19**) 100
methyl 1-benzyl-5-(1-(but-3-en-1-yloxy)-2-methoxy-2-oxoethyl)-1H-1,2,3-triazole-4-carboxylate (2**0**) 101
methyl 1-benzyl-5-(1-cyclohexyl-2-methoxy-2-oxoethyl)-1H-1,2,3-triazole-4-carboxylate (**21**)........ 102
methyl 5-(1-(benzoyloxy)-2-methoxy-2-oxoethyl)-1-benzyl-1H-1,2,3-triazole-4-carboxylate (**22a**) 103

methyl 5-(1-(benzoyloxy)-2-methoxy-2-oxoethyl)-1-phenyl-1H-1,2,3-triazole-4-carboxylate (**22b**) 104

methyl 5-(1-(benzoyloxy)-2-methoxy-2-oxoethyl)-1-cyclopropyl-1H-1,2,3-triazole-4-carboxylate (**22c**) 105

methyl 5-(1-(benzoyloxy)-2-methoxy-2-oxoethyl)-1-propyl-1H-1,2,3-triazole-4-carboxylate (**22d**) 106

methyl 1-benzyl-5-(2-methoxy-2-oxo-1-(phenylthio)ethyl)-1H-1,2,3-triazole-4-carboxylate (**23**) 107

methyl 1-benzyl-5-(1-(benzylamino)-2-methoxy-2-oxoethyl)-1H-1,2,3-triazole-4-carboxylate (**24**) 108

methyl 1-benzyl-5-((2R,3S)-1-(methoxycarbonyl)-2,3-diphenylcyclopropyl)-1H-1,2,3-triazole-4-carboxylate (**S3**) 109

methyl 1-benzyl-5-(2,3-dichloro-1-methoxy-1-oxopropan-2-yl-3,3-d2)-1H-1,2,3-triazole-4-carboxylate (**25**) 110

10. References 111

1. General Information

Materials

All solvents and commercially available reagents utilized in this study were procured from Sigma-Aldrich, TCI, and Acros Organics, and were of reagent-grade quality. Dry solvents were either obtained directly from the Solvent Purification System (SPS) or sourced from Sigma Aldrich. All deuterated solvents used were purchased from Eurisotop or Deutero.

General Procedures

The photochemical reactions were conducted in a 10 mL glass vials equipped with an aluminum cap and sealed with a rubber septum. Reactions were monitored by thin layer chromatography (TLC), using 0.20 mm Merck silica plates (60F-254), and visualized using UV-light, cerium molybdate with heat applied as a developing agent. Colum chromatography was performed on Merck silica gel 60 (230-400 mesh). All reported yields, when determined by ^{1}H NMR analysis, were normalized using bromoform as an internal standard. Isolated yields refer to spectroscopically (^{1}H NMR) homogeneous materials.

NMR spectra were recorded at ambient temperature (unless otherwise stated) on Bruker 400 MHz or Varian 500, 600 MHz. Chemical shifts are reported in ppm relative to the tetramethyl silane signal or a residual undeuterated solvent peak (TMS 0 ppm for ^{1}H and ^{13}C, $CHCl_3$ – 7.26 ppm for ^{1}H and 77.16 ppm for ^{13}C. Multiplicities are given as: singlet (s), doublet (d), triplet (t), quartet (q), multiplet (m*)* broad singlet (br), triplet of quartets (tq), triplet of doublet (td), triplet of triplets (tt), doublet of doublets of doublets (ddd).

LR and HRMS. Low-resolution mass spectra (LRMS) were recorded on an Applied Biosystems API 365 mass spectrometer using electrospray ionization (ESI) technique. High-resolution mass spectra (HRMS) were recorded on Waters SYNAPT G2-S HDMS instrument using electrospray ionization (ESI) or atmospheric-pressure chemical ionization (APCI) with time-of-flight detector (TOF).

Melting points were recorded on a Marienfeld MPM-H2 melting point apparatus and are uncorrected.

UV-Vis absorption spectra were recorded on 60 UV-Vis Agilent Spectrophotometer.

GC-FID analyses were performed using Shimadzu GCMS-QP2010 SE gas chromatograph with FID detector and Zebron ZB 5MSi column (length: 30.0 m; thickness: 0.25 um, diameter: 0.25mm).

GC method used:
• time: 14.92 min;
• pressure: 90.8 kPa;
• total flow: 5.3 mL/min;
• column flow: 1.11 mL/min;
• linear velocity: 27.5 cm/s;
• purge flow: 2.0 mL/min;
• split ratio: 2.0.

Preparative HPLC separations were performed using Knauer HPLC chromatograph with PDA detector and Preparative column chromatography Knauer EII 100-10 Si column (250 x 20 mm), flow rate: 10 mL/min.

Analytical HPLC analysis were performed using Knauer Azura (ASM 2.1 L, DAD 6.1 L) using C18 reversed-phase silicagel 90 Å (Sigma-Aldrich) with redistilled water and HPLC grade MeCN as eluents.
HPLC measurement conditions:

- column: Kromasil Eternity – 5 - C18, 250 mm × 4.6 mm with a precolumn;
- detection, UV/vis;
- pressure, 10 MPa;
- temperature, 30 °C;
- flow rates: 1 mL/min;
- mobile Phase: 0.03% v formic acid in water/MeCN.

Flash column chromatography was performed on CombiFlash NextGen 300 Flash Chromatography System.

2. Photoreactor setups

1) Photocatalytic reactions were performed using TAK 120 photoreactor equipped with blue LEDs (OSRAM OSLON® SSL 80 GD CS8PM1. 14 LEDs (λ = 455 nm (± 15 nm, 7 W), unless stated otherwise (Figure S1). The reaction vials (10 mL crimp cap vials) were illuminated from the bottom with blue LEDs and cooled from the side using a custom-made fan.

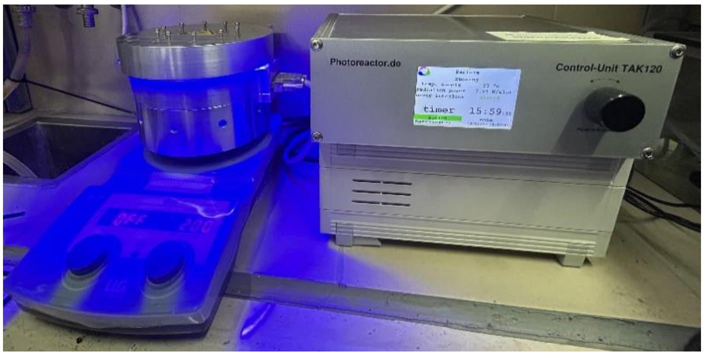

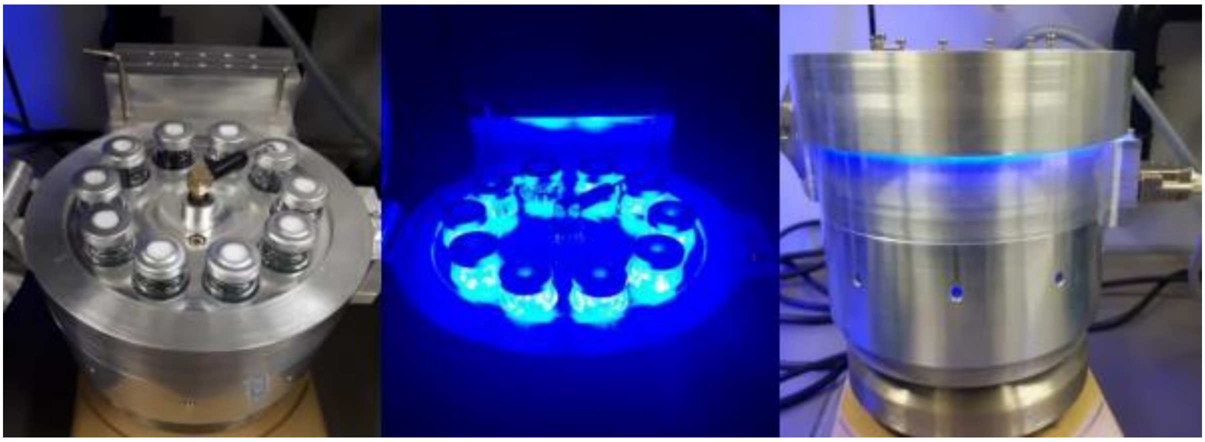

Figure S1. Standard photoreactor setup.

2) Photocatalytic reactions on 1mmol scale between diazo compounds **7a** and DCM was performed using commercially available Kessil lamps. Blue (emission maximum at 440 nm) light was supplied to each reaction vial with the use of two Kessil lamps, each of overall 40 W intensity (when 100% power applied), placed at opposite sites (Figure F2). The ambient temperature of LED block was maintained by cooling with the use of fans (Treaction ~30°C)

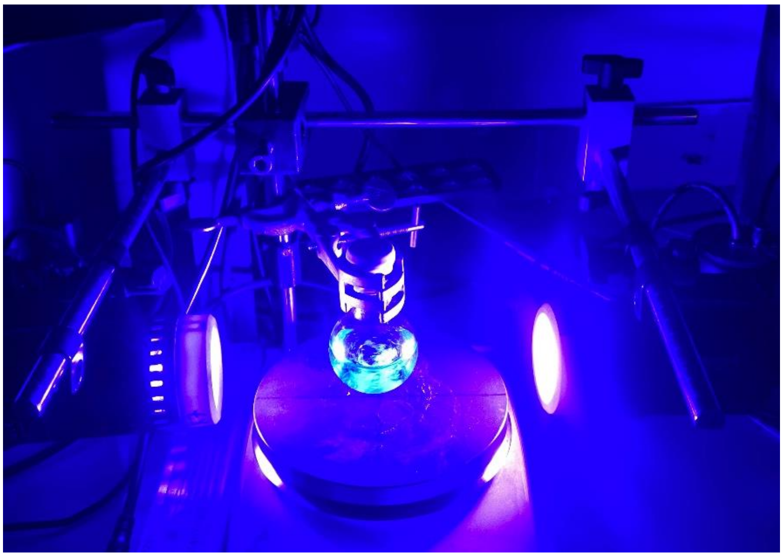

Figure F2. Standard photoreactor setup (440 nm, 2 x 40 W).

3. Synthesis of enamine derivatives

3.1 General procedure for the synthesis of enamine derivatives

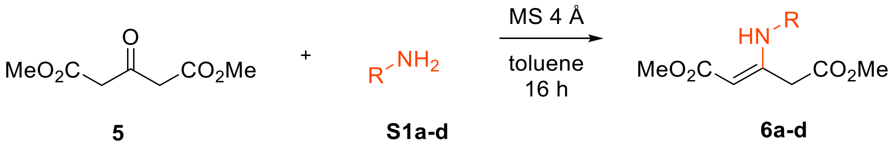

To the flask with activated molecular sieves (4 Å) a solution of dimethyl 3-oxopentanedioate (1.0 equiv., 20 mmol, 2.9 mL) and an amine (1.2 equiv.) in toluene (10 mL, c = 2.0 M for ketone) were added, then stirred at room temperature 16 h. The crude mixture was filtered by a plug of silica gel, next the organic layer was concentrated *in vacuo* and purified by column chromatography using hexanes/AcOEt to afford the final product.

Flash program: time: 30 min; column: silica 24 g; flow rate: 40 mL/min; peak hold on.

Entry	Time (min)	Hexane (%)	EtOAc (%)
1	0	100	0
2	5	100	0
3	15	20	80
4	20	20	80
5	25	40	60
6	27	40	60
7	30	0	100

3.2 Characterization of enamines

dimethyl 3-(benzylamino)pent-2-enedioate (6a)

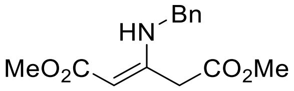

Synthesized according to the general procedure (20.0 mmol scale).

Yield: 3.48 g (66%) as a mixture of *Z* and *E* diastereoisomers: 80:20, yellow oil from flash column chromatography (hexanes/AcOEt, 80:20 (v/v)).

m.p. 47-51 ºC; **^{1}H NMR** ($CDCl_3$, 500 MHz): major (Z): δ = 8.91 (s, 1H), 7.37–7.30 (m, 3H), 7.28–7.25 (m, 2H), 4.61 (s, 1H), 4.44 (d, *J* = 6.3 Hz, 2H), 3.67 (s, 3H), 3.64 (s, 3H), 3.21 (s, 2H); minor (E): δ = 7.37–7.30 (m, 3H), 7.28–7.25 (m, 2H), 5.14 (s, 1H), 4,78 (s, 1H), 4.23 (d, *J* = 5.1 Hz, 2H), 3.96 (s, 2H), 3.73 (s, 3H), 3.62 (s, 3H) ppm; **^{13}C NMR** ($CDCl_3$, 126 MHz): major: δ = 170.9, 169.2, 156.9, 138.4, 129.0, 127.7, 127.0, 85.3, 52.6, 50.5, 47.2, 38.8; minor: δ = 170.9, 169.1, 154.2, 136.9, 129.0, 128.0, 127.8, 85.4, 52.4, 50.4, 47.9, 36.8 ppm.

NMR spectra are in agreement with the reported one[4].

dimethyl-3-(phenylamino)pent-2-enedioate (6b)

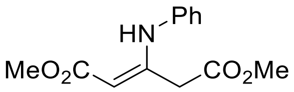

Synthesized according to the general procedure (20.0 mmol scale).

Yield: 3.68 g (70%) as a mixture of *Z* and *E* diastereoisomers: 88:12, yellow oil from flash column chromatography (hexanes/AcOEt, 80:20 (v/v)).

^{1}H NMR ($CDCl_3$, 600 MHz): major (Z): δ = 10.26 (s, 1H), 7.33 (t, *J* = 7.9 Hz, 2H), 7.21 (t, *J* = 7.5 Hz, 1H), 7.11 (d, *J* = 7.8 Hz, 2H), 4.78 (s, 1H), 3.70 (s, 3H), 3.61 (s, 3H), 3.29 (s, 2H); minor (E): δ = 7.33 (t, *J* = 7.9 Hz, 2H), 7.21 (t, *J* = 7.5 Hz, 1H), 7.11 (d, *J* = 7.8 Hz, 2H), 5.27 (s, 1H), 4.09 (s, 2H), 3.78 (s, 3H), 3.61 (s, 3H) ppm; **^{13}C NMR** ($CDCl_3$, 151 MHz): δ = 170.7, 169.4, 154.8,
138.7, 129.4, 126.2, 125.8, 87.4, 52.5, 50.7, 38.4 ppm; **HRMS** (ESI): *m/z* calcd for: $C_{13}H_{15}NO_4+Na^{+\cdot}$: 272.0899 $[M+Na]^+$; found: 272.0900.
GC Chromatogram: t_r = 2.67 min (> 99% purity).

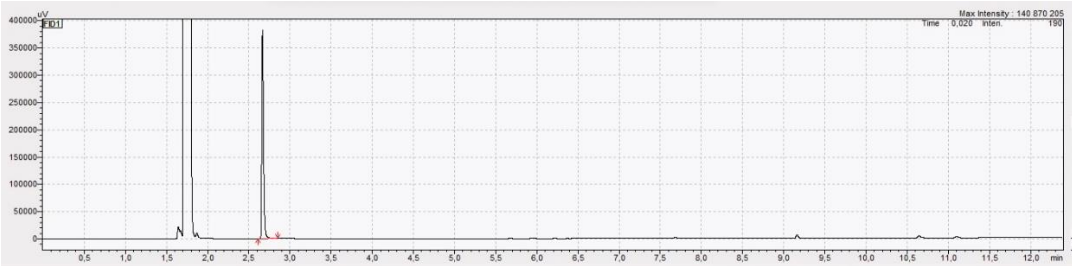

dimethyl 3-(cyclopropylamino)pent-2-enedioate (6c)

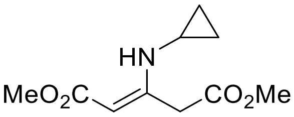

Synthesized according to the general procedure (20.0 mmol scale).
Yield: 3.88 g (91%) as a mixture of *Z* and *E* diastereoisomers: 82:18, white solid from flash column chromatography (hexanes/AcOEt, 80:20 (v/v)).
m.p. 66-69 ºC; **^{1}H NMR** ($CDCl_3$, 500 MHz): major (Z): δ = 8.52 (s, 1H) 4.56 (s, 1H), 3.74 (s, 3H), 3.62 (s, 3H), 3.41 (s, 2H), 2.60–2.55 (m, 1H), 0.77–0.73 (m, 2H), 0.61–0.58 (m, 2H); minor (E): δ = 5.16 (s, 1H), 5.08 (s, 1H), 3.86 (s, 2H), 3.71 (s, 3H), 3.64 (s, 3H), 2.40–2.37 (m, 1H), 0.77–0.73 (m, 2H), 0.55–0.52 (m, 2H) ppm;**^{13}C NMR** ($CDCl_3$, 126 MHz): major: δ = 170.7, 169.6, 158.6, 84.9, 52.6, 50.4, 39.0, 24.7, 8.0; minor: δ = 171.2, 169.1, 155.3, 86.6, 52.4, 50.5, 36.4, 24.9, 7.5 ppm. **HRMS** (ESI): *m/z* calcd for $C_{10}H_{15}NO_4+Na^+$: 236.0899 $[M+Na]^+$; found: 236.0903.
GC Chromatogram: t_r = 5.91 min (> 99% purity).

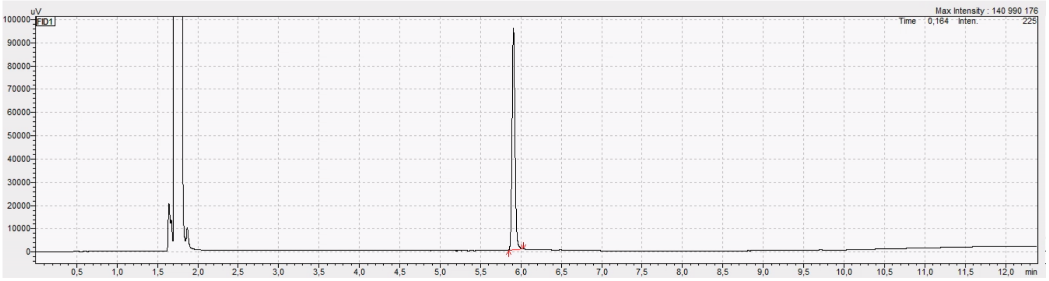

dimethyl 3-(propylamino)pent-2-enedioate (6d)

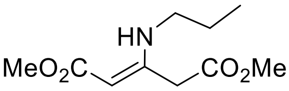

Synthesized according to the general procedure (20.0 mmol scale).
Yield: 3.35 g (78%) as a mixture of *Z* and *E* diastereoisomers: 80:20, colorless oil from flash column chromatography (hexanes/AcOEt, 80:20 (v/v)).

^{1}H NMR ($CDCl_3$, 600 MHz): major: δ = 8.52 (s, 1H), 4.47 (s, 1H), 3.70 (s, 3H), 3.60 (s, 3H), 3.20 (s, 2H), 3.14 (td, *J* = 7.0, 5.8 Hz, 2H), 1.58 (tq, *J* = 14.5, 7.3 Hz, 2H), 0.94 (td, *J* = 7.4, 1.6 Hz, 3H); minor: δ = 4.94 (s, 1H), 4.66 (s, 1H), 3.88 (s, 2H), 3.69 (s, 3H), 3.59 (s, 3H), 2.97 (td, *J* = 7.1, 5.0 Hz, 2H), 1.58 (tq, *J* = 14.5, 7.3 Hz, 2H), 0.94 (td, *J* = 7.4, 1.6 Hz, 3H) ppm; **^{13}C NMR** ($CDCl_3$, 151 MHz): major: δ = 170.9, 169.3, 157.0, 83.7, 52.5, 50.2, 45.1, 38.7, 23.6, 11.4; minor: δ = 171.0, 169.2, 154.6, 83.9, 52.3, 50.3, 45.2, 36.7, 21.6, 11.6 ppm. **HRMS** (ESI): *m/z* calcd for $C_{10}H_{17}NO_4+H^+$: 216.1236 $[M+H]^+$; found: 216.1240.

GC Chromatogram: t_r = 5.78 min (> 99% purity).

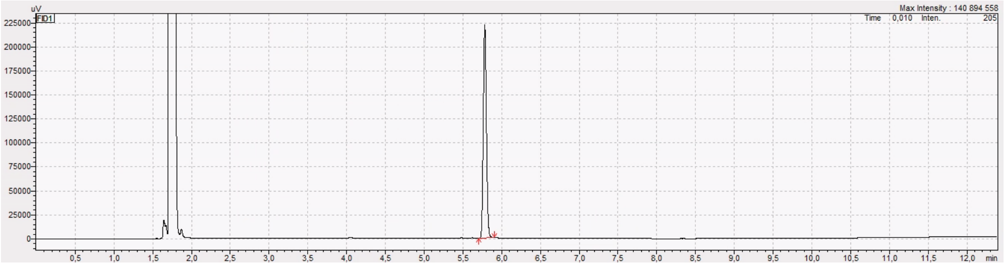

4. Synthesis of diazo compounds

4.1 Diazo transfer reactions – optimization studies

4.1.1 Model reaction

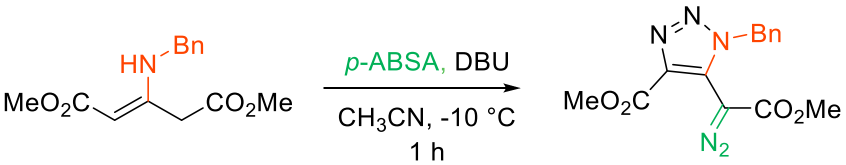

Reaction conditions: To a solution of substrate **6a** (0.1 mmol) in dry MeCN (0.5 mL, *c* = 0.2 M for **6a**), DBU (1.25 equiv., 0.12 mmol) and *p*-ABSA (1.5 equiv., 0.15 mmol) were added, Ar atmosphere, -10 °C, 1 h.

4.1.2 Control experiments

**Entry**	**Deviations from the optimized conditions**	**Product 7a [%][a]**
1	none	34
2	no base	0
3	no *p*-ABSA	0
4	air atmosphere	traces
5	MeCN (not dry)	traces

Reaction conditions: To a solution of substrate **6a** (0.1 mmol) in dry MeCN (0.5 mL, *c* = 0.2 M for **6a**), DBU (1.6 equiv., 0.16 mmol) and *p*-ABSA (1.5 equiv., 0.15 mmol) were added; Ar atmosphere; 0 °C for 30 min; [a] yield determined by NMR.

4.1.3 Screening of solvents

**Entry**	**Dry Solvent**	**Product 7a [%][a]**
1	MeCN	34
2	acetone	-
3	DCM	9
4	THF	-
5	hexane	undefined product

Reaction conditions: To a solution of substrate **6a** (0.1 mmol) in dry solvent (0.5 mL, *c* = 0.2 M for **6a**), DBU (1.6 equiv., 0.16 mmol) and *p*-ABSA (1.5 equiv., 0.15 mmol) were added; Ar atmosphere; 0 °C for 30 min; [a] yield determined by NMR.

4.1.4 Screening of amines

Entry	Amines	Product 7a [%][a]
1	DBU	34
2	DBU[b]	
3	Et_3N[c]	-
4	DBN	undefined product
5	pyrrolidine[c]	-
6	DBN then DBU[d]	15
7	DMAP[c]	-
8	TBD[c]	udefined product

Reaction conditions: To a solution of substrate **6a** (0.1 mmol) in dry MeCN (0.5 mL, *c* = 0.2 M for **6a**), DBU (1.6 equiv., 0.16 mmol) and *p*-ABSA (1.5 equiv., 0.15 mmol) were added; Ar atmosphere 0 °C 30 min; [a] yield determined by NMR; [b] Reaction performed in dry conditions; [c] Reaction was run at 0 °C, 30 min, then at RT for 16 h; [d] after 30 min at 0 °C DBU (1.6 equiv.) was added, and the reaction was run for another 30 min.

4.1.5 The influence of the amount of an amine

Entry	Amount of DBU (equiv.)	Product 7a [%][a]
1	1.6	34
2	1.25	36
3	2.0	16

Reaction conditions: To a solution of substrate **6a** (0.1 mmol) in dry MeCN (0.5 mL, *c* = 0.2 M for **6a**), DBU, and *p*-ABSA (1.5 equiv., 0.15 mmol) were added; Ar atmosphere; 0 °C for 30 min; [a] yield determined by NMR.

4.1.6 Selection of diazo transfer reagents

Entry	Diazo transfer reagent	Product 7a [%][a]
1	*p*-ABSA	36
2	TsN_3[b]	-
3	TfN_3[b]	-

Reaction conditions: To a solution of substrate **6a** (0.1 mmol) in dry MeCN (0.5 mL, *c* = 0.2 M for **6a**), DBU (1.25 equiv., 0.125 mmol) and diazo transfer reagent (1.5 equiv., 0.15 mmol) were added; Ar atmosphere; 0 °C for 30 min; [a] yield determined by NMR; [b] Reaction was run at 0 °C for 30 min, then at RT for 16 h.

4.1.7 Influence of the amount of a p-ABSA

Entry	Diazo transfer reagent (equiv.)	Product 7a [%][a]
1	1.0	35
2	1.5	36
3	2.1	36

Reaction conditions: To a solution of substrate **6a** (0.1 mmol) in dry MeCN (0.5 mL, *c* = 0.2 M for **6a**), DBU (1.25 equiv., 0.125 mmol) and *p*-ABSA were added; Ar atmosphere; 0 °C, 30 min; [a] yield determined by NMR; [b] Reaction was run at 0 °C for 30 min, then at RT for 16 h.

4.1.8 Optimization of the reaction temperature

Entry	Temperature [°C]	Product 7a [%][a]
1	RT[b]	13
2	-20[c]	16
3	0	36
4	0[c]	36
5	-10[c]	38

Reaction conditions: To a solution of substrate **6a** (0.1 mmol) in dry MeCN (0.5 mL, *c* = 0.2 M for **6a**), DBU (1.25 equiv., 0.125 mmol) and *p*-ABSA (1.5 equiv., 0.15 mmol) were added; 0 °C for 30 min; [a] yield determined by NMR; [b]3h; [c]To a solution of *p*-ABSA (1.5 equiv., 0.15 mmol) in dry MeCN (0.25 mL, *c* = 0.6 M for *p*-ABSA) at -10 °C, DBU (1.25 equiv., 0.125 mmol) and solution of substrate **6a** (0.1 mmol) in dry MeCN (0.25 mL, *c* = 0.4 M for **6a**) were added; Ar atmosphere; 1 h.

4.1.9 Optimization of the concentration of diazo reagent

Entry	Concentration [mol/dm^3]	Product 7a [%][a]
1	0.05	13
2	0.2	38
4	0.4	19

Reaction conditions: To a solution of *p*-ABSA (1.5 equiv., 0.15 mmol) in dry MeCN (0.25 mL, *c* = 0.6 M for *p*-ABSA) at -10 °C, DBU (1.25 equiv., 0.125 mmol) and solution of condensation substrate **6a** (1 equiv., 0.1 mmol) in dry MeCN (0.25 mL, *c* = 0.4 M for **6a**) were added; Ar atmosphere; 1 h; [a] yield determined by NMR.

4.1.10 Screening of other different reaction conditions

Entry	Diazo transfer reagent	Base	Solvent	Temperature	Product 7a
1	TsN_3	KF on Alumina	THF	0 °C, RT	-
2	*p*-ABSA	KF on Alumina	THF	0 °C	-
3	TsN_3	KF	DCM	RT Dark	-
4	*p*-ABSA	KF	DCM	RT Dark	-
5	TsN_3	K_2CO_3	MeCN	RT	-
6	*p*-ABSA	K_2CO_3	MeCN	RT	-
7	TsN_3	4 Å MS	acetone	RT	-
8	TsN_3	4 Å MS	THF	RT	-
9	TsN_3	4 Å MS	hexane	RT	undefined product

Reaction conditions: To a solution of substrate **6a** (0.1 mmol) in dry solvent (0.6 mL, *c* = 0.2 M for **6a**), a base (1.25 equiv., 0.125 mmol) and a diazo transfer reagent (1.5 equiv., 0.15 mmol) were added; 16 h, Ar atmosphere.

4.2 General procedure for the synthesis of diazo compounds

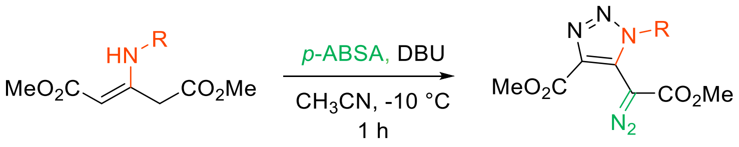

6a-d **7a-d**

To a solution of *p*-ABSA (1.5 equiv., 5.7 mmol, 1.37 g) in dry MeCN (5 mL, *c* = 1.14 M) at -10 °C, DBU (1.25 equiv., 4.75 mmol, 0.71 mL) and a solution of enamine **6a-d** (1 equiv., 3.8 mmol) in dry MeCN (5 mL, c = 0.76 M) were added. The reaction mixture was stirred at -10 °C for 1 h, then MeCN was evaporated *in vacuo*. To the reaction mixture saturated $NaHCO_3$ and DCM were added, layers were separated, and the aqueous layer was washed with DCM. The combined organic layers are dried over sodium sulfate, filtrated, concentrated in vacuo, and purified by flash column chromatography using hexanes/AcOEt to afford the final product.

Flash program: time: 30 min; column: silica 12 g; flow rate: 30 mL/min; automatic peak hold on.

Entry	Time (min)	EtOAc (%)	Hexane (%)
1	0	0	100
2	5	0	100
3	15	20	80
4	20	20	80
5	25	40	60
6	27	40	60
7	30	100	0

4.3 Characterization of diazo compounds

methyl 1-benzyl-5-(1-diazo-2-methoxy-2-oxoethyl)-1H-1,2,3-triazole-4-carboxylate (7a)

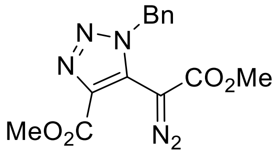

Molecular Weight: 315,2890

Synthesized from compound **6a** (3.8 mmol scale) according to the general procedure.

Yield: 455 mg (38%), yellow oil from flash column chromatography (hexanes/AcOEt, 80:20 (v/v)).

^{1}H NMR ($CDCl_3$, 500 MHz): δ = 7.36–7.33 (m, 3H), 7.16–7.15 (m, 2H), 5.65 (s, 2H), 3.97 (s, 3H), 3.82 (s, 3H) ppm; **^{13}C NMR** ($CDCl_3$, 151 MHz): δ = 163.6, 161.1, 136.9, 134.0, 129.1, 128.8, 127.7, 127.5, 53.9, 53.5, 52.8, 52.5 ppm; **HRMS** (ESI): *m/z* calcd for $C_{14}H_{13}N_5O_4+Na^+$: 338.0865 $[M+Na]^+$; found: 338.0873; **IR** (cm^{-1}): 2123, 1709, 1462, 1439.

methyl 5-(1-diazo-2-methoxy-2-oxoethyl)-1-phenyl-1H-1,2,3-triazole-4-carboxylate (7b)

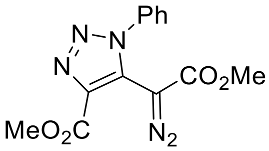

Synthesized from compound **6b** (3.8 mmol scale) according to the general procedure.
Yield: 364 mg (32%), yellow solid from flash column chromatography (hexanes/AcOEt, 50:50 (v/v))
^{1}H NMR ($CDCl_3$, 500 MHz): δ = 7.56–7.50 (m, 5H), 4.02 (s, 3H), 3.48 (s, 3H) ppm; **^{13}C NMR** ($CDCl_3$, 126 MHz): δ = 163.1, 161.1, 136.7, 136.4, 130.2, 129.8, 128.1, 124.3, 52.7, 52.6 ppm. **HRMS** (ESI): *m/z* calcd for $C_{13}H_{11}N_5O_4+Na^+$: 324.0709 $[M+Na]^+$; found: 324.0713; **IR** (cm^{-1}): 2126, 1713, 1498, 1448.

methyl 1-cyclopropyl-5-(1-diazo-2-methoxy-2-oxoethyl)-1H-1,2,3-triazole-4-carboxylate (7c)

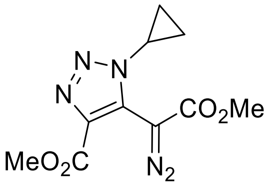

Synthesized from compound **6c** (3.8 mmol scale) according to the general procedure.
Yield: 352 mg (35%), yellow solid from flash column chromatography (hexanes/AcOEt, 50:50 (v/v)).
^{1}H NMR ($CDCl_3$, 500 MHz): δ = 3.97 (s, 3H), 3.86 (s, 3H), 3.70–3.67 (m, 1H), 1.36–1.33 (m, 2H), 1.20–1.15 (m, 2H) ppm; **^{13}C NMR** ($CDCl_3$, 126 MHz): δ = 163.4, 161.0, 136.0, 129.4, 52.8, 52.4, 31.3, 7.0 ppm; **HRMS** (ESI): *m/z* calcd for $C_{10}H_{11}N_5O_4+Na^+$: 288.0709 $[M+Na]^+$; found: 288.0711; **IR** (cm^{-1}): 2125, 1710, 1438, 1455.

methyl 5-(1-diazo-2-methoxy-2-oxoethyl)-1-propyl-1H-1,2,3-triazole-4-carboxylate (7d)

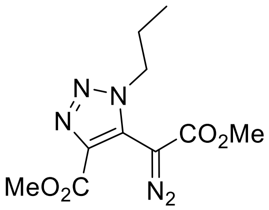

Synthesized from compound **6d** (3.8 mmol scale) according to the general procedure.
Yield: 345 mg (34%), yellow solid from flash column chromatography (hexanes/AcOEt, 50:50 (v/v)).
^{1}H NMR ($CDCl_3$, 500 MHz): δ = 4.28–4.25 (m, 2H), 3.98 (s, 3H), 3.85 (s, 3H), 2.06–1.98 (m, 2H), 0.96 (t, J = 7.4 Hz, 3H) ppm; **^{13}C NMR** ($CDCl_3$, 126 MHz) δ = 163.6, 161.2, 136.2, 127.5, 52.8, 52.4, 51.4, 22.8, 11.2 ppm; **HRMS** (ESI): *m/z* calcd for $C_{10}H_{13}N_5O_4+Na^+$: 290.0865 $[M+Na]^+$; found: 290.0864; **IR** (cm^{-1}): 2124, 1709, 1462, 1430.

5. Photochemical reactions

5.1 Optimization studies for the model reaction of diazo reagent 7a in DCM

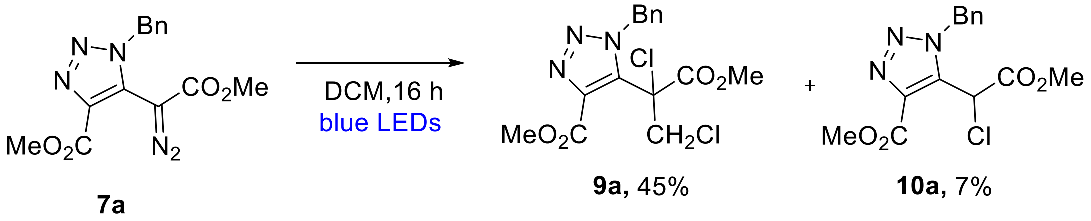

Reaction conditions: Solution of diazo compound **7a** (0.16 mmol) in dry DCM (4.3 mL, c = 0.04 M), 16 h, blue LED (450 nm, 7 W).

5.1.1 Background experiments

Entry	Deviations from optimized conditions	Major product 9a [%][a]
1	none	45
2	no light	0
3	air atmosphere	45
4	Ar atmosphere	traces
5	DCM (not dry)	traces[b]

Reaction conditions: Solution of diazo compound **7a** (0.16 mmol) in dry DCM (4.3 mL, c = 0.04 M), 16 h, blue LED (450 nm, 7 W); [a] yield determined by NMR; [b] We observed in the crude mixture the OH insertion to diazo compound.

5.1.2 Optimization of the reaction time

Entry	Time [h]	Major product 9a [%][a]
1	8	traces
2	16	45
3	24	45

Reaction conditions: Solution of diazo compound **7a** (0.16 mmol) in dry DCM (4.3 mL, c = 0.04 M), blue LED (450 nm, 7 W); [a] yield determined by NMR.

5.1.3 The influence of light on the model photoreaction

Entry	Power of photoreactor [W]	Major product 9a [%][a]
1	3.5	20
2	5	32
3	7	45
4	12	40
5	25	38
6	2 x 40	37

Reaction conditions: Solution of diazo compound **7a** (0.16 mmol) in dry DCM (4.3 mL, c = 0.04 M), 16 h, blue LED (450 nm); [a] yield determined by NMR.

5.2 Reactions of diazo compounds 7a with different alkyl chloride solvents

A glass vial equipped with a stirring bar and sealed with an aluminum cap with a rubber septum was charged with a diazo compound **7a-d** (0.16 mmol) in DCM (4.3 mL, c = 0.04 M). The reaction mixture was placed in a photoreactor and irradiated with blue LED (450 nm, 7 W) for 16 h. After that time, the crude reaction mixture was concentrated *in vacuo* and purified by flash column chromatography using hexanes/AcOEt to afford the corresponding product.

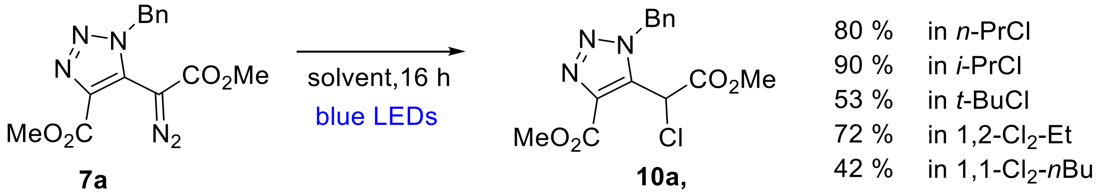

Reaction conditions: Solution of diazo compound **7a** (0.16 mmol) in solvent (4.3 mL, c = 0.04 M), 16 h, blue LED (450 nm, 7 W).

5.3 Reaction of diazo compound 7a with DCM on a 1 mmol scale

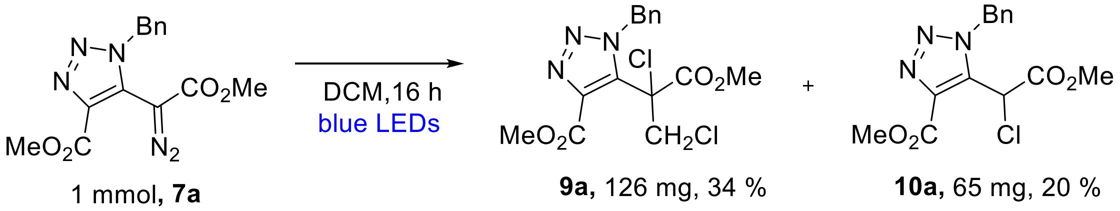

Reaction conditions: Solution of diazo compound **7a** (1 mmol, 315 mg) in dry DCM (26.9 mL, c = 0.04 M) was placed in a photoreactor and irradiated with blue-LED (440 nm, 2 x 40 W) for 16 h. After that time, the crude reaction mixture was concentrated *in vacuo* and purified by flash column chromatography using hexanes/AcOEt to afford the corresponding products **9a** (126 mg, 34 % yield) and **10a** (65 mg, 20 % yield).

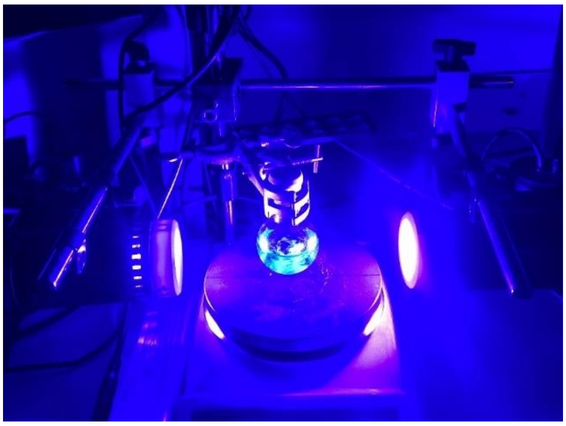

Figure S2. Set up for photochemical reaction diazo compound **7a** with DCM

Comment: *The* ***9a*****:*****10a*** *ratio obtained in the reaction on a bigger scale is slightly different from the one obtained previously, which we attributed to the use of a different light source- blue LED*

(440 nm, 2 x 40 W) versus blue LED (450 nm, 7 W). TAK 120 photoreactor is not suitable for performing reactions on a bigger scale.

5.3 General procedure for photochemical reactions in various solvents

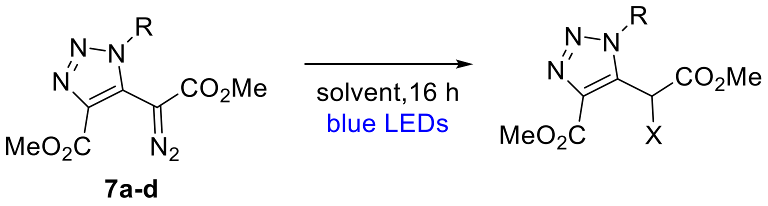

A glass vial equipped with a stirring bar and sealed with an aluminum cap with a rubber septum was charged with a diazo compound **7a-d** (0.16 mmol) in a solvent (4.3 mL, c = 0.04 M). The reaction mixture was placed in a photoreactor and irradiated with blue LED (450 nm, 7 W) for 16 h. After that time, the crude reaction mixture was concentrated *in vacuo* and purified by flash column chromatography using hexanes/AcOEt to afford the corresponding product.
Flash program: time: 40 min; column: silica 4g; flow rate: 13 mL/min; automatic peak holds on.

Entry	Time (min)	EtOAc (%)	Hexane (%)
1	0	0	100
2	2	0	100
3	10	20	80
4	12	20	80
5	20	40	60
6	22	40	60
7	30	50	50
8	35	50	50
9	40	100	0

HPLC program: time: 60 min.

Entry	Time (min)	EtOAc (%)	Hexane (%)
1	0	5	95
2	2	5	95
3	10	20	80
4	12	20	80
5	20	40	60
6	22	40	60
7	30	45	55
8	40	45	55
7	45	50	50
8	50	50	50
9	60	70	30

5.2.1 Characterization of products generated in halogenated solvents

methyl 1-benzyl-5-(2,3-dichloro-1-methoxy-1-oxopropan-2-yl)-1H-1,2,3-triazole-4-carboxylate (9a)

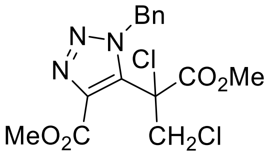

Synthesized from diazo compound **7a** (0.16 mmol scale) according to the general procedure.
Yield: 27 mg (45%), yellow oil from flash column chromatography (hexanes/AcOEt, 60:40 (v/v)).
^{1}H NMR ($CDCl_3$, 600 MHz): δ = 7.35–7.29 (m, 3H), 7.14–7.12 (m, 2H), 6.16 (d, J = 15.9 Hz, 1H), 5.99 (d, J = 15.9 Hz, 1H), 4.53 (d, J = 12.4 Hz, 1H), 4.28 (d, J = 12.4 Hz, 1H), 3.95 (s, 3H), 3.74 (s, 3H) ppm; **^{13}C NMR** ($CDCl_3$, 151 MHz) δ = 166.1, 162.3, 136.8, 136.6, 135.3, 128.9, 128.2, 126.8, 66.0, 55.3, 54.2, 52.9, 50.9 ppm; **HRMS** (ESI): *m/z* calcd for $C_{15}H_{15}N_3O_4Cl_2$+Na^+: 394.0337 $[M+Na]^+$; found 394.0350.
GC Chromatogram: t_r = 9.25 min (> 97% purity).

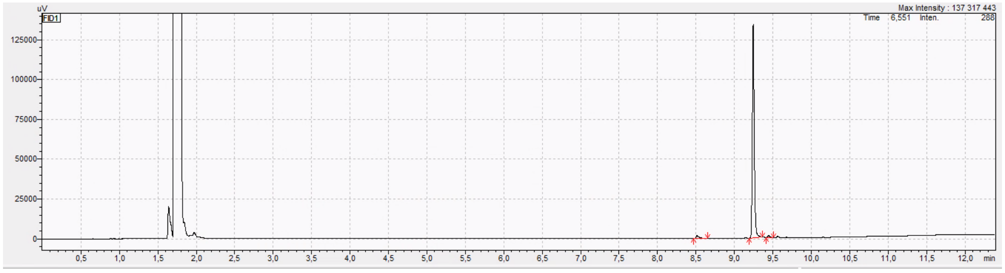

methyl 1-benzyl-5-(1-chloro-2-methoxy-2-oxoethyl)-1H-1,2,3-triazole-4-carboxylate (10a)

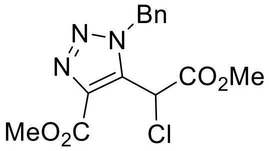

Isolated as a side product from the reaction of diazo **7a** with dry DCM (0.16 mmol scale).
Yield: 4 mg (7%), yellow oil from flash column chromatography (hexanes/AcOEt, 50:50 (v/v)).
^{1}H NMR ($CDCl_3$, 500 MHz): δ = 7.35–7.29 (m, 3H), 7.26–7.18 (m, 2H), 6.62 (s, 1H), 5.80 (d, J = 15.7 Hz, 1H), 5.63 (d, J = 15.7 Hz, 1H), 3.99 (s, 3H), 3.35 (s, 3H) ppm; **^{13}C NMR** ($CDCl_3$, 126 MHz) δ = 165.2, 161.6, 136.9, 136.1, 133.4, 129.0, 128.8, 127.7, 53.9, 53.3, 52.7, 46.3 ppm; **HRMS** (ESI): *m/z* calcd for $C_{14}H_{14}N_3O_4Cl$+Na^+: 346.0571 $[M+Na]^+$; found 346.0572.
HPLC Chromatogram: t_r = 17.67 min (> 96% purity).

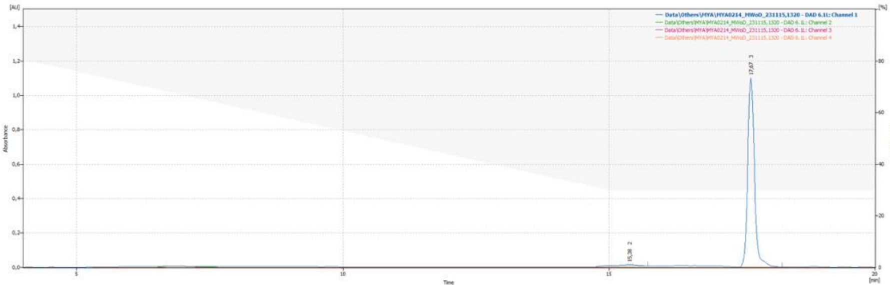

methyl 5-(2,3-dichloro-1-methoxy-1-oxopropan-2-yl)-1-phenyl-1H-1,2,3-triazole-4-carboxylate (9b)

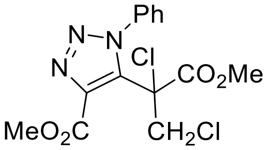

Synthesized from diazo compound **7b** (0.16 mmol scale) according to the general procedure.

Yield: 26 mg (45%), yellow oil from flash column chromatography (hexanes/AcOEt 60:40 (v/v)), repurified by HPLC using general condition (hexanes/AcOEt, 50:50 (v/v), t_r = 30.55 min).

^{1}H NMR ($CDCl_3$, 600 MHz): δ = 7.59–7.56 (m, 1H), 7.52–7.51 (m, 4H), 4.69 (d, J = 12.3 Hz, 1H), 4.26 (d, J = 12.3 Hz, 1H), 4.00 (s, 3H), 3.73 (s, 3H) ppm; **^{13}C NMR** ($CDCl_3$, 151 MHz): δ = 166.2, 162.2, 137.9, 136.9, 136.2, 131.0, 129.0, 128.1, 65.4, 54.3, 53.0, 50.3 ppm; **HRMS** (ESI): m/z calcd for $C_{14}H_{13}N_3O_4Cl_2+H^+$: 358.0361 $[M+H]^+$; found: 358.0359.

HPLC Chromatogram: t_r = 19.00 min (> 99% purity).

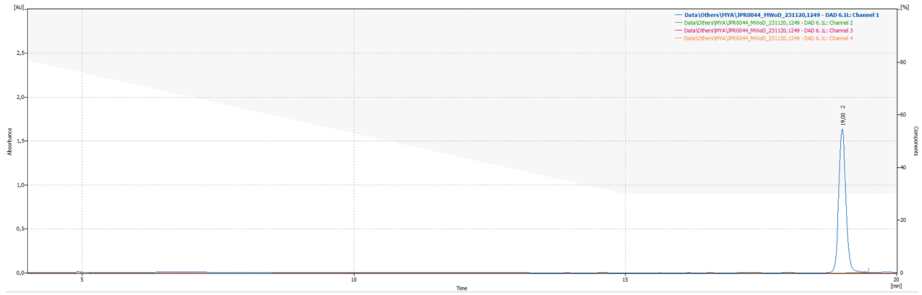

methyl 5-(1-chloro-2-methoxy-2-oxoethyl)-1-phenyl-1H-1,2,3-triazole-4-carboxylate (10b)

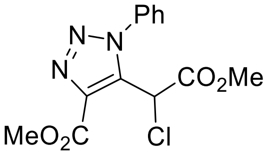

Isolated as a side product from the reaction of diazo **7b** with DCM (0.16 mmol scale).

Yield: 7 mg (14%), yellow oil from flash column chromatography (hexanes/AcOEt 50:50 (v/v)), repurified by HPLC (general condition, hexanes/AcOEt, 40:60 (v/v), t_r = 36.13 min).

^{1}H NMR ($CDCl_3$, 500 MHz): δ = 7.63–7.58 (m, 3H), 7.56–7.54 (m, 2H), 6.15 (s, 1H), 4.02 (s, 3H), 3.62 (s, 3H) ppm; **^{13}C NMR** ($CDCl_3$, 126 MHz): δ = 165.1, 161.3, 137.8, 136.9, 134.8, 131.3, 129.9, 126.2, 54.1, 52.7, 46.9 ppm; **HRMS** (ESI): m/z calcd for $C_{13}H_{12}N_3O_4Cl+H^+$: 310.0595 $[M+H]^+$; found: 310.0592.

HPLC Chromatogram: t_r = 17.13 min (> 98% purity).

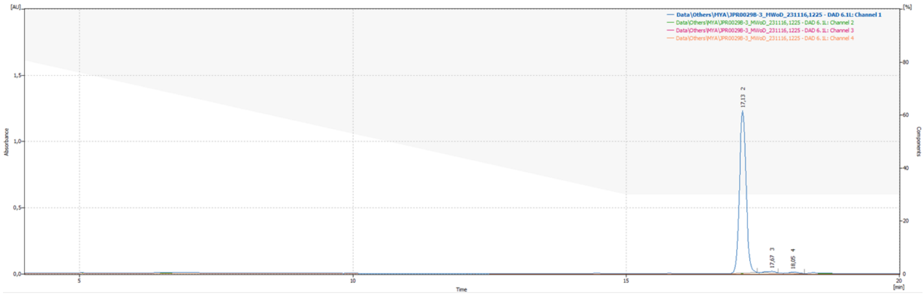

methyl 1-cyclopropyl-5-(2,3-dichloro-1-methoxy-1-oxopropan-2-yl)-1H-1,2,3-triazole-4-carboxylate (9c)

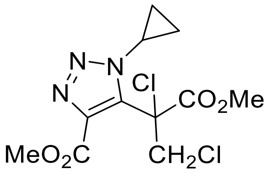

Synthesized from diazo compound **7c** (0.16 mmol scale) according to the general procedure.
Yield: 24 mg (46%), yellow oil, flash column chromatography (hexanes/AcOEt, 60:40 (v/v)).
^{1}H NMR ($CDCl_3$, 600 MHz): δ = 4.64 (d, *J* = 12.3 Hz, 1H), 4.45 (d, *J* = 12.4 Hz, 1H), 4.12 (tt, *J* = 7.3, 4.0 Hz, 1H), 3.92 (s, 3H), 3.81 (s, 3H), 1.95-1.88 (m, 1H), 1.44–1.38 (m, 1H), 1.31–1.21 (m, 2H) ppm; **^{13}C NMR** ($CDCl_3$, 126 MHz): δ = 166.4, 162.3, 137.7, 136.9, 66.1, 54.2, 52.8, 51.2, 34.7, 10.4, 8.9 ppm; **HRMS** (ESI): *m/z* calcd for $C_{11}H_{13}N_3O_4Cl_2+H^+$: 322.0361 $[M+H]^+$; found: 322.0362.
HPLC Chromatogram: t_r = 17.88 min (> 95% purity).

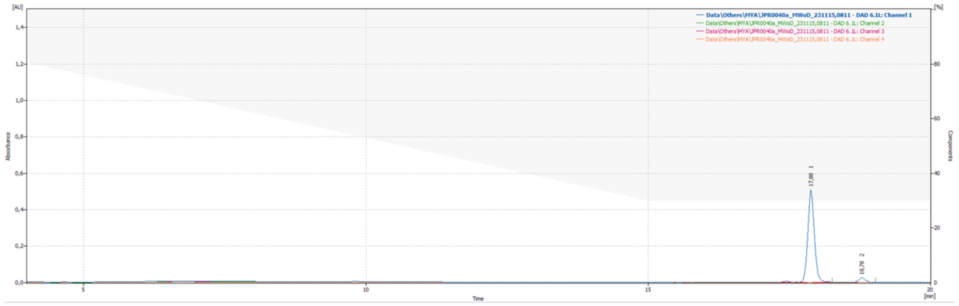

methyl 5-(1-chloro-2-methoxy-2-oxoethyl)-1-cyclopropyl-1H-1,2,3-triazole-4-carboxylate (10c)

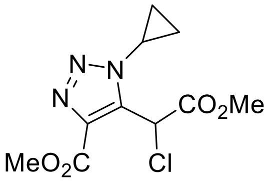

Isolated as a side product Isolated as a side product from the reaction of diazo compound **7c** with dry DCM (0.16 mmol scale).
Yield: 4 mg (9%), yellow oil from flash column chromatography (hexanes/AcOEt, 50:50 (v/v)).
^{1}H NMR ($CDCl_3$, 500 MHz): δ = 6.65 (s, 1H), 3.98 (s, 3H), 3.84 (s, 3H), 3.74 (tt, *J* = 7.4, 3.6 Hz, 1H), 1.68–1.63 (m, 1H), 1.34–1.26 (m, 2H), 1.24–1.16 (m. 1H) ppm; **^{13}C NMR** ($CDCl_3$, 151 MHz): δ = 165.7, 161.5, 137.6, 137.0, 54.3, 52.6, 46.5, 31.3, 7.8, 7.2 ppm; **HRMS** (ESI): *m/z* calcd for $C_{10}H_{12}N_3O_4Cl+Na^+$: 296.0410 $[M+Na]^+$; found: 296.0410.
GC Chromatogram: t_r = 7.56 min (> 95% purity).

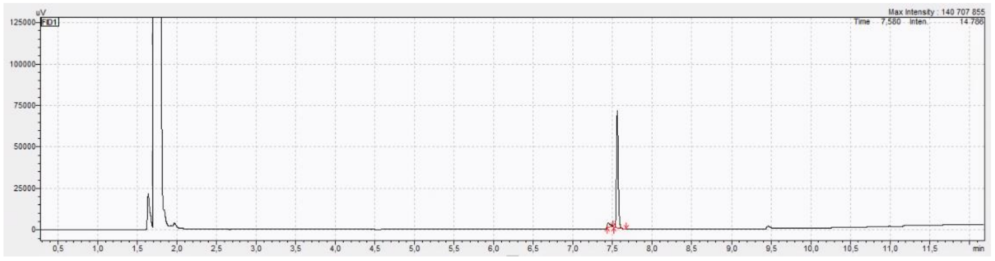

methyl 5-(2,3-dichloro-1-methoxy-1-oxopropan-2-yl)-1-propyl-1H-1,2,3-triazole-4-carboxylate (9d)

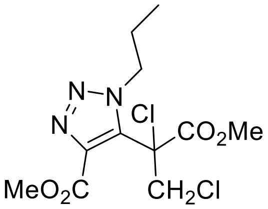

Synthesized from diazo compound **7d** (0.16 mmol scale) according to the general procedure.
Yield: 23 mg (45%), yellow oil, flash column chromatography (hexanes/AcOEt, 60:40 (v/v)).
^{1}H NMR ($CDCl_3$, 500 MHz): δ = 4.85 (ddd, *J* = 13.8, 8.7, 6.3 Hz, 1H), 4.64 (ddd, *J* = 13.8, 8.9, 7.0 Hz, 1H), 4.56 (d, *J* = 12.5 Hz, 1H), 4.41 (d, *J* = 12.5 Hz, 1H), 3.93 (s, 3H), 3.80 (s, 3H), 2.12–2.07 (m, 2H), 1.03 (t, *J* = 7.4 Hz, 3H) ppm; **^{13}C NMR** ($CDCl_3$, 126 MHz): δ = 166.3, 162.5, 136.2, 136.1, 66.4, 54.2, 53.9, 52.8, 51.2, 24.5, 11.2 ppm; **HRMS** (ESI): *m/z* calcd for $C_{11}H_{15}N_3O_4Cl_2+H^+$: 324.0518 $[M+H]^+$; found: 324.0521.
HPLC Chromatogram: t_r = 18.70 min (> 97% purity).

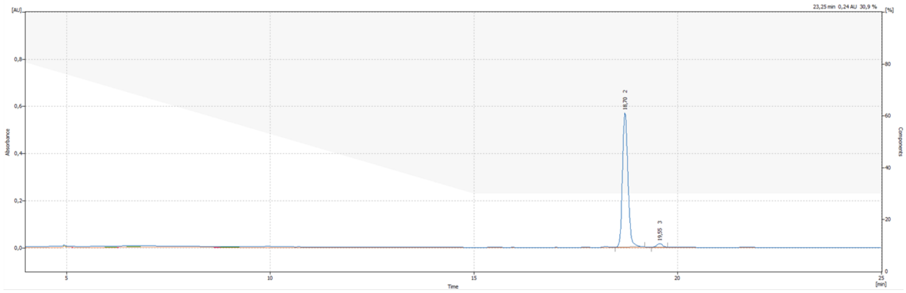

methyl 5-(1-chloro-2-methoxy-2-oxoethyl)-1-propyl-1H-1,2,3-triazole-4-carboxylate (10d)

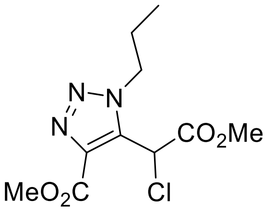

Isolated as a side product Isolated as a side product from the reaction of diazo compound **7d** with dry DCM (0.16 mmol scale).
Yield: 4 mg (9%), yellow oil from flash column chromatography (hexanes/AcOEt, 50:50 (v/v)).
^{1}H NMR ($CDCl_3$, 600 MHz): δ = 6.64 (s, 1H), 4.36 (t, *J* = 7.6 Hz, 2H), 3.98 (s, 3H), 3.82 (s, 3H), 2.10–1.98 (m, 2H), 1.00 (t, *J* = 7.4 Hz, 3H) ppm; **^{13}C NMR** ($CDCl_3$, 151 MHz): δ = 166.0, 161.8, 136.4, 135.5, 54.3, 52.6, 51.6, 46.0, 23.2, 11.2 ppm; **HRMS** (ESI): *m/z* calcd for $C_{10}H_{14}N_3O_4Cl+H^+$: 276.0751 $[M+H]^+$; found: 276.0753.
GC Chromatogram: t_r = 7.25 min (> 98% purity).

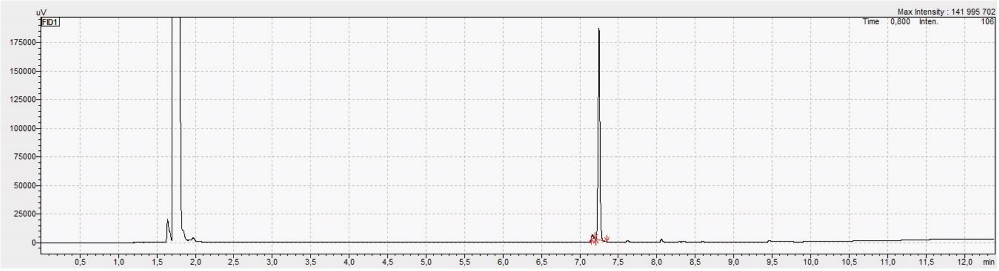

methyl 1-benzyl-5-(1,1,2-trichloro-3-methoxy-3-oxopropan-2-yl)-1H-1,2,3-triazole-4-carboxylate (11)

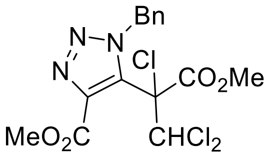

Synthesized from diazo compound **7a** (0.16 mmol scale) according to the general procedure in dry chloroform.

Yield: 8 mg (12%), yellow oil from flash column chromatography (hexanes/AcOEt, 60:40 (v/v)), repurified by HPLC (general condition, hexanes/AcOEt 60:40 (v/v), t_r = 24.75 min).

^{1}H NMR ($CDCl_3$, 500 MHz): δ = 7.36–7.29 (m, 3H), 7.18 (s, 1H), 7.11–7.08 (m, 2H), 6.08 (d, *J* = 15.9 Hz, 1H), 5.98 (d, *J* = 15.9 Hz, 1H), 4.00 (s, 3H), 3.59 (s, 3H) ppm; **^{13}C NMR** ($CDCl_3$, 126 MHz): δ = 164.0, 162.4, 137.9, 134.7, 134.3, 128.9, 128.4, 127.0, 73.9, 71.7, 55.3, 54.7, 53.3 ppm; **HRMS** (ESI): *m/z* calcd for $C_{15}H_{14}N_3O_4Cl_3+H^+$: 406.0128 $[M+H]^+$; found: 406.0121.

HPLC Chromatogram: t_r = 20.47 min (> 99% purity).

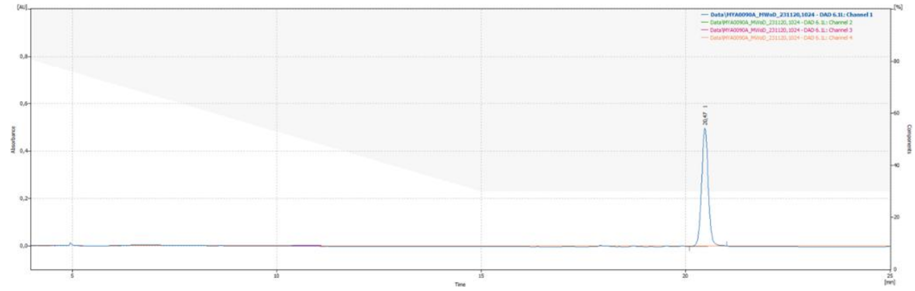

methyl 1-benzyl-5-(1-bromo-2-methoxy-2-oxoethyl)-1H-1,2,3-triazole-4-carboxylate (12)

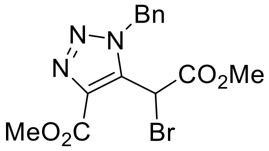

Synthesized from diazo compound **7a** (0.16 mmol scale) according to the general procedure in dry *n*-butyl bromide.

Yield: 41 mg (71%), yellow oil, flash column chromatography (hexanes/AcOEt,, 60:40 (v/v)), repurified by HPLC using general condition (hexanes/AcOEt 40:60 (v/v), t_r = 42.45 min).

^{1}H NMR ($CDCl_3$, 500 MHz): δ = 7.36–7.30 (m, 3H), 7.23–7.21 (m, 2H), 6.64 (s, 1H), 5.79 (d, *J* = 15.7 Hz, 1H), 5.66 (d, *J* = 15.7 Hz, 1H), 3.99 (s, 3H), 3.43 (s, 3H);) ppm; **^{13}C NMR** ($CDCl_3$, 126 MHz): δ = 165.1, 161.7, 136.42, 136.38, 133.5, 129.0, 128.8, 127.8, 54.1, 53.4, 52.7, 32.4 ppm; **HRMS** (ESI): *m/z* calcd for $C_{14}H_{14}N_3O_4Br+H^+$: 368.0246 $[M+H]^+$; found: 368.0244.

HPLC Chromatogram: t_r = 17.92 min (> 96% purity).

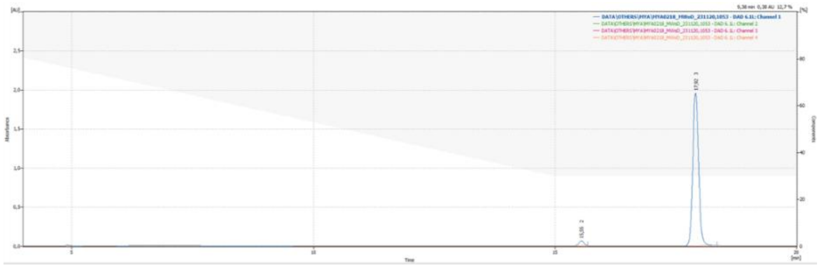

methyl 1-benzyl-5-(3-methoxy-3-oxoprop-1-en-2-yl)-1H-1,2,3-triazole-4-carboxylate (13)

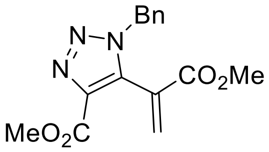

Synthesized from diazo compound **7a** (0.16 mmol scale) according to the general procedure in dry CH_2Br_2.

Yield: 12 mg (25%), colorless oil from flash column chromatography (hexanes/AcOEt, 60:40 (v/v)), repurified by HPLC using general condition (hexanes/AcOEt 20:80 (v/v), t_r = 41.20 min).

^{1}H NMR ($CDCl_3$, 600 MHz): δ = 7.34–7.30 (m, 3H), 7.15–7.13 (m, 2H), 6.77 (s, 1H), 5.75 (s, 1H), 5.50 (s, 2H), 3.89 (s, 3H), 3.60 (s, 3H) ppm; **^{13}C NMR** ($CDCl_3$, 151 MHz): δ = 164.0, 161.2, 138.3, 137.1, 134.6, 134.3, 129.1, 128.8, 128.0, 127.8, 52.8, 52.7, 52.3; **HRMS** (ESI): *m/z* calcd for $C_{13}H_{13}N_3O_4$+Na^+: 324.0960 $[M+Na]^+$; found: 324.0961.

5.2.2 Characterization of products generated in non-halogenated solvents

methyl 1-benzyl-5-(2-methoxy-2-oxo-1-(prop-1-en-2-yloxy)ethyl)-1H-1,2,3-triazole-4-carboxylate (14a)

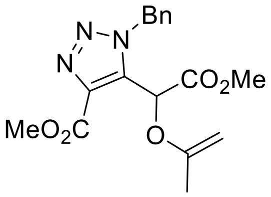

Synthesized from diazo compound **7a** (0.16 mmol scale) according to the general procedure in dry acetone.

Yield: 30 mg (55%), white oil from flash column chromatography (hexanes/AcOEt, 60:40 (v/v)).

^{1}H NMR ($CDCl_3$, 600 MHz): δ = 7.32–7.27 (m, 3H), 7.15–7.13 (m, 2H), 6.65 (s, 1H), 5.83 (d, *J* = 15.4 Hz, 1H), 5.65 (d, *J* = 15.4 Hz, 1H), 4.00 (s, 5H), 3.45 (s, 3H), 1.70 (s, 3H) ppm; **^{13}C NMR** ($CDCl_3$, 151 MHz) δ = 166.8, 161.9, 157.5, 137.6, 136.0, 134.4, 128.8, 128.4, 127.5, 85.2, 67.2, 53.4, 53.1, 52.6, 20.4 ppm; **HRMS** (ESI): *m/z* calcd for $C_{17}H_{19}N_3O_5$+K^+: 384.0962 $[M+K]^+$; found: 384.0959.

HPLC Chromatogram: t_r = 7.30 min (> 95% purity).

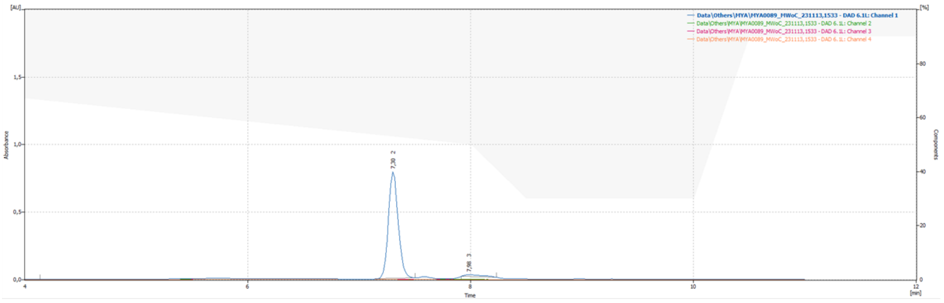

methyl 5-(2-methoxy-2-oxo-1-(prop-1-en-2-yloxy)ethyl)-1-phenyl-1H-1,2,3-triazole-4-carboxylate (14b)

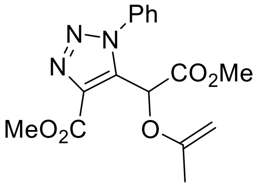

Synthesized from diazo compound **7b** (0.16 mmol scale) according to the general procedure in dry acetone.

Yield: 19 mg (36%), yellow oil, flash column chromatography (hexanes/AcOEt, 50:50 (v/v)).
^{1}H NMR ($CDCl_3$, 500 MHz): δ = 7.55–7.45 (m, 5H), 6.47 (s, 1H), 4.01 (s, 3H), 3.98 (d, *J* = 2.2 Hz, 1H), 3.85 (d, *J* = 3.3 Hz, 1H), 3.59 (s, 3H), 1.63 (s, 3H) ppm; **^{13}C NMR** ($CDCl_3$, 126 MHz) δ = 166.9, 161.7, 157.6, 137.7, 137.1, 135.7, 130.7, 129.3, 126.3, 85.0, 67.2, 53.1, 52.6, 20.4 ppm; **HRMS** (ESI): *m/z* calcd for $C_{16}H_{17}N_3O_5$+Na^+: 354.1066 $[M+Na]^+$; found: 354.1068.
HPLC Chromatogram: t_r = 18.02 min (> 94% purity).

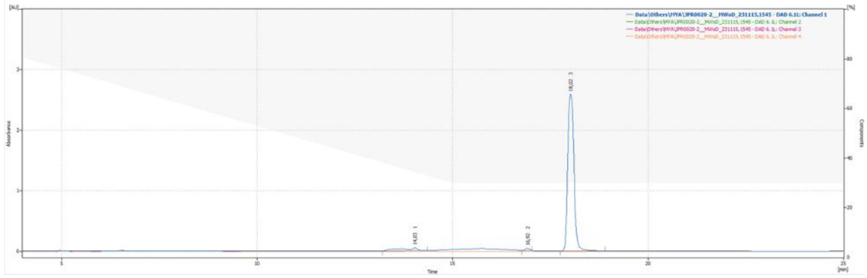

methyl 1-cyclopropyl-5-(2-methoxy-2-oxo-1-(prop-1-en-2-yloxy)ethyl)-1H-1,2,3-triazole-4-carboxylate (14c)

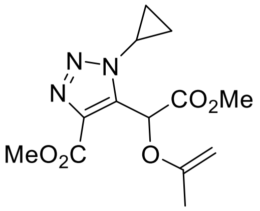

Synthesized from diazo compound **7c** (0.16 mmol) according to the general procedure in acetone.

Yield: 21 mg (45%), yellow oil, flash column chromatography (hexanes/AcOEt, 50:50 (v/v)).
m.p. 96-97 ºC; **^{1}H NMR** ($CDCl_3$, 600 MHz): δ = 6.67 (s, 1H), 4.05 (s, 2H), 3.98 (s, 3H), 3.92 (tt, *J* = 7.6, 3.9 Hz, 1H), 3.77 (s, 3H), 1.90 (s, 3H), 1.63–1.61 (m, 1H), 1.25–1.22 (m, 1H), 1.19–1.17 (m, 1H), 1.16–1.10 (m, 1H) ppm; **^{13}C NMR** ($CDCl_3$, 151 MHz): δ = 167.1, 161.9, 157.4, 137.8, 137.1, 85.3, 67.1, 53.3, 52.5, 32.0, 20.7, 7.8, 7.1 ppm; **HRMS** (ESI): *m/z* calcd for $C_{13}H_{17}N_3O_5$+Na^+: 318.1066 $[M+Na]^+$; found: 318.1069.
GC Chromatogram: t_r = 7.76 min (> 99% purity).

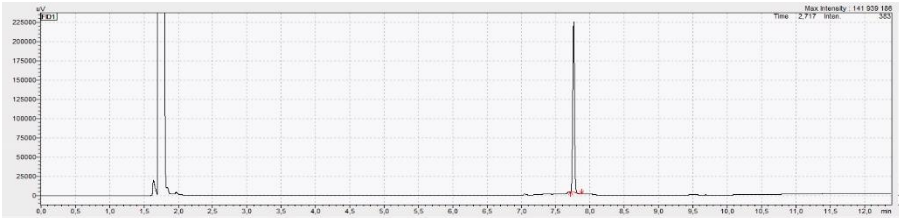

methyl 5-(2-methoxy-2-oxo-1-(prop-1-en-2-yloxy)ethyl)-1-propyl-1H-1,2,3-triazole-4-carboxylate (14d)

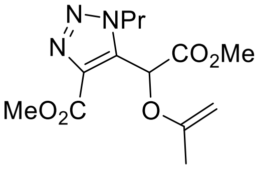

Synthesized from diazo compound **7d** (0.16 mmol scale) according to the general procedure in dry acetone.

Yield: 21 mg (45%), yellow oil, flash column chromatography (hexanes/AcOEt, 50:50 (v/v)).

^{1}H NMR ($CDCl_3$, 600 MHz): δ = 6.63 (s, 1H), 4.48–4.43 (m, 1H), 4.41–4.37 (m, 1H), 4.03 (d, *J* = 3.3 Hz, 1H), 3.99 (d, *J* = 3.4 Hz, 1H), 3.97 (s, 3H), 3.72 (s, 3H), 1.96–1.90 (m, 2H), 1.87 (m, 3H), 0.95 (t, *J* = 7.5 Hz, 3H) ppm; **^{13}C NMR** ($CDCl_3$, 151 MHz): δ = 167.4, 161.9, 157.3, 136.9, 135.3, 85.2, 67.3, 53.3, 52.4, 51.7, 23.6, 20.6, 11.0 ppm; HRMS (APCI): *m/z* calcd for $C_{13}H_{18}N_3O_5$-H^+ : 296.1246 $[M$-$H^+]^-$found: 296.1252.

GC Chromatogram: t_r = 7.51 min (> 99% purity).

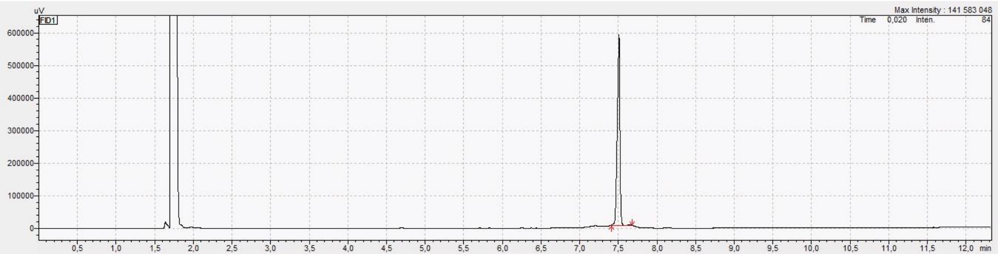

methyl 1-benzyl-5-(1,2-dimethoxy-2-oxoethyl)-1H-1,2,3-triazole-4-carboxylate (15a)

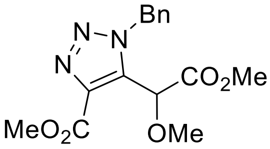

Synthesized from diazo compound **7a** (0.16 mmol scale) according to the general procedure in dry methanol.

Yield: 45 mg (88%), white solid from flash column chromatography (hexanes/AcOEt, 50:50 (v/v)).

m.p. 75-76 ºC; **^{1}H NMR** ($CDCl_3$, 600 MHz): δ = 7.33–7.28 (m, 3H), 7.22–7.21 (m, 2H), 5.97 (s, 1H), 5.74 (d, *J* = 15.2 Hz, 1H), 5.61 (d, *J* = 15.3 Hz, 1H), 3.99 (s, 3H), 3.37 (s, 3H), 3.35 (s, 3H) ppm; **^{13}C NMR** ($CDCl_3$, 151 MHz): δ = 167.7, 162.0, 138.2, 136.4, 134.2, 128.8, 128.5, 127.9, 71.7, 58.9, 53.3, 52.8, 52.5 ppm; **HRMS** (ESI): *m/z* calcd for $C_{15}H_{17}N_3O_5$+Na^+: 342.1066 $[M+Na]^+$; found: 342.1070.

GC Chromatogram: t_r = 9.15 min (> 99% purity).

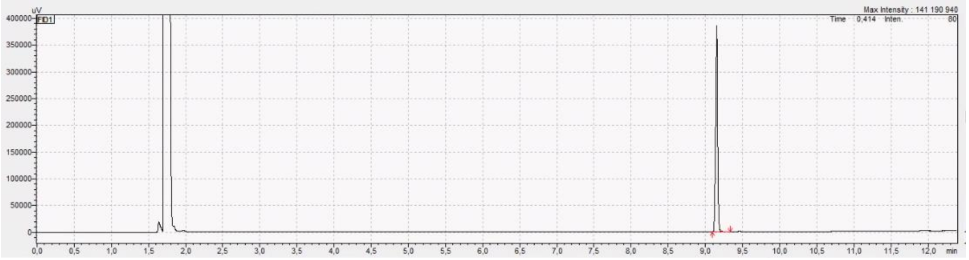

methyl 5-(1,2-dimethoxy-2-oxoethyl)-1-phenyl-1H-1,2,3-triazole-4-carboxylate (15b)

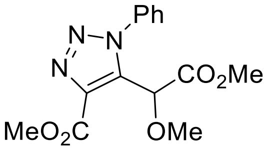

Synthesized from diazo compound **7b** (0.16 mmol scale) according to the general procedure in dry methanol.

Yield: 18 mg (36%), white solid from flash column chromatography (hexanes/AcOEt, 50:50 (v/v)).

m.p. 94-96 ºC; **^{1}H NMR** ($CDCl_3$, 600 MHz): δ = 7.56–7.51 (m, 5H), 5.81 (s, 1H), 4.01 (s, 3H), 3.54 (s, 3H), 3.41 (s, 3H) ppm; **^{13}C NMR** ($CDCl_3$, 151 MHz) δ 167.7, 161.9, 138.2, 137.1, 135.4, 130.7, 129.5, 125.9, 70.7, 59.1, 52.61, 52.58 ppm; **HRMS** (ESI): *m/z* calcd for $C_{14}H_{15}N_3O_5+Na^+$: 328.0909 [M+Na]$^+$; found: 328.0910.

GC Chromatogram: t_r = 8.79 min (> 99% purity).

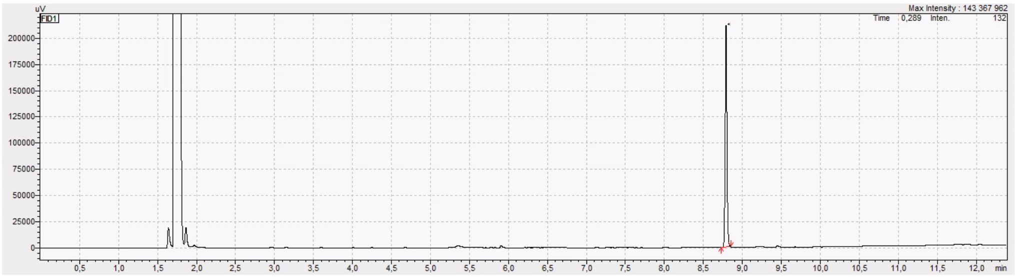

methyl 1-cyclopropyl-5-(1,2-dimethoxy-2-oxoethyl)-1H-1,2,3-triazole-4-carboxylate (15c)

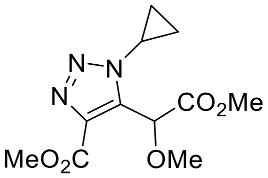

Synthesized from diazo compound **7c** (0.16 mmol scale) according to the general procedure in dry methanol.

Yield: 19 mg (45%), white solid from flash column chromatography (hexanes/AcOEt, 50:50 (v/v)).

m.p. 71-72 ºC; **^{1}H NMR** ($CDCl_3$, 600 MHz): δ = 6.01 (s, 1H), 3.98 (s, 3H), 3.83–3.78 (m, 1H), 3.77 (s, 3H), 3.49 (s, 3H), 1.76–1.72 (m, 1H), 1.19–1.15 (m, 1H), 1.10–1.06 (m, 2H) ppm; **^{13}C NMR** ($CDCl_3$, 151 MHz): δ = 168.2, 161.9, 138.4, 137.6, 71.3, 58.8, 53.1, 52.5, 31.4, 8.1, 6.4 ppm; **HRMS** (ESI): *m/z* calcd for $C_{11}H_{15}N_3O_5+Na^+$: 292.0909 [M+Na]$^+$; found: 292.0905

GC Chromatogram: t_r = 7.41 min (> 99% purity).

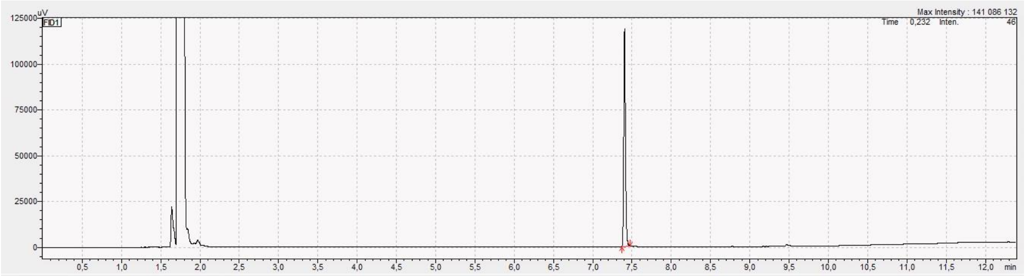

methyl 5-(1,2-dimethoxy-2-oxoethyl)-1-propyl-1H-1,2,3-triazole-4-carboxylate (15d)

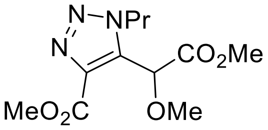

Synthesized from diazo compound **7d** (0.16 mmol scale) according to the general procedure in dry methanol.

Yield: 20 mg (45%), white solid from flash column chromatography (hexanes/AcOEt 50:50 (v/v)).

m.p. 42-43 ºC; **^{1}H NMR** ($CDCl_3$, 500 MHz): δ = 5.98 (s, 1H), 4.38–4.29 (m, 2H), 3.95 (s, 3H), 3.72 (s, 3H), 3.44 (s, 3H), 1.98–1.84 (m, 2H), 0.92 (t, *J* = 7.4 Hz, 3H) ppm; **^{13}C NMR** ($CDCl_3$, 126 MHz): δ = 168.3, 162.0, 137.5, 135.9, 71.7, 58.8, 53.1, 52.4, 51.4, 23.3, 11.0 ppm; **HRMS** (ESI): *m/z* calcd for $C_{11}H_{17}N_3O_5$+Na^+: 294.1066 [M+Na]$^+$; found: 294.1071.

GC Chromatogram: t_r = 7.12 min (> 99% purity).

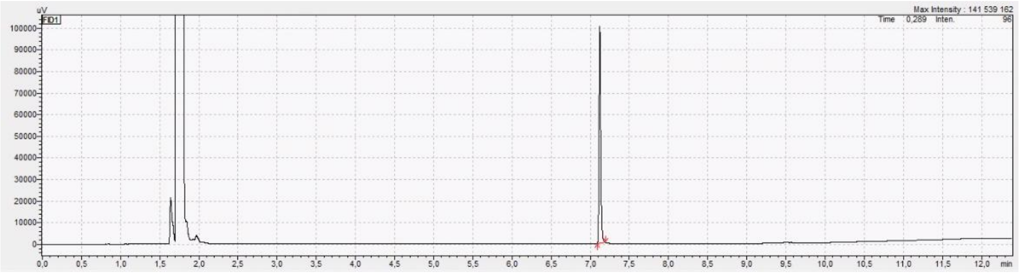

4-ethyl 1-methyl 2-(1-benzyl-4-(methoxycarbonyl)-1H-1,2,3-triazol-5-yl)succinate (16)

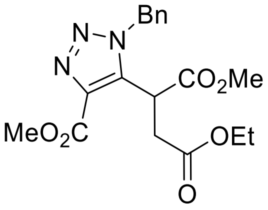

Synthesized from diazo compound **7a** (0.16 mmol scale) according to the general procedure in dry ethyl acetate.

Yield: 38 mg (63%), yellow oil from flash column chromatography (hexanes/AcOEt, 50:50 (v/v)).

^{1}H NMR ($CDCl_3$, 500 MHz): δ = 7.33–7.28 (m, 3H), 7.14–7.13 (m, 2H), 6.22 (s, 1H), 5.68 (s, 2H),4.64 (d, *J* = 3.4 Hz, 1H), 4.27 (d, *J* = 3.4 Hz, 1H), 3.98 (s, 3H), 3.61 (s, 3H), 3.59–3.57 (m, 2H), 1.08 (t, *J* = 7.0 Hz, 3H) ppm; **^{13}C NMR** ($CDCl_3$,126 MHz) δ = 170.0, 163.8, 157.8, 141.0, 137.2, 135.1, 128.6, 128.1, 127.1, 86.1, 75.7, 64.4, 53.6, 53.2, 53.1, 14.0 ppm; **HRMS** (ESI): *m/z* calcd for $C_{18}H_{21}N_3O_6$+H^+: 376.1509 [M+H]$^+$; found: 376.1514

GC Chromatogram: t_r = 10.40 min (> 95% purity).

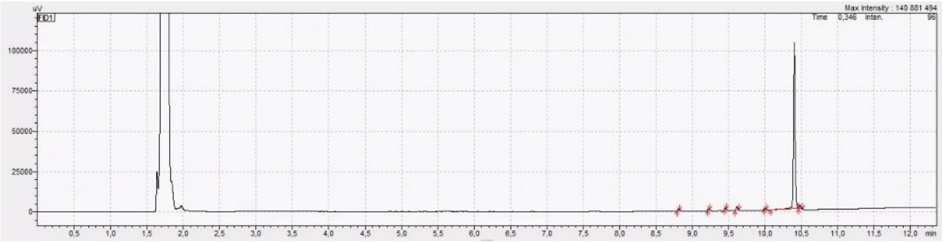

methyl 1-benzyl-5-(5-methoxy-2-methyloxazol-4-yl)-1H-1,2,3-triazole-4-carboxylate (17a)

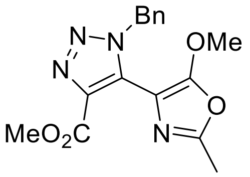

Synthesized from diazo compound **7a** (0.16 mmol scale) according to the general procedure in dry MeCN.

Yield: 26 mg (50%), yellow oil from flash column chromatography (hexanes/AcOEt, 50:50 (v/v)).

^{1}H NMR ($CDCl_3$, 600 MHz): δ = 7.26–7.25 (m, 3H), 7.11–7.10 (m, 2H), 5.70 (s, 2H), 3.91 (s, 3H), 3.72 (s, 3H), 2.42 (s, 3H) ppm; **^{13}C NMR** ($CDCl_3$, 151 MHz): δ = 161.3, 157.4, 152.7, 137.2, 134.8, 131.7, 128.7, 128.4, 127.8, 99.9, 59.6, 52.7, 52.1, 14.2 ppm; **HRMS** (ESI): *m/z* calcd for $C_{16}H_{16}N_4O_4$+Na^+: 351.1069 $[M+Na]^+$; 351.1073.

HPLC Chromatogram: t_r = 17.00 min (> 96% purity).

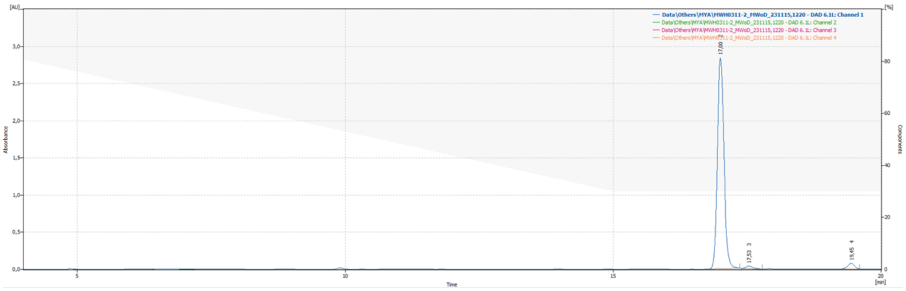

methyl 5-(5-methoxy-2-methyloxazol-4-yl)-1-phenyl-1H-1,2,3-triazole-4-carboxylate (17b)

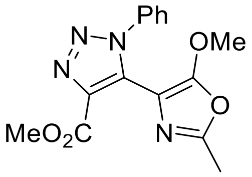

Synthesized from diazo compound **7b** (0.16 mmol scale) according to the general procedure in dry MeCN.

Yield: 24 mg (48%), yellow oil from flash column chromatography (hexanes/AcOEt, 50:50 (v/v)).

^{1}H NMR ($CDCl_3$, 600 MHz): δ = 7.50–7.45 (m, 5H), 3.96 (s, 3H), 3.77 (s, 3H), 2.34 (s, 3H) ppm; **^{13}C NMR** ($CDCl_3$, 151 MHz): δ = 161.2, 157.3, 152.7, 138.1, 136.3, 132.3, 129.8, 129.3, 125.2, 99.9, 59.6, 52.3, 14.3 ppm; **HRMS** (ESI): *m/z* calcd for $C_{15}H_{14}N_4O_4$+Na^+: 337.0913 $[M+Na]^+$; found: 337.0919.

GC Chromatogram: t_r = 9.55 min (> 98% purity).

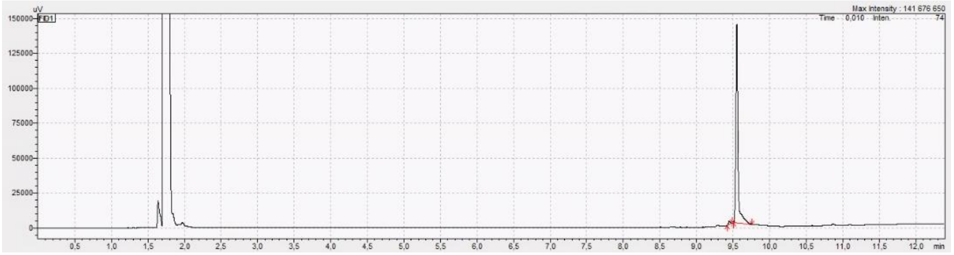

methyl 1-cyclopropyl-5-(5-methoxy-2-methyloxazol-4-yl)-1H-1,2,3-triazole-4-carboxylate (17c)

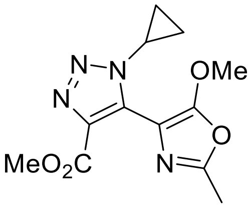

Synthesized from diazo compound **7c** (0.16 mmol scale) according to the general procedure in dry MeCN.
Yield: 19 mg (43%), yellow oil, flash column chromatography (hexanes/AcOEt, 50:50 (v/v)).
^{1}H NMR ($CDCl_3$, 600 MHz): δ = 3.99 (s, 3H), 3.91 (s, 3H), 3.83 (tt, J = 7.5, 3.9 Hz, 1H), 2.44 (s, 3H), 1.37–1.34 (m, 2H), 1.11–1.07 (m, 2H) ppm; **^{13}C NMR** ($CDCl_3$, 151 MHz) δ = 161.3, 157.4, 152.7, 137.1, 133.3, 100.2, 59.8, 52.1, 31.1, 14.4, 7.5 ppm; HRMS (APCI): *m/z* calcd for $C_{12}H_{14}N_4O_4$+H^+: 279.1093 $[M+H]^+$; found 279.1094.
GC Chromatogram: t_r = 8.45 min (> 99% purity).

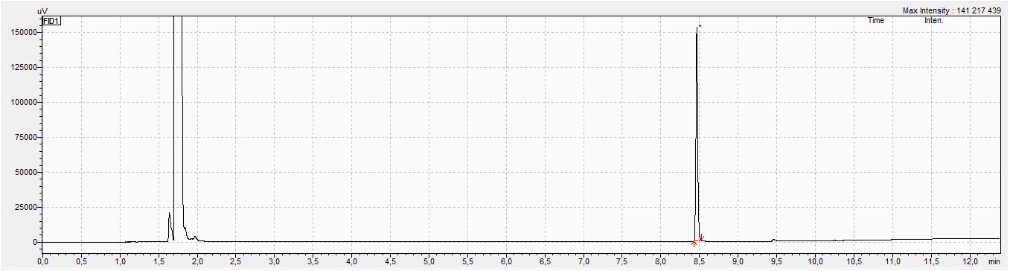

methyl 5-(5-methoxy-2-methyloxazol-4-yl)-1-propyl-1H-1,2,3-triazole-4-carboxylate (17d)

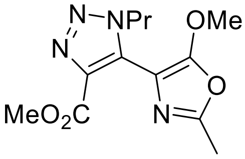

Synthesized from diazo compound **7d** (0.16 mmol scale) according to the general procedure in dry MeCN.
Yield: 19 mg (43%), yellow oil, flash column chromatography (hexanes/AcOEt, 50:50 (v/v)).
^{1}H NMR ($CDCl_3$, 600 MHz): δ = 4.40–4.37 (m, 2H), 3.98 (s, 3H), 3.91 (s, 3H), 2.42 (s, 3H), 1.92–1.86 (m, 2H), 0.89 (t, J = 7.4 Hz, 3H) ppm; **^{13}C NMR** ($CDCl_3$,151 MHz) δ = 161.4, 157.4, 152.7, 136.7, 131.5, 100.1, 59.7, 52.1, 50.7, 23.3, 14.3, 11.0 ppm; **HRMS** (ESI): *m/z* calcd for $C_{12}H_{16}N_4O_4$+Na^+: 303.1073 $[M+Na]^+$; found: 303.1069.
GC Chromatogram: t_r = 8.15 min (> 99% purity).

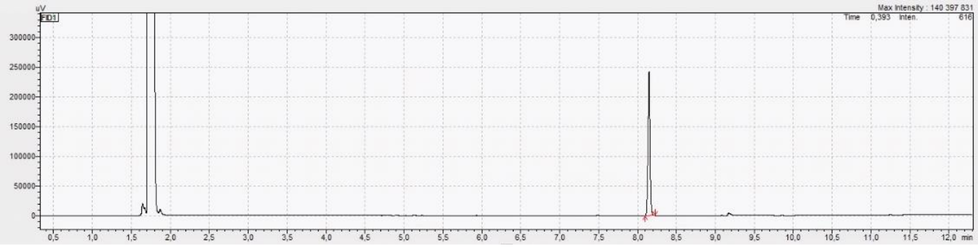

methyl 1-benzyl-5-(7-(methoxycarbonyl)bicyclo[4.1.0]hepta-2,4-dien-7-yl)-1H-1,2,3-triazole-4-carboxylate (18)

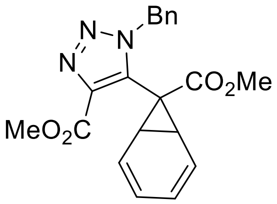

Synthesized from diazo compound **7a** (0.16 mmol scale) according to the general procedure in dry benzene.

Yield: 32 mg (55%), pale yellow solid from flash column chromatography (hexanes/AcOEt, 50:50 (v/v)).

m.p. 131-132 ºC; **^{1}H NMR** ($CDCl_3$, 500 MHz): δ = 7.35–7.30 (m, 3H), 7.29–7.25 (m, 2H), 6.22 (br, 2H), 5.90 (dd, *J* = 7.3, 2.9 Hz, 2H), 5.41 (br, 2H), 3.91 (s, 3H), 3.32 (s, 2H), 3.12 (s, 3H) ppm; **^{13}C NMR** ($CDCl_3$,126 MHz) δ = 173.2, 161.2, 139.7, 134.2, 134.0, 128.9, 128.7, 128.3, 125.6, 53.0, 52.8, 52.1, 38.2, 14.2 ppm; **HRMS** (ESI): *m/z* calcd for $C_{20}H_{19}N_3O_4+Na^+$: 388.1273 $[M+Na]^+$; found: 388.1274.

GC Chromatogram: t_r = 11.12 min (> 98% purity).

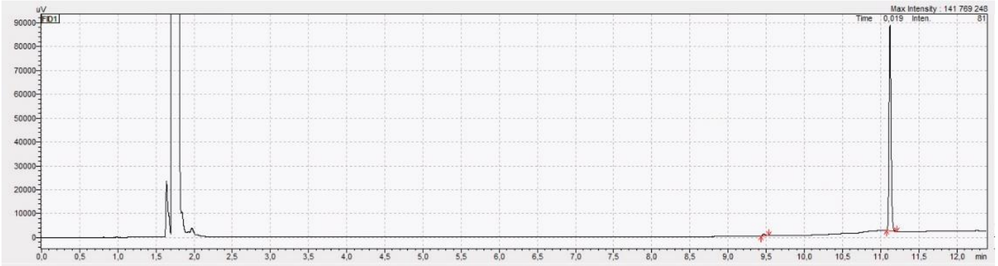

methyl 1-benzyl-5-(2-methoxy-2-oxo-1-(tetrahydrofuran-2-yl)ethyl)-1H-1,2,3-triazole-4-carboxylate (19)

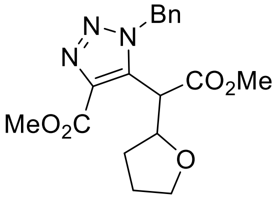

Synthesized from diazo compound **7a** (0.16 mmol scale) according to the general procedure in dry THF.

Yield: 17 mg (29%), yellow oil from flash column chromatography (hexanes/AcOEt, 60:40 (v/v)).

^{1}H NMR ($CDCl_3$, 500 MHz): δ = 7.34–7.28 (m, 3H), 7.14–7.12 (m, 2H), 5.96 (d, *J* = 14.8 Hz, 1H), 5.86 (d, *J* = 14.8 Hz, 1H), 3.91 (s, 3H), 3.79–3.72 (m, 1H), 3.61 (s, 3H), 3.61–3.54 (m, 1H), 2.40–2.37 (m, 1H), 2.19–2.09 (m, 1H), 1.44–1.35 (m, 3H), 1.13–1.04 (m, 1H) ppm; **^{13}C NMR** ($CDCl_3$, 126 MHz): δ = 168.7, 161.9, 141.5, 136.4, 129.1, 128.7, 128.1, 127.3, 78.0, 64.6, 55.9, 52.6, 52.5, 30.8, 23.8, 18.8 ppm; **HRMS** (ESI): *m/z* calcd for $C_{18}H_{21}N_3O_5+H^+$: 360.1559 $[M+H]^+$; found: 360.1556.

GC Chromatogram: t_r = 10.56 min (> 99% purity).

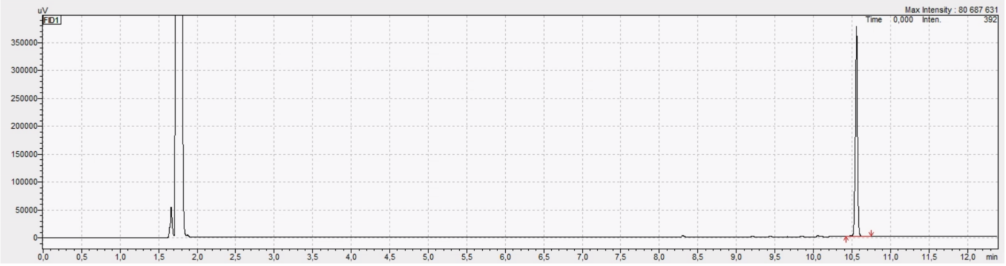

methyl 1-benzyl-5-(1-(but-3-en-1-yloxy)-2-methoxy-2-oxoethyl)-1H-1,2,3-triazole-4-carboxylate (20)

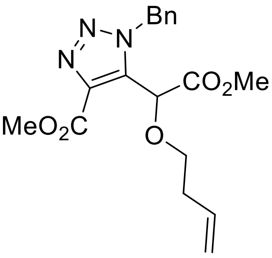

Synthesized from diazo compound **7a** (0.16 mmol scale) according to the general procedure in dry THF.

Yield: 7 mg (12%), yellow oil from flash column chromatography (hexanes/AcOEt, 60:40 (v/v)).

^{1}H NMR ($CDCl_3$, 500 MHz): δ = 7.33–7.26 (m, 3H), 7.20–7.18 (m, 2H), 6.07 (s, 1H), 5.75 (d, *J* = 15.4 Hz, 1H), 5.73–5.67 (m, 1H), 5.64 (d, *J* = 15.3 Hz, 1H), 5.06–5.01 (m, 2H), 3.99 (s, 3H), 3.78–3.74 (m, 1H), 3.47–3.43 (m, 1H), 3.34 (s, 3H), 2.35–2.22 (m, 2H) ppm; **^{13}C NMR** ($CDCl_3$, 126 MHz) δ = 168.0, 162.0, 137.7, 136.9, 134.4, 134.3, 128.7, 128.4, 127.8, 117.3, 71.1, 70.3, 53.3, 52.7, 52.5, 33.9 ppm; **HRMS** (ESI): *m/z* calcd for $C_{18}H_{21}N_3O_5+Na^+$: 382.1379 $[M+Na]^+$; found: 382.1385.

HPLC Chromatogram: t_r = 17.00 min (> 96% purity).

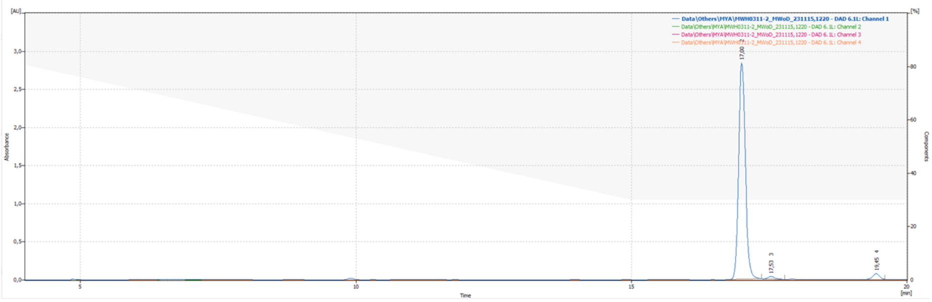

methyl 1-benzyl-5-(1-cyclohexyl-2-methoxy-2-oxoethyl)-1H-1,2,3-triazole-4-carboxylate (21)

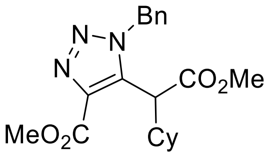

Synthesized from diazo compound **7a** (0.16 mmol scale) according to the general procedure in dry cyclohexane.

Yield: 37 mg (63%), yellow oil from flash column chromatography (hexanes/AcOEt, 60:40 (v/v)).

^{1}H NMR ($CDCl_3$, 500 MHz): δ = 7.34–7.29 (m, 3H), 7.19 (dd, *J* = 7.8, 1.7 Hz, 2H), 5.83 (d, *J* = 15.8 Hz, 1H), 5.74 (d, *J* = 15.8 Hz, 1H), 4.63 (d, *J* = 10.9 Hz, 1H), 3.96 (s, 3H), 3.52 (s, 3H), 1.99–1.96 (m, 1H), 1.89–1.86 (m, 1H), 1.68–1.62 (m, 1H), 1.50–1.46 (m, 1H), 1.34–1.30 (m, 1H), 1.14–1.08 (m, 1H), 1.01–0.95 (m, 2H), 0.87–0.85 (m, 1H), 0.74–0.72 (m, 1H), 0.61–0.51 (m, 1H) ppm; **^{13}C NMR** ($CDCl_3$,126 MHz) δ = 170.9, 162.2, 138.3, 137.8, 134.6, 128.9, 128.5, 127.3, 53.1, 52.4, 52.3, 46.2, 38.1, 32.4, 30.2, 25.8, 25.3 ppm; **HRMS** (ESI): *m/z* calcd for $C_{20}H_{25}N_3O_4$+Na^+: 394.1743 $[M+Na]^+$; found: 394.1742.

GC Chromatogram: t_r = 10.91 min (> 99% purity).

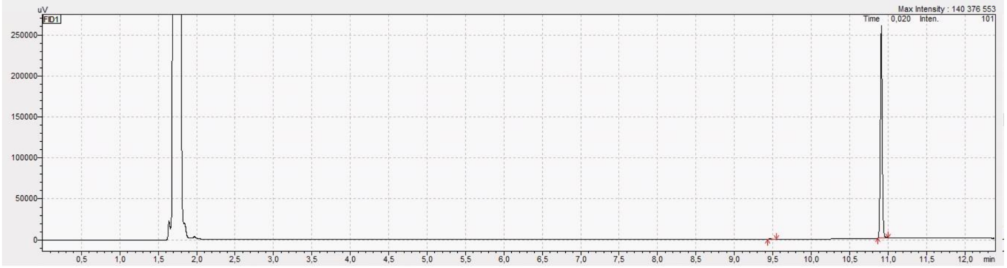

5.3 Insertion reactions

5.3.1 General procedure for photochemical – NH, OH or SH insertions

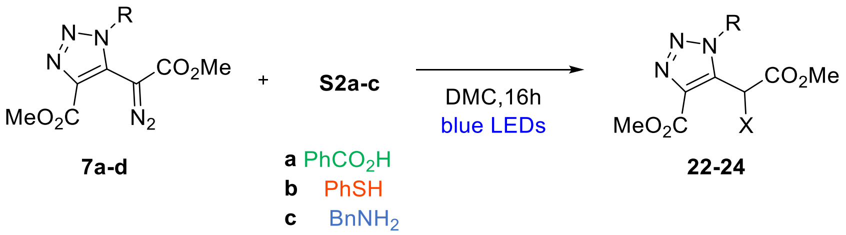

A glass vial equipped with a stirring bar and sealed with an aluminum cap with a rubber septum was charged with a diazo compound (1 equiv., 0.1 mmol) and X–H reagent **S2 a-c** (thiophenol, benzoic acid or benzylamine 10 equiv., 1 mmol) dissolved in dry dimethyl dicarbonate (2.0 mL, c = 0.05 M for diazo compound). The reaction mixture was placed in a photoreactor and irradiated with blue LED (450 nm, 7 W) for 16 h. After that time, the crude reaction mixture was concentrated *in vacuo* and purified by flash column chromatography using hexanes/AcOEt to afford the corresponding product.

Flash program: time: 40 min; column: silica 4g; flow rate: 13 mL/min; automatic peak hold on.

Entry	Time (min)	EtOAc (%)	Hexane (%)
1	0	0	100
2	2	0	100
3	10	20	80
4	12	20	80
5	20	40	60
6	22	40	60
7	30	50	50
8	35	50	50
9	40	100	0

HPLC program: time: 60 min.

Entry	Time (min)	EtOAc (%)	Hexane (%)
1	0	5	95
2	2	5	95
3	10	20	80
4	12	20	80
5	20	40	60
6	22	40	60
7	30	45	55
8	40	45	55
7	45	50	50
8	50	50	50
9	60	70	30

5.3.2 Characterization of insertion products

methyl 5-(1-(benzoyloxy)-2-methoxy-2-oxoethyl)-1-benzyl-1H-1,2,3-triazole-4-carboxylate (22a)

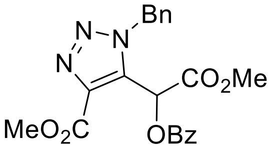

Synthesized from benzoic acid and diazo compound **7a** (0.1 mmol scale) according to the general procedure.

Yield: 25 mg (61%), colorless oil, flash column chromatography (hexanes/AcOEt, 50:50 (v/v)). **^{1}H NMR** ($CDCl_3$, 500 MHz): δ = 7.80–7.78 (m, 2H), 7.59–7.55 (m, 1H), 7.37 (t, *J* = 7.8 Hz, 2H), 7.33 (s, 1H), 7.32–7.28 (m, 3H), 7.15–7.13 (m, 2H), 5.86 (d, *J* = 15.8 Hz, 1H), 5.80 (d, *J* = 15.7 Hz, 1H), 4.01 (s, 3H), 3.53 (s, 3H) ppm; **^{13}C NMR** ($CDCl_3$, 126 MHz) δ = 166.1, 164.8, 161.2, 138.3, 134.7, 134.2, 134.0, 130.1, 129.1, 128.7, 128.6, 128.2, 127.1, 63.1, 53.4, 53.2, 52.6 ppm; **HRMS** (ESI): *m/z* calcd for $C_{21}H_{19}N_3O_6$+H^+: 410.1352 $[M+H]^+$; found: 410.1354.

GC Chromatogram: t_r = 12.12 min (> 98% purity).

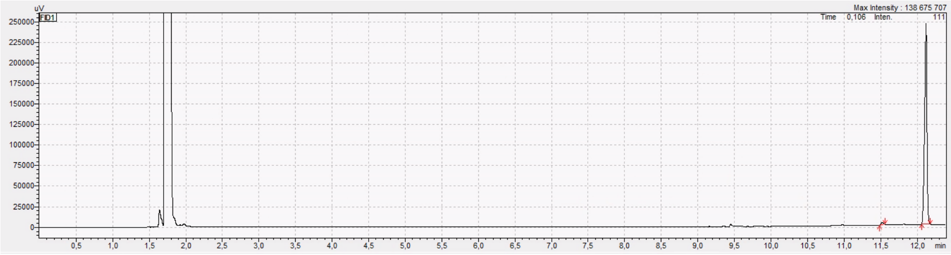

methyl 5-(1-(benzoyloxy)-2-methoxy-2-oxoethyl)-1-phenyl-1H-1,2,3-triazole-4-carboxylate (22b)

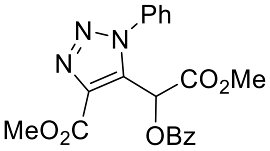

Synthesized from benzoic acid and diazo compound **7b** (0.1 mmol scale) according to the general procedure.

Yield: 25 mg (63%), colorless oil from flash column chromatography (hexanes/AcOEt 50:50 (v/v)), repurified by HPLC using general condition (hexanes/AcOEt, 50:50 (v/v), t_r = 35.07 min).

^{1}H NMR ($CDCl_3$, 500 MHz): δ = 7.92–7.90 (m, 2H), 7.62–7.57 (m, 4H), 7.55–7.53 (m, 2H), 7.43 (t, *J* = 7.9 Hz, 2H), 7.23 (s, 1H), 4.03 (s, 3H), 3.65 (s, 3H) ppm; **^{13}C NMR** ($CDCl_3$, 126 MHz) δ = 166.1, 164.6, 161.2, 138.3, 135.8, 135.3, 134.1, 131.1, 130.2, 129.8, 128.7, 128.4, 126.2, 63.1, 53.4, 52.7 ppm; **HRMS** (ESI): *m/z* calcd for $C_{20}H_{17}N_3O_6$+H^+: 396.1196 $[M+H]^+$; found: 396.1193.

GC Chromatogram: t_r = 11.70 min (> 95% purity).

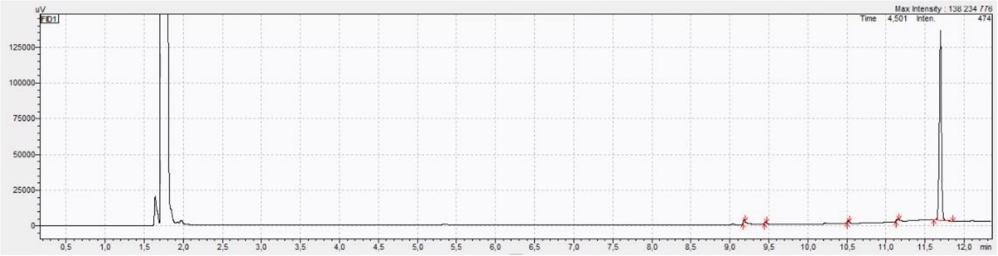

methyl 5-(1-(benzoyloxy)-2-methoxy-2-oxoethyl)-1-cyclopropyl-1H-1,2,3-triazole-4-carboxylate (22c)

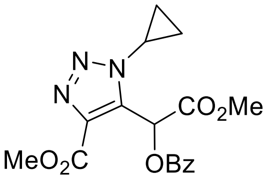

Synthesized from benzoic acid and diazo compound **7c** (0.1 mmol scale) according to the general procedure.

Yield: 18 mg (50%), colorless oil from flash column chromatography (hexanes/AcOEt, 50:50 (v/v)), repurified by HPLC using general condition (hexanes/AcOEt 50:50 (v/v), t_r = 33.27 min).

^{1}H NMR ($CDCl_3$, 500 MHz): δ = 8.07–8.05 (m, 2H), 7.63–7.60 (m, 1H), 7.48–7.46 (m, 2H), 7.45 (s, 1H), 3.98 (s, 3H), 3.87–3.83 (m, 1H), 3.82 (s, 3H), 1.63–1.57 (m, 1H), 1.38–1.33 (m, 1 H), 1.32–1.20 (m, 2H) ppm; **^{13}C NMR** ($CDCl_3$, 126 MHz) δ = 166.3, 164.9, 161.2, 138.2, 136.2, 134.1, 130.2, 128.8, 128.5, 63.0, 53.6, 52.5, 31.1, 7.4, 7.3 ppm; **HRMS** (ESI): *m/z* calcd for $C_{17}H_{17}N_3O_6+H^+$: 360.1196 $[M+H]^+$; found: 360.1201.

GC Chromatogram: t_r = 10.53 min (> 99% purity).

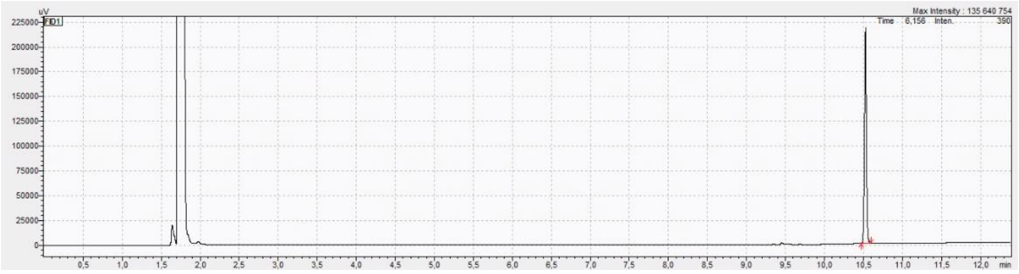

methyl 5-(1-(benzoyloxy)-2-methoxy-2-oxoethyl)-1-propyl-1H-1,2,3-triazole-4-carboxylate (22d)

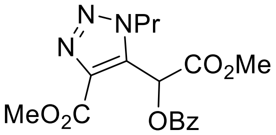

Synthesized from benzoic acid and diazo compound **7d** (0.1 mmol scale) according to the general procedure.

Yield: 17 mg (54%), colorless oil from flash column chromatography (hexanes/AcOEt, 50:50 (v/v)), repurified by HPLC (general condition, hexanes/AcOEt 50:50 (v/v), t_r = 34.90 min).

^{1}H NMR ($CDCl_3$, 400 MHz): δ = 8.06–8.03 (m, 2H), 7.62 (ddt, *J* = 8.2, 7.2, 1.4 Hz, 1H), 7.49–7.45 (m, 2H), 7.35 (s, 1H), 4.50 (td, *J* = 7.1, 1.2 Hz, 2H), 4.00 (s, 3H), 3.80 (s, 3H), 2.06–1.97 (m, 2H), 0.99 (t, *J* = 7.4 Hz, 3H) ppm; **^{13}C NMR** ($CDCl_3$, 126 MHz): δ = 166.5, 164.9, 161.4, 137.7, 134.3, 134.2, 130.1, 128.8, 128.4, 63.2, 53.6, 52.6, 51.4, 23.8, 11.1 ppm; **HRMS** (ESI): *m/z* calcd for $C_{17}H_{19}N_3O_6$+H^+: 362.1352 $[M+H]^+$; found: 362.1354.

HPLC Chromatogram: t_r = 18.72 min (> 95% purity).

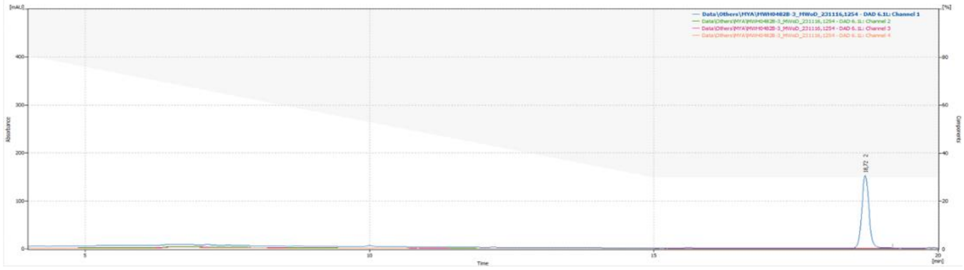

methyl 1-benzyl-5-(2-methoxy-2-oxo-1-(phenylthio)ethyl)-1H-1,2,3-triazole-4-carboxylate (23)

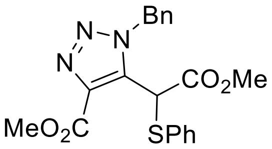

Synthesized from thiophenol and diazo compound **7a** (0.1 mmol scale) according to the general procedure.

Yield: 35 mg (89%), white solid, flash column chromatography (hexanes/AcOEt, 50:50 (v/v)).

m.p. 129-130 ºC; **^{1}H NMR** ($CDCl_3$, 500 MHz): δ = 7.35–7.28 (m, 4H), 7.27–7.22 (m, 4H), 7.18–7.16 (m, 2H), 6.15 (s, 1H), 5.73 (d, *J* = 15.8 Hz, 1H), 5.58 (d, *J* = 15.8 Hz, 1H), 3.84 (s, 3H), 3.32 (s, 3H) ppm; **^{13}C NMR** ($CDCl_3$,126 MHz): δ = 167.1, 161.6, 137.20, 137.18, 134.3, 133.9, 130.8, 129.42, 129.41, 128.9, 128.6, 127.6, 53.3, 52.8, 52.3, 45.2 ppm; **HRMS** (ESI): *m/z* calcd for $C_{20}H_{19}N_3O_4S$+H^+: 398.1175 $[M+H]^+$; found: 398.1180.

HPLC Chromatogram: t_r = 19.67 min (> 99% purity).

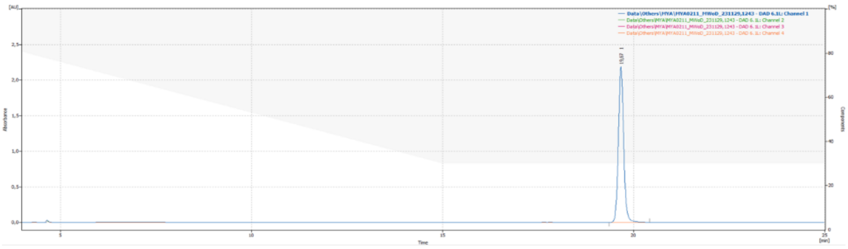

methyl 1-benzyl-5-(1-(benzylamino)-2-methoxy-2-oxoethyl)-1H-1,2,3-triazole-4-carboxylate (24)

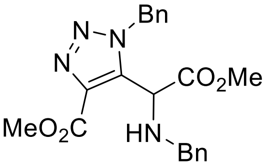

Synthesized from benzyl amine and diazo compound **7a** (0.1 mmol scale) according to the general procedure.

Yield: 23 mg (57%), yellow oil from flash column chromatography (hexanes/AcOEt, 60:40 (v/v)), repurified by HPLC (general condition, hexanes/AcOEt, 50:50 (v/v), t_r = 35.25 min).

^{1}H NMR ($CDCl_3$, 500 MHz): δ = 7.31–7.25 (m, 6H), 7.19–7.17 (m, 2H), 7.10–7.08 (m, 2H), 5.83 (d, *J* = 15.4 Hz, 1H), 5.51 (d, *J* = 15.4 Hz, 1H), 5.32 (s, 1H), 3.93 (s, 3H), 3.71–3.65 (m, 1H), 3.20 (s, 3H), 2.88 (br, 1H) ppm; **^{13}C NMR** ($CDCl_3$,126 MHz): δ = 169.4, 162.0, 138.7, 138.6, 138.1, 134.1, 128.9, 128.7, 128.5, 128.3, 127.8, 127.7, 54.0, 52.74, 52.69, 52.3, 52.1 ppm; **HRMS** (ESI): *m/z* calcd for $C_{21}H_{22}N_4O_4$+H^+: 395.1719 $[M+H]^+$; found: 395.1721.

HPLC Chromatogram: t_r = 19.12 min (> 98% purity).

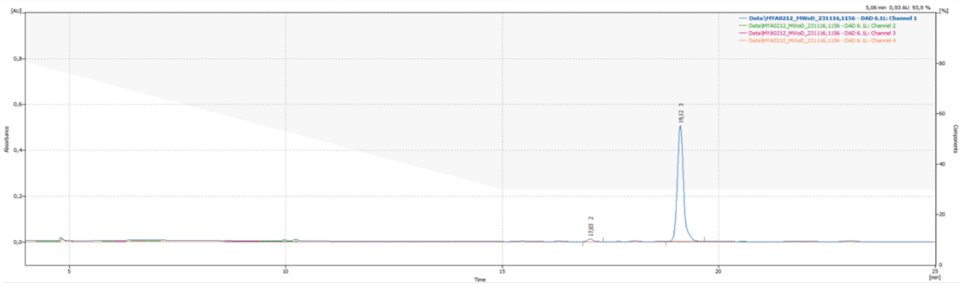

methyl 1-benzyl-5-((2R,3S)-1-(methoxycarbonyl)-2,3-diphenylcyclopropyl)-1H-1,2,3-triazole-4-carboxylate (S3)

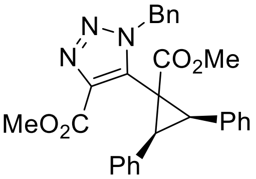

Synthesized from *cis*-stillben and diazo compound **7a** (0.16 mmol scale) according to the general procedure.

Yield: 9 mg (12%), yellow oil, flash column chromatography (hexanes/AcOEt, 60:40 (v/v)).

^{1}H NMR ($CDCl_3$, 500 MHz): δ = 7.32–7.27 (m, 1H), 7.24–7.13 (m, 4H), 7.13–7.06 (m, 5H), 7.02–6.97 (m, 2H), 6.84–6.76 (m, 2H), 5.19 (d, J = 15.1 Hz, 1H), 4.61 (d, J = 15.1 Hz, 1H), 3.68 (s, 2H), 3.61 (s, 3H), 3.07 (s, 3H) ppm; **^{13}C NMR** ($CDCl_3$, 126 MHz): δ = 171.4, 161.6, 141.6, 134.0, 133.8, 133.6, 133.2, 130.3, 129.6, 128.8, 128.63, 128.60, 128.3, 128.0, 127.7, 127.1, 53.4, 53.1, 52.1, 38.8, 38.4, 31.0 ppm; **HRMS** (APCI): *m/z* calcd for $C_{28}H_{25}N_3O_4$+H^+: 468.1923 $[M+H]^+$; found: 468.1927.

GC Chromatogram: t_r = 8.75 min (> 99% purity).

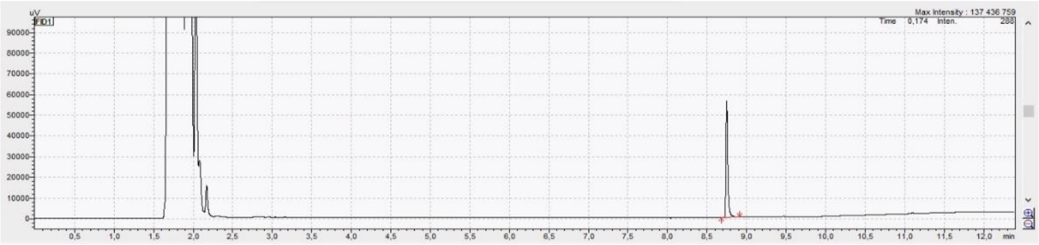
uV
FID1
Max Intensity : 137 436 759
Time 0,174 Inten. 288
90000
80000
70000
60000
50000
40000
30000
20000
10000
0
0,5 1,0 1,5 2,0 2,5 3,0 3,5 4,0 4,5 5,0 5,5 6,0 6,5 7,0 7,5 8,0 8,5 9,0 9,5 10,0 10,5 11,0 11,5 12,0 min

6. Thermal reactions

6.1 General procedure for thermal reactions involving diazo compounds

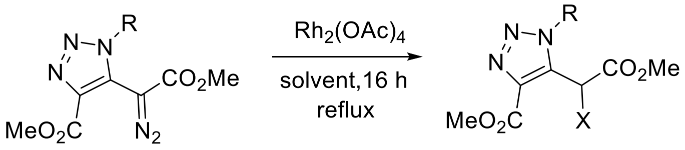

7a-b

R = Bn, Ph
Solvent: DCM, MeOH, MeCN

a) with a metal catalyst: A Schenk flask was charged with a diazo compound (0.1 mmol), $Rh_2(OAc)_4$ (5 mol%, 2.7 mg) and dissolved in a solvent (2.5 mL, c = 0.04 M). The reaction mixture was stirred at reflux for 16 h. After that, the crude reaction mixture was concentrated *in vacuo* and purified by flash column chromatography using hexanes/AcOEt to afford the final product.

b) without a metal catalyst: A Schenk flask was charged with a diazo compound (0.1 mmol), dissolved in a solvent (2.5 mL, c = 0.04 M), then the reaction mixture was stirred at reflux for 16 h. After that, the crude reaction mixture was concentrated *in vacuo* and purified by flash column chromatography using hexanes/AcOEt to afford the final product.

7. Mechanistic considerations

7.1 Experiments with the TEMPO radical trap

Two reactions were set up following the general procedure on 0.1 mmol scale. A) TEMPO (0.2 mmol, 2.0 equiv., 31 mg) was added to the solution of compound **7a** in DCM (c = 0.04 M) and the mixture was irradiated with blue LED (entry 1). B) the solution of compound **7a** in DCM was irradiated for 1 h then TEMPO solution in DCM (c = 0.2 M) was added (same scale). Both reactions were irradiated for 16 h and after that time crude reaction mixtures were analyzed by ESI MS.

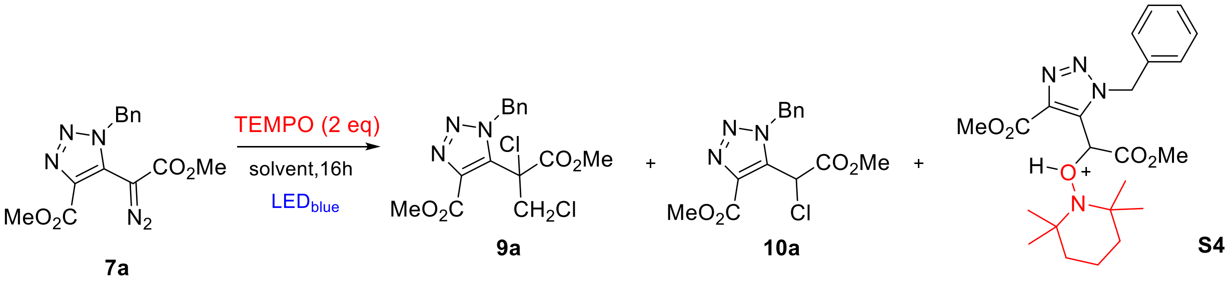

Chemical Formula: $C_{23}H_{33}N_4O_5^+$

m/z: 445.2446

Entry	Solvent	TEMPO added	Yield of 9a [%][a]	Yield of 10a [%][a]
1	DCM	no	45	7
2	DCM	prior irradiation	traces	8
3	DCM	after 1 h	15	11
4	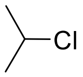	no	-	90
5	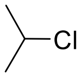	prior irradiation	-	78

MS analysis of the crude reaction mixture when TEMPO was added before irradiation revealed the presence of 445.24 m/z peak, corresponding to protonated TEMPO adduct **S4**:

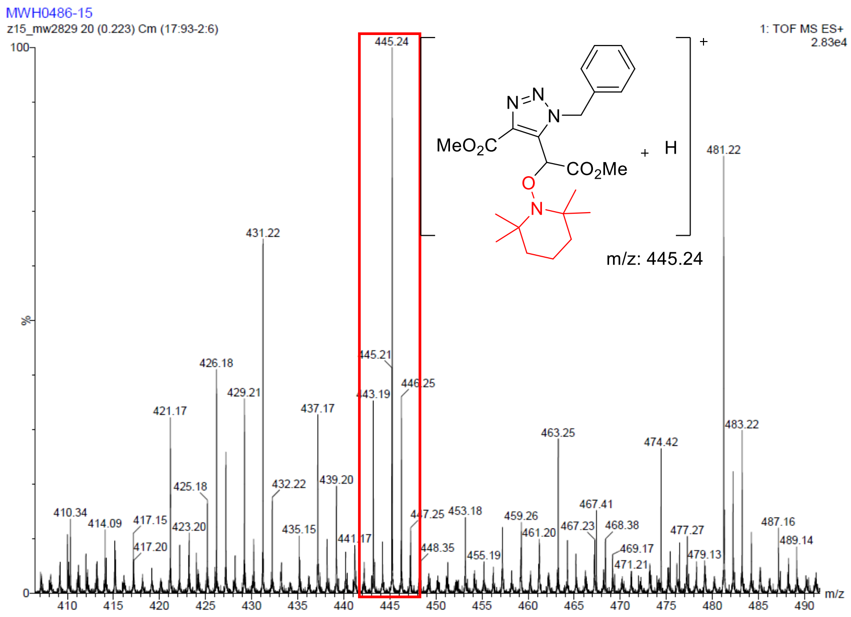

7.2 Experiments in CD_2Cl_2

7.2.1 Characterization of the deuterated products

methyl 1-benzyl-5-(2,3-dichloro-1-methoxy-1-oxopropan-2-yl-3,3-d₂)-1H-1,2,3-triazole-4-carboxylate (25)

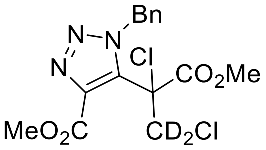

Synthesized from diazo compound **7a** (0.16 mmol scale) according to the general procedure in dry CD_2Cl_2.

Yield: 24 mg (40%), yellow oil from flash column chromatography (hexanes/AcOEt, 60:40 (v/v)), repurified by HPLC using general condition (hexanes/AcOEt 50:50 (v/v), t_r = 28.68 min).

^{1}H NMR ($CDCl_3$, 400 MHz): δ = 7.36–7.28 (m, 3H), 7.15–7.12 (m, 2H), 6.16 (d, *J* = 15.9 Hz, 1H), 5.99 (d, *J* = 15.9 Hz, 1H), 3.95 (s, 3H), 3.74 (s, 3H) ppm; **^{13}C NMR** ($CDCl_3$, 126 MHz): δ = 166.1, 162.3, 136.7, 136.6, 135.3, 128.9, 128.2, 126.8, 65.9, 55.3, 54.2, 52.9 ppm; **HRMS** (ESI): *m/z* calcd for $C_{15}H_{13}D_2N_3O_4Cl_2+H^+$: 374.0643 $[M+H]^+$; found: 374.0642.

GC Chromatogram: t_r = 9.29 min (> 96% purity).

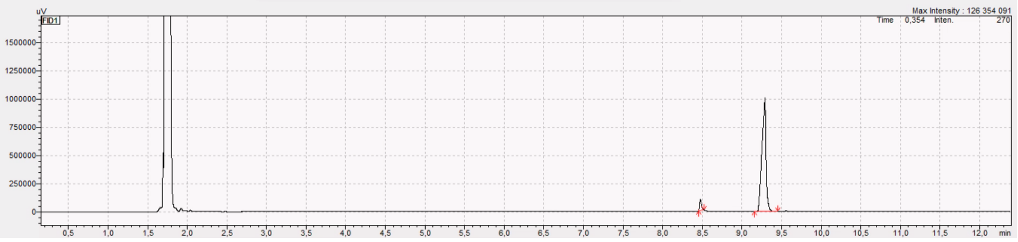

8. Crystallographic Data

Single crystals of **7b**, **15a** and **18** were prepared by slow evaporation of a DCM/ hexane solution. Suitable crystal, of mentioned compounds, were embedded in the inert perfluoropolyalkylether oil (viscosity 1800cSt; ABCR GmbH) and mounted using Hampton Research Cryoloops. All data were collected at 100.0(1) K, SuperNova Agilent diffractometer fitted with MoKα radiation (λ =0.71073 Å) and CuKα radiation (λ = 1.54184 Å). The data were processed with CrysAlisPro.[2] Structures were solved by direct methods and refined using SHELXL[3] under WinGX.[4] All non-hydrogen atoms were refined with anisotropic displacement parameters. All hydrogen atom positions were determined by geometry and refined by a riding model.

Crystallographic Data for Compound 7b

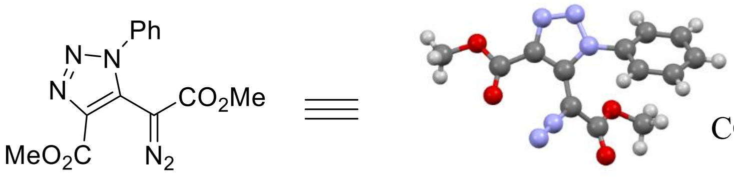

CCC2338526

Figure S. ORTEP drawing of **7b** showing thermal ellipsoids at the 50% probability level.

Crystallographic Data for Compound 15a

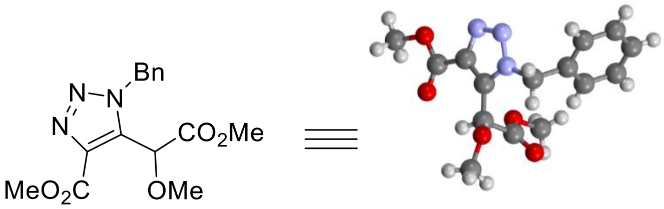

CCC2338527

Figure S. ORTEP drawing of **16a** showing thermal ellipsoids at the 50% probability level.

Crystallographic Data for Compound 18

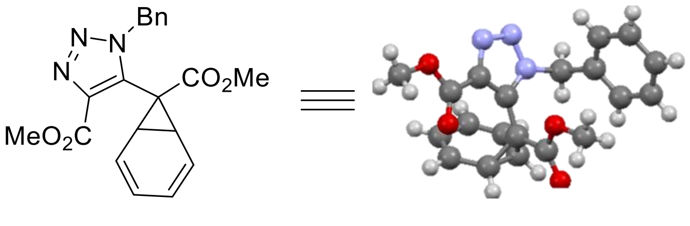

CCC2338528

Figure S. ORTEP drawing of **18** showing thermal ellipsoids at the 50% probability level.

Table S4: Crystallographic data and structure refinement for Compounds **7b**, **16a**, **19**.

Identification code	Compound 7b	Compound 15a	Compound 18
Empirical formula	$C_{13}H_{11}N_5O_4$	$C_{15}H_{17}N_3O_5$	$C_{20}H_{19}N_3O_4$
Formula weight	301.27	319.32	365.38
Crystal system	Monoclinic	Triclinic	Triclinic
Space group	*P2₁/c*	*P -1*	*P -1*
a (Å)	9.2111(3)	9.3850(3)	8.5141(4)
b (Å)	16.7585(5)	11.3895(4)	13.2198(5)
c (Å)	9.1102(2)	15.2021(4)	15.9795(4)
α (°)	90	108.527(3)	84.878(3)
β (°)	98.137(3)	90.246(2)	87.365(3)
γ (°)	90	101.077(2)	80.451(4)
Volume (Å^3)	1392.13(7)	1508.31(9)	1765.68(12)
Z	4	4	4
ρ (calc.)	1.437	1.406	1.374
λ	0.71073	0.71073	1.54184
Temp. (K)	100.0(1)	100.0(1)	100.0(1)
Crystal Size(mm)	0.420x 0.310x 0.250	0.580x 0.410x 0.220	0.230x 0.110x 0.080
Crystal Color	Yellow	colorless	colorless
Crystal Morphology	prism	Prism	prism
F(000)	624	672	768
μ (mm^{-1})	0.110	0.107	0.803
T_{min}, T_{max}	0.955, 0.973	0.940, 0.977	0.837, 0.939
θ_{min}, θ_{max} (°)	2.431 to 30.153	2.217 to 30.119	5.562 to 66.600
Reflections collected	11607	24013	31445
Independent reflections	3659 [R(int) = 0.0399]	7919 [R(int) = 0.0234]	6218 [R(int) = 0.0764]
Completeness	99.9%	99.8%	99.8%
Data / restraints / parameters	3659 / 0 / 201	7919 / 0 / 421	6218/ 0 / 491

Observed data [I > 2σ(I)]	2786	6660	5722
$wR(F^2$ all data)	0.1110	0.1084	0.1723
$R(F$ obsd data)	0.0454	0.0396	0.0643
Goodness-of-fit on F^2	1.031	0.912	0.955
largest diff. peak and hole (e Å^{-3})	0.32 / -0.27	0.38 / -0.24	0.36 / -0.33

$$wR_2 = \{ \Sigma [w(F_o^2 - F_c^2)^2] / \Sigma [w(F_o^2)^2] \}^{1/2}$$

$$R_1 = \Sigma ||F_o| - |F_c|| / \Sigma |F_o|$$

9. Computational studies

General

All the calculations were performed with Gaussian 16 package.[5] Structures of minima and transition states were optimized employing B3LYP/6-31G(d) level of theory with the D3 version of Grimme's empirical dispersion correction.[6] Frequency analysis was performed at the same level to provide correction to thermodynamic functions and confirm the nature of optimized structures (minima and transition states featured zero or one imaginary frequency, respectively). Wavefunctions were tested for stability (if an instability was found the wavefunction was reoptimized with stable=opt keyword). Unrestricted DFT calculation was used to model homolytic cleavage of bond. Single point energies were calculated at M06/6-311+G(d,p) level of theory employing solvation (DCM) with the SMD model.[7] Molecular structures were visualized in CYLview.[8]

Optimized geometries, energies and corrections to thermodynamic functions.

DCM

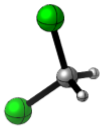

E (B3LYP/6-31G(d)) = -959.698158369
E (M06/6-311++G(d,p)/SMD(DCM)) = -959.669658339

Zero-point correction=	0.029522 (Hartree/Particle)
Thermal correction to Energy=	0.033099
Thermal correction to Enthalpy=	0.034043
Thermal correction to Gibbs Free Energy=	0.003338

Charge = 0 Multiplicity = 1

C	0.00000000	0.00000000	0.76835900
H	-0.89918100	0.00000000	1.38217500
H	0.89918100	0.00000000	1.38217500
Cl	0.00000000	-1.49874400	-0.21689700
Cl	0.00000000	1.49874400	-0.21689700

Carbene (singlet)

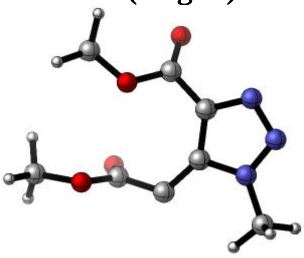

E (B3LYP/6-31G(d)) = -775.301898722
E (M06/6-311++G(d,p)/SMD(DCM)) = -775.051164964

Zero-point correction=	0.176565 (Hartree/Particle)
Thermal correction to Energy=	0.191911
Thermal correction to Enthalpy=	0.192855

Thermal correction to Gibbs Free Energy= 0.132281

Charge = 0 Multiplicity = 1

C	-1.05906600	0.93618100	-0.06267400
C	-1.12898300	-0.48456800	-0.01811000
N	-2.51397700	-0.68175100	-0.10928300
N	-3.16388800	0.44686400	-0.21510200
N	-2.28318300	1.45733800	-0.16307000
C	-3.22025900	-1.95778200	-0.11731800
H	-3.61120900	-2.15052600	-1.11930100
H	-2.49498500	-2.72339500	0.16030500
H	-4.04231300	-1.90947400	0.59831000
C	0.13674200	1.81751400	0.07276500
O	1.19123300	1.27902000	-0.56470300
O	0.14160000	2.86327500	0.67650200
C	2.43784200	1.97986300	-0.39174900
H	2.32187900	3.03661700	-0.64331300
H	2.77047100	1.88389300	0.64565300
H	3.13868800	1.49272100	-1.06919300
C	-0.27031200	-1.55765500	0.15497600
C	1.13483300	-1.37335000	0.37813400
O	1.93623300	-1.69827400	-0.65165000
O	1.52082100	-1.07751500	1.50497600
C	3.34338900	-1.69642100	-0.35111700
H	3.83864000	-1.96509200	-1.28464200
H	3.67016000	-0.70856800	-0.01506900
H	3.57180400	-2.42806600	0.42898900

TS1

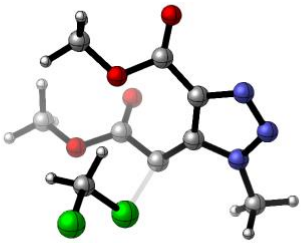

E (B3LYP/6-31G(d)) = -1735.00404040
E (M06/6-311++G(d,p)/SMD(DCM)) = -1734.72288897

Zero-point correction= 0.207359 (Hartree/Particle)
Thermal correction to Energy= 0.227041
Thermal correction to Enthalpy= 0.227985
Thermal correction to Gibbs Free Energy= 0.156006

Charge = 0 Multiplicity = 1

C	-1.85849600	0.08436200	-0.69207000
C	-1.31911100	-0.58239200	0.41930500
N	-2.13710900	-1.68187600	0.51937000
N	-3.07105500	-1.69164200	-0.43749600
N	-2.92211900	-0.60545800	-1.16394800

C	-2.05987900	-2.76216600	1.48914900
H	-1.41197800	-3.56072800	1.11498200
H	-1.64267200	-2.35770600	2.41274400
H	-3.06680500	-3.14930000	1.64724300
C	-1.44628200	1.36889500	-1.30059600
O	-0.09966400	1.54504000	-1.17690600
O	-2.17700400	2.16020500	-1.84376000
C	0.37862000	2.84472600	-1.56519800
H	0.15459700	3.04094600	-2.61666000
H	-0.09091200	3.61271500	-0.94466600
H	1.45598400	2.82017200	-1.39610200
C	-0.28552500	-0.32054800	1.37722700
C	0.17477200	1.03530800	1.57345900
O	1.51881800	1.25170500	1.57129300
O	-0.63990200	1.91007200	1.83677200
C	1.90367700	2.57851400	1.96871400
H	2.99454600	2.58213600	1.95214300
H	1.50642200	3.32223200	1.27188800
H	1.53442400	2.80456100	2.97237000
Cl	1.37571200	-1.49243900	0.74698100
C	2.17443500	-0.71010100	-0.72067800
H	1.35727600	-0.30166100	-1.31205500
H	2.81233800	0.06950600	-0.31246100
Cl	3.12531800	-1.87143200	-1.66521500

I

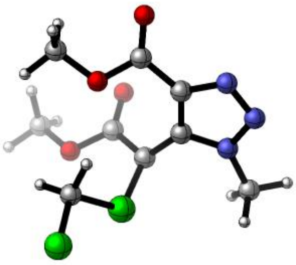

E (B3LYP/6-31G(d)) = -1735.01004232
E (M06/6-311++G(d,p)/SMD(DCM)) = -1734.73052224

Zero-point correction=	0.207955 (Hartree/Particle)
Thermal correction to Energy=	0.228101
Thermal correction to Enthalpy=	0.229045
Thermal correction to Gibbs Free Energy=	0.155729

Charge = 0 Multiplicity = 1

C	-1.92189900	-0.01988800	-0.67540800
C	-1.24705300	-0.67359400	0.35563000
N	-2.01741100	-1.77658300	0.57162300
N	-3.08052200	-1.80513700	-0.26663400
N	-3.03173600	-0.73452700	-1.00823200
C	-1.81584000	-2.84221300	1.53733900
H	-1.14804500	-3.60968100	1.13307700

H	-1.37536700	-2.41692100	2.44146800
H	-2.78850200	-3.28399300	1.75561800
C	-1.58369000	1.24955100	-1.34520400
O	-0.24230200	1.50095900	-1.24842700
O	-2.35475200	1.98075800	-1.91843000
C	0.15931000	2.80186700	-1.70747200
H	-0.07370800	2.92887900	-2.76793300
H	-0.35256900	3.57771200	-1.13164000
H	1.23681700	2.85023700	-1.54058100
C	-0.04950100	-0.35943100	1.11794700
C	0.17379700	1.00112000	1.57447500
O	1.49530500	1.32304500	1.71580800
O	-0.72788200	1.78372600	1.81878500
C	1.73127600	2.64218300	2.22972000
H	2.81520900	2.73476200	2.31311500
H	1.33127800	3.39881800	1.54795900
H	1.25859300	2.76857900	3.20756400
Cl	1.35364800	-1.27752700	0.69840600
C	2.24899700	-0.55410600	-0.94552900
H	1.40275600	-0.34029900	-1.59465200
H	2.74626900	0.33042000	-0.55513200
Cl	3.37103600	-1.71692100	-1.62058400

TS2

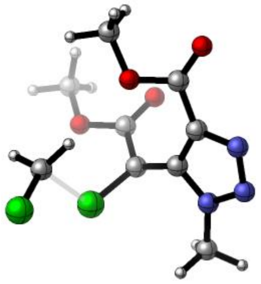

E (B3LYP/6-31G(d)) = -1735.00744462
E (M06/6-311++G(d,p)/SMD(DCM)) = -1734.72165802

Zero-point correction=	0.206413 (Hartree/Particle)
Thermal correction to Energy=	0.226299
Thermal correction to Enthalpy=	0.227243
Thermal correction to Gibbs Free Energy=	0.154144

Charge = 0 Multiplicity = 1

C	1.84133600	-0.46315400	0.65614400
C	1.05661200	-0.83083500	-0.43716900
N	1.53164400	-2.06674500	-0.75639200
N	2.53535500	-2.43323300	0.07755900
N	2.72808500	-1.46055900	0.92159300
C	1.10481600	-2.95318400	-1.82485400
H	0.18222300	-3.47299200	-1.54958800
H	0.93325300	-2.36680000	-2.73058400
H	1.90214500	-3.67866800	-1.98728600

C	1.78132600	0.76933700	1.46551500
O	0.52465700	1.30422100	1.42921300
O	2.69245000	1.25106500	2.09430400
C	0.40714000	2.60257800	2.03386000
H	0.67399900	2.56256500	3.09312400
H	1.06283300	3.31361500	1.52369700
H	-0.63882300	2.88783100	1.90989300
C	-0.00924600	-0.15402400	-1.14713200
C	0.10690600	1.26826500	-1.44408400
O	-1.08832600	1.87341500	-1.67089600
O	1.17545900	1.85451700	-1.49113000
C	-0.99516000	3.26654900	-2.00492800
H	-2.02083100	3.59464900	-2.17850600
H	-0.54384600	3.82992700	-1.18262000
H	-0.38728500	3.41033600	-2.90249900
Cl	-1.56840200	-0.84585800	-1.03867100
C	-2.58408200	-0.19725800	0.91826500
H	-1.65290800	0.03461300	1.42905300
H	-3.16609700	0.63135100	0.52735500
Cl	-3.52583800	-1.45638100	1.63649500

Radical, product of chlorination of carbene

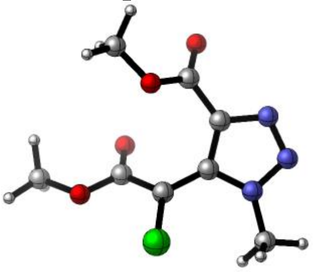

E (B3LYP/6-31G(d)) = -1235.57130134
E (M06/6-311++G(d,p)/SMD(DCM)) = -1235.31231375

Zero-point correction=	0.179650 (Hartree/Particle)
Thermal correction to Energy=	0.195999
Thermal correction to Enthalpy=	0.196943
Thermal correction to Gibbs Free Energy=	0.133871

Charge = 0 Multiplicity = 2

C	0.94525200	1.35597400	-0.07365600
C	1.08859300	-0.04369100	-0.05025900
N	2.44334700	-0.21403300	-0.17866400
N	3.06694600	0.97979900	-0.28861000
N	2.16686500	1.92085700	-0.21809500
C	3.23498800	-1.42945400	-0.31158300
H	3.31862900	-1.94295600	0.64837700
H	2.76984800	-2.09724500	-1.04001000
H	4.22114000	-1.12251700	-0.65999100
C	-0.26781700	2.19465500	0.09147200
O	-1.13584700	1.61443100	0.95144900

O	-0.44123400	3.26309200	-0.44452300
C	-2.41125700	2.26982500	1.05106800
H	-2.28809400	3.30890100	1.36688200
H	-2.91746100	2.24386300	0.08166600
H	-2.97231900	1.70380500	1.79561700
C	0.11181900	-1.07972000	0.05913900
C	-1.22344700	-0.92231000	-0.54110000
O	-2.09561800	-1.86506800	-0.14260800
O	-1.49121500	-0.03444600	-1.33344500
C	-3.40459800	-1.76114000	-0.72858200
H	-3.97146700	-2.59772600	-0.32020400
H	-3.86883300	-0.80875800	-0.45680900
H	-3.34377900	-1.82709200	-1.81840200
Cl	0.48131400	-2.52914900	0.93967600

CH_2Cl - radical

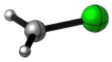

E (B3LYP/6-31G(d)) = -499.439080569
E (M06/6-311++G(d,p)/SMD(DCM)) = -499.408382843

Zero-point correction=	0.022632 (Hartree/Particle)
Thermal correction to Energy=	0.026280
Thermal correction to Enthalpy=	0.027224
Thermal correction to Gibbs Free Energy=	-0.002042

Charge = 0 Multiplicity = 2

C	-0.00419800	1.12655200	0.00000000
H	0.04827300	1.63067600	0.95510000
H	0.04827300	1.63067600	-0.95510000
Cl	-0.00419800	-0.58945100	0.00000000

II

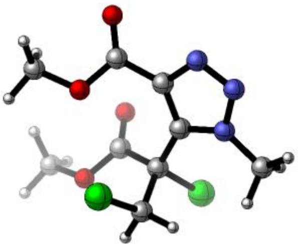

E (B3LYP/6-31G(d)) = -1735.11924886
E (M06/6-311++G(d,p)/SMD(DCM)) = -1734.83825280

Zero-point correction=	0.211977 (Hartree/Particle)
Thermal correction to Energy=	0.230644
Thermal correction to Enthalpy=	0.231588
Thermal correction to Gibbs Free Energy=	0.164585

Charge = 0 Multiplicity = 1

C	0.47828600	1.67433100	-0.23413400

C	1.07461200	0.41795000	-0.15253000
N	2.40553900	0.70627600	-0.05498300
N	2.60681800	2.04706000	-0.09363400
N	1.45272900	2.62571900	-0.20326900
C	3.59272000	-0.13752800	0.01932700
H	3.46552400	-0.92028000	0.76830200
H	3.80083600	-0.59525400	-0.94909500
H	4.41317500	0.51954200	0.30786800
C	-0.93989900	2.10832900	-0.29626600
O	-1.73620300	1.28020000	0.42160000
O	-1.33167900	3.09532000	-0.87100200
C	-3.13868100	1.58303600	0.36026500
H	-3.32791900	2.59706600	0.72134200
H	-3.49802000	1.49787500	-0.66971900
H	-3.62111300	0.84770800	1.00516400
C	0.48597600	-0.96151000	-0.09702300
C	-0.90985900	-1.01584500	-0.76774500
O	-1.71755200	-1.86558000	-0.12541100
O	-1.18052800	-0.43074300	-1.78710600
C	-3.00962400	-2.06000200	-0.73540900
H	-3.52420300	-2.77851500	-0.09820900
H	-3.55264100	-1.11293100	-0.77215200
H	-2.89215300	-2.44823500	-1.75022600
Cl	1.44304300	-2.14355100	-1.18847800
C	0.57138000	-1.61662900	1.29418300
H	0.10560400	-2.59948000	1.26536800
H	1.61511700	-1.71468900	1.59060700
Cl	-0.25137000	-0.65248100	2.57643800

TS3

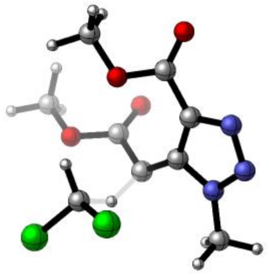

E (B3LYP/6-31G(d)) = -1735.00088986
E (M06/6-311++G(d,p)/SMD(DCM)) = -1734.71412827

Zero-point correction=	0.203796 (Hartree/Particle)
Thermal correction to Energy=	0.223411
Thermal correction to Enthalpy=	0.224355
Thermal correction to Gibbs Free Energy=	0.152799

Charge = 0 Multiplicity = 1

C	-1.89046500	-0.68404100	-0.11443200
C	-0.72429300	-0.92778400	0.63564500
N	-0.82245700	-2.28011200	0.88606200

N	-1.92641300	-2.79539400	0.35872000
N	-2.59076900	-1.82492800	-0.24946600
C	0.11290900	-3.12381100	1.61774000
H	0.89761500	-3.47623200	0.94337000
H	0.55899800	-2.53048900	2.41693800
H	-0.44362800	-3.96998900	2.02057200
C	-2.35275400	0.58878800	-0.72255500
O	-1.28756000	1.34674100	-1.08627400
O	-3.50481400	0.90734300	-0.88698500
C	-1.61635000	2.66344200	-1.56529700
H	-2.25427800	2.60116600	-2.45044600
H	-2.13626200	3.22522800	-0.78427700
H	-0.66113200	3.13001200	-1.80711100
C	0.34439900	-0.14003900	1.13640000
C	0.14510600	1.29536000	1.28818600
O	1.12653800	2.06732800	0.76741000
O	-0.78338100	1.72976400	1.95168400
C	1.02727200	3.46812600	1.07717800
H	1.88633700	3.93301300	0.59268000
H	0.09317600	3.88320400	0.68834500
H	1.06195400	3.62562200	2.15833000
Cl	1.62819000	-1.77280500	-1.51700300
C	1.92426800	-0.24517500	-0.67741500
H	1.42943800	0.57640500	-1.18538700
H	1.46537000	-0.40372800	0.52941500
Cl	3.62199000	0.12164200	-0.37176300

TS4

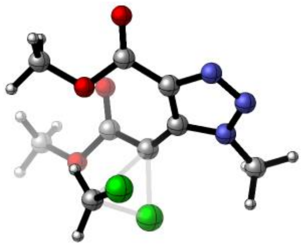

E (B3LYP/6-31G(d)) = -1734.96599795
E (M06/6-311++G(d,p)/SMD(DCM)) = -1734.69186961

Zero-point correction=	0.206223 (Hartree/Particle)
Thermal correction to Energy=	0.225819
Thermal correction to Enthalpy=	0.226763
Thermal correction to Gibbs Free Energy=	0.156946

Charge = 0 Multiplicity = 1

C	-1.19789500	-1.27783800	-0.39444300
C	-1.03462600	0.09948700	-0.63487100
N	-2.31774800	0.50999700	-0.88472800
N	-3.19281600	-0.52569500	-0.79601400
N	-2.51068500	-1.60207600	-0.52963200
C	-2.77974300	1.82199700	-1.29516900

H	-2.81936100	2.51045900	-0.44594300
H	-2.09398200	2.22056400	-2.04799700
H	-3.77704500	1.69925900	-1.71840800
C	-0.18147400	-2.30132000	-0.08888100
O	0.86546200	-1.77241600	0.61256100
O	-0.24986900	-3.46920500	-0.39192900
C	2.01292000	-2.63265900	0.70844100
H	1.74716300	-3.58732000	1.16852700
H	2.42509800	-2.80410000	-0.28923200
H	2.73337500	-2.09537900	1.32843300
C	0.12027300	0.91707000	-0.69452500
C	1.42378000	0.52616200	-1.09677000
O	2.43485500	1.32240000	-0.60980700
O	1.64512100	-0.45346700	-1.80411000
C	3.73838900	0.97990300	-1.09810700
H	4.41876300	1.70690600	-0.65098300
H	4.01431800	-0.03685100	-0.80184900
H	3.77482800	1.04426200	-2.18940100
Cl	-0.00976800	2.55042600	0.34316000
C	0.69253000	1.05654600	1.94894300
H	1.35669100	0.42447900	1.37603200
H	1.08950600	1.88574500	2.52134800
Cl	-0.64023700	0.28497500	2.63570300

Carbene (triplet)

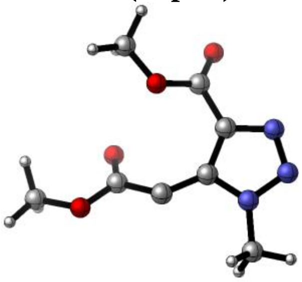

E (B3LYP/6-31G(d)) = -775.299508471
E (M06/6-311++G(d,p)/SMD(DCM)) = -775.046590491

Zero-point correction=	0.176096 (Hartree/Particle)
Thermal correction to Energy=	0.191533
Thermal correction to Enthalpy=	0.192477
Thermal correction to Gibbs Free Energy=	0.130435

Charge = 0 Multiplicity = 3

C	-1.39403400	0.66076400	0.02779100
C	-0.93574600	-0.67620400	-0.09437200
N	-2.11108400	-1.39283600	-0.09073800
N	-3.17651000	-0.57806100	0.04095100
N	-2.74267700	0.65277700	0.11616000
C	-2.27976800	-2.83404400	-0.16067800
H	-1.81724900	-3.22013200	-1.07312500
H	-1.81868600	-3.31089400	0.70918500

H	-3.35146800	-3.03353100	-0.17016800
C	-0.60325300	1.91269300	0.05932900
O	0.50280100	1.78366200	-0.70311400
O	-0.90895700	2.90791200	0.67503800
C	1.44671800	2.86281000	-0.59702000
H	0.97405800	3.81425800	-0.85366500
H	1.83992300	2.91159900	0.42187400
H	2.24288900	2.62289700	-1.30228900
C	0.31585400	-1.25819000	-0.21080100
C	1.64716400	-0.91677600	0.22488400
O	2.58452000	-1.71162000	-0.33019000
O	1.88886700	-0.02057200	1.02054800
C	3.93517900	-1.43515500	0.08116300
H	4.55297000	-2.16707800	-0.43932300
H	4.22141600	-0.41800300	-0.20208200
H	4.03752000	-1.54271400	1.16496100

TS5

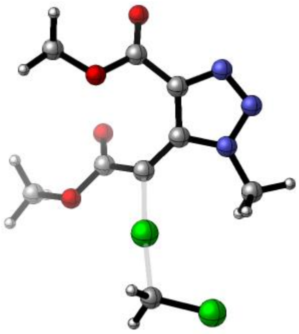

E (B3LYP/6-31G(d)) = -1734.98424324
E (M06/6-311++G(d,p)/SMD(DCM)) = -1734.69455784

Zero-point correction=	0.205214 (Hartree/Particle)
Thermal correction to Energy=	0.225472
Thermal correction to Enthalpy=	0.226416
Thermal correction to Gibbs Free Energy=	0.150894

Charge = 0 Multiplicity = 3

C	1.68051100	-1.55871500	0.05142600
C	0.44467800	-0.98529400	-0.32176700
N	-0.32052600	-2.08617700	-0.62079300
N	0.38957400	-3.22175200	-0.46024400
N	1.58967300	-2.90350000	-0.05567600
C	-1.68444100	-2.16553800	-1.12649700
H	-2.40451800	-2.01407900	-0.31989100
H	-1.83618600	-1.39761200	-1.88731400
H	-1.80521900	-3.15871000	-1.55951700
C	2.92384900	-0.89645100	0.51110700
O	2.63542100	0.22951700	1.20216900
O	4.04065300	-1.31346200	0.31096500
C	3.77295800	1.04217200	1.53463200

H	4.49845400	0.47094600	2.11939300
H	4.24907200	1.40504600	0.61921300
H	3.37590300	1.87394100	2.11783500
C	0.02656700	0.35557000	-0.39335500
C	0.84428400	1.49162500	-0.79386700
O	0.28177400	2.68389300	-0.52767400
O	1.93184000	1.35536800	-1.33570000
C	1.06475900	3.82068500	-0.92863800
H	0.46837200	4.69272800	-0.65979800
H	2.02318500	3.83078200	-0.40123100
H	1.25468700	3.79770900	-2.00557600
Cl	-1.91519100	0.76401700	0.33561900
C	-3.87786600	1.07725100	0.89506200
H	-4.15060600	1.96854100	0.33663300
H	-3.79507300	1.20673700	1.97105800
Cl	-4.89530400	-0.28909700	0.47964400

C_2H_5Cl

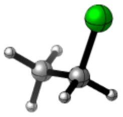

E (B3LYP/6-31G(d)) = -539.429753770
E (M06/6-311++G(d,p)/SMD(DCM)) = -539.377151518

Zero-point correction=	0.067123 (Hartree/Particle)
Thermal correction to Energy=	0.071129
Thermal correction to Enthalpy=	0.072073
Thermal correction to Gibbs Free Energy=	0.040910

Charge = 0 Multiplicity = 1

C	1.61623400	-0.36042200	-0.00000100
H	1.56744100	-0.99819500	-0.88755800
H	1.56719400	-0.99850200	0.88732300
H	2.58128800	0.16176800	0.00022200
C	0.49169000	0.66080200	0.00000000
H	0.51604400	1.29596500	0.88837600
H	0.51605600	1.29601900	-0.88834000
Cl	-1.14091600	-0.15054900	-0.00000100

TS6

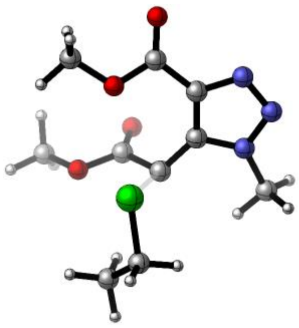

E (B3LYP/6-31G(d)) = -1314.73462188
E (M06/6-311++G(d,p)/SMD(DCM)) = -1314.43469224

Zero-point correction=	0.245189 (Hartree/Particle)
Thermal correction to Energy=	0.265196
Thermal correction to Enthalpy=	0.266140
Thermal correction to Gibbs Free Energy=	0.193689

Charge = 0 Multiplicity = 1

C	-1.99790800	-0.18499300	-0.09779900
C	-0.85639400	-0.90382600	0.27681100
N	-1.36067100	-2.15945500	0.49293500
N	-2.68652100	-2.20259500	0.28387900
N	-3.07735900	-1.00581300	-0.08149500
C	-0.65795600	-3.34347000	0.95810700
H	-0.73280000	-4.13436800	0.20728900
H	0.38334300	-3.06084600	1.11903800
H	-1.10511400	-3.68746700	1.89334800
C	-2.11771400	1.23912500	-0.47259000
O	-0.89259300	1.75822700	-0.75734300
O	-3.14486000	1.87401200	-0.52969700
C	-0.87076100	3.16537500	-1.03896300
H	-1.45091500	3.38714600	-1.93924300
H	-1.28869700	3.72851800	-0.19978500
H	0.18143800	3.41215500	-1.18466500
C	0.54719000	-0.59656300	0.45269400
C	0.88152700	0.61228100	1.17401300
O	1.93024200	1.34270100	0.70599700
O	0.31872700	0.86132300	2.23270800
C	2.29723300	2.44863500	1.54254700
H	3.12971300	2.93625600	1.03244200
H	1.45941700	3.14287800	1.65726200
H	2.60342400	2.10450300	2.53475800
Cl	1.29178200	-0.42131100	-1.47621300
C	2.66181200	-1.65141400	-1.37010700
H	2.17318800	-2.56072900	-1.01780100
H	2.99135700	-1.77220300	-2.40467900
C	3.74842300	-1.16507100	-0.43631600
H	4.19552600	-0.23417200	-0.79432900
H	3.33939500	-0.99968300	0.56246900
H	4.52745600	-1.93523500	-0.38144800

III

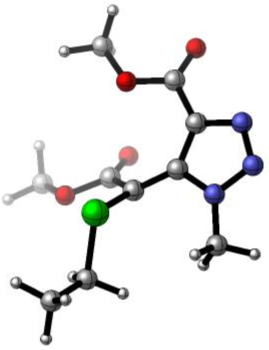

E (B3LYP/6-31G(d)) = -1314.73677052
E (M06/6-311++G(d,p)/SMD(DCM)) = -1314.44032290

Zero-point correction=	0.245733 (Hartree/Particle)
Thermal correction to Energy=	0.265845
Thermal correction to Enthalpy=	0.266789
Thermal correction to Gibbs Free Energy=	0.196111

Charge = 0 Multiplicity = 1

C	1.84135800	-1.05379100	0.07129000
C	0.46779100	-1.06747100	-0.17932200
N	0.22354500	-2.38649400	-0.44130600
N	1.35730000	-3.13007500	-0.33832500
N	2.33346600	-2.31786300	-0.05935800
C	-1.02372100	-3.01209700	-0.82061600
H	-1.69948600	-3.10457000	0.03750500
H	-1.50336300	-2.41202800	-1.60033400
H	-0.79039200	-4.00632100	-1.20305400
C	2.72307300	0.09077000	0.38134100
O	2.03578700	1.06594800	1.02680900
O	3.90149800	0.15831200	0.11441500
C	2.74489000	2.30383600	1.17241400
H	3.67538700	2.15852500	1.72805100
H	2.97237500	2.72060200	0.18704400
H	2.07011700	2.96487600	1.71905900
C	-0.52816900	-0.02591000	-0.26473400
C	-0.28738700	1.28002800	-0.80467900
O	-1.30311500	2.17374900	-0.53624000
O	0.67323600	1.57575600	-1.49910900
C	-1.08827700	3.48342900	-1.07526500
H	-1.96168100	4.06949200	-0.78281300
H	-0.17682900	3.92845000	-0.66466600
H	-0.99664700	3.44808500	-2.16482900
Cl	-1.81274500	-0.12345000	0.96232200
C	-3.44973700	-0.26442700	0.01229700
H	-3.39795000	0.61841300	-0.62505100
H	-3.33282800	-1.17974500	-0.56772600
C	-4.59821100	-0.27308600	0.99372200

H	-4.55855700	-1.14077300	1.65935700
H	-4.61610600	0.63944500	1.59705000
H	-5.53646900	-0.32257800	0.42817300

TS7

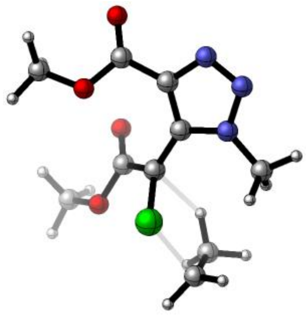

E (B3LYP/6-31G(d)) = -1314.73642883
E (M06/6-311++G(d,p)/SMD(DCM)) = -1314.43756887

Zero-point correction=	0.244776 (Hartree/Particle)
Thermal correction to Energy=	0.264160
Thermal correction to Enthalpy=	0.265105
Thermal correction to Gibbs Free Energy=	0.196123

Charge = 0 Multiplicity = 1

C	1.65727600	-1.17937600	-0.08360900
C	0.26679600	-1.07574900	-0.02619600
N	-0.14379300	-2.36547300	-0.20188600
N	0.92201700	-3.20298800	-0.34622000
N	1.99853800	-2.48186500	-0.29495900
C	-1.48240600	-2.91412900	-0.27801000
H	-2.01496300	-2.77917000	0.66813300
H	-2.04304000	-2.43558000	-1.08503300
H	-1.37601600	-3.97899700	-0.48696600
C	2.68810600	-0.12719500	0.04486500
O	2.26300600	0.86394400	0.86693200
O	3.77456000	-0.14728000	-0.48662200
C	3.10648200	2.02392200	0.89301900
H	4.11895800	1.76273800	1.21252100
H	3.14661400	2.47712500	-0.10180200
H	2.64246900	2.70702100	1.60638400
C	-0.60402500	0.07586200	0.12886000
C	-0.31382900	1.35084500	-0.50734800
O	-1.16591700	2.34442600	-0.12060700
O	0.56264800	1.51762200	-1.33891500
C	-0.91470200	3.61275300	-0.74088700
H	-1.66786100	4.29059400	-0.33612000
H	0.09082300	3.96935700	-0.49904400
H	-1.00633600	3.53932900	-1.82856400
Cl	-1.74875200	0.05308100	1.40463700
C	-3.73886100	-0.17288500	0.47624200
H	-3.99188600	-1.17014900	0.82801100

H	-4.21584100	0.62911700	1.03218800
C	-3.46762500	0.01363800	-0.96097200
H	-3.73915200	1.01038000	-1.31491600
H	-2.36301700	-0.09266400	-1.13847800
H	-3.94696400	-0.75501000	-1.57578700

IV

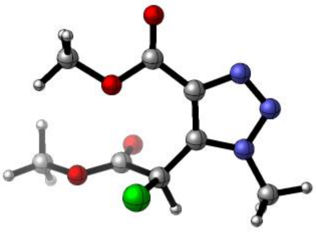

E (B3LYP/6-31G(d)) = -1236.21345102
E (M06/6-311++G(d,p)/SMD(DCM)) = -1235.96062462

Zero-point correction=	0.192531 (Hartree/Particle)
Thermal correction to Energy=	0.209064
Thermal correction to Enthalpy=	0.210008
Thermal correction to Gibbs Free Energy=	0.146857

Charge = 0 Multiplicity = 1

C	1.10909400	1.15333800	-0.09059200
C	1.15004800	-0.23657900	-0.09498200
N	2.46159800	-0.51687600	-0.29033100
N	3.18857900	0.62595000	-0.40752800
N	2.37135800	1.62785300	-0.28923800
C	3.12776900	-1.80596200	-0.37763700
H	2.93595700	-2.39240000	0.52535900
H	2.78612700	-2.35629800	-1.25953300
H	4.19542700	-1.60488400	-0.46605800
C	-0.03422900	2.06498300	0.10722200
O	-1.14091200	1.35259700	0.44414800
O	-0.01049500	3.26868400	0.00457200
C	-2.33197300	2.12131400	0.66792700
H	-2.17056700	2.85175200	1.46504200
H	-2.62362900	2.64931600	-0.24475100
H	-3.09146400	1.39503200	0.95837100
C	0.12054000	-1.29972700	0.09174300
C	-1.12277900	-1.04475800	-0.77425600
O	-2.27622100	-1.32413300	-0.17013600
O	-0.99727300	-0.70952900	-1.92995300
C	-3.44651700	-1.13947100	-0.99174500
H	-4.28988800	-1.40999200	-0.35694000
H	-3.51788300	-0.09695000	-1.31285700
H	-3.39936400	-1.78317600	-1.87387800
Cl	-0.20738800	-1.57272500	1.86614800
H	0.51763600	-2.25433700	-0.26471900

C2H4

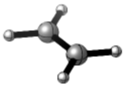

E (B3LYP/6-31G(d)) = -78.5883044152
E (M06/6-311++G(d,p)/SMD(DCM)) = -78.5407542353

Zero-point correction=	0.051217 (Hartree/Particle)
Thermal correction to Energy=	0.054257
Thermal correction to Enthalpy=	0.055201
Thermal correction to Gibbs Free Energy=	0.029688

Charge = 0 Multiplicity = 1

C	0.00000000	-0.66559000	0.00000000
H	0.92384300	-1.23977600	-0.00000100
H	-0.92384300	-1.23977600	-0.00000100
C	0.00000000	0.66559000	0.00000000
H	-0.92384300	1.23977600	-0.00000100
H	0.92384300	1.23977600	-0.00000100

10. NMR and IR spectra

dimethyl 3-(benzylamino)pent-2-enedioate (6a)

^{1}*H NMR (CDCl$_3$, 500 MHz)*, ratio of diasteroisomers: 80:20.

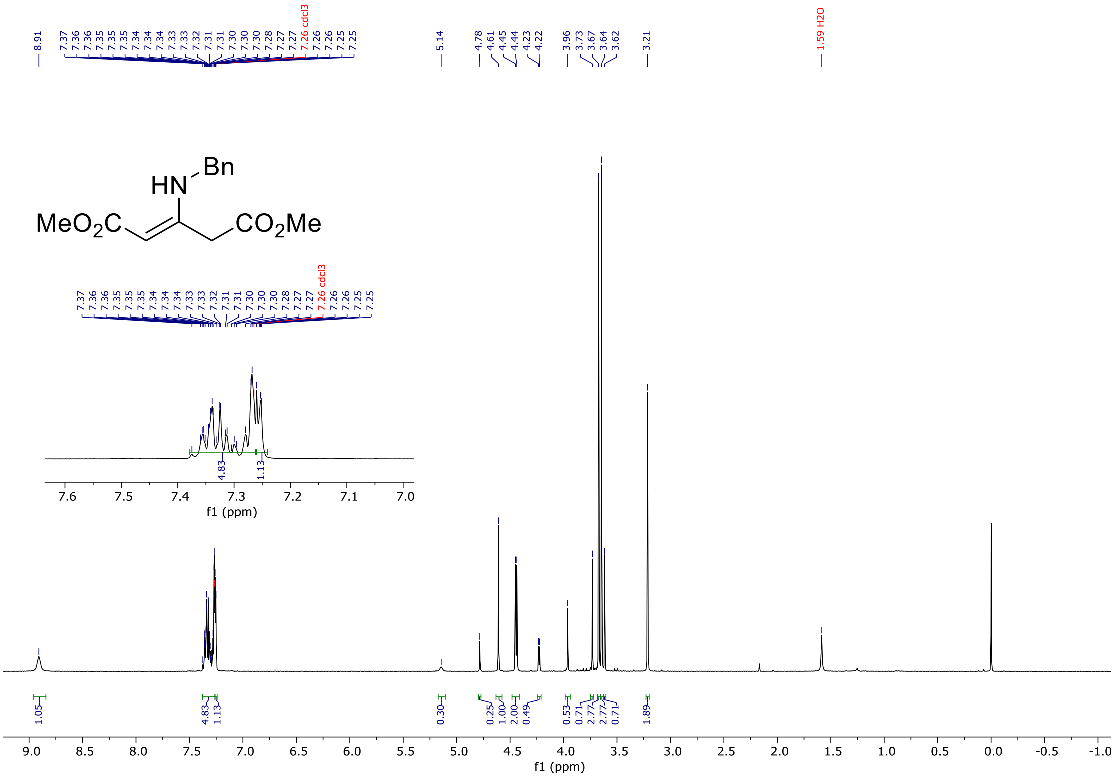

^{13}*C NMR (CDCl$_3$, 126 MHz)*

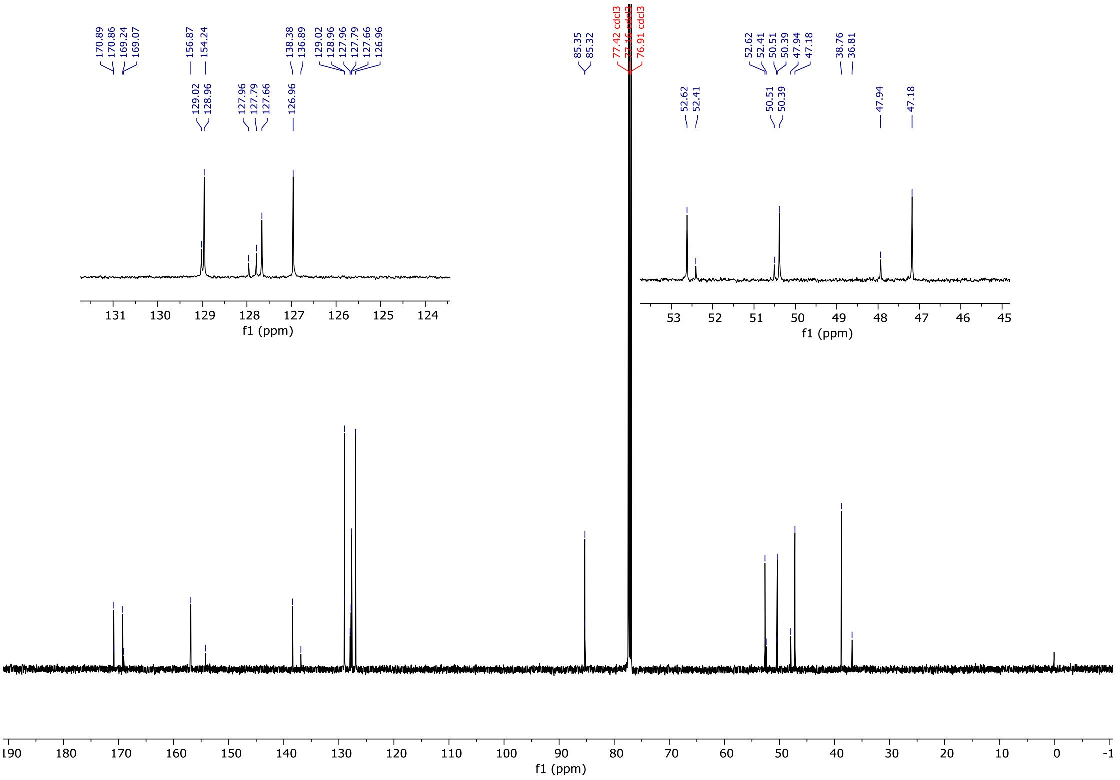

dimethyl 3-(phenylamino)pent-2-enedioate (6b)

[1]*H NMR (CDCl₃, 600 MHz)*, ratio of diasteroisomers: 88:12.

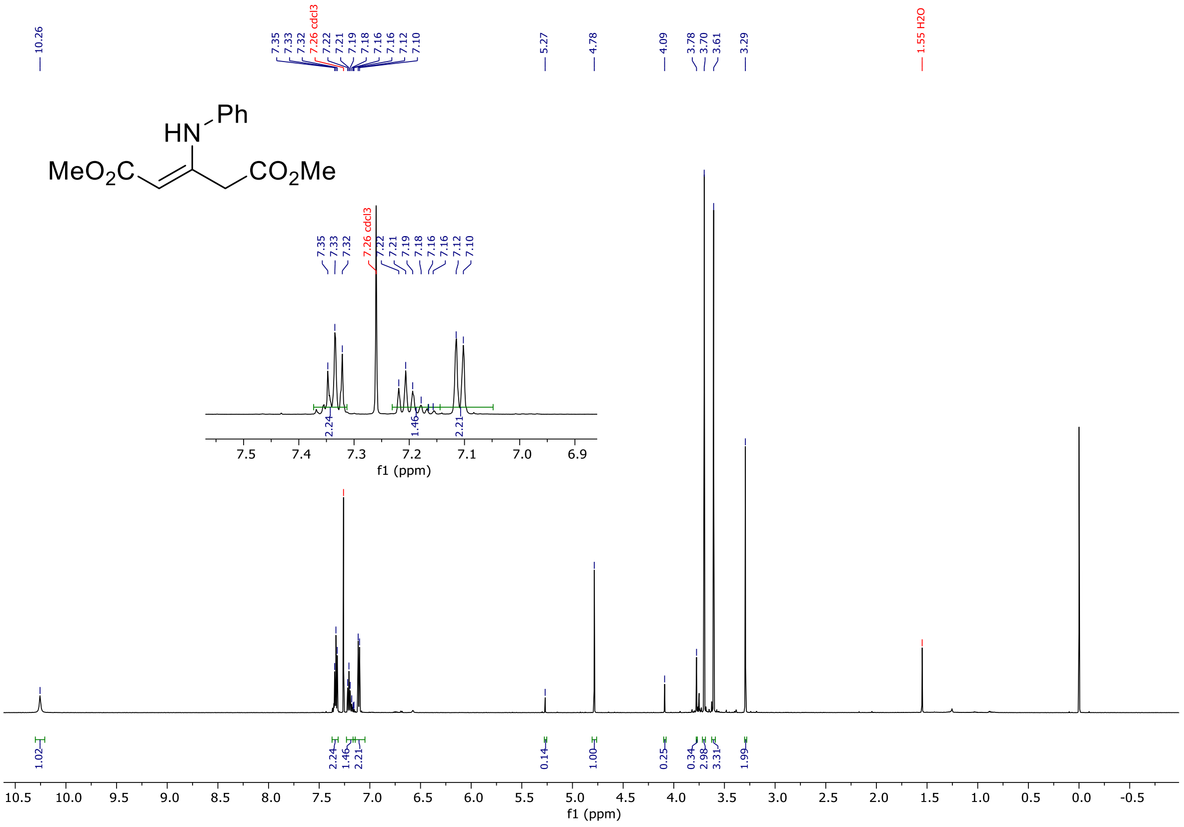

[13]*C NMR (CDCl₃, 151 MHz)*

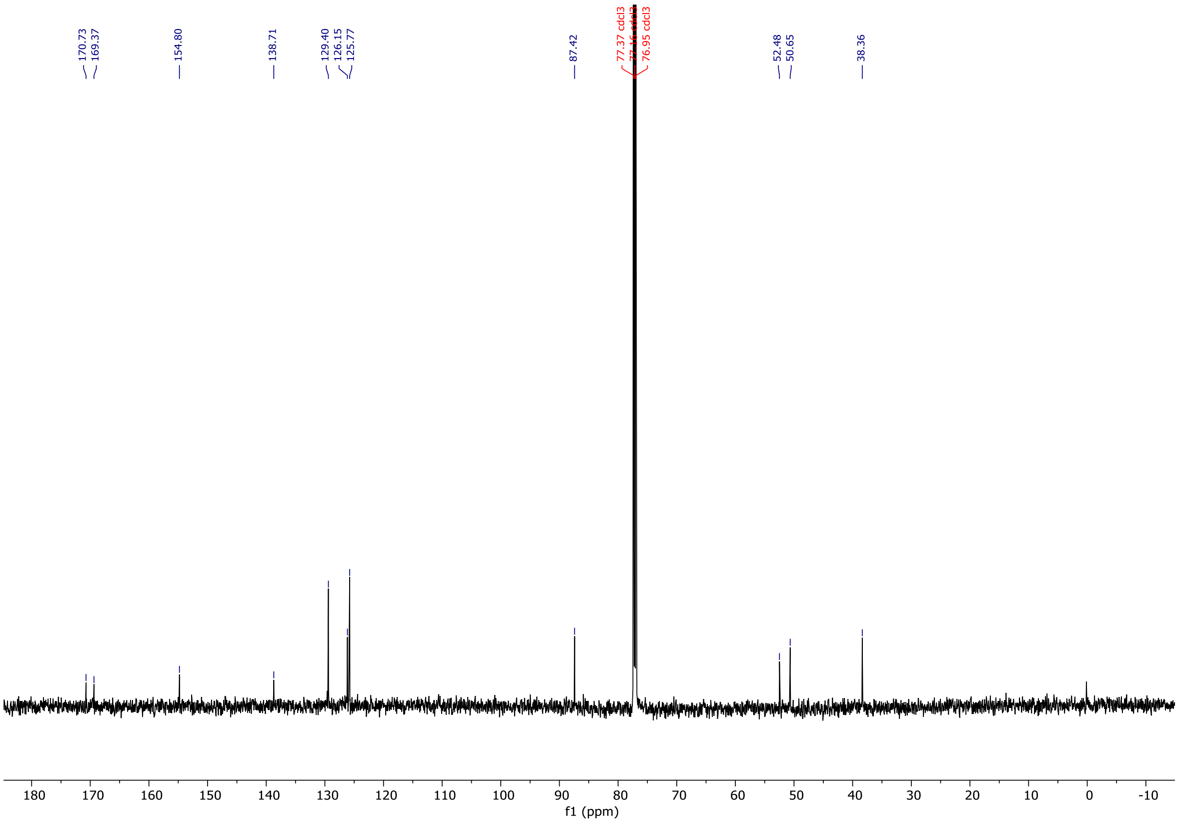

dimethyl 3-(propylamino)pent-2-enedioate (6c)

[1]*H NMR (CDCl$_3$, 500 MHz)*, ratio of diasteroisomers: 82:18.

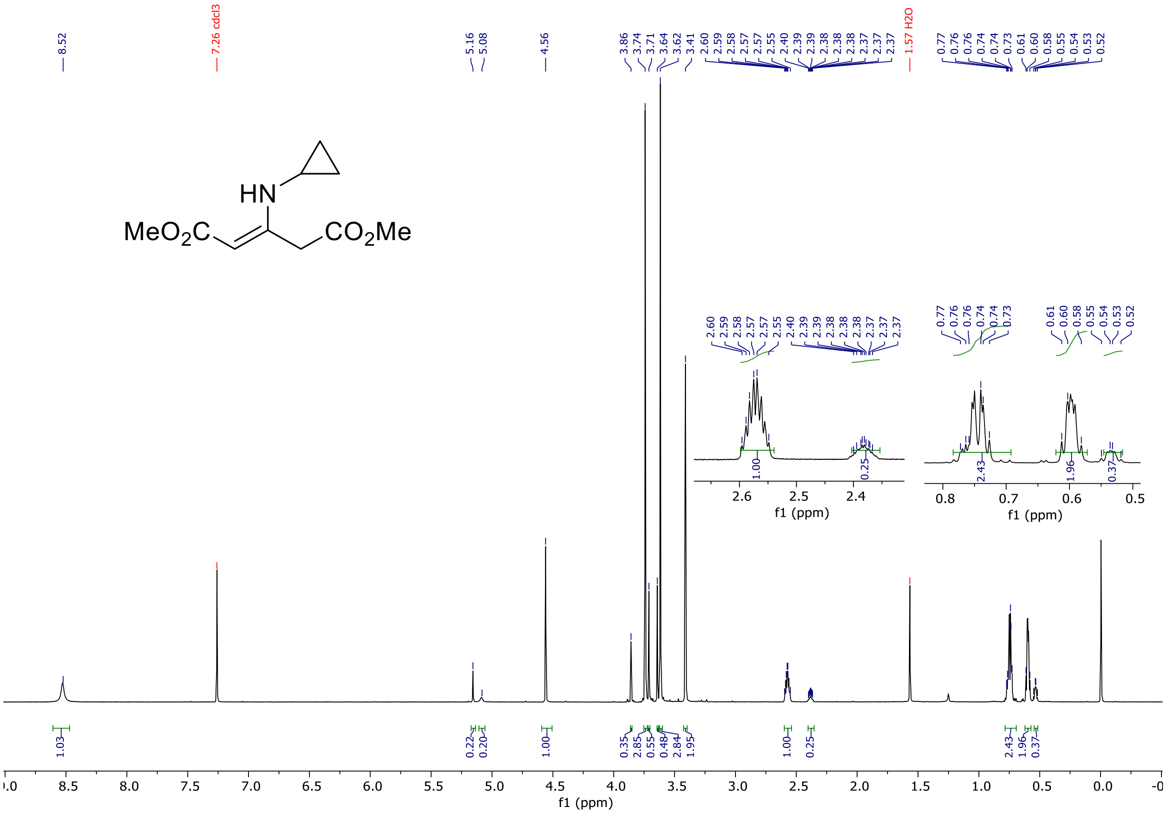

[13]*C NMR (CDCl$_3$, 126 MHz)*

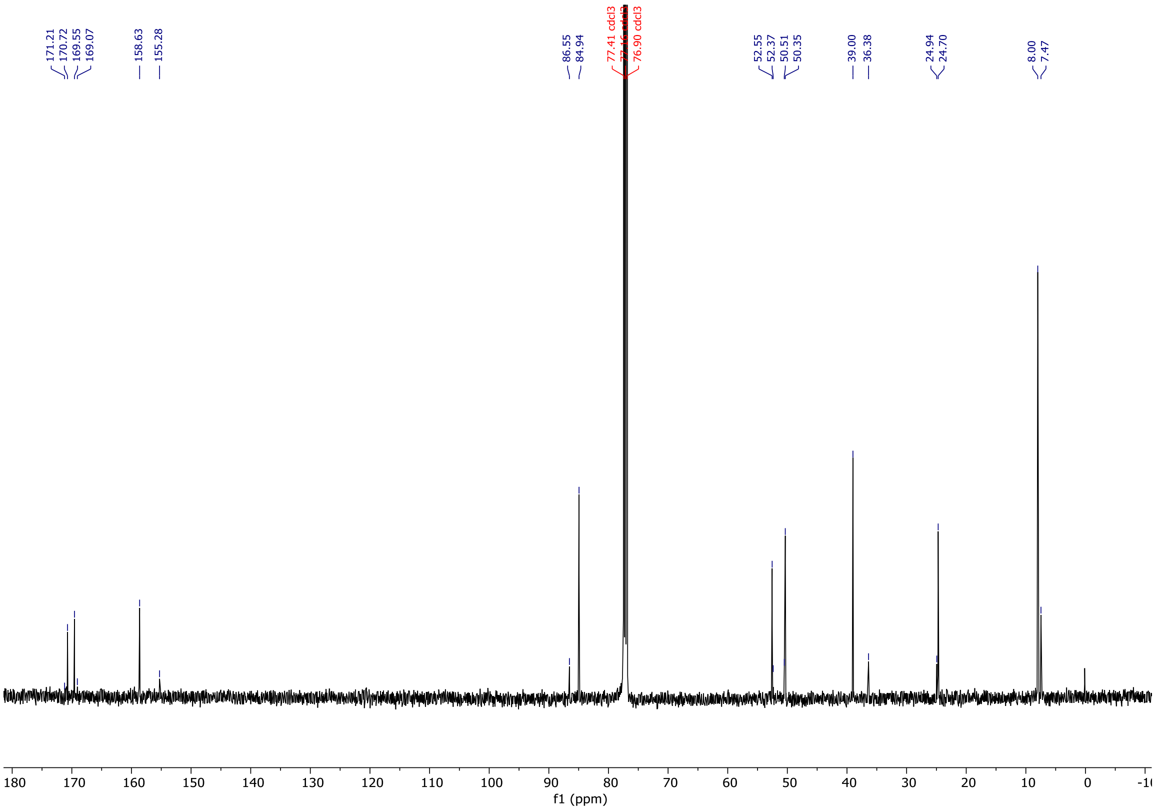

dimethyl 3-(cyclopropylamino)pent-2-enedioate (6d)

^{1}*H NMR (CDCl$_3$, 600 MHz)*, ratio of diasteroisomers: 80:20.

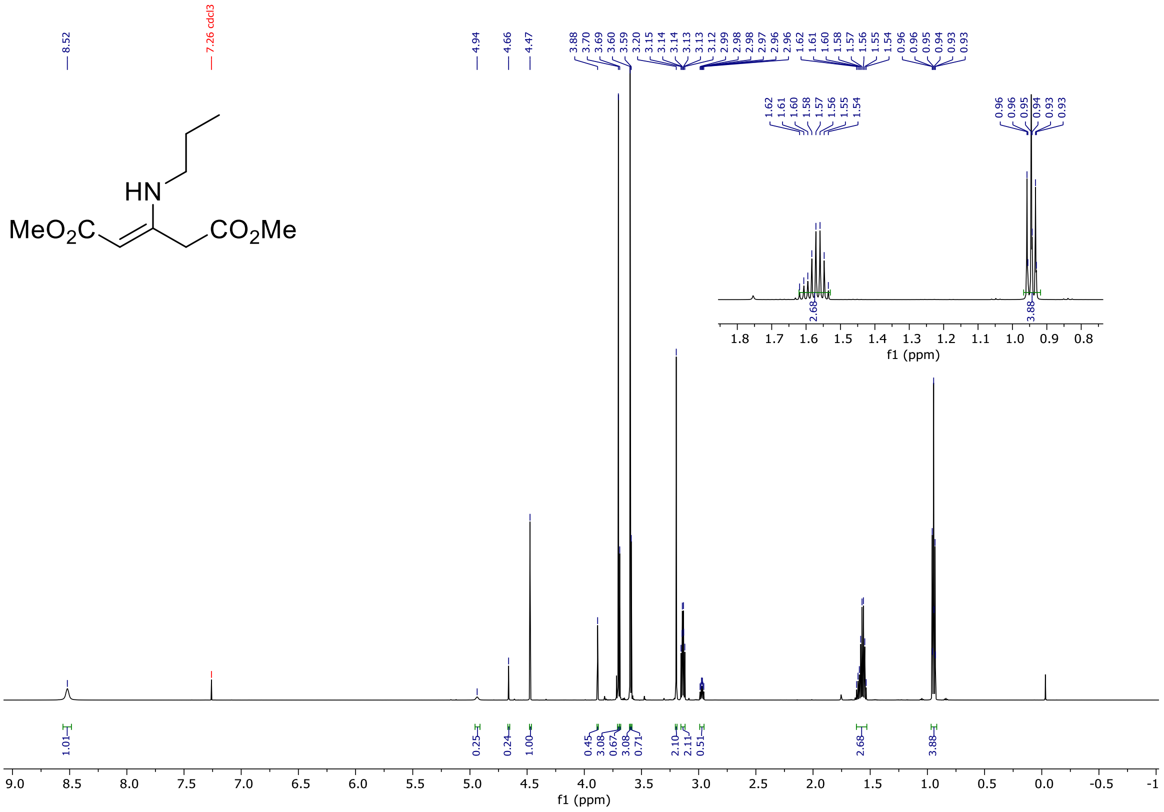

^{13}*C NMR (CDCl$_3$, 151 MHz)*

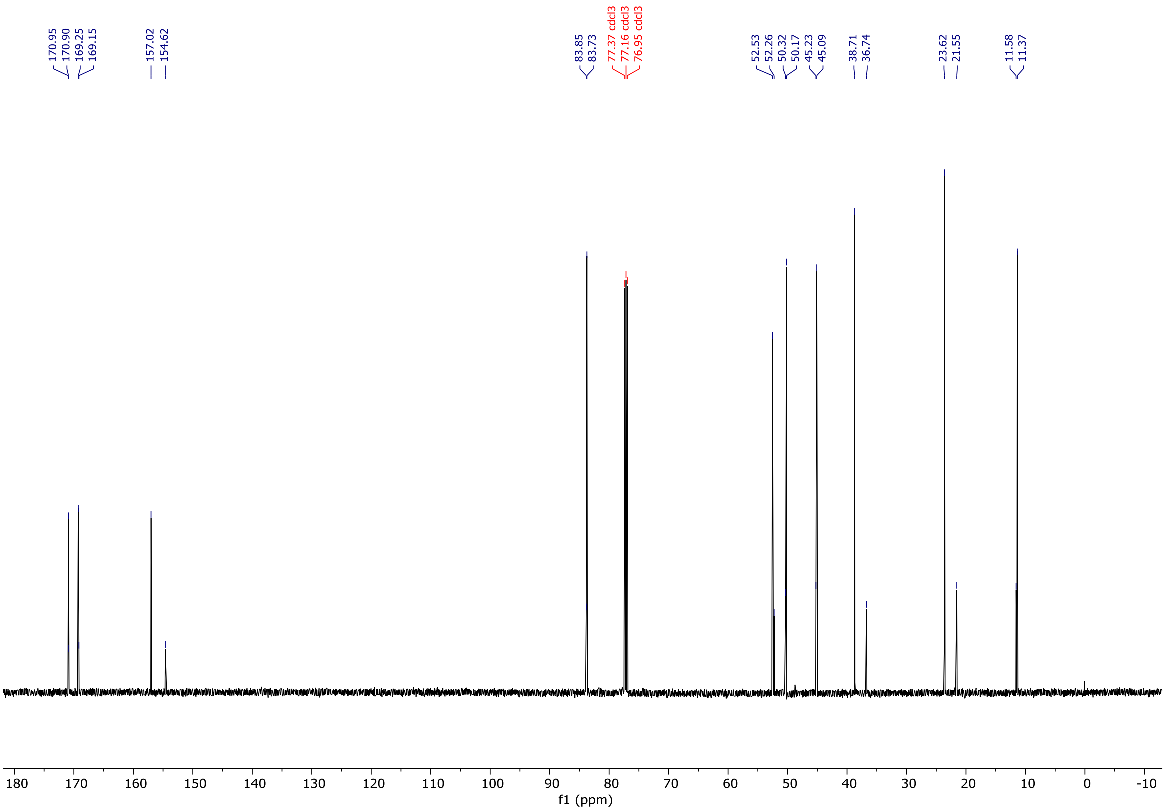

methyl 1-benzyl-5-(1-diazo-2-methoxy-2-oxoethyl)-1H-1,2,3-triazole-4-carboxylate (7a)

^{1}H NMR ($CDCl_3$, 500 MHz)

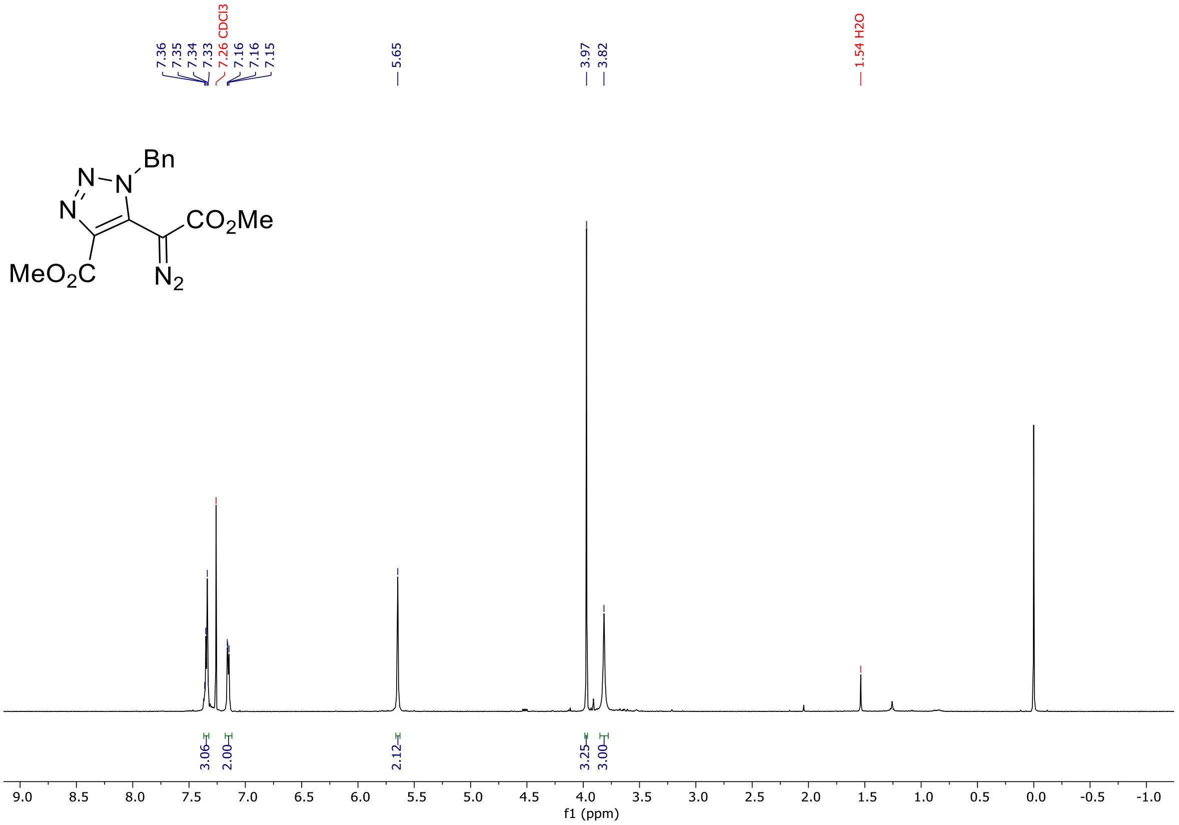

^{13}C NMR ($CDCl_3$, 151 MHz)

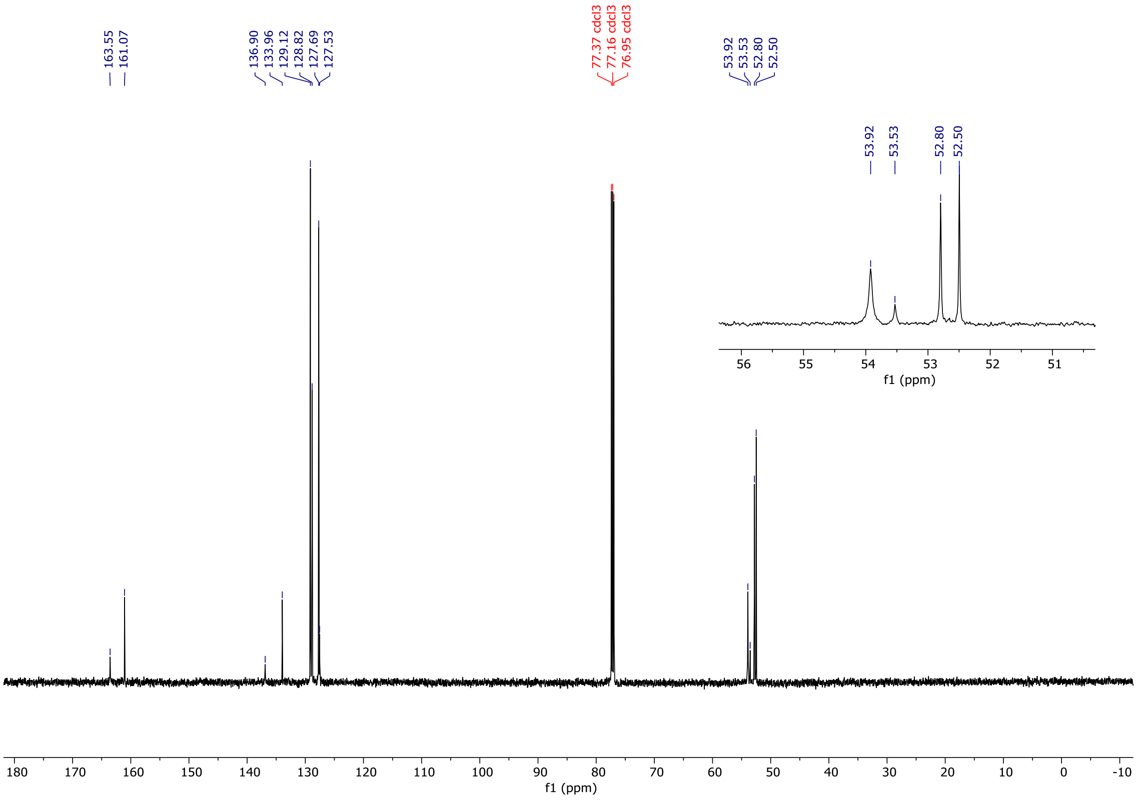

IR (cm⁻¹)

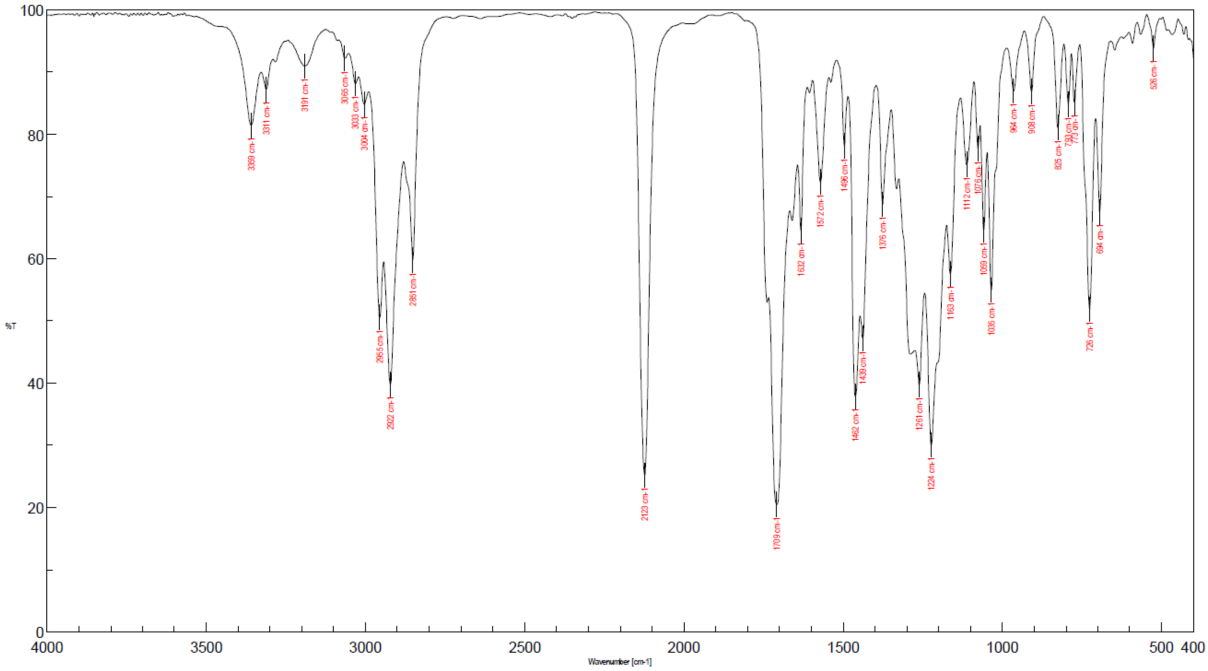

100
80
60
40
20
0
%T
4000
3500
3000
2500
2000
1500
1000
500
400
Wavenumber [cm-1]
3369 cm-1
3311 cm-1
3191 cm-1
3065 cm-1
3033 cm-1
3004 cm-1
2955 cm-1
2922 cm-1
2861 cm-1
2123 cm-1
1709 cm-1
1632 cm-1
1572 cm-1
1496 cm-1
1462 cm-1
1439 cm-1
1376 cm-1
1261 cm-1
1224 cm-1
1163 cm-1
1112 cm-1
1059 cm-1
1035 cm-1
964 cm-1
908 cm-1
825 cm-1
773 cm-1
726 cm-1
694 cm-1
526 cm-1

methyl 5-(1-diazo-2-methoxy-2-oxoethyl)-1-phenyl-1H-1,2,3-triazole-4-carboxylate (7b)

^{1}H NMR ($CDCl_3$, 500 MHz)

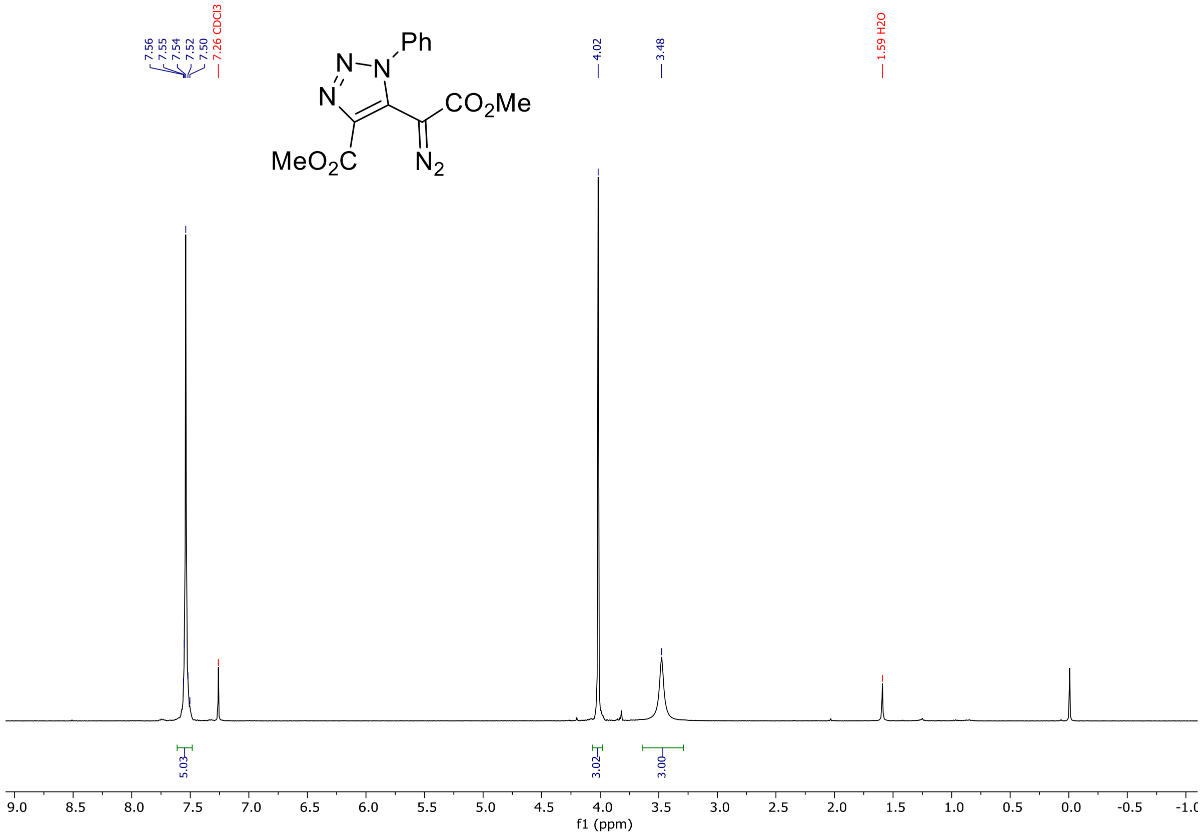

^{13}C NMR ($CDCl_3$, 126 MHz)

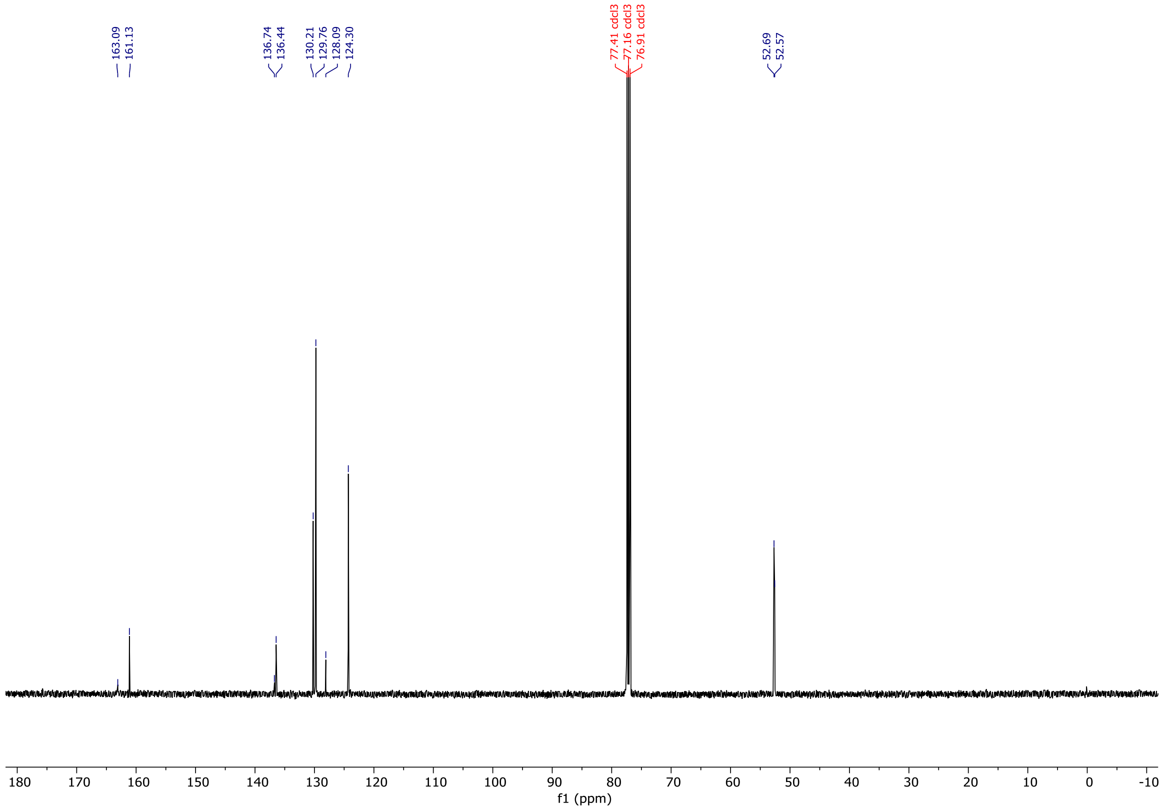

IR (cm^{-1})

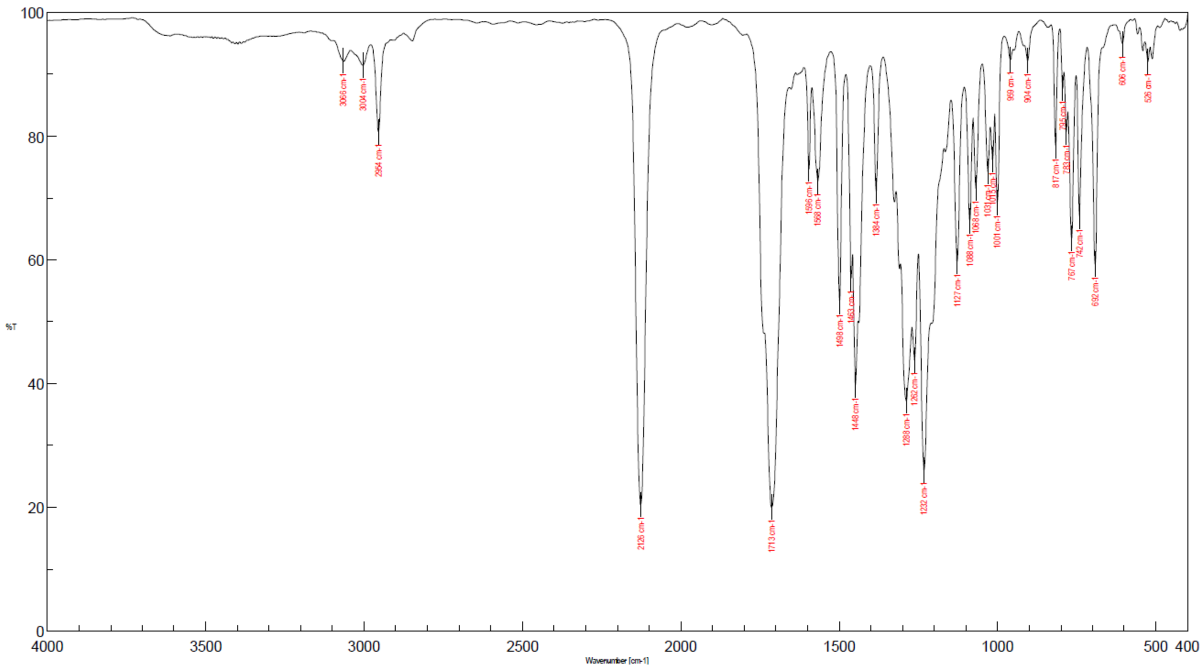
3066 cm-1
3004 cm-1
2954 cm-1
2126 cm-1
1713 cm-1
1596 cm-1
1568 cm-1
1498 cm-1
1463 cm-1
1448 cm-1
1384 cm-1
1288 cm-1
1262 cm-1
1232 cm-1
1127 cm-1
1088 cm-1
1068 cm-1
1001 cm-1
969 cm-1
904 cm-1
817 cm-1
783 cm-1
767 cm-1
752 cm-1
692 cm-1
606 cm-1
526 cm-1
%T
Wavenumber [cm-1]

methyl 1-cyclopropyl-5-(1-diazo-2-methoxy-2-oxoethyl)-1H-1,2,3-triazole-4-carboxylate (7c)

^{1}H NMR (CDCl$_3$, 500 MHz)

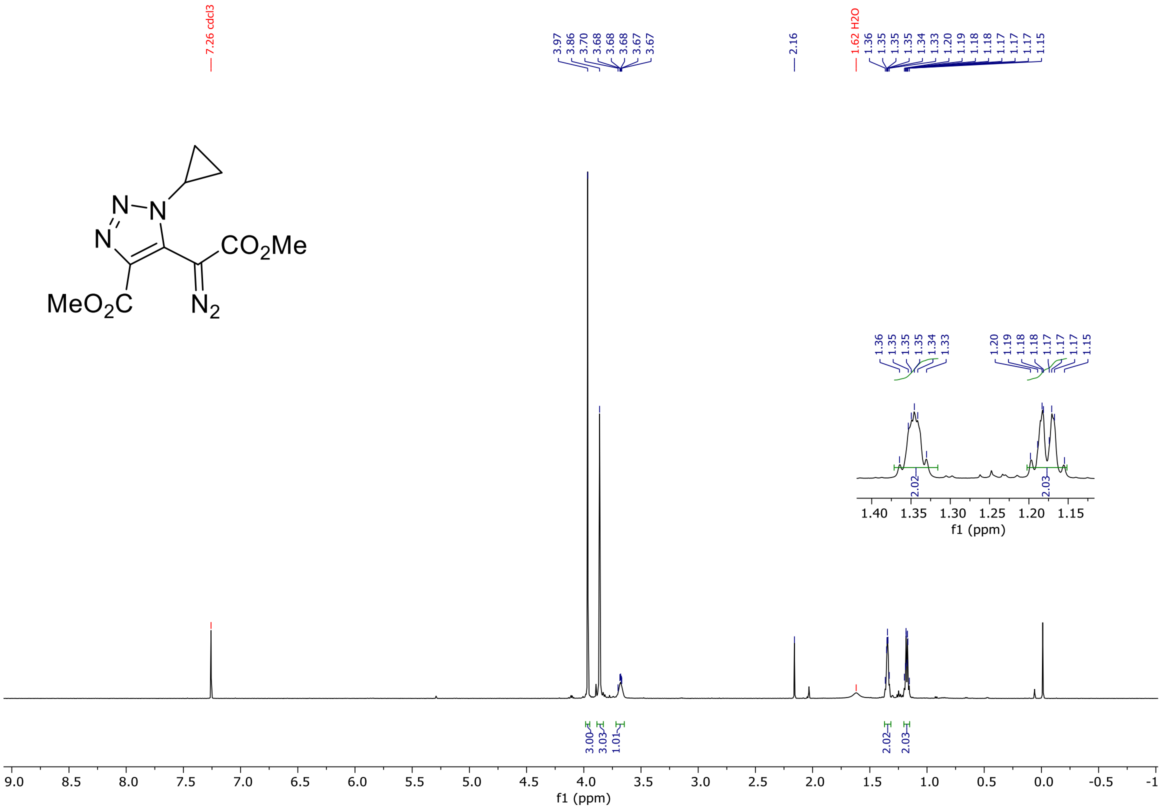

^{13}C NMR (CDCl$_3$, 126 MHz)

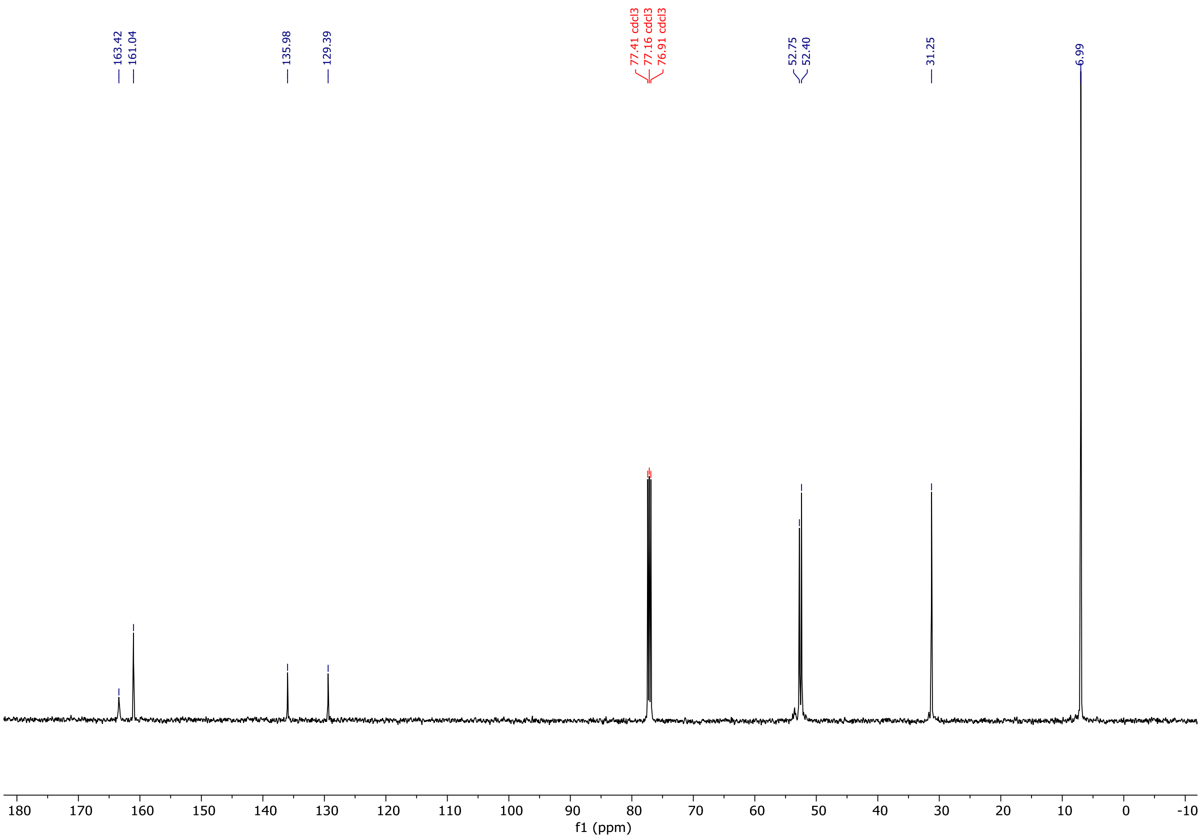

IR (cm^{-1})

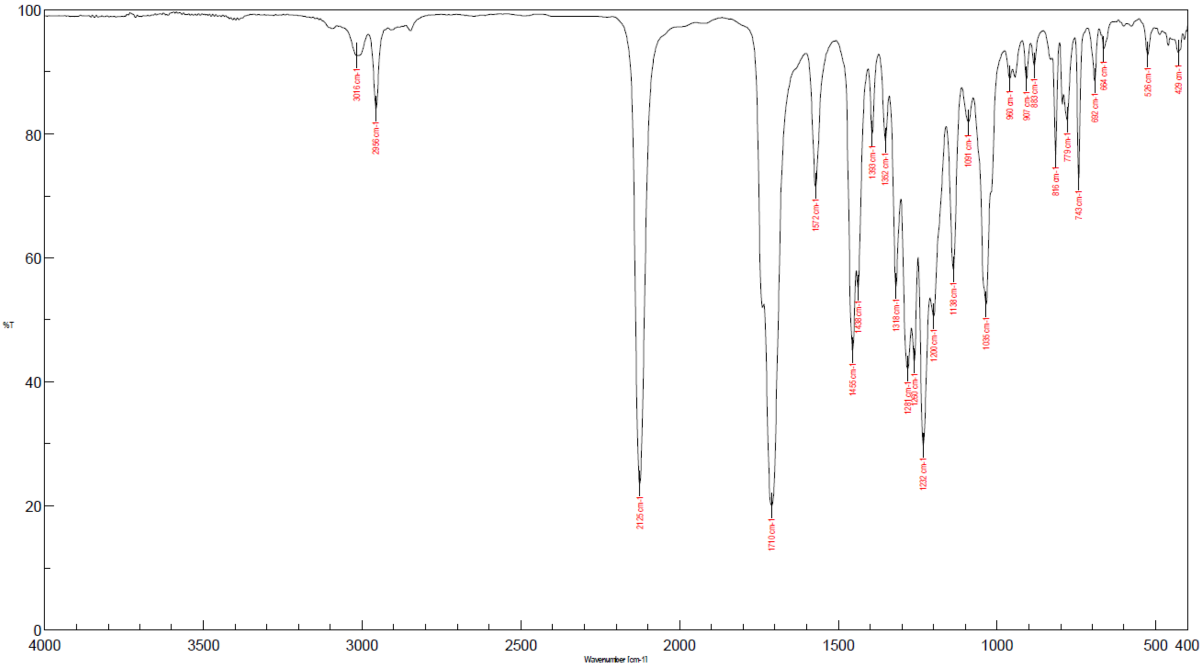

100
80
60
40
20
0
%T
4000
3500
3000
2500
2000
1500
1000
500
400
Wavenumber [cm-1]
3016 cm-1
2956 cm-1
2125 cm-1
1710 cm-1
1572 cm-1
1455 cm-1
1438 cm-1
1390 cm-1
1352 cm-1
1318 cm-1
1281 cm-1
1260 cm-1
1232 cm-1
1200 cm-1
1138 cm-1
1091 cm-1
1035 cm-1
960 cm-1
907 cm-1
883 cm-1
816 cm-1
779 cm-1
743 cm-1
692 cm-1
664 cm-1
526 cm-1
429 cm-1

methyl 5-(1-diazo-2-methoxy-2-oxoethyl)-1-propyl-1H-1,2,3-triazole-4-carboxylate (7d)

^{1}H NMR ($CDCl_3$, 500 MHz)

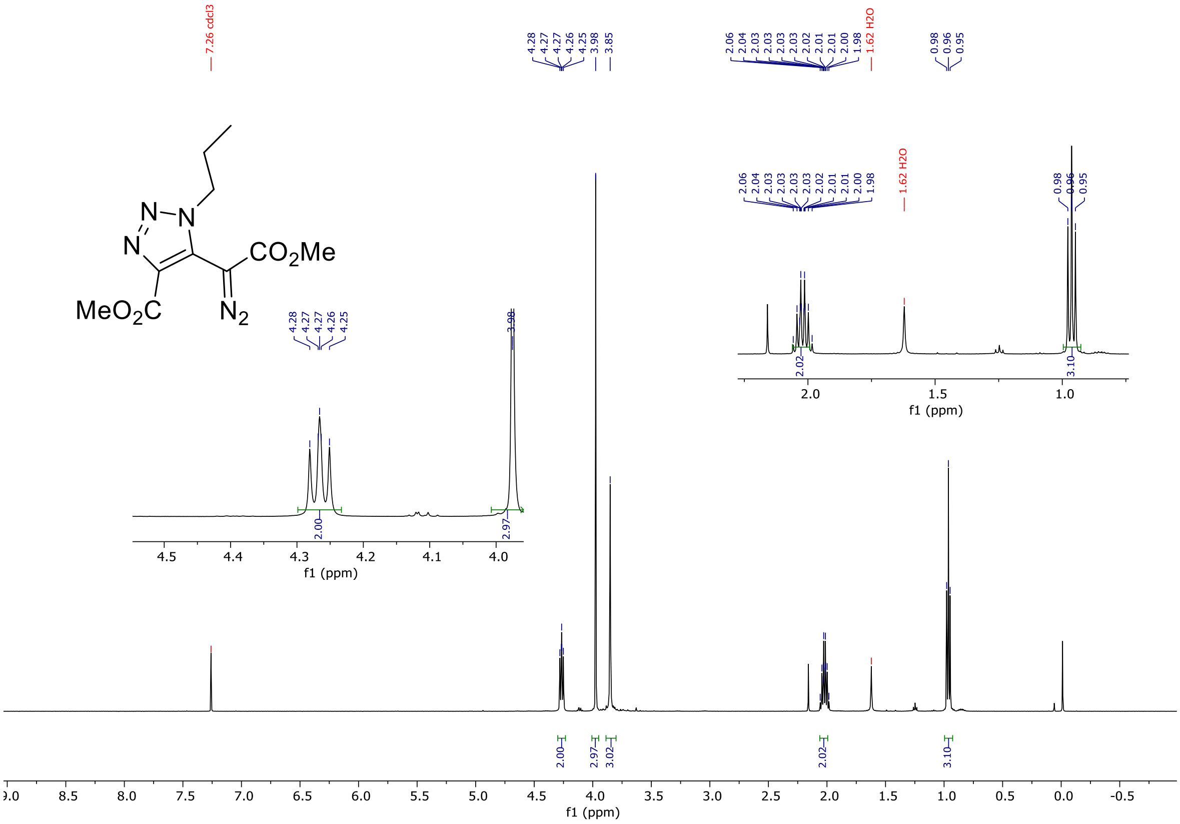

^{13}C NMR ($CDCl_3$, 126 MHz)

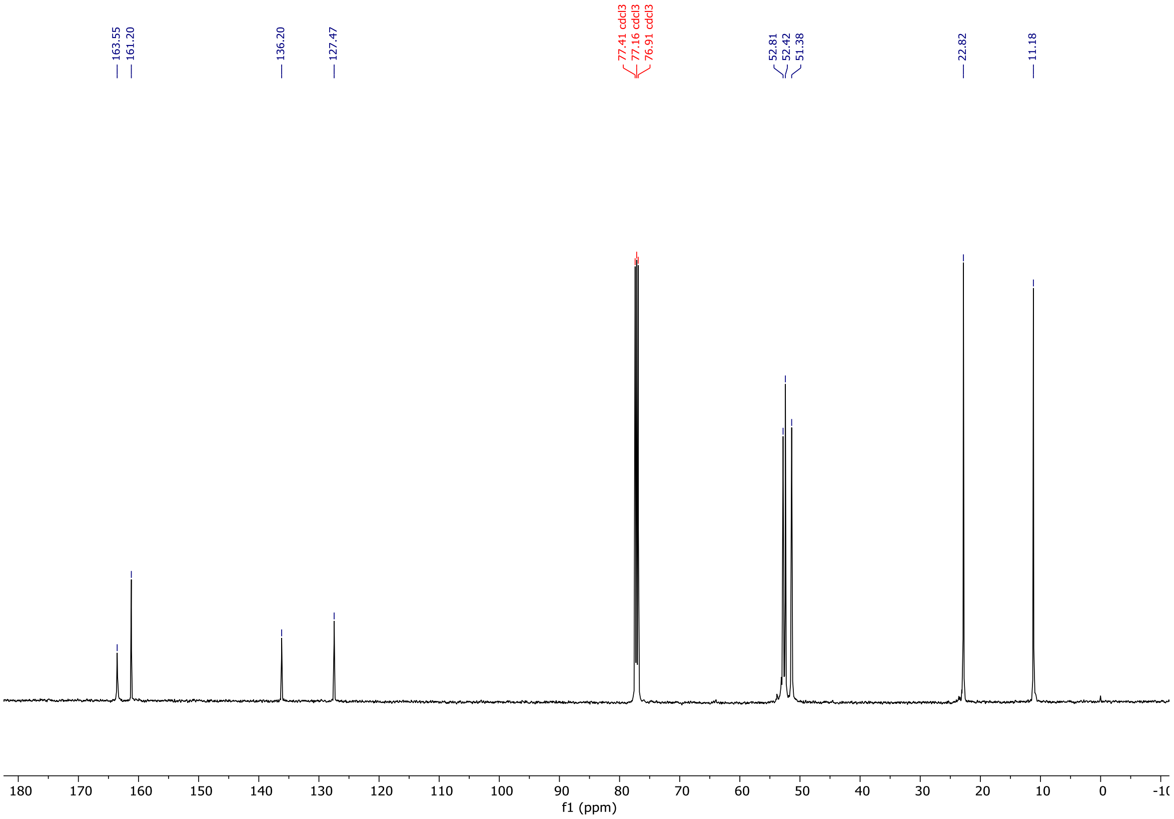

IR (cm^{-1})

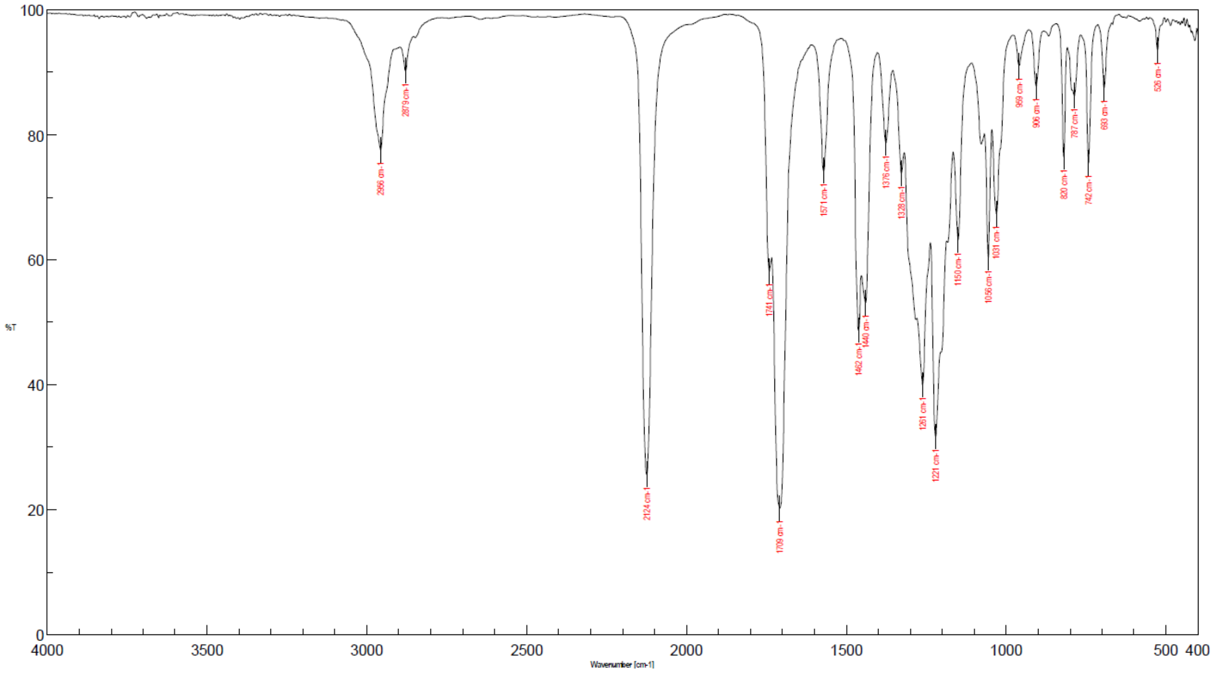

100
80
60
%T
40
20
0
4000
3500
3000
2500
2000
1500
1000
500
400
Wavenumber [cm-1]
2956 cm-1
2879 cm-1
2124 cm-1
1741 cm-1
1709 cm-1
1571 cm-1
1462 cm-1
1440 cm-1
1376 cm-1
1328 cm-1
1261 cm-1
1221 cm-1
1150 cm-1
1056 cm-1
1031 cm-1
969 cm-1
906 cm-1
820 cm-1
787 cm-1
742 cm-1
693 cm-1
526 cm-1

methyl 1-benzyl-5-(2,3-dichloro-1-methoxy-1-oxopropan-2-yl)-1H-1,2,3-triazole-4-carboxylate (9a)

^{1}H NMR ($CDCl_3$, 600 MHz)

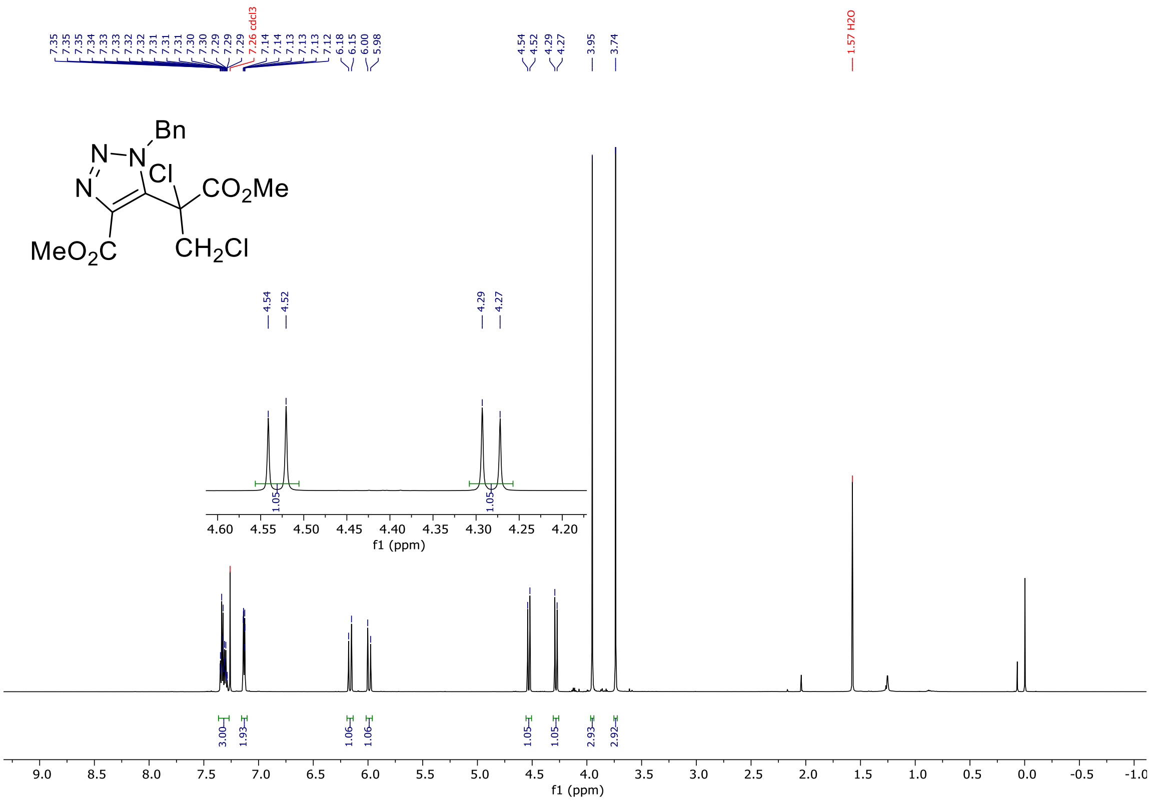

^{13}C NMR ($CDCl_3$, 151 MHz)

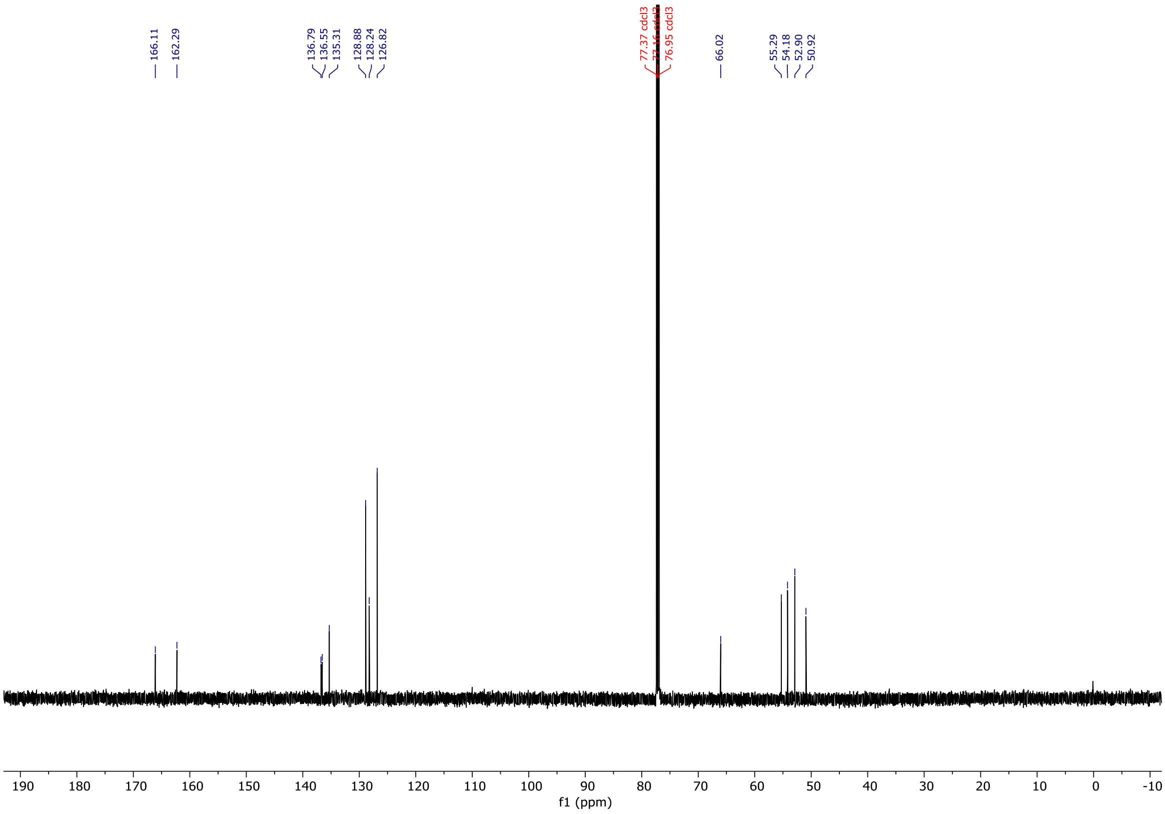

methyl 1-benzyl-5-(1-chloro-2-methoxy-2-oxoethyl)-1H-1,2,3-triazole-4-carboxylate (10a)

^{1}H NMR ($CDCl_3$, 500 MHz)

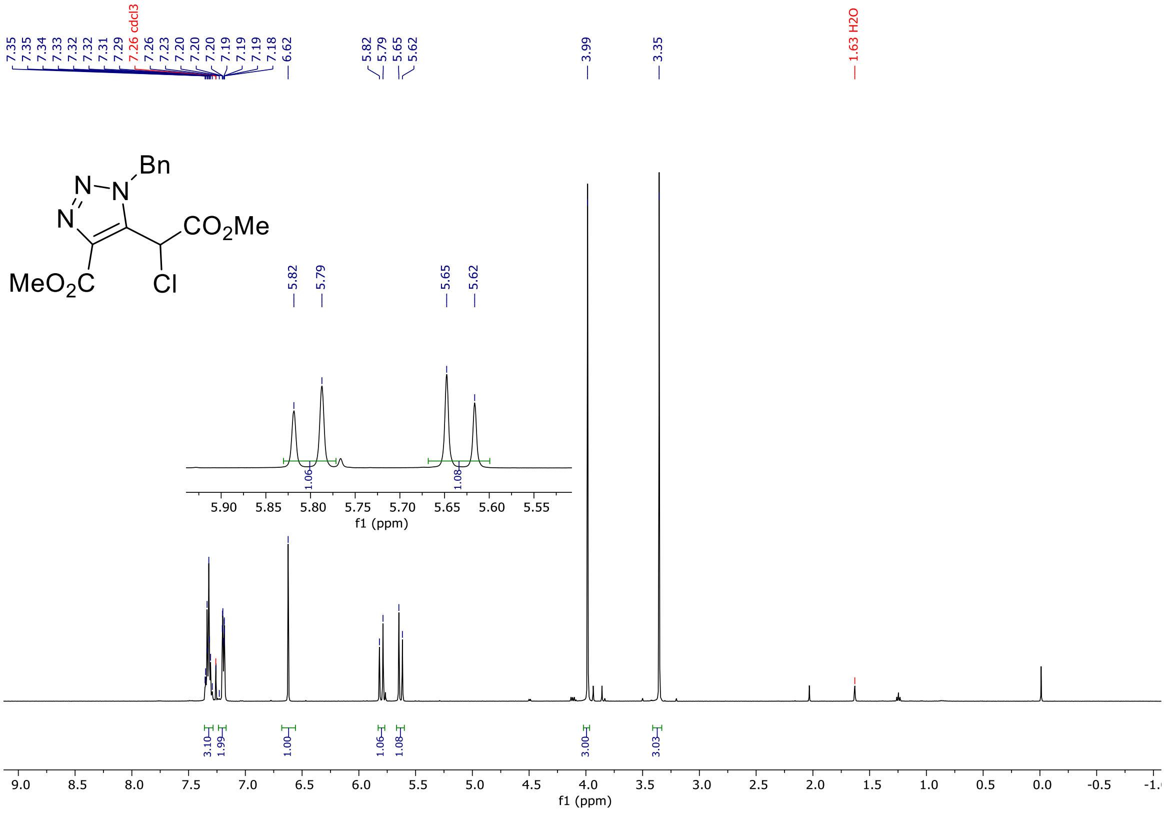

^{13}C NMR ($CDCl_3$, 126 MHz)

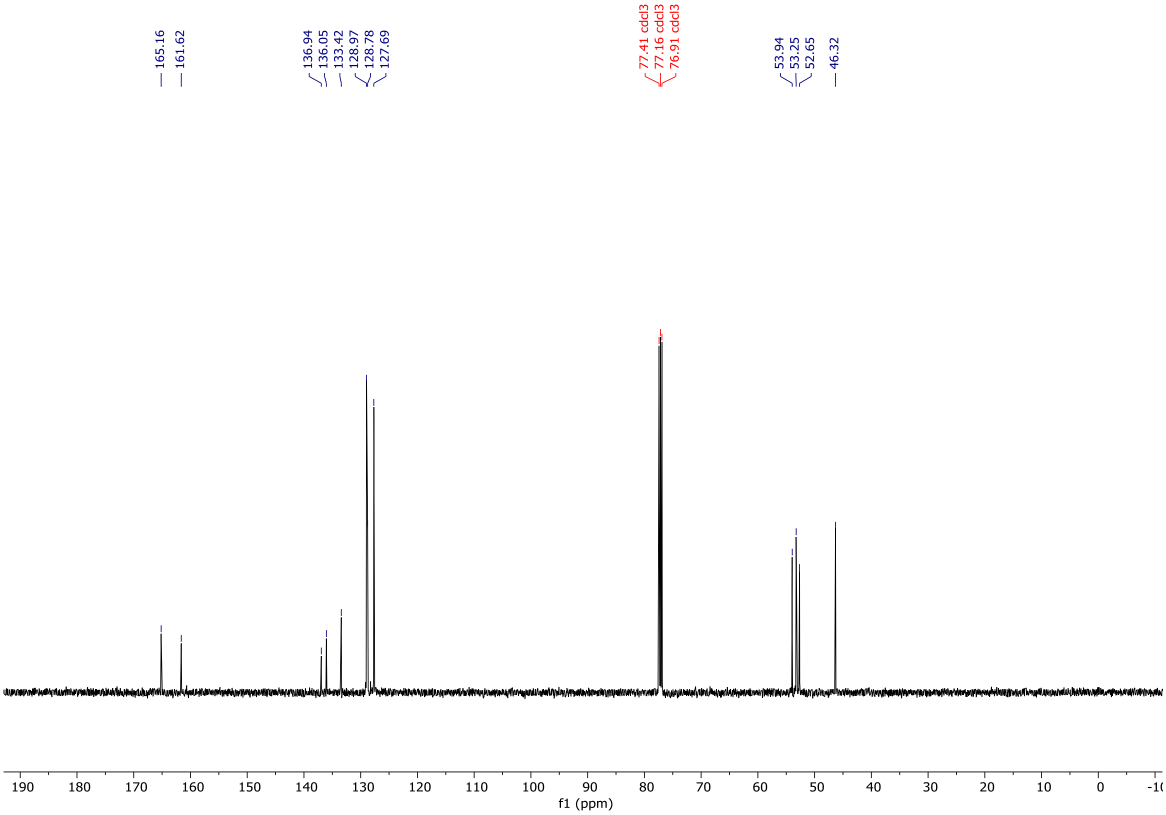

methyl 5-(2,3-dichloro-1-methoxy-1-oxopropan-2-yl)-1-phenyl-1H-1,2,3-triazole-4-carboxylate (9b)

^{1}H NMR ($CDCl_3$, 600 MHz)

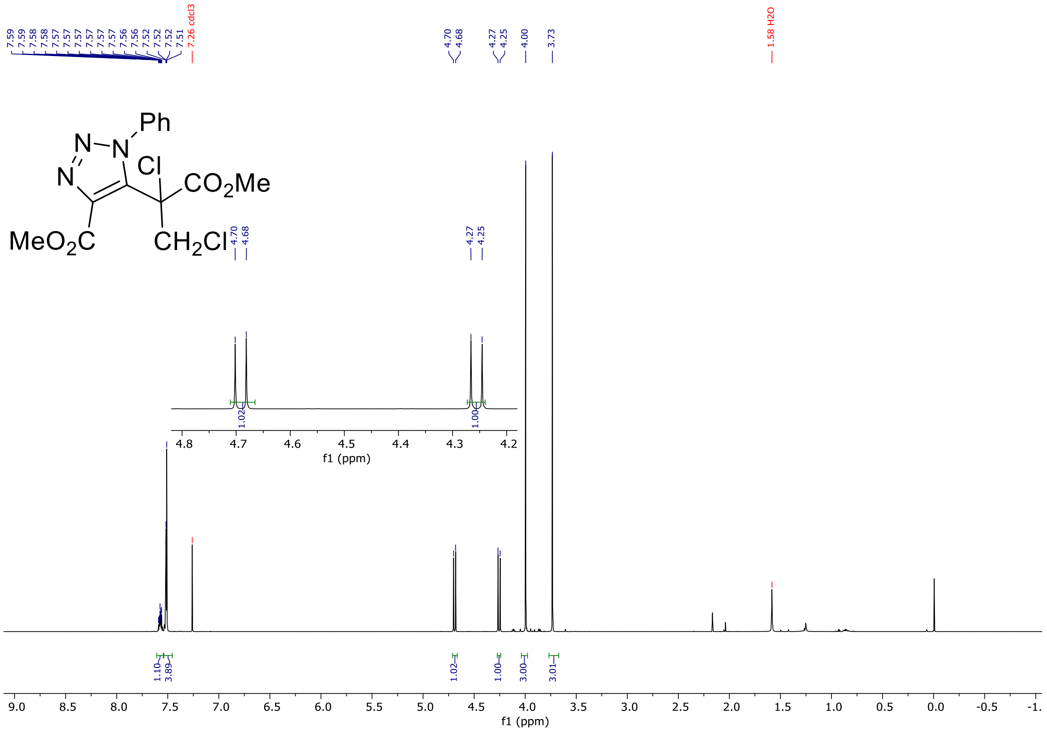

^{13}C NMR ($CDCl_3$, 151 MHz)

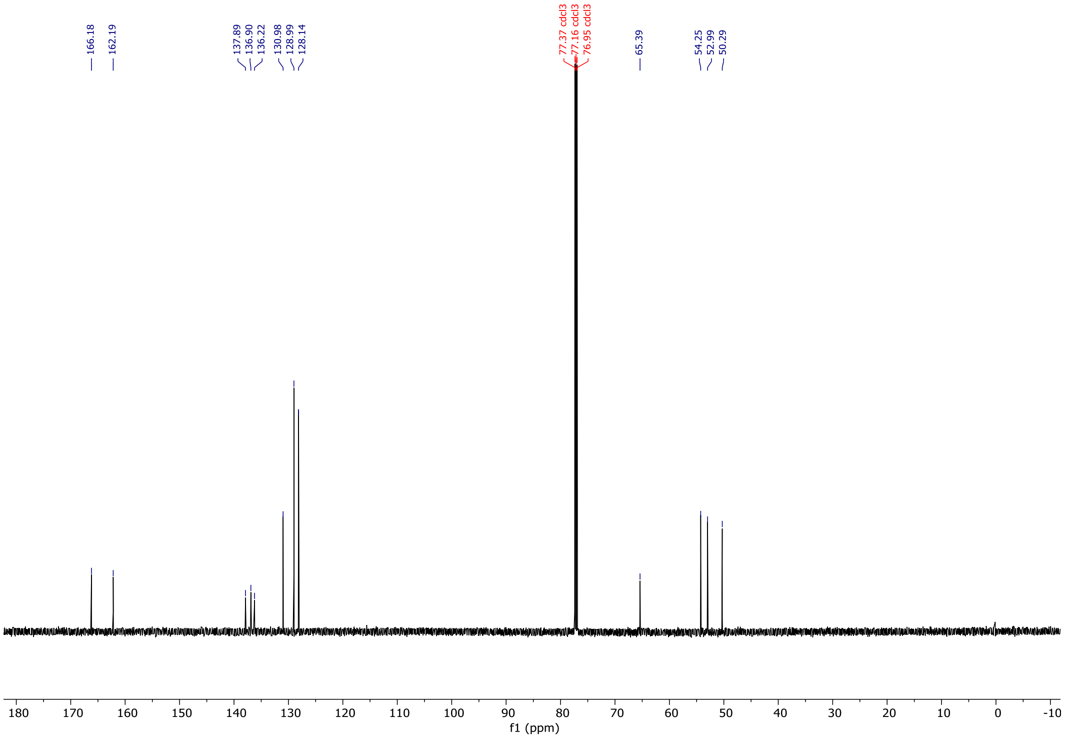

methyl 5-(1-chloro-2-methoxy-2-oxoethyl)-1-phenyl-1H-1,2,3-triazole-4-carboxylate (10b)

^{1}H NMR ($CDCl_3$, 500 MHz)

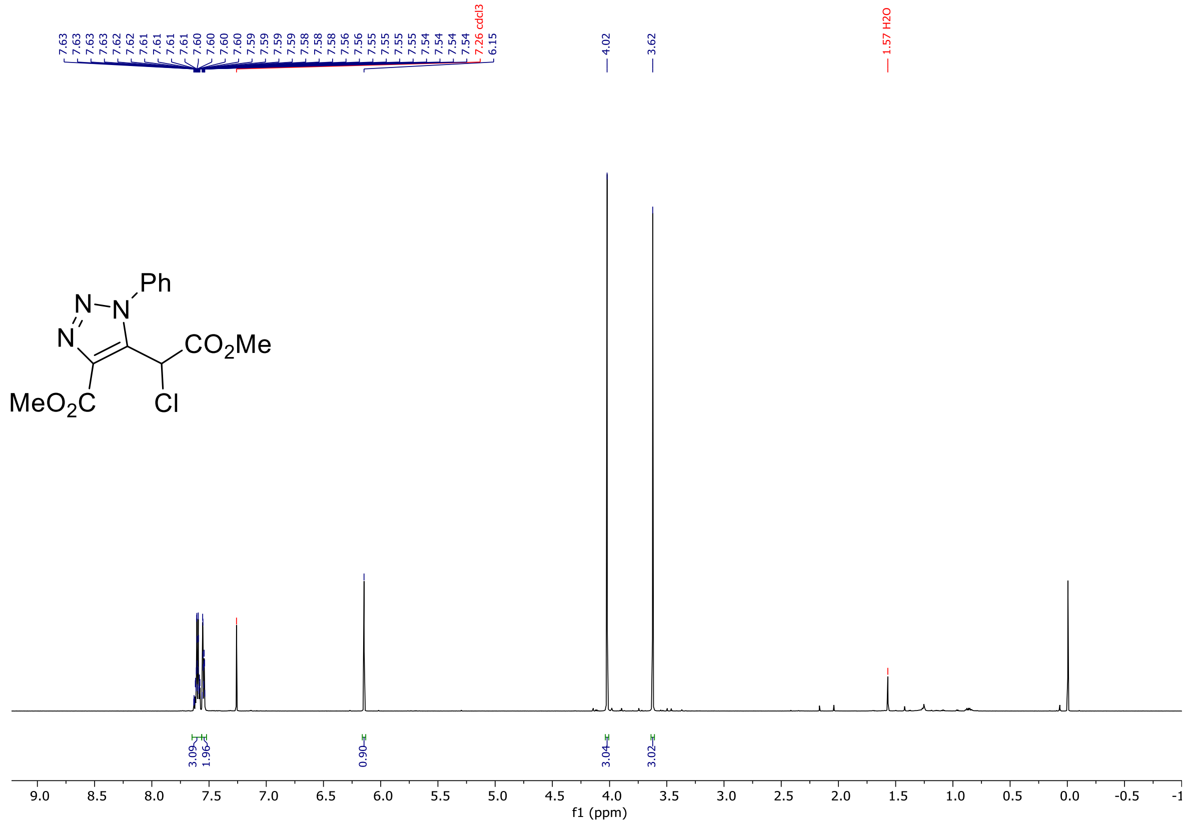

^{13}C NMR ($CDCl_3$, 126 MHz)

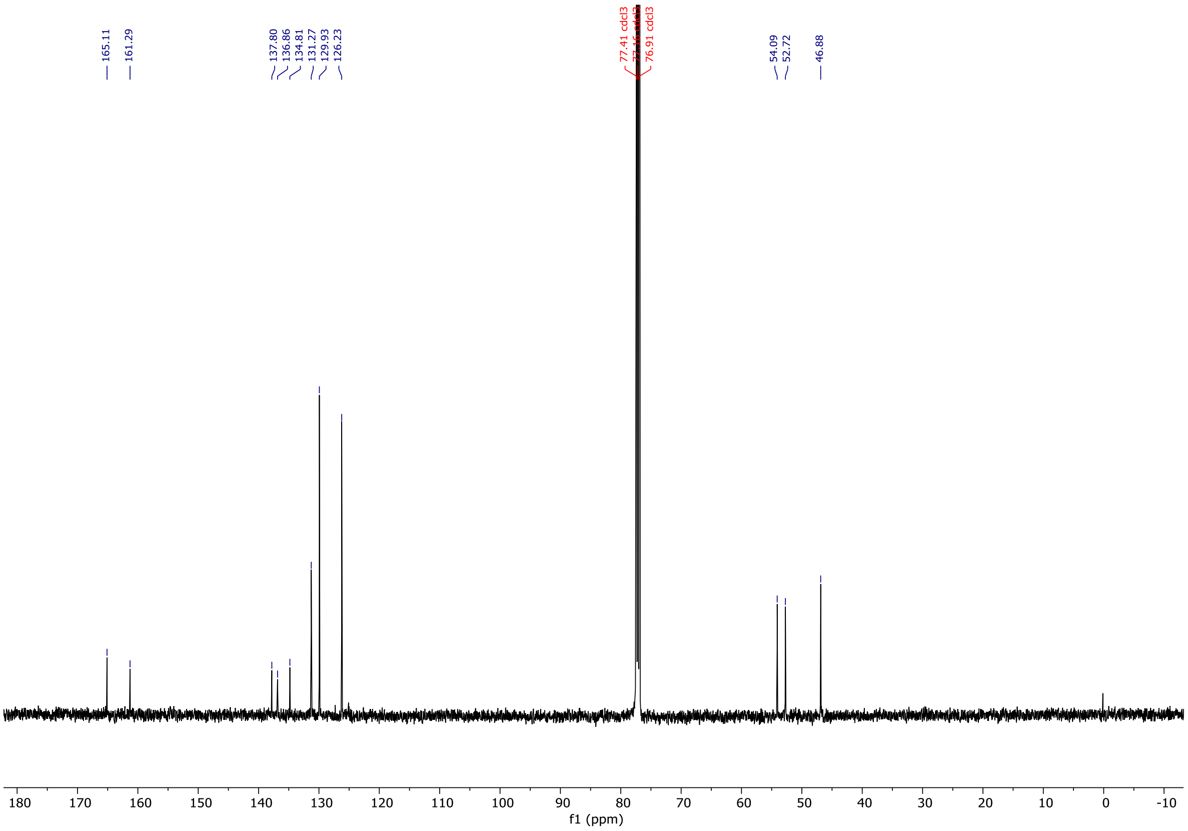

methyl 1-cyclopropyl-5-(2,3-dichloro-1-methoxy-1-oxopropan-2-yl)-1H-1,2,3-triazole-4-carboxylate (9c)

^{1}H NMR (CDCl$_3$, 600 MHz)

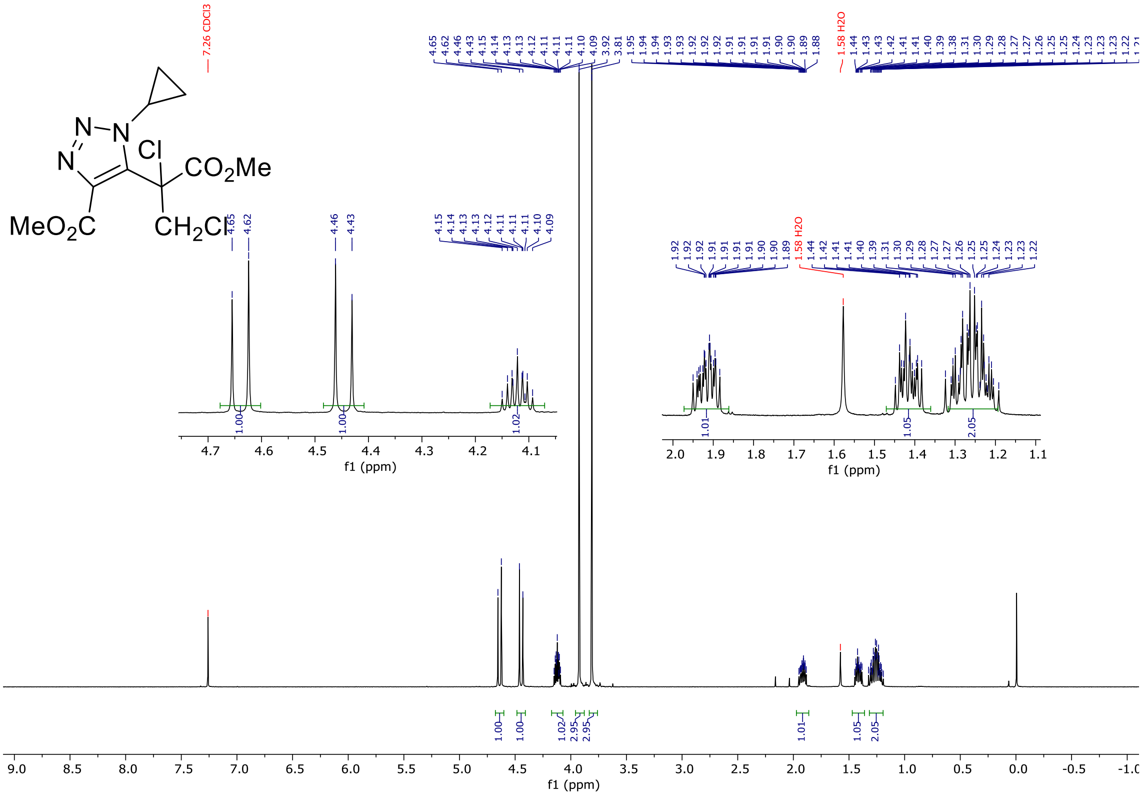

^{13}C NMR (CDCl$_3$, 126 MHz)

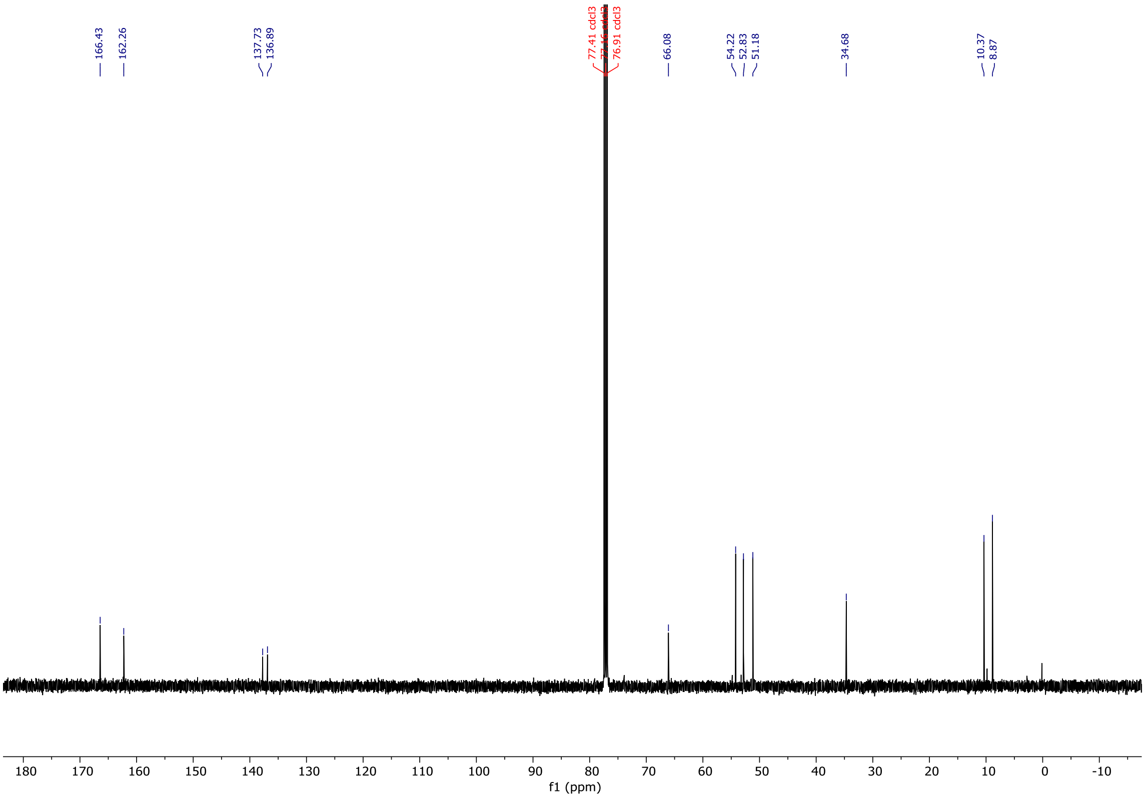

methyl 5-(1-chloro-2-methoxy-2-oxoethyl)-1-cyclopropyl-1H-1,2,3-triazole-4-carboxylate (10c)

^{1}H NMR ($CDCl_3$, 500 MHz)

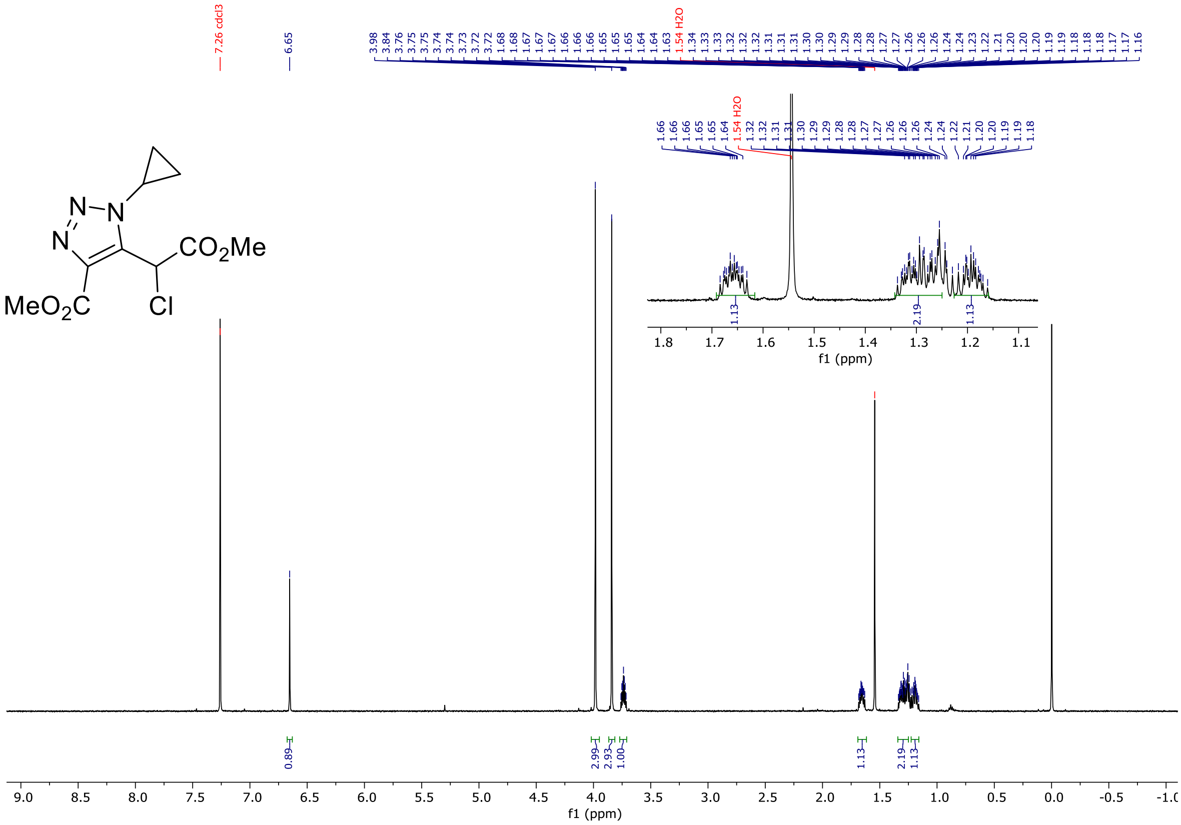

^{13}C NMR ($CDCl_3$, 151 MHz)

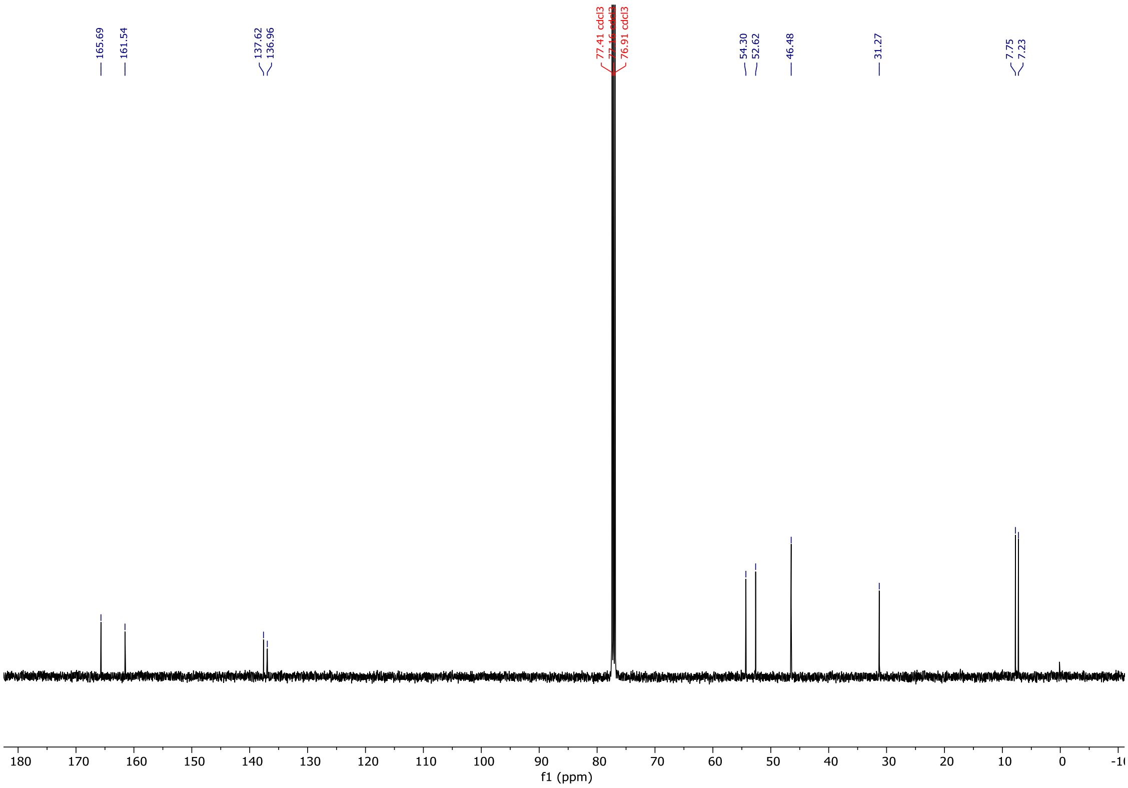

methyl 5-(2,3-dichloro-1-methoxy-1-oxopropan-2-yl)-1-propyl-1H-1,2,3-triazole-4-carboxylate (9d)

^{1}H NMR ($CDCl_3$, 500 MHz)

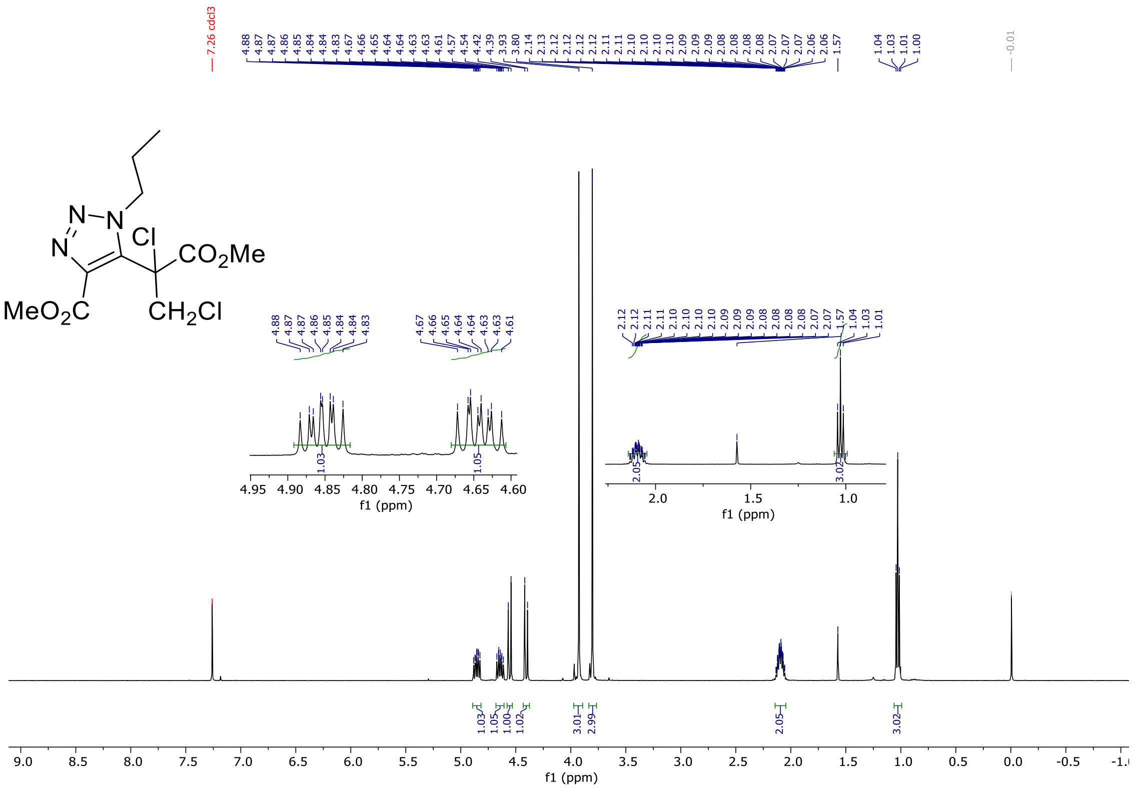

^{13}C NMR ($CDCl_3$, 126 MHz)

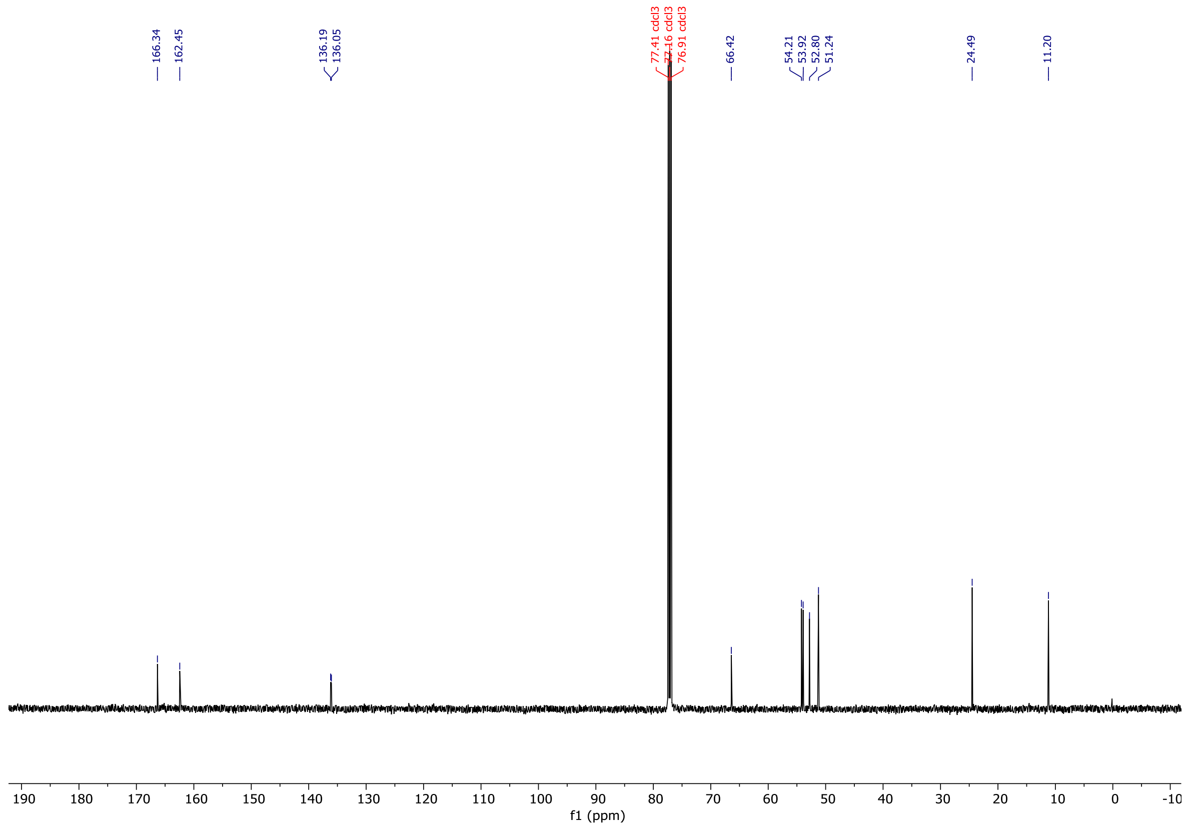

methyl 5-(1-chloro-2-methoxy-2-oxoethyl)-1-propyl-1H-1,2,3-triazole-4-carboxylate (10d)

^{1}H NMR (CDCl$_3$, 600 MHz)

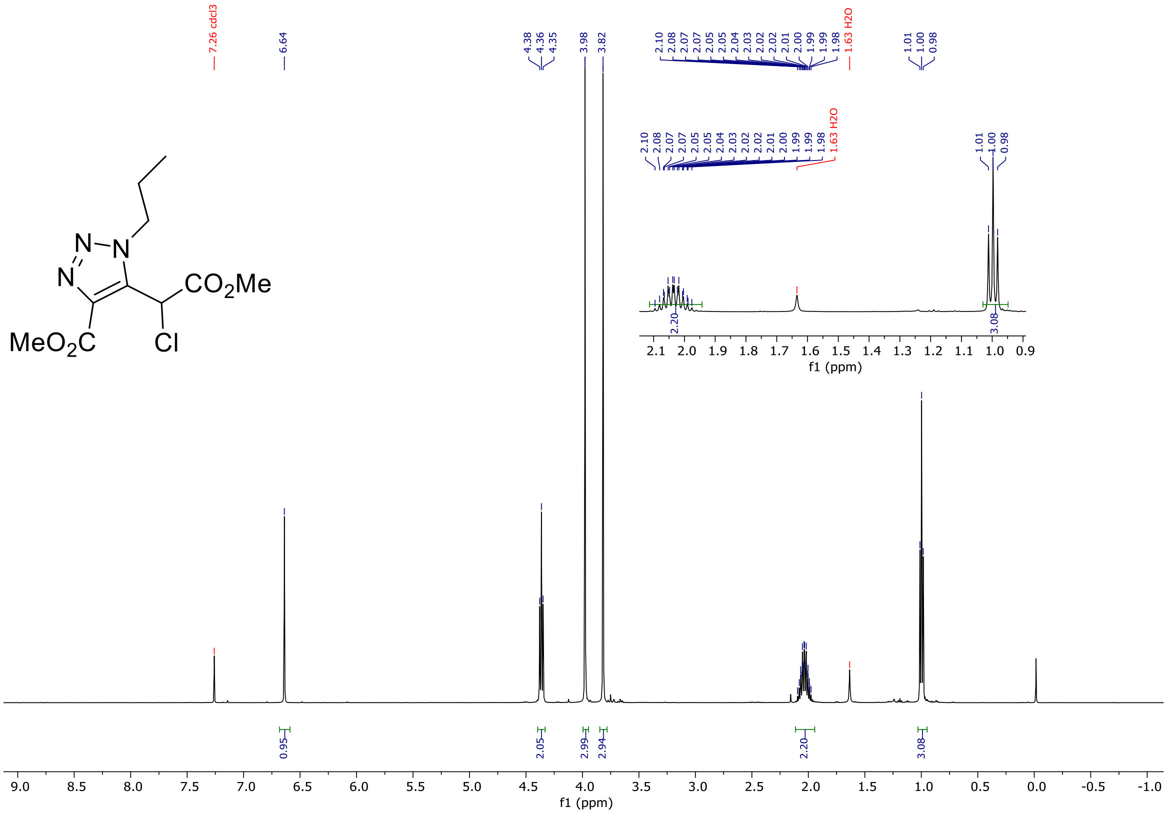

^{13}C NMR (CDCl$_3$, 151 MHz)

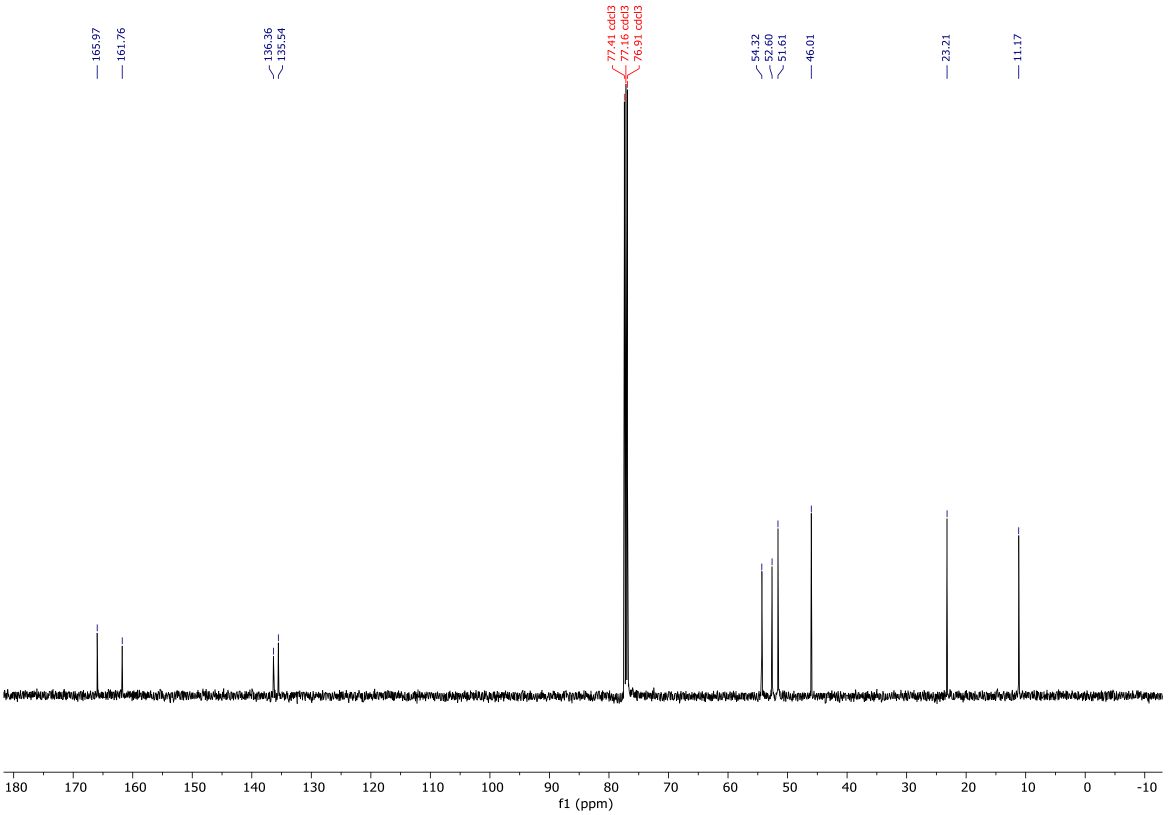

methyl 1-benzyl-5-(1,1,2-trichloro-3-methoxy-3-oxopropan-2-yl)-1H-1,2,3-triazole-4-carboxylate (11)

^{1}H NMR ($CDCl_3$, 500 MHz)

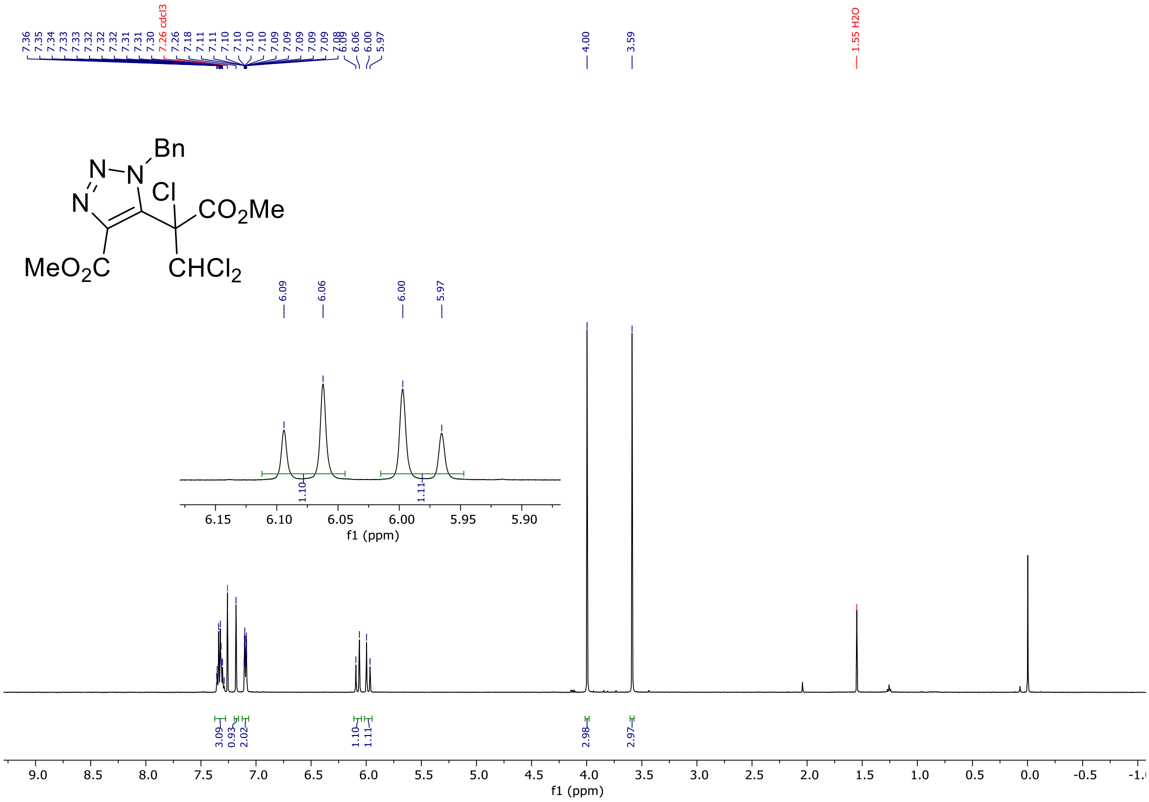

^{13}C NMR ($CDCl_3$, 126 MHz)

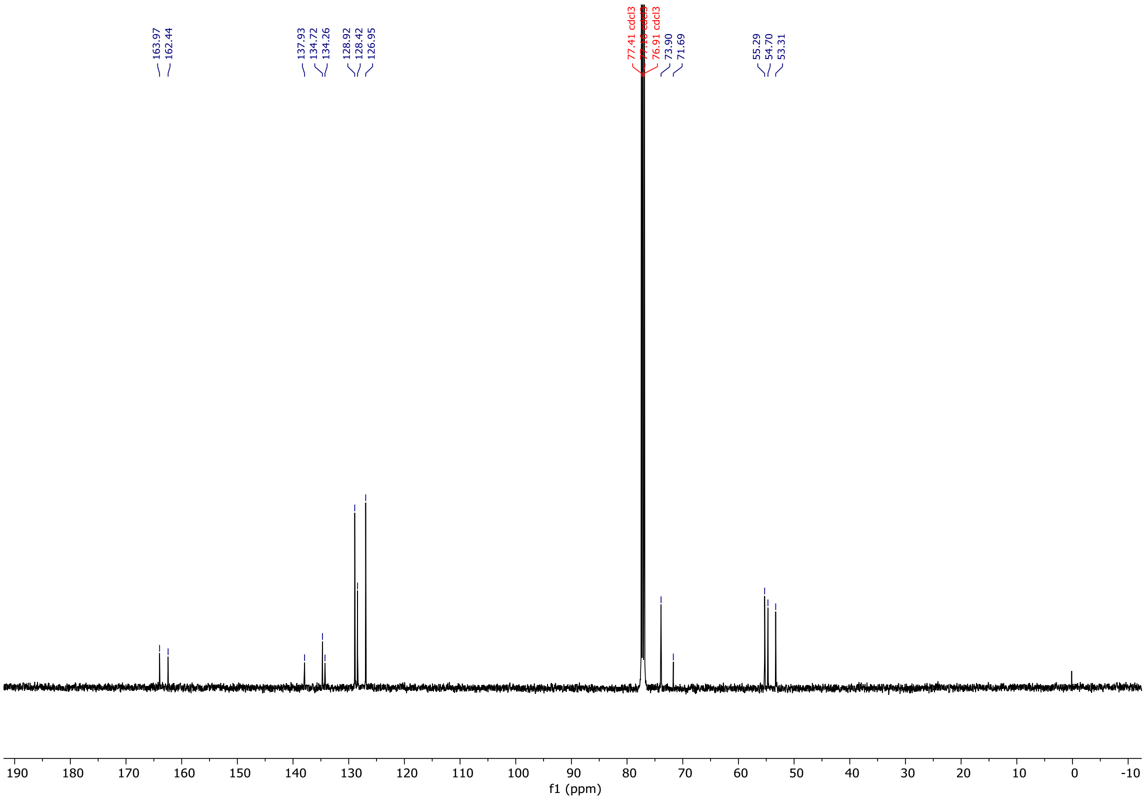

methyl 1-benzyl-5-(1-bromo-2-methoxy-2-oxoethyl)-1H-1,2,3-triazole-4-carboxylate (12)

^{1}H NMR ($CDCl_3$, 500 MHz)

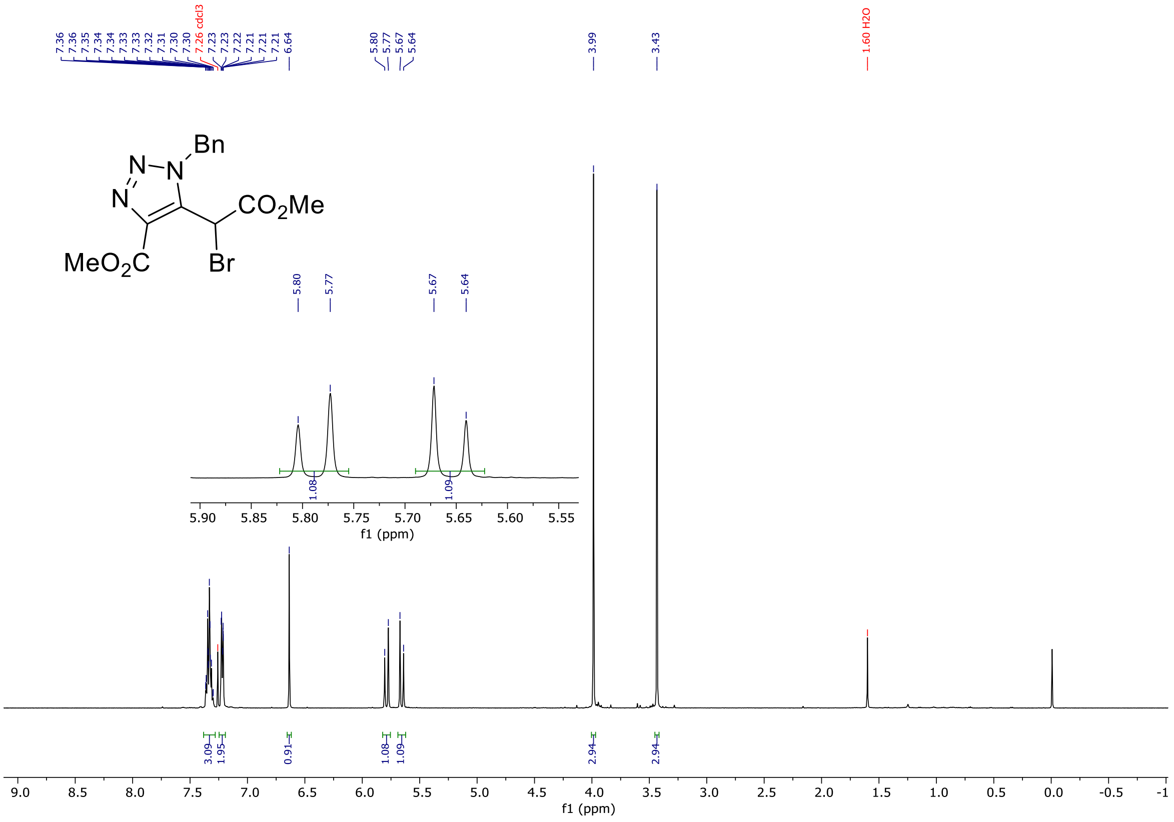

^{13}C NMR ($CDCl_3$, 126 MHz)

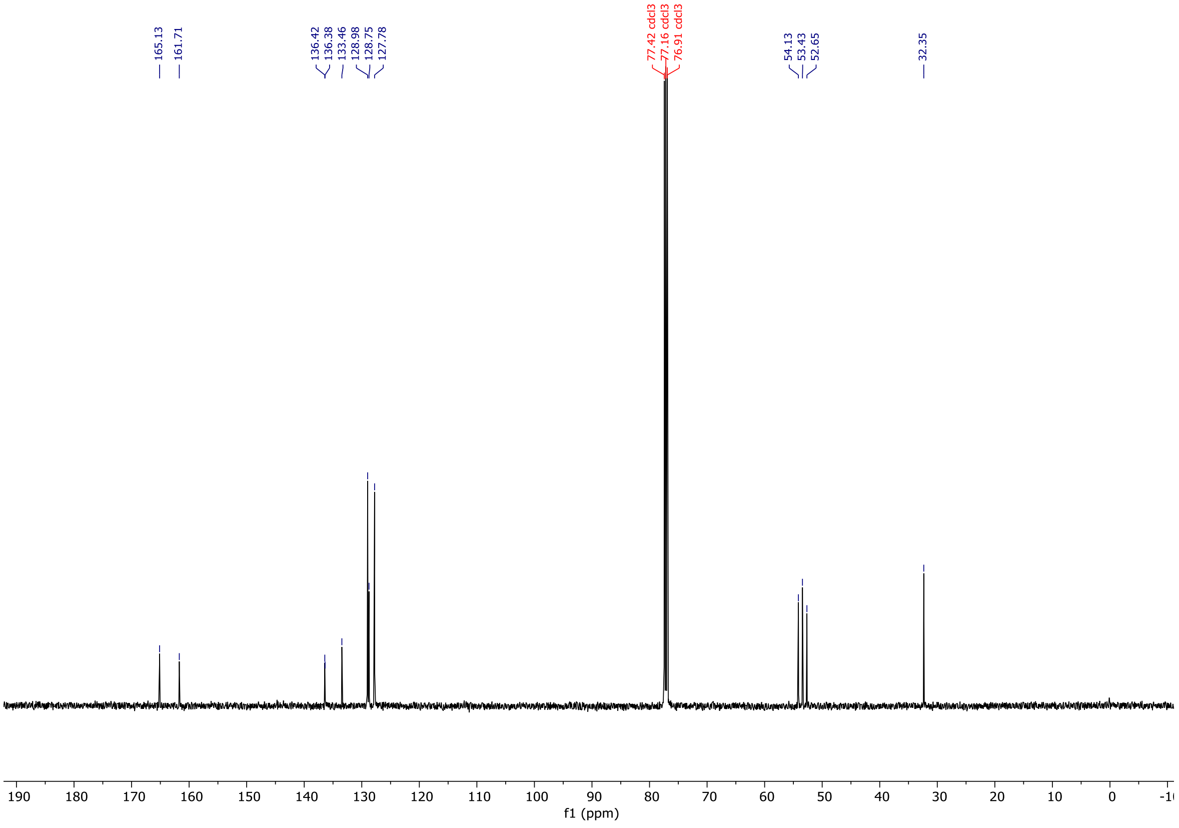

methyl 1-benzyl-5-(3-methoxy-3-oxoprop-1-en-2-yl)-1H-1,2,3-triazole-4-carboxylate (13)

^{1}H NMR ($CDCl_3$, 600 MHz)

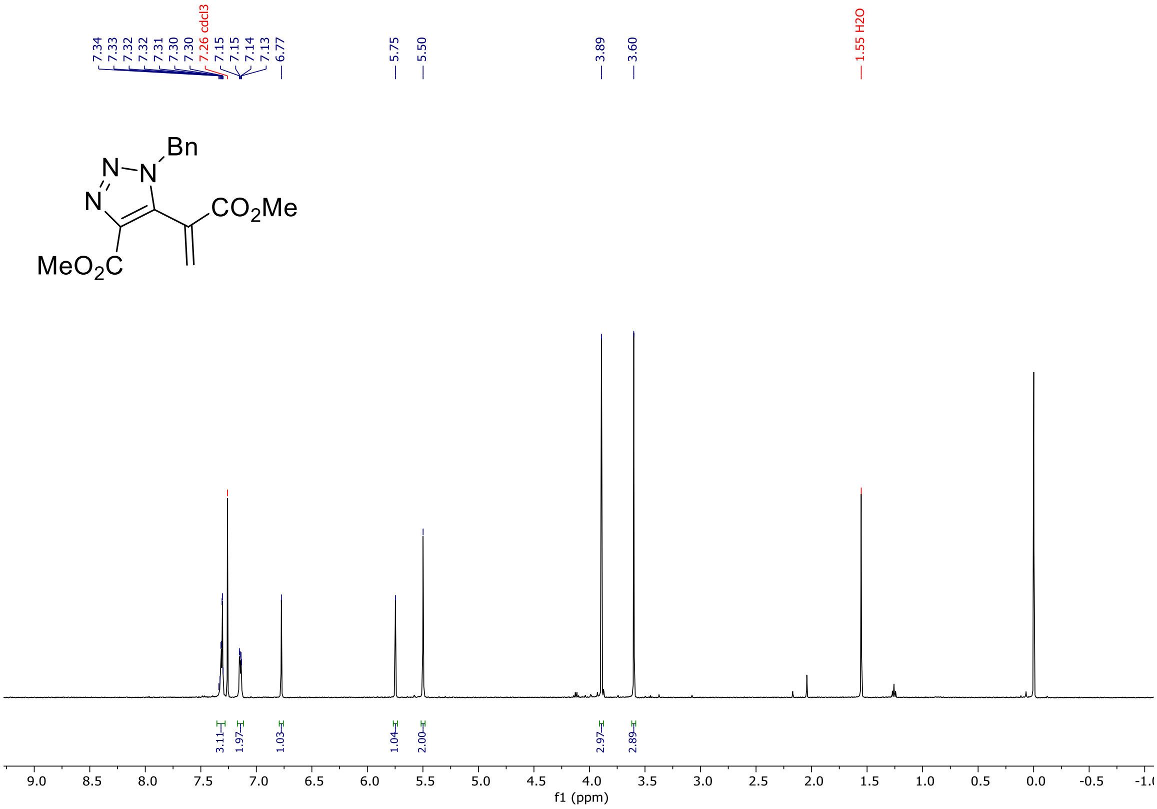

^{13}C NMR ($CDCl_3$, 151 MHz)

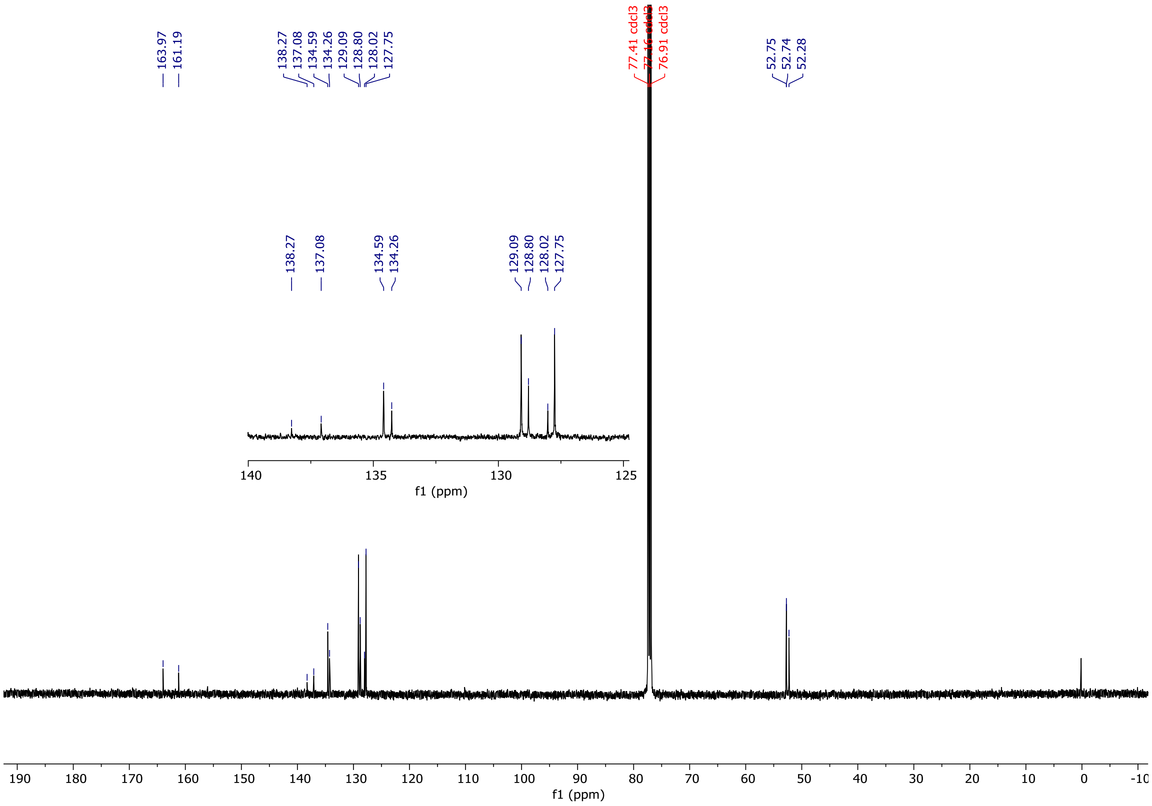

methyl 1-benzyl-5-(2-methoxy-2-oxo-1-(prop-1-en-2-yloxy)ethyl)-1H-1,2,3-triazole-4-carboxylate (14a)

^{1}H NMR ($CDCl_3$, 600 MHz)

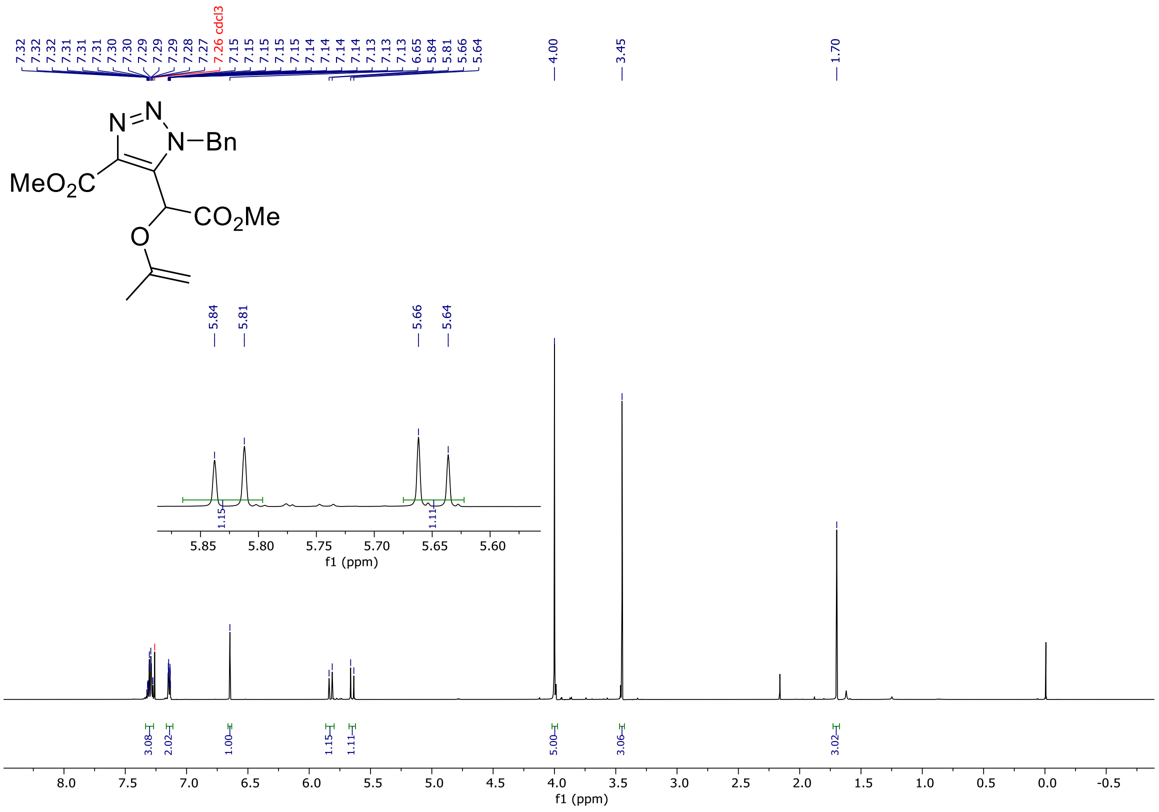

^{13}C NMR ($CDCl_3$, 151 MHz)

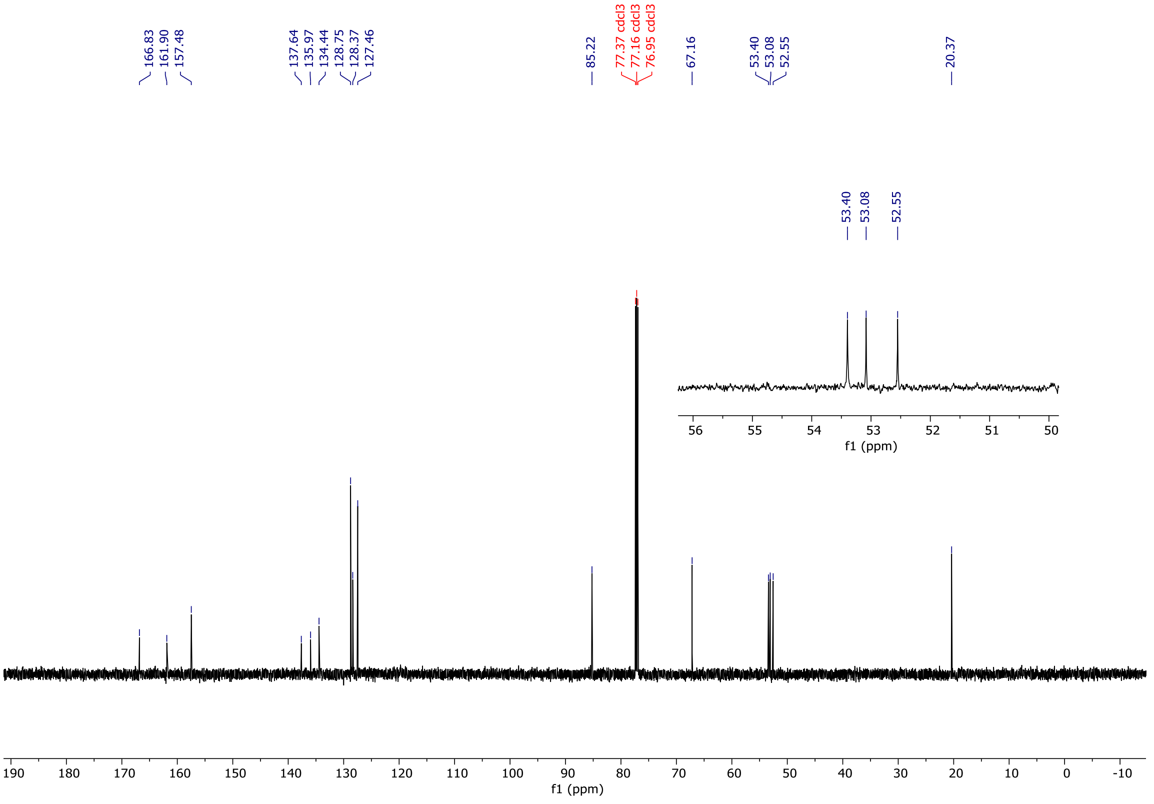

methyl 5-(2-methoxy-2-oxo-1-(prop-1-en-2-yloxy)ethyl)-1-phenyl-1H-1,2,3-triazole-4-carboxylate (14b)

^{1}H NMR ($CDCl_3$, 500 MHz)

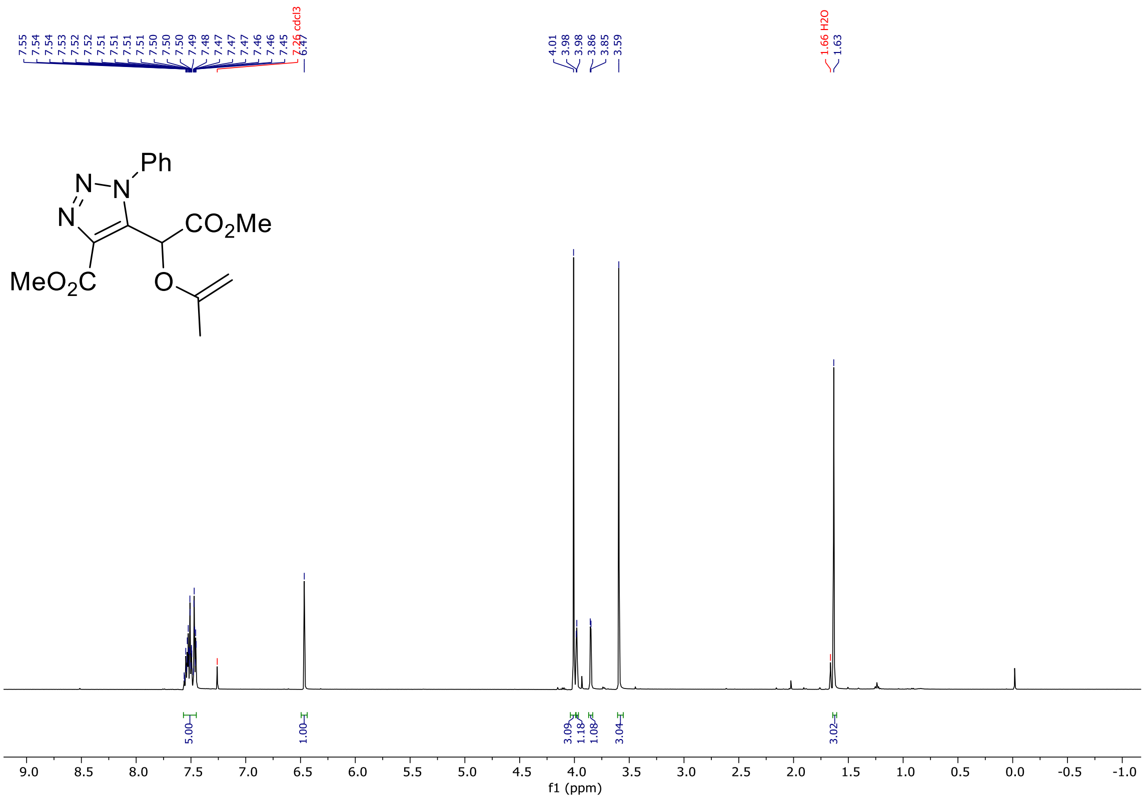

^{13}C NMR ($CDCl_3$, 126 MHz)

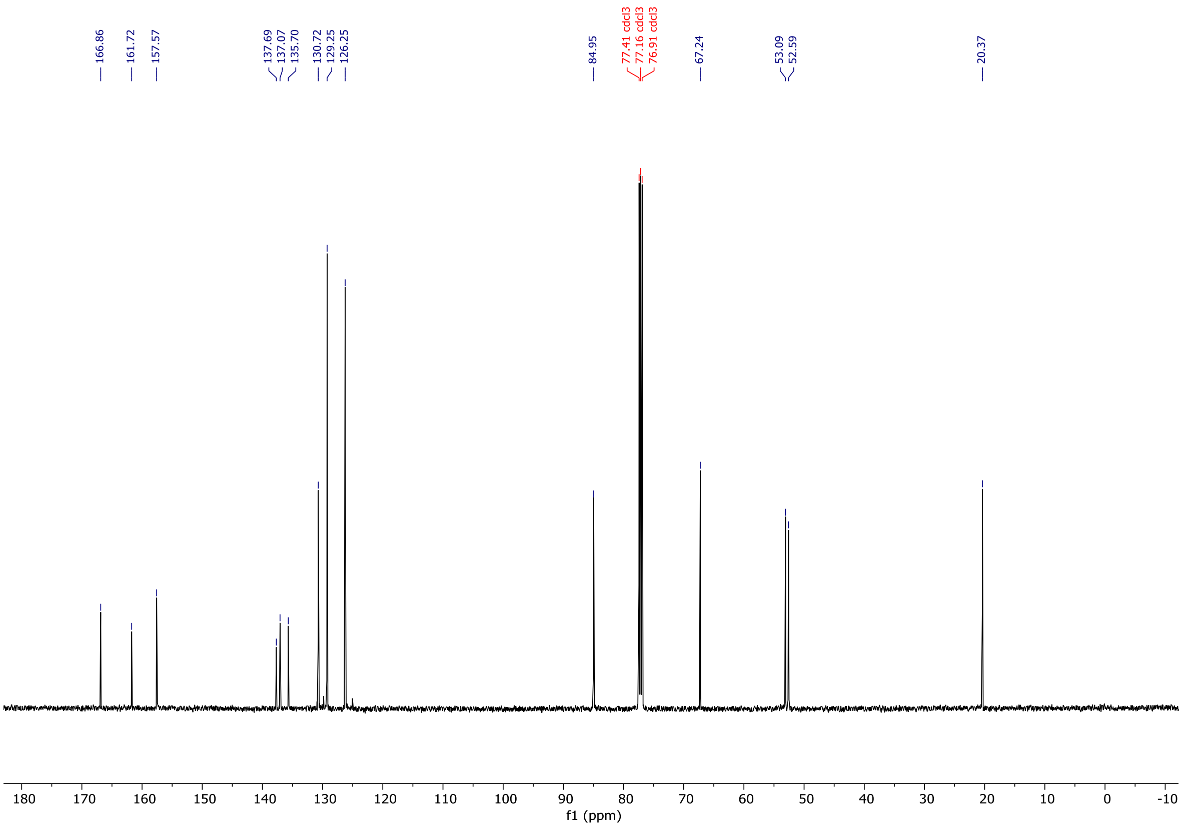

methyl 1-cyclopropyl-5-(2-methoxy-2-oxo-1-(prop-1-en-2-yloxy)ethyl)-1H-1,2,3-triazole-4-carboxylate (14c)

^{1}H NMR ($CDCl_3$, 600 MHz)

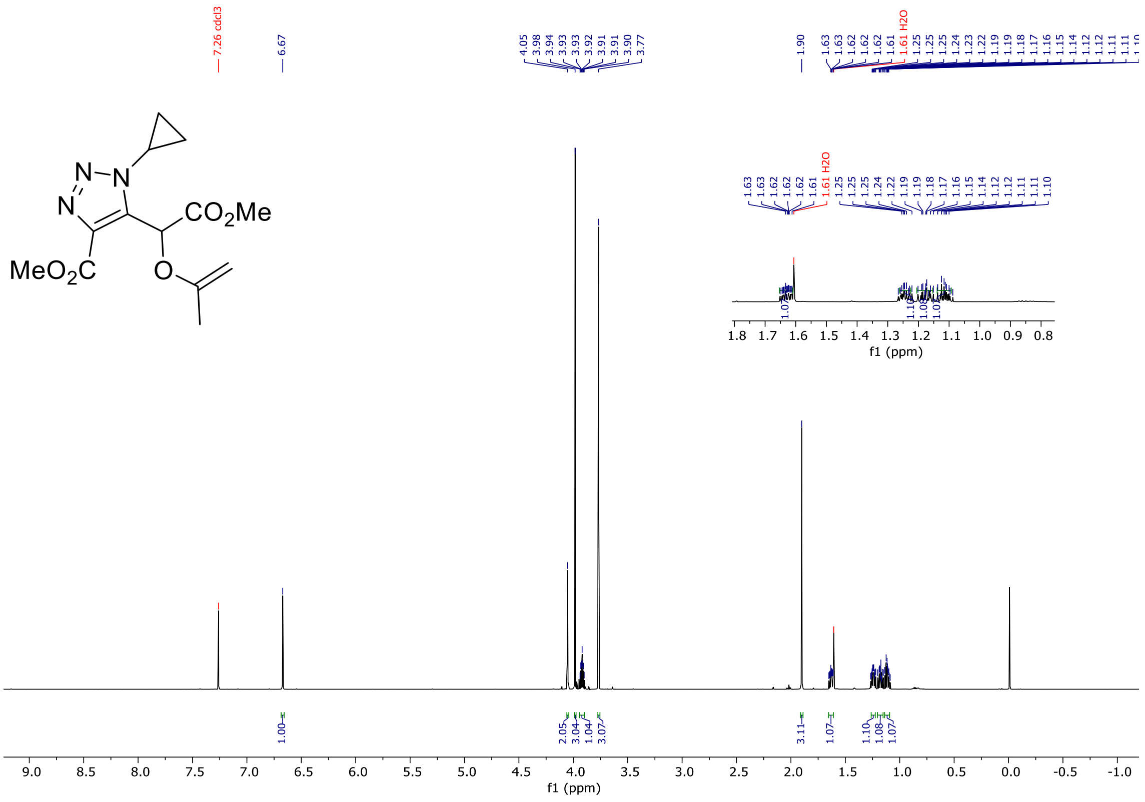

^{13}C NMR ($CDCl_3$, 151 MHz)

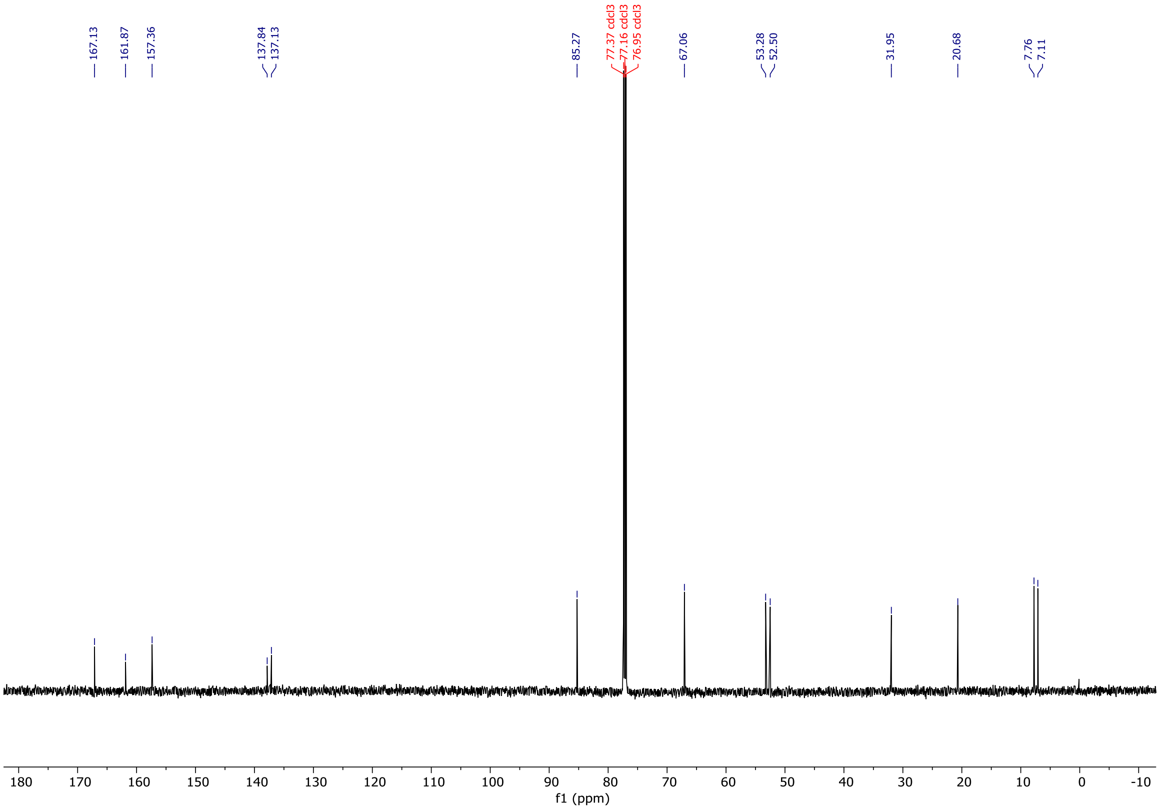

methyl 5-(2-methoxy-2-oxo-1-(prop-1-en-2-yloxy)ethyl)-1-propyl-1H-1,2,3-triazole-4-carboxylate (14d)

^{1}H NMR ($CDCl_3$, 600 MHz)

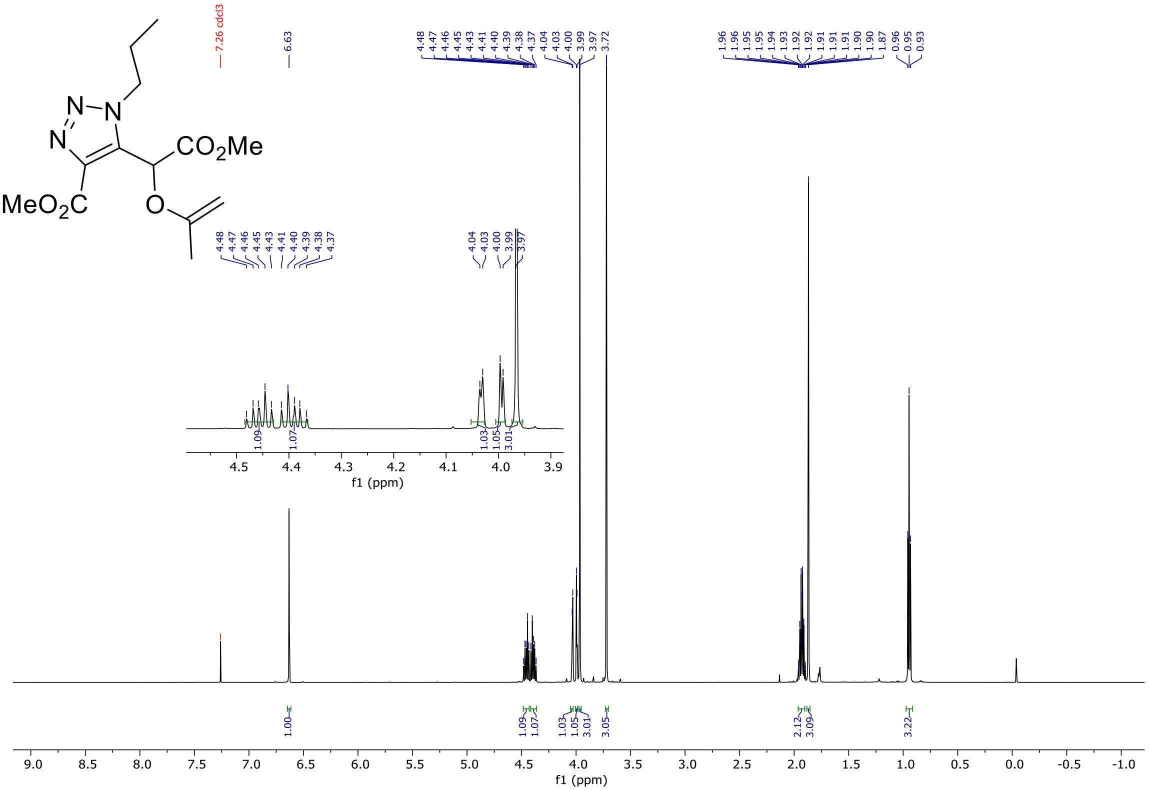

^{13}C NMR ($CDCl_3$, 151 MHz)

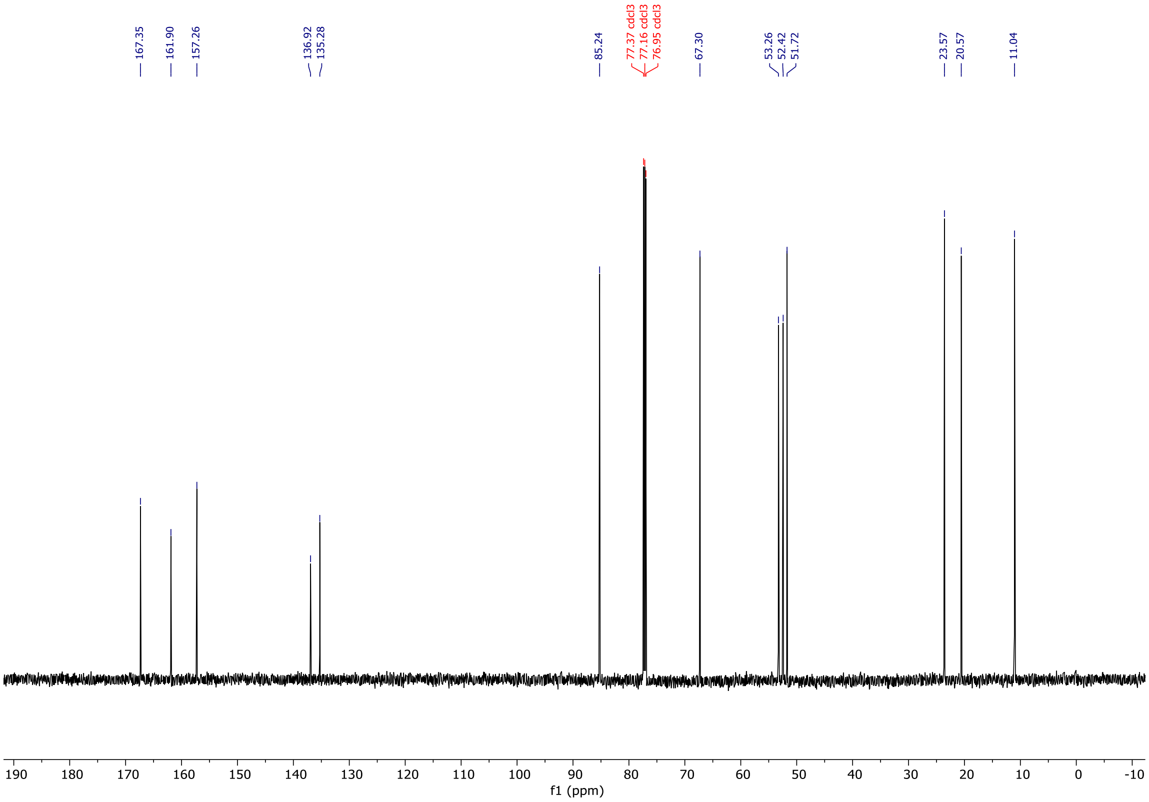

methyl 1-benzyl-5-(1,2-dimethoxy-2-oxoethyl)-1H-1,2,3-triazole-4-carboxylate (15a)

^{1}H NMR ($CDCl_3$, 600 MHz)

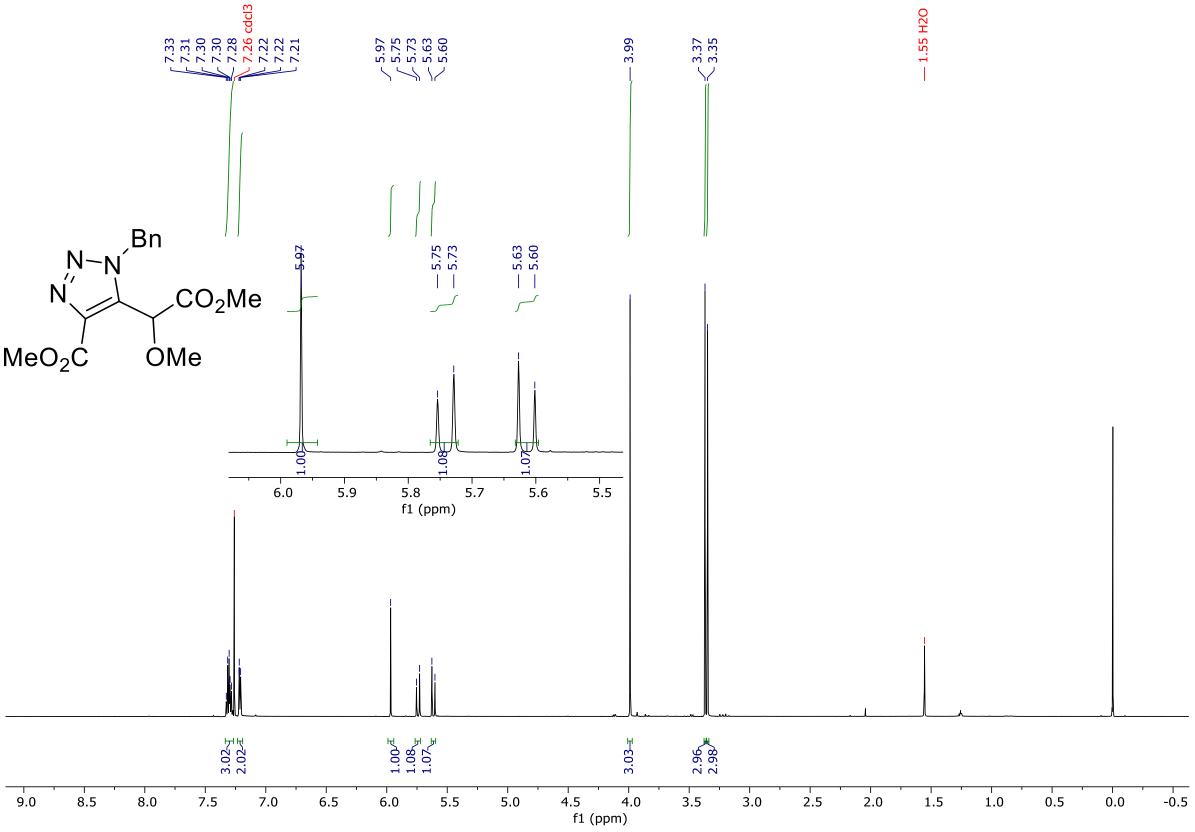

^{13}C NMR ($CDCl_3$, 151 MHz)

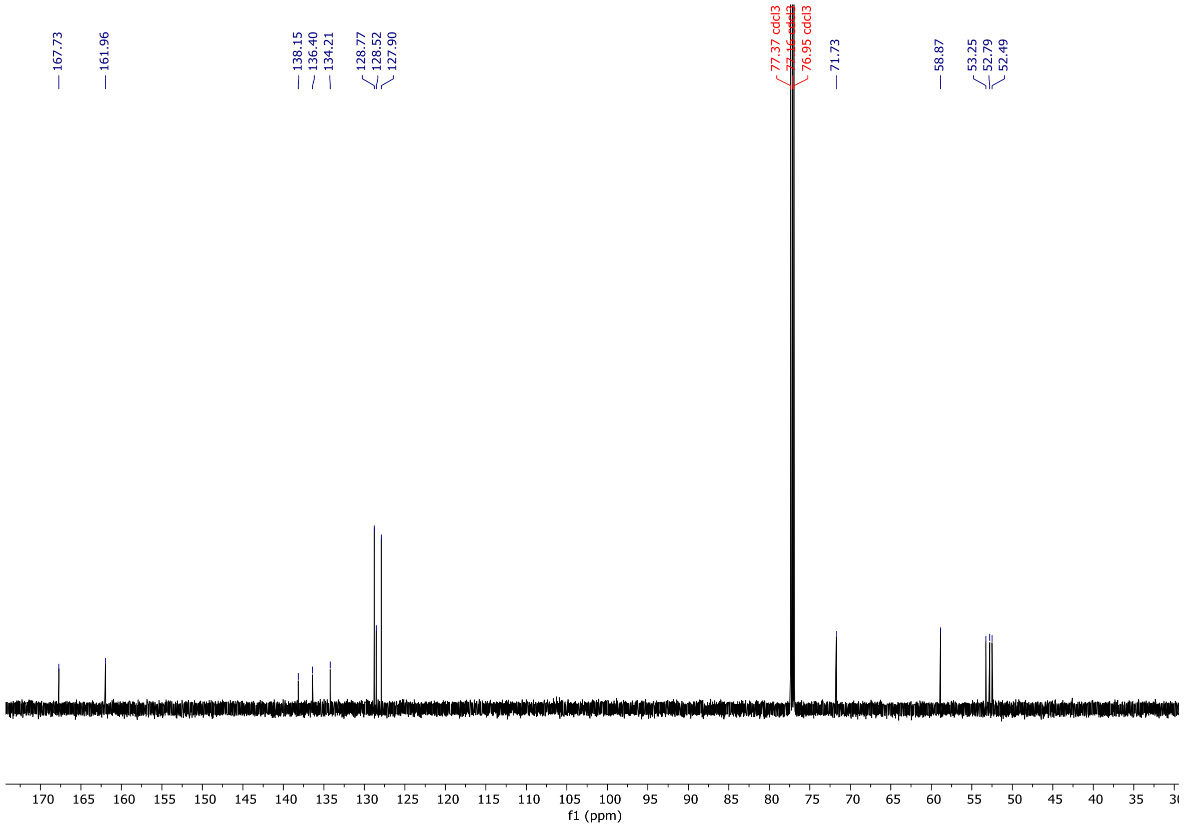

methyl 5-(1,2-dimethoxy-2-oxoethyl)-1-phenyl-1H-1,2,3-triazole-4-carboxylate (15b)

^{1}H NMR ($CDCl_3$, 600 MHz)

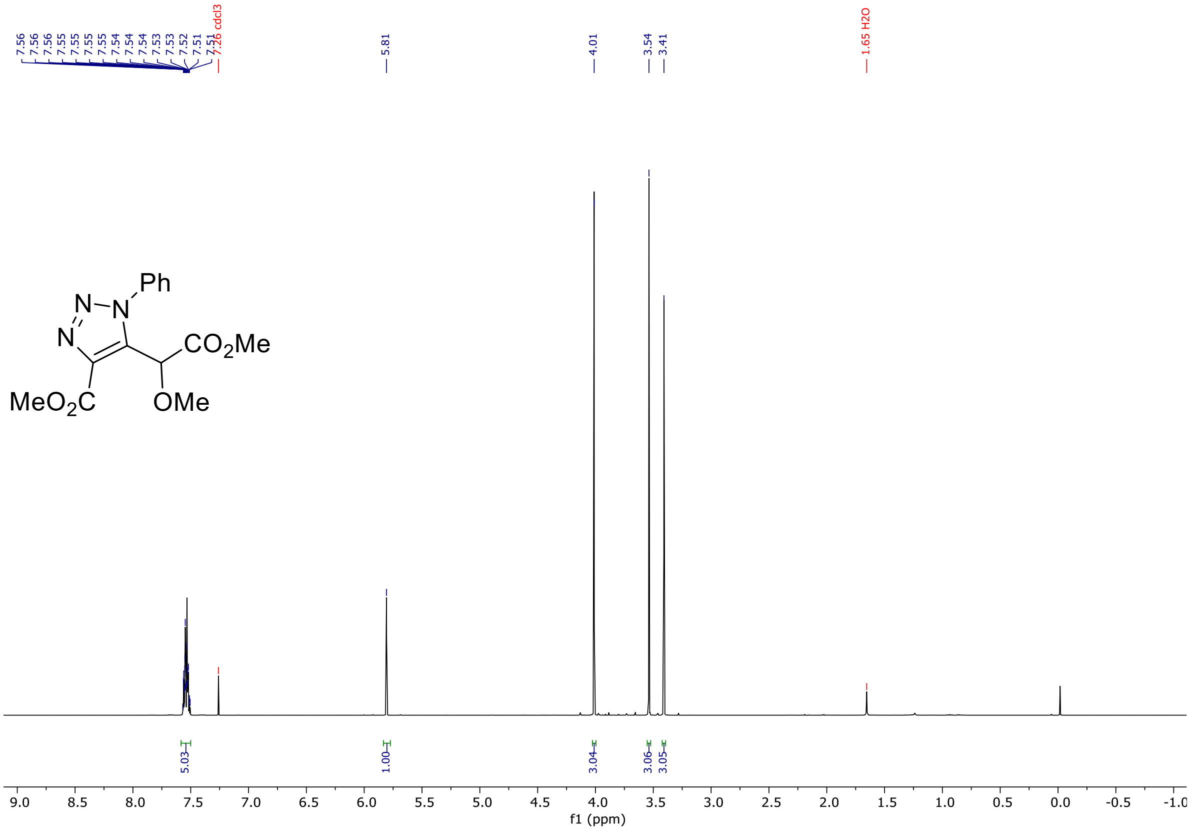

^{13}C NMR ($CDCl_3$, 151 MHz)

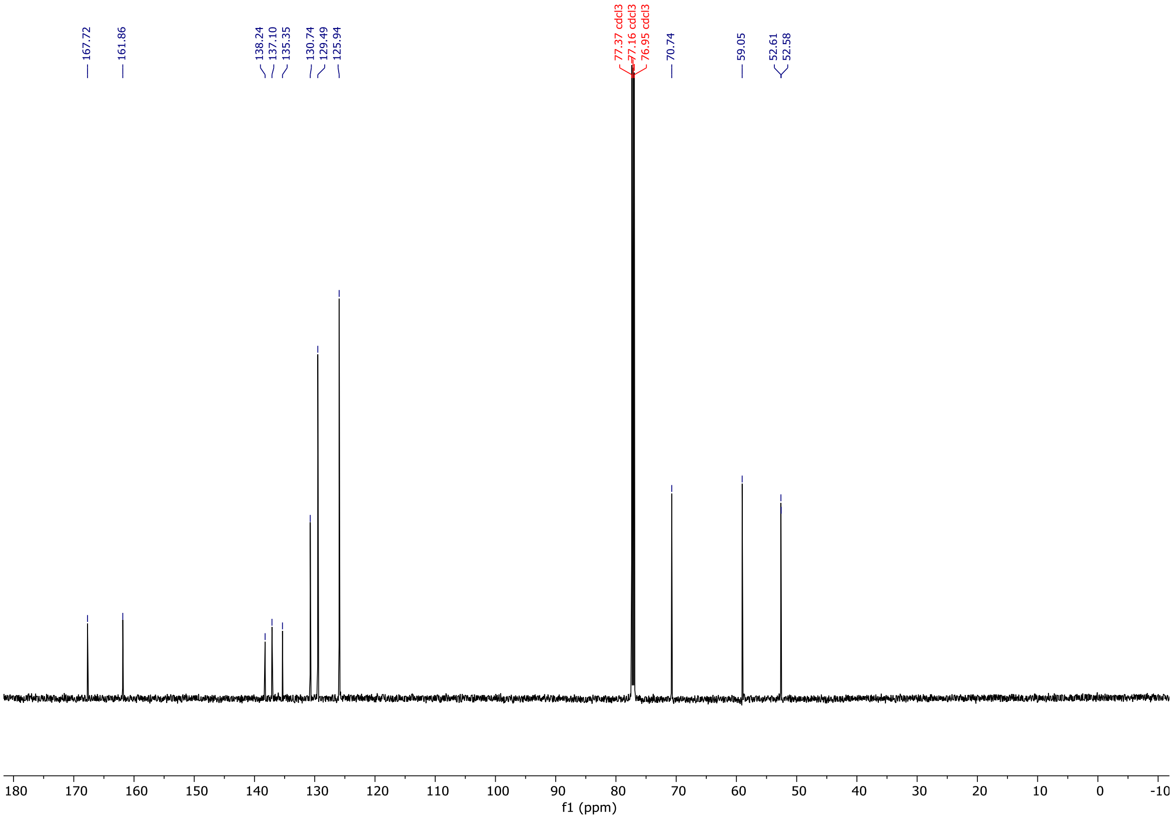

methyl 1-cyclopropyl-5-(1,2-dimethoxy-2-oxoethyl)-1H-1,2,3-triazole-4-carboxylate (15c)

^{1}H NMR ($CDCl_3$, 600 MHz)

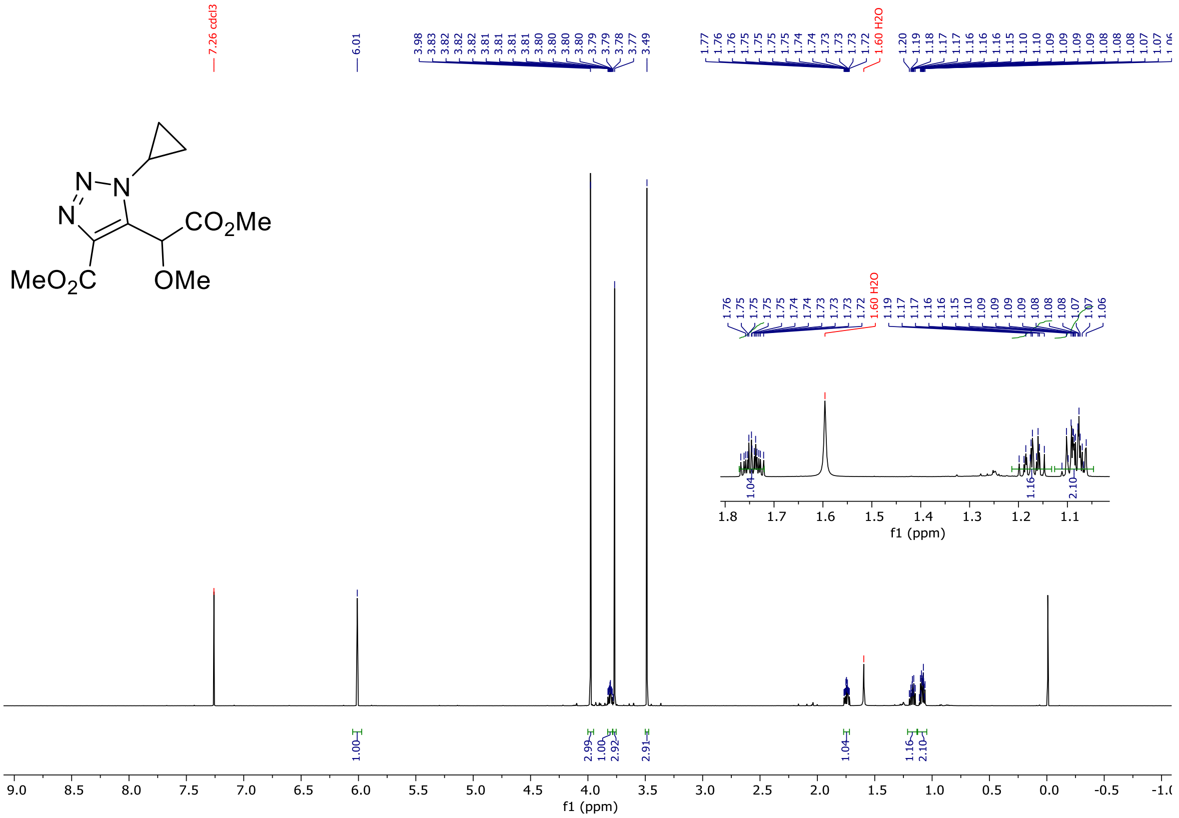

^{13}C NMR ($CDCl_3$, 151 MHz)

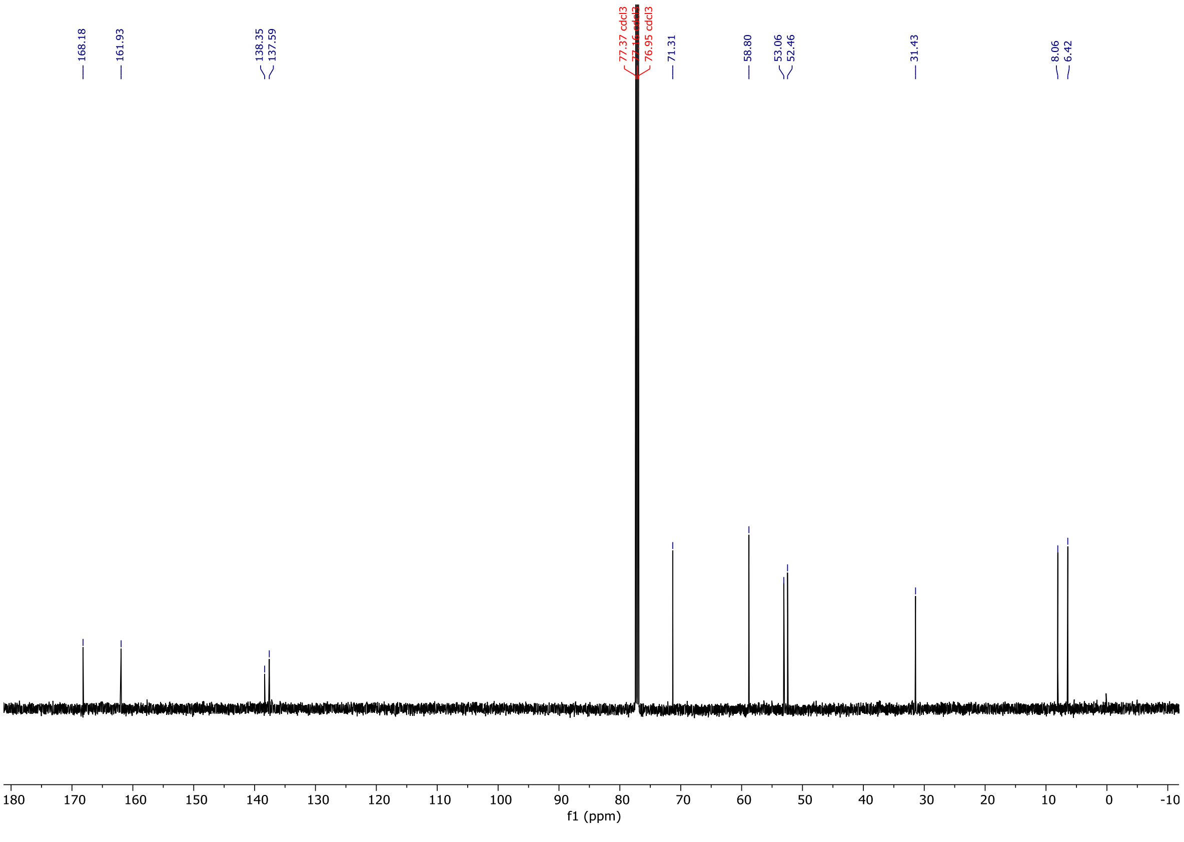

methyl 5-(1,2-dimethoxy-2-oxoethyl)-1-propyl-1H-1,2,3-triazole-4-carboxylate (15d)

^{1}H NMR ($CDCl_3$, 500 MHz)

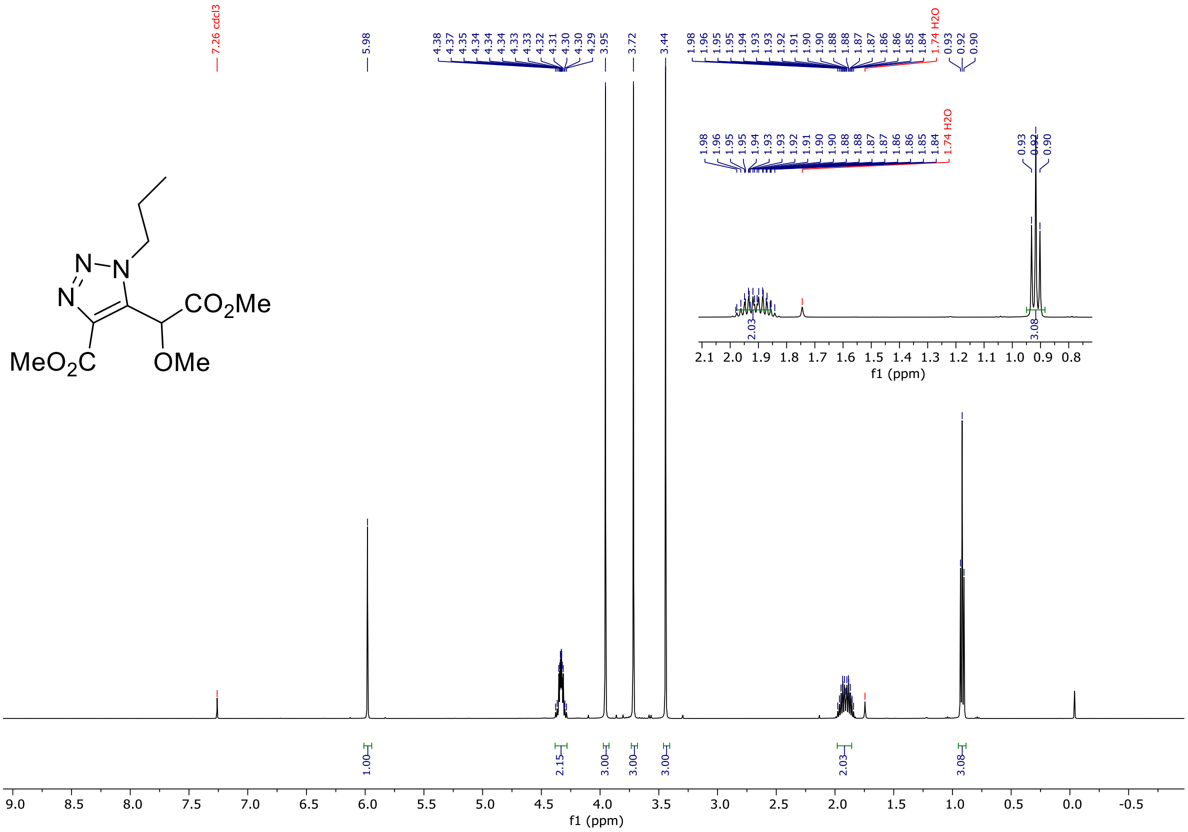

^{13}C NMR ($CDCl_3$, 151 MHz)

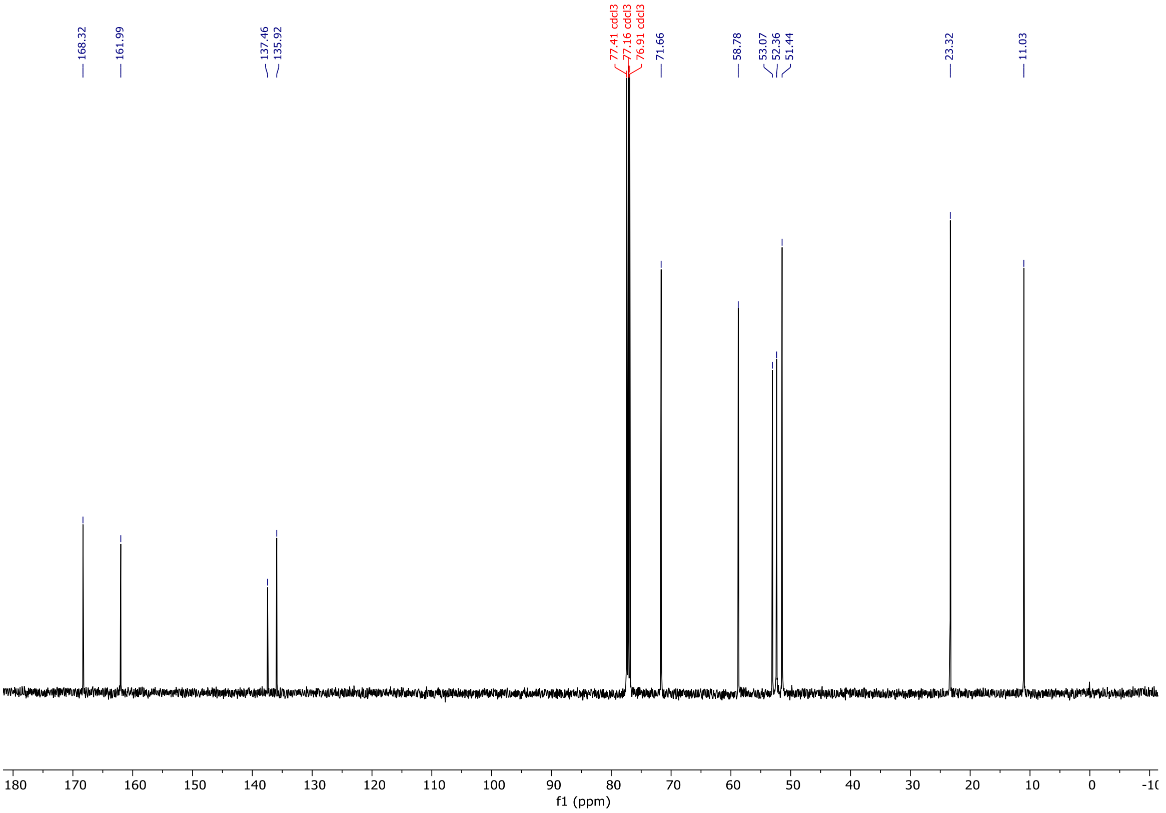

4-ethyl 1-methyl 2-(1-benzyl-4-(methoxycarbonyl)-1H-1,2,3-triazol-5-yl)succinate (16)

^{1}H NMR (CDCl$_3$, 500 MHz)

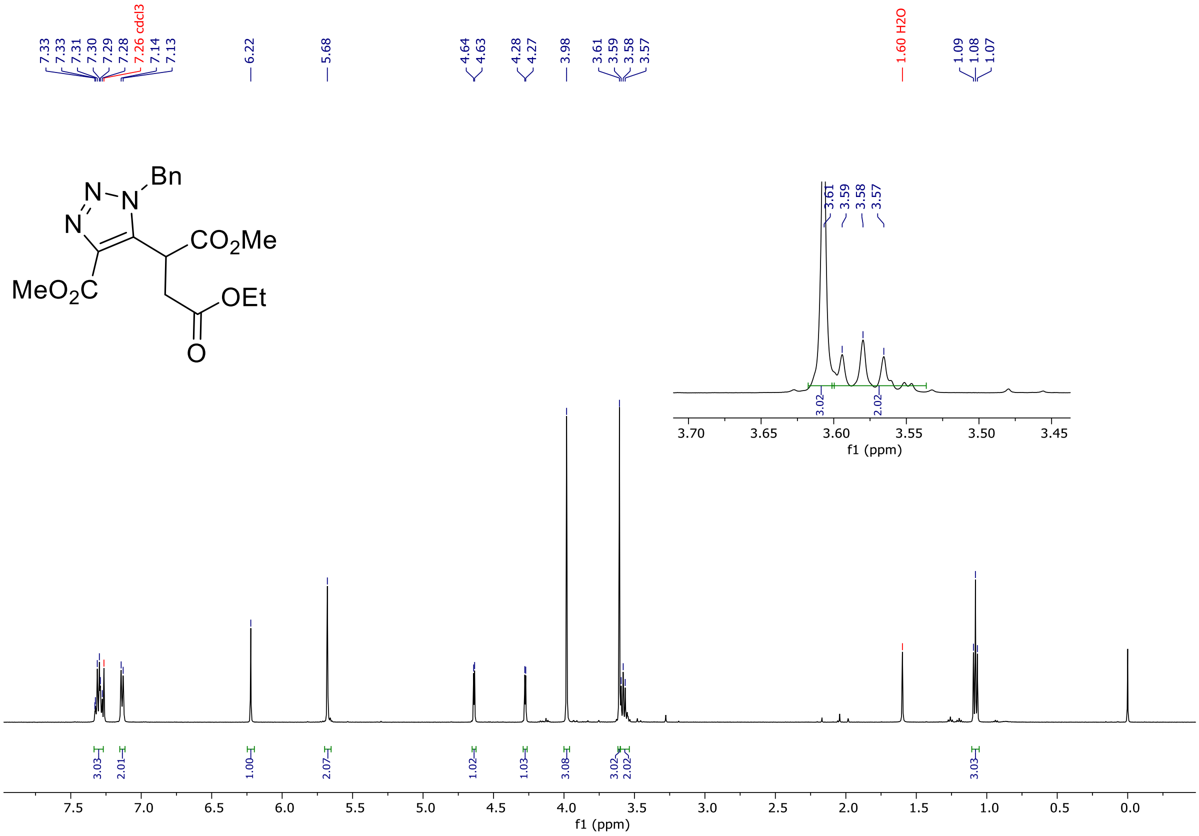

^{13}C NMR (CDCl$_3$, 126 MHz)

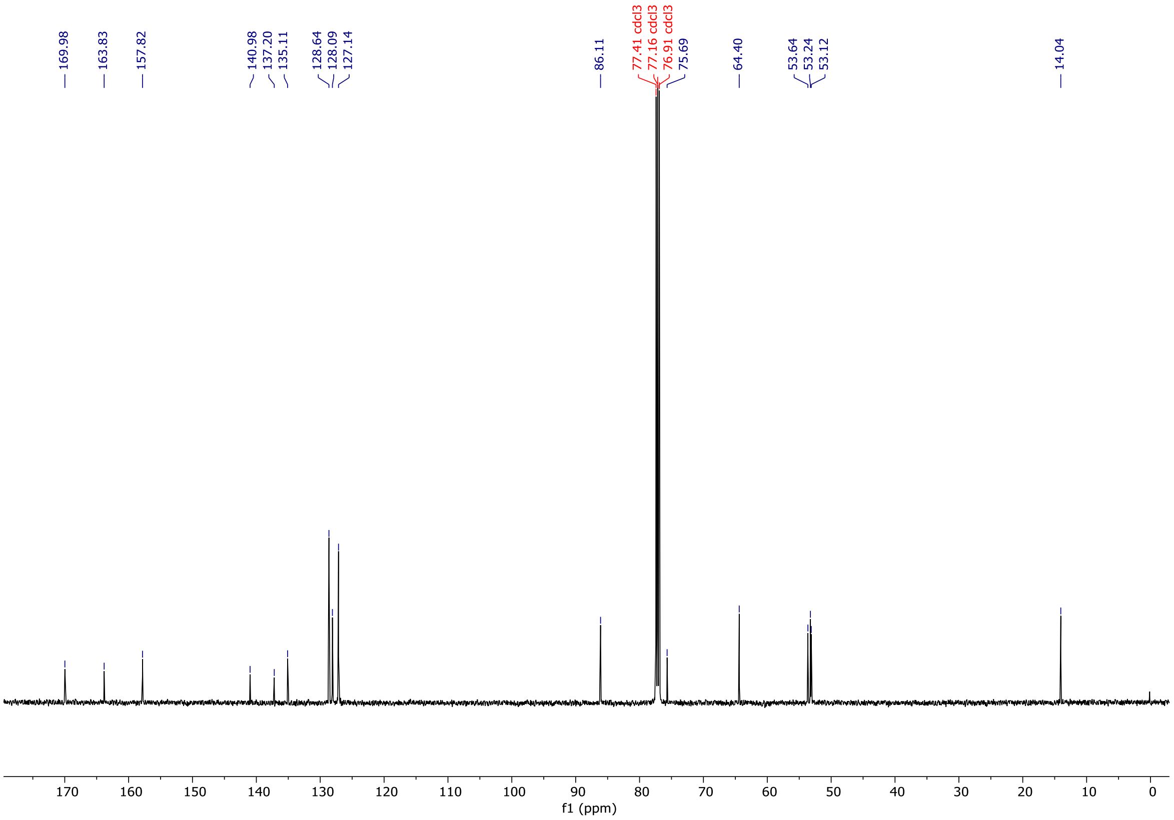

methyl 1-benzyl-5-(5-methoxy-2-methyloxazol-4-yl)-1H-1,2,3-triazole-4-carboxylate (17a)

^{1}H NMR ($CDCl_3$, 600 MHz)

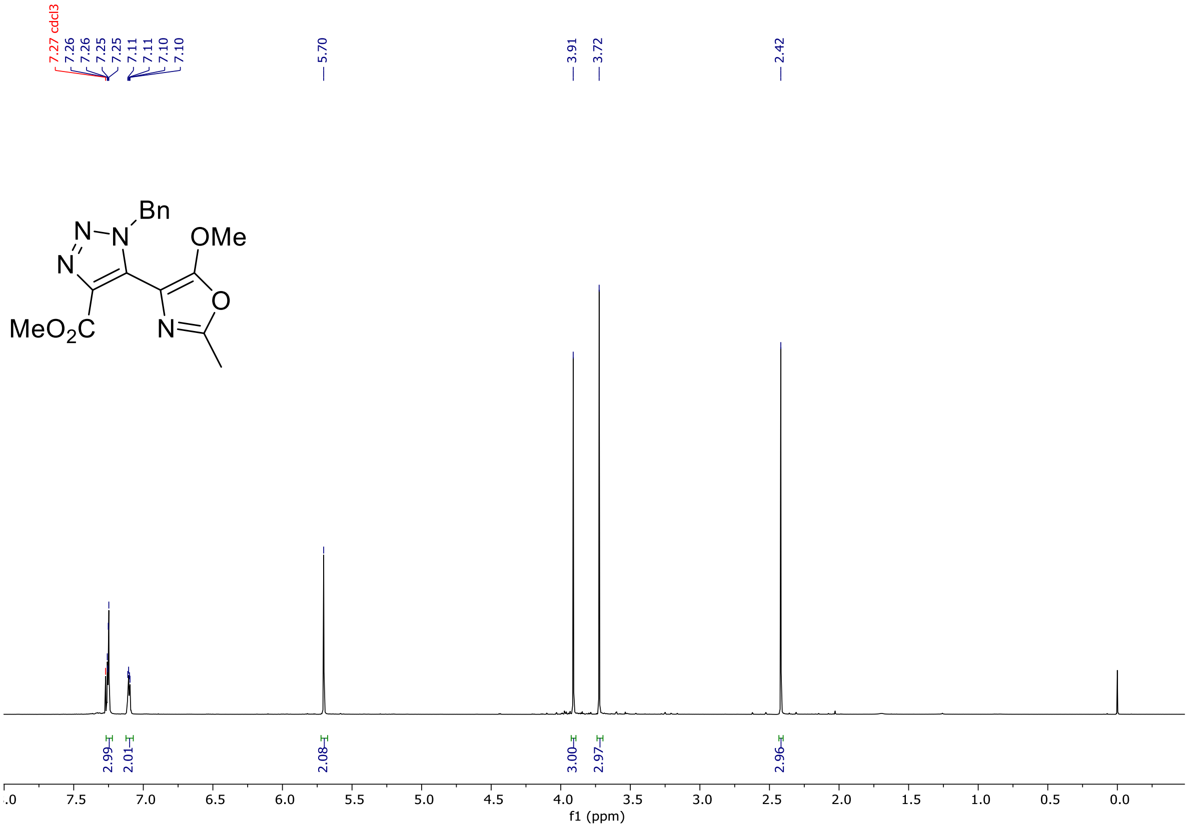

^{13}C NMR ($CDCl_3$, 151 MHz)

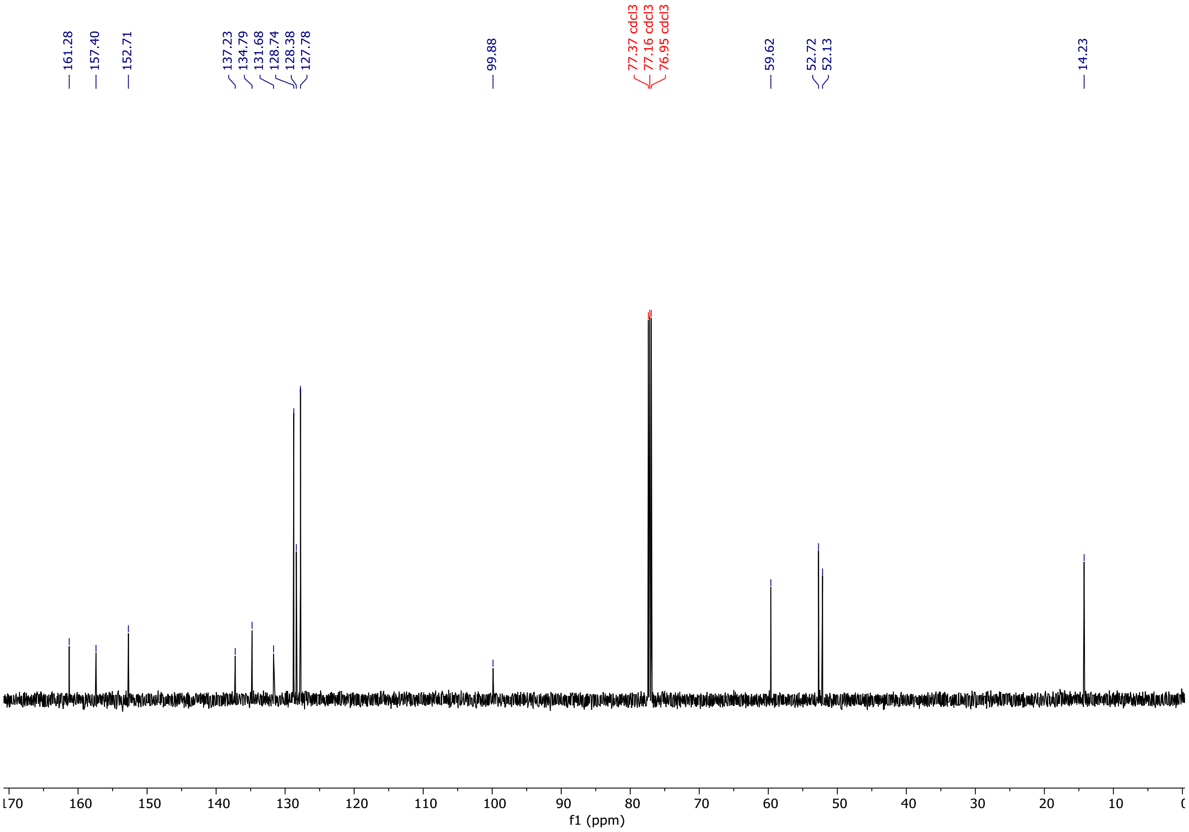

methyl 5-(5-methoxy-2-methyloxazol-4-yl)-1-phenyl-1H-1,2,3-triazole-4-carboxylate (17b)

^{1}H NMR ($CDCl_3$, 600 MHz)

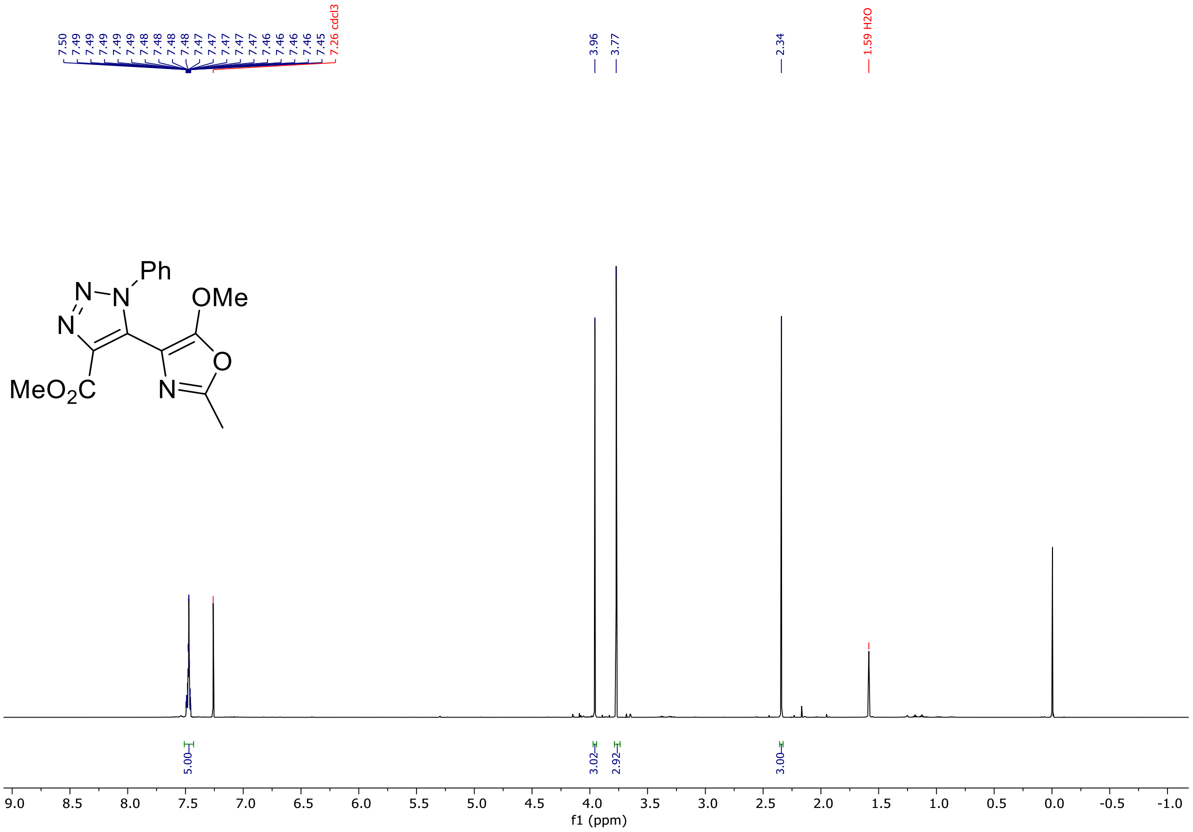

^{13}C NMR ($CDCl_3$, 151 MHz)

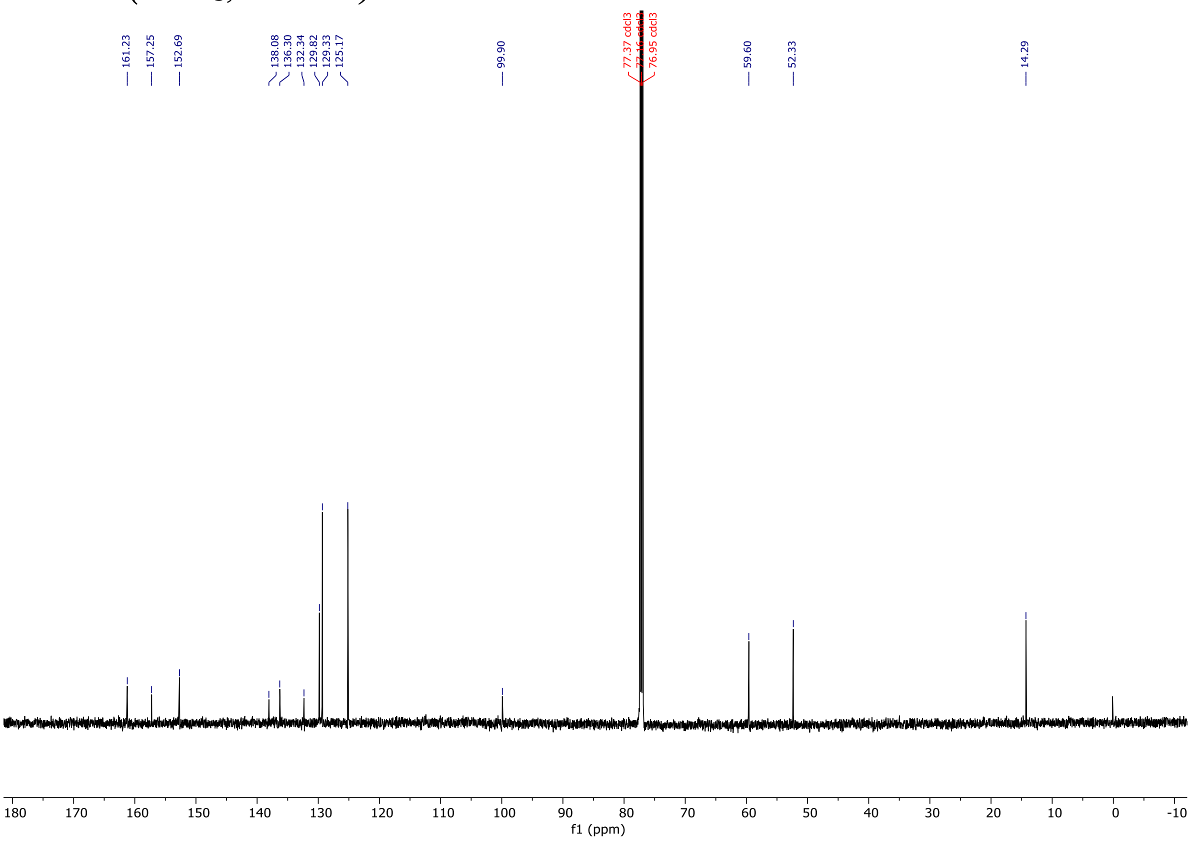

methyl 1-cyclopropyl-5-(5-methoxy-2-methyloxazol-4-yl)-1H-1,2,3-triazole-4-carboxylate (17c)

^{1}H NMR (CDCl$_3$, 600 MHz)

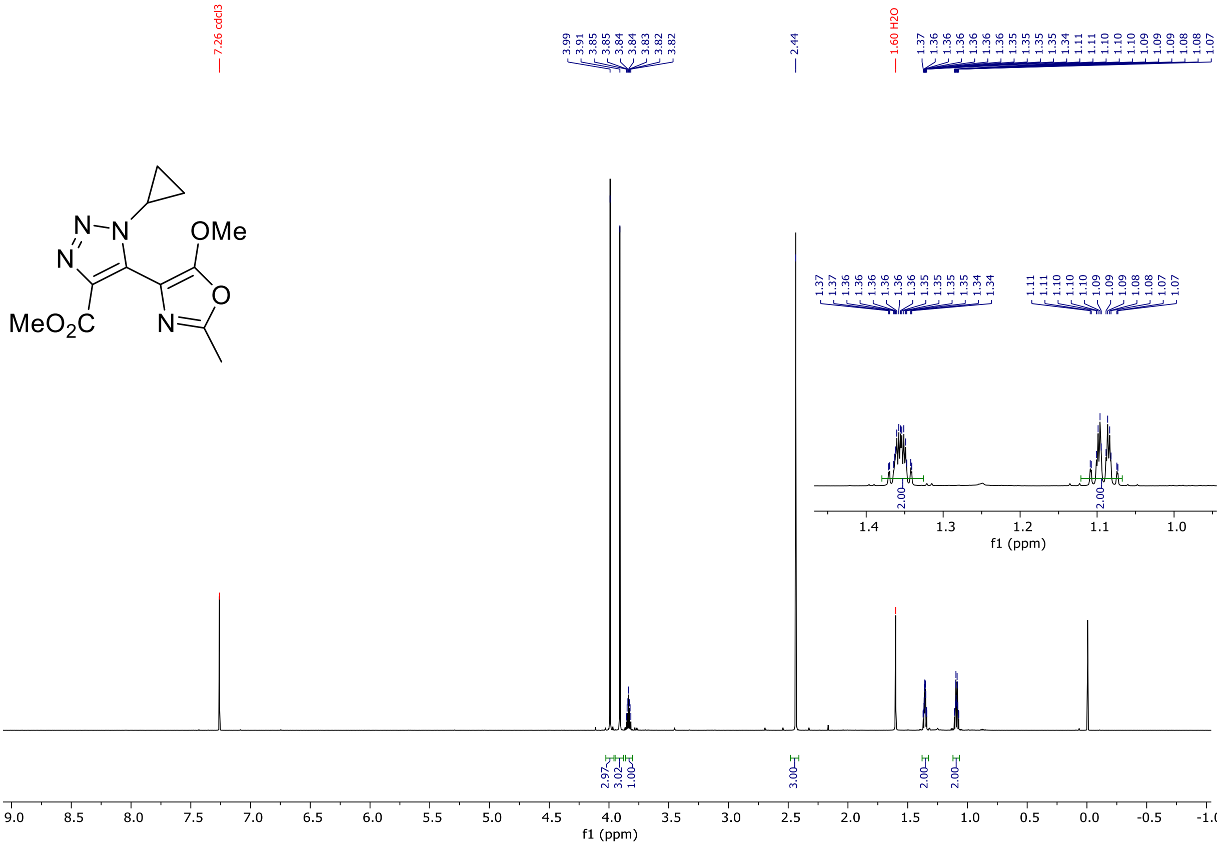

^{13}C NMR (CDCl$_3$, 151 MHz)

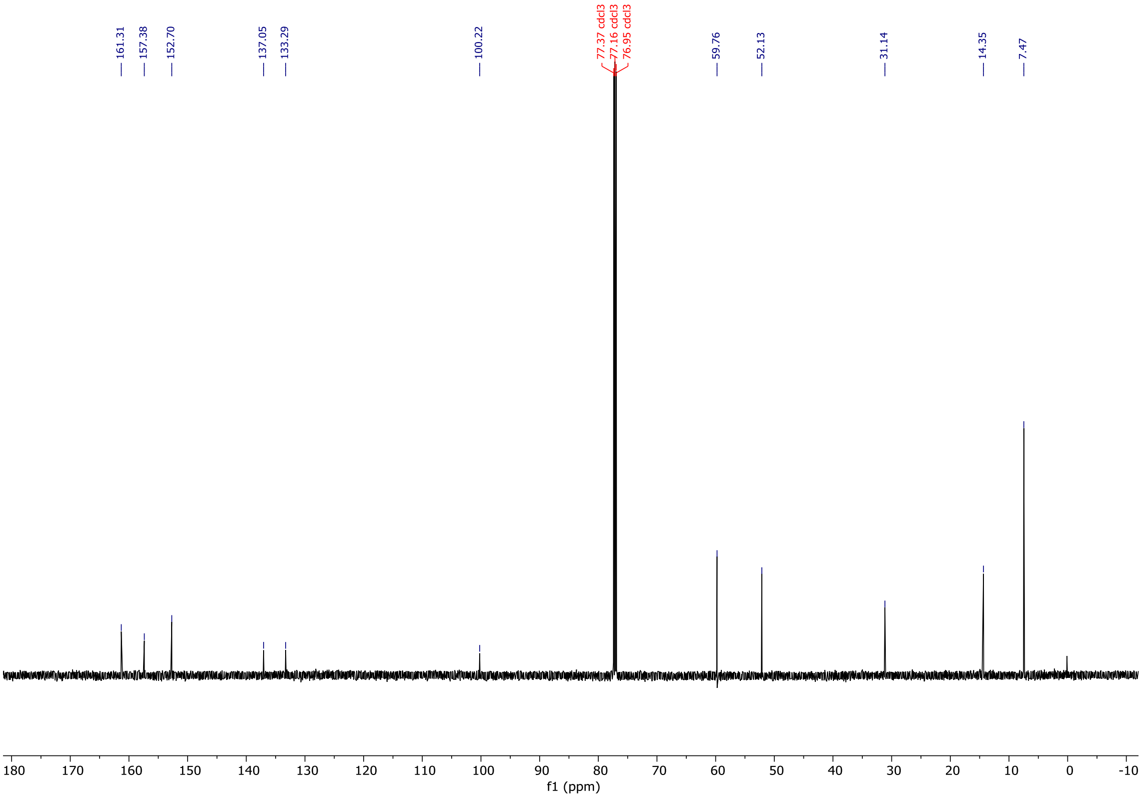

methyl 5-(5-methoxy-2-methyloxazol-4-yl)-1-propyl-1H-1,2,3-triazole-4-carboxylate (17d)

^{1}H NMR (CDCl$_3$, 600 MHz)

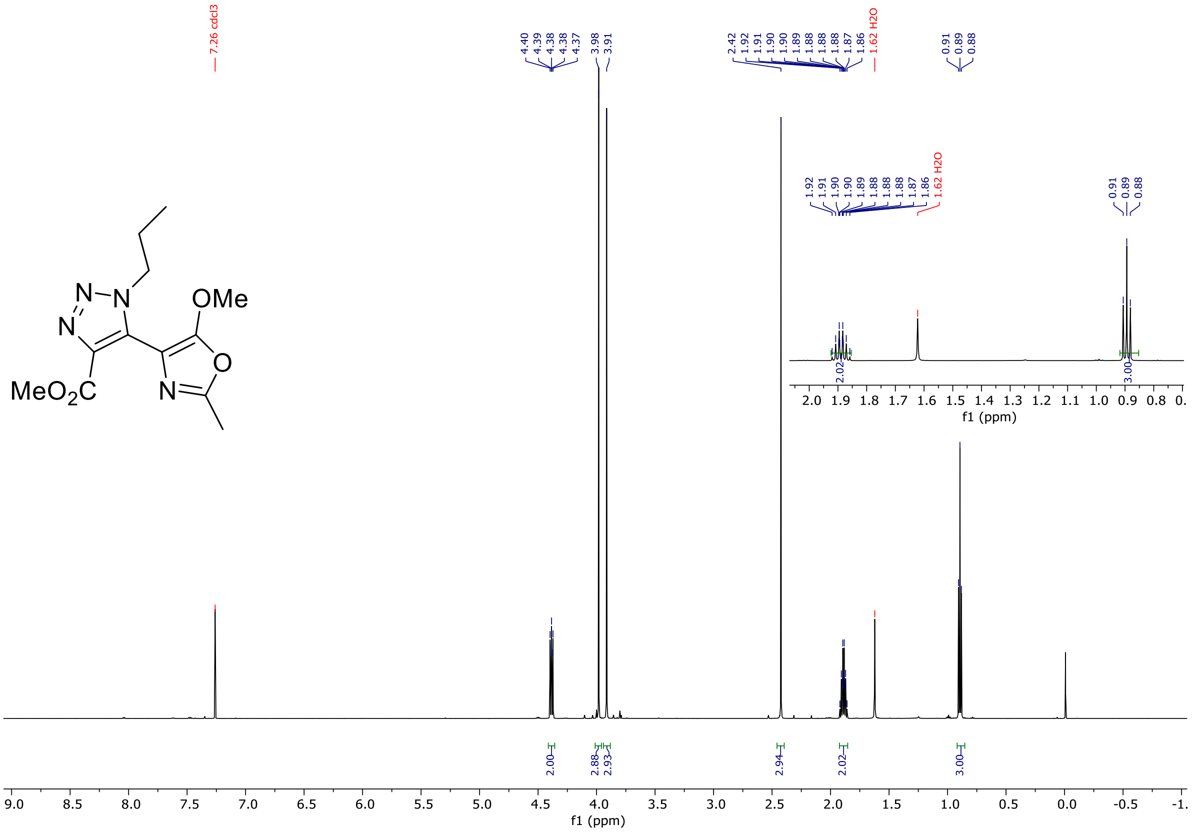

^{13}C NMR (CDCl$_3$, 151 MHz)

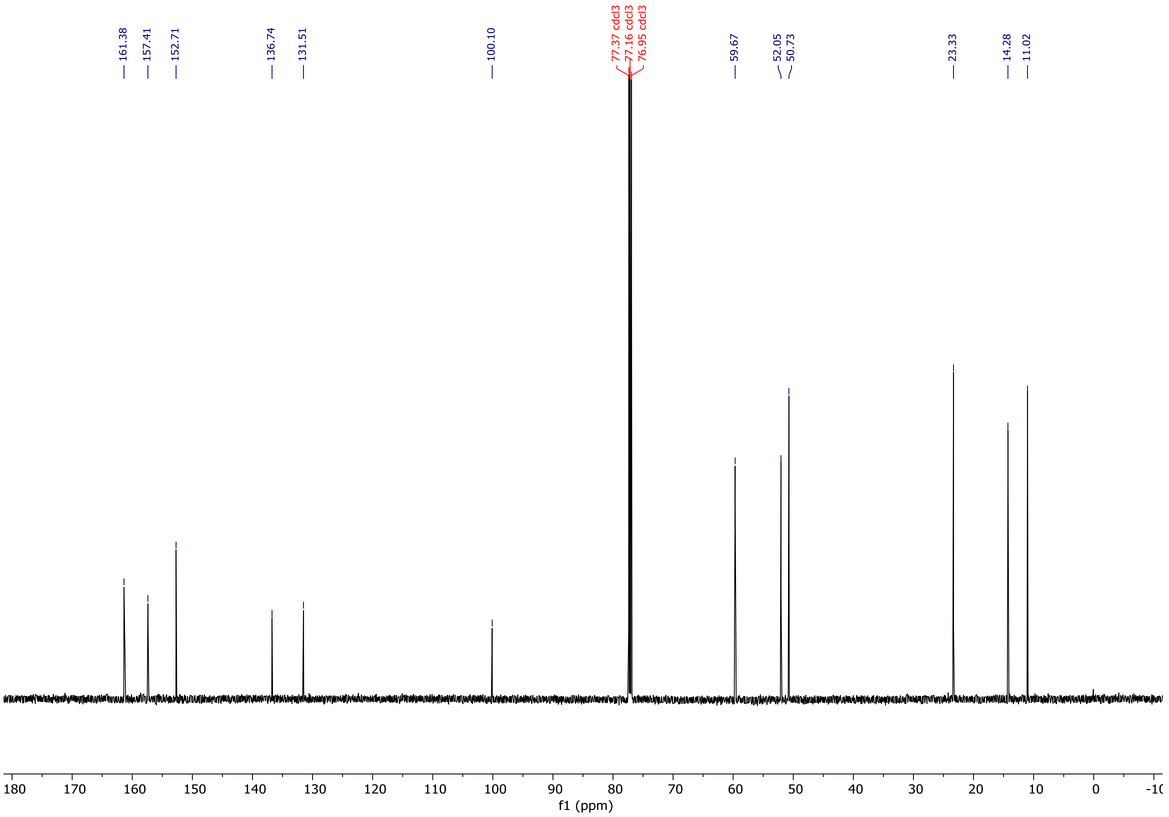

methyl 1-benzyl-5-(7-(methoxycarbonyl)bicyclo[4.1.0]hepta-2,4-dien-7-yl)-1H-1,2,3-triazole-4-carboxylate (18)

^{1}H NMR ($CDCl_3$, 500 MHz)

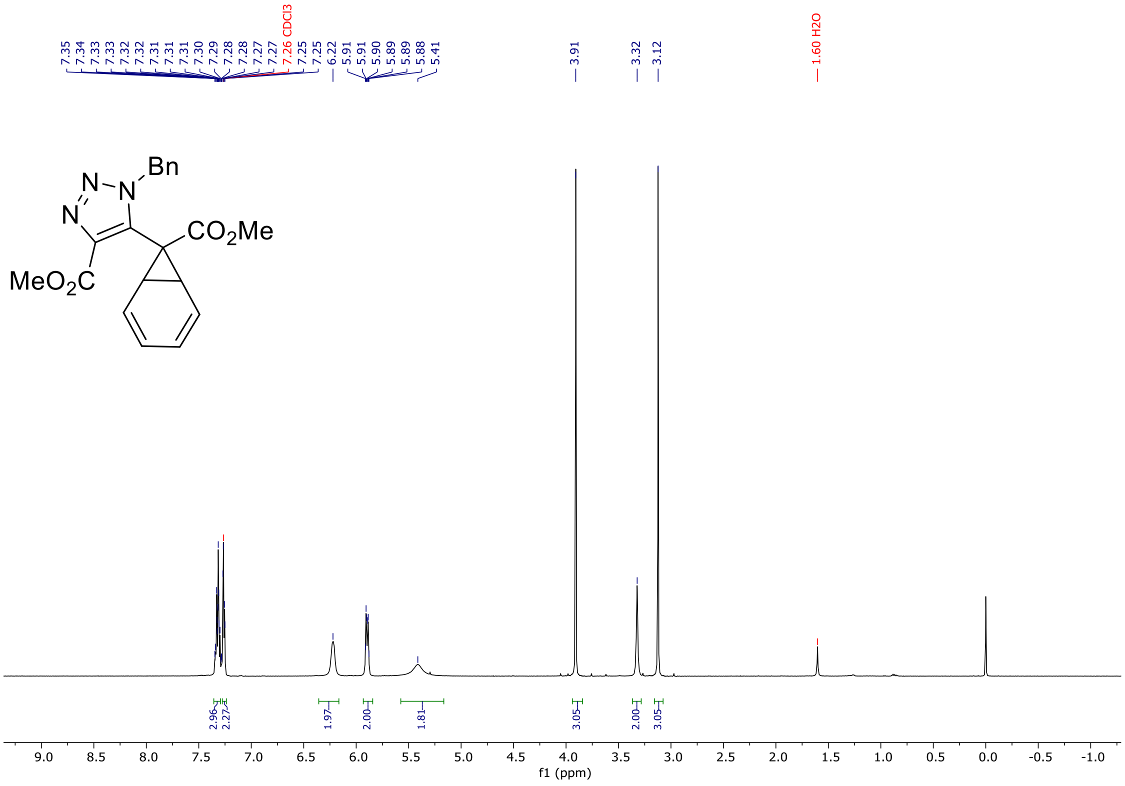

^{13}C NMR ($CDCl_3$, 126 MHz)

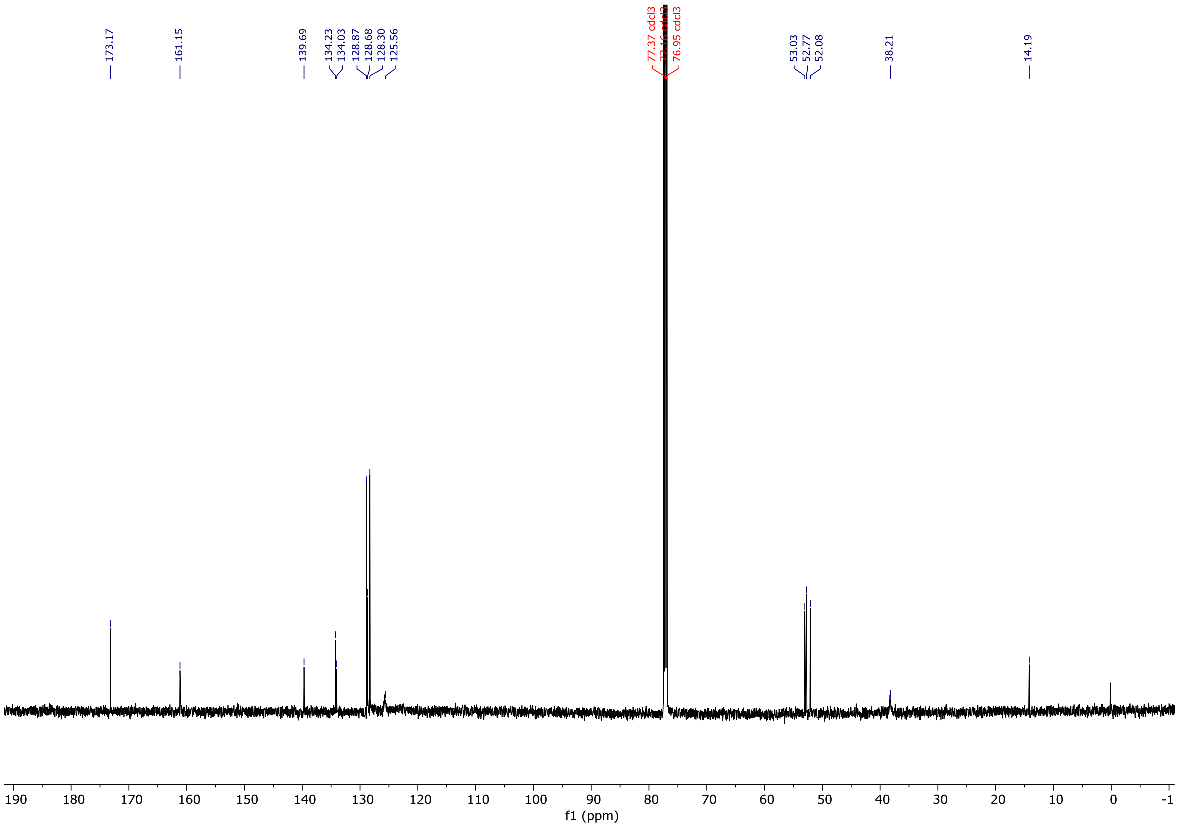

methyl 1-benzyl-5-(2-methoxy-2-oxo-1-(tetrahydrofuran-2-yl)ethyl)-1H-1,2,3-triazole-4-carboxylate (19)

^{1}H NMR (CDCl$_3$, 500 MHz)

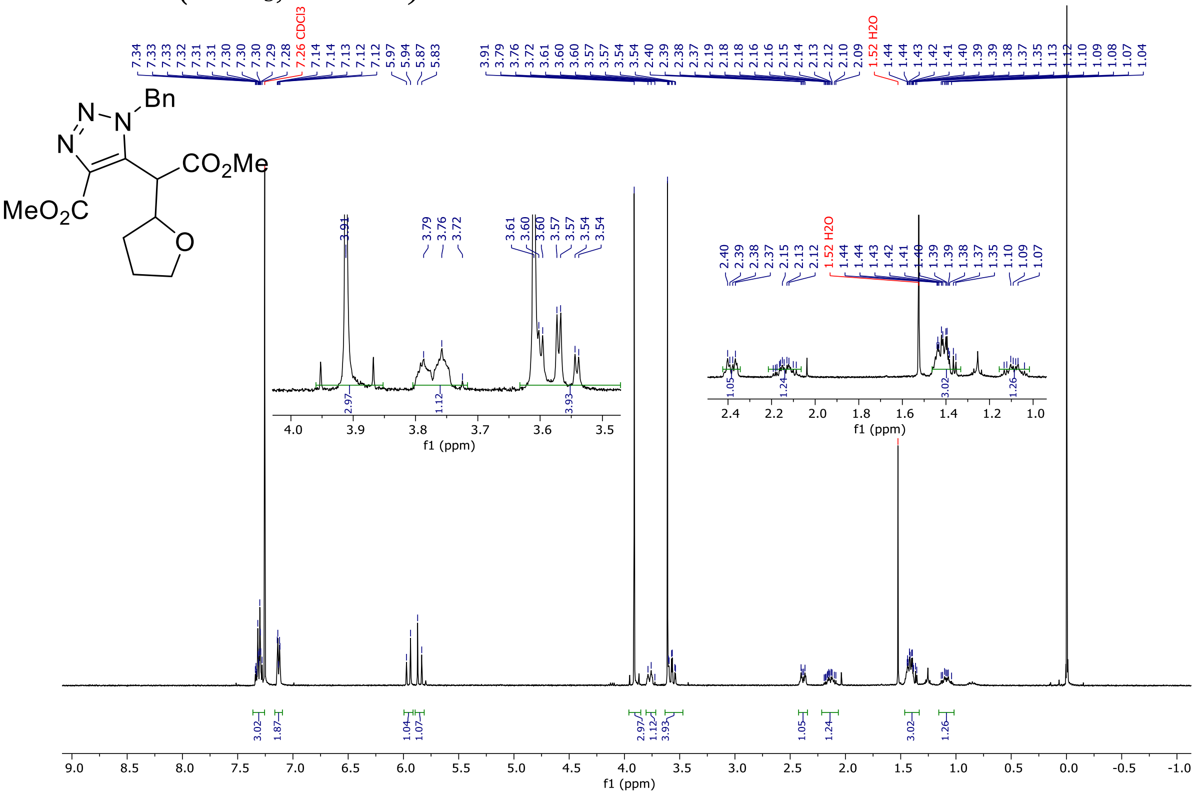

^{13}C NMR (CDCl$_3$, 126 MHz)

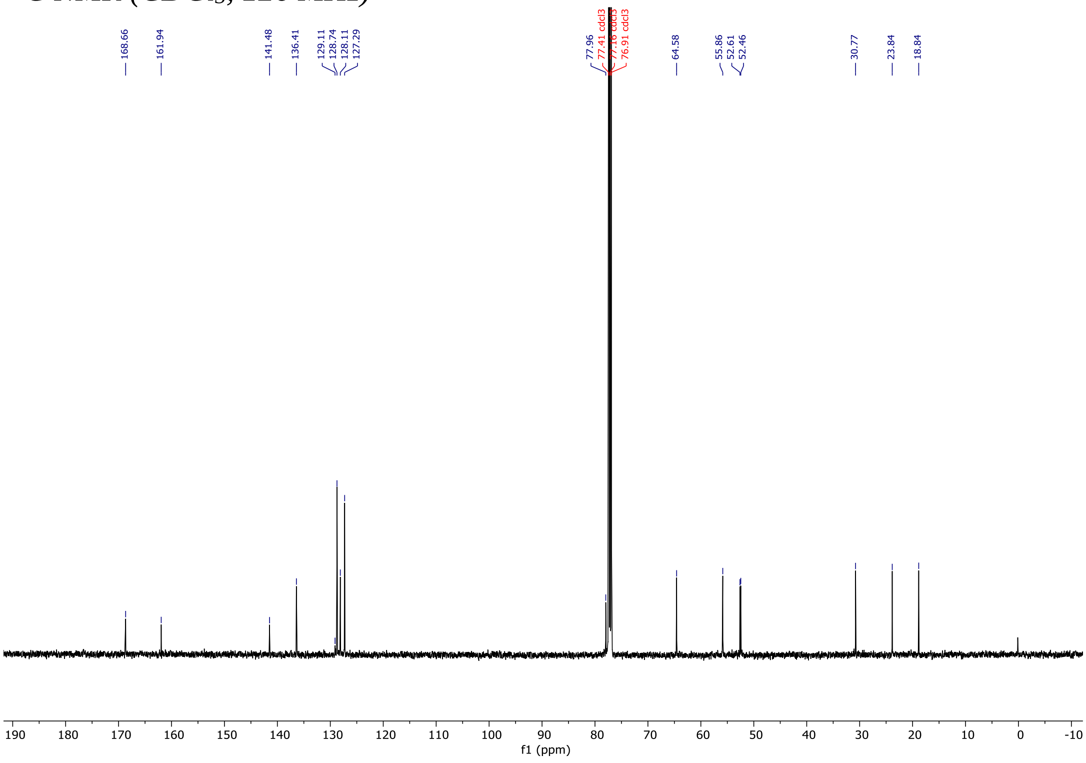

methyl 1-benzyl-5-(1-(but-3-en-1-yloxy)-2-methoxy-2-oxoethyl)-1H-1,2,3-triazole-4-carboxylate (20)

^{1}H NMR ($CDCl_3$, 500 MHz)

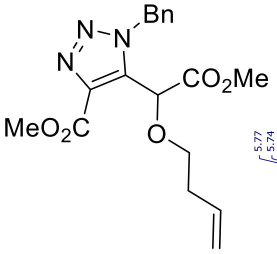

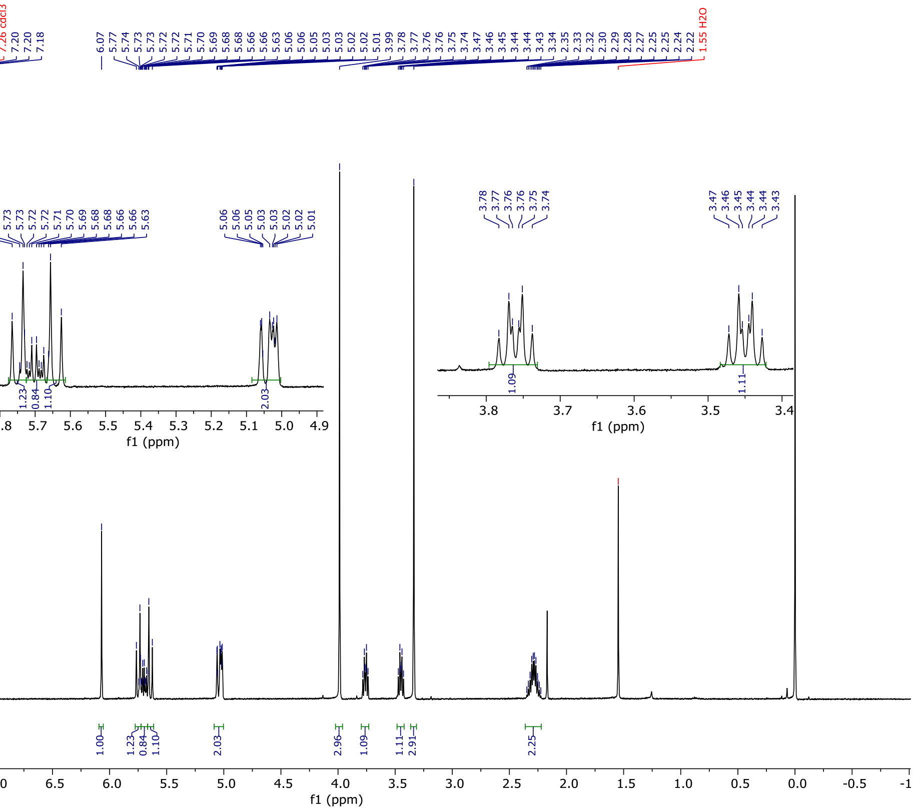

^{13}C NMR ($CDCl_3$, 126 MHz)

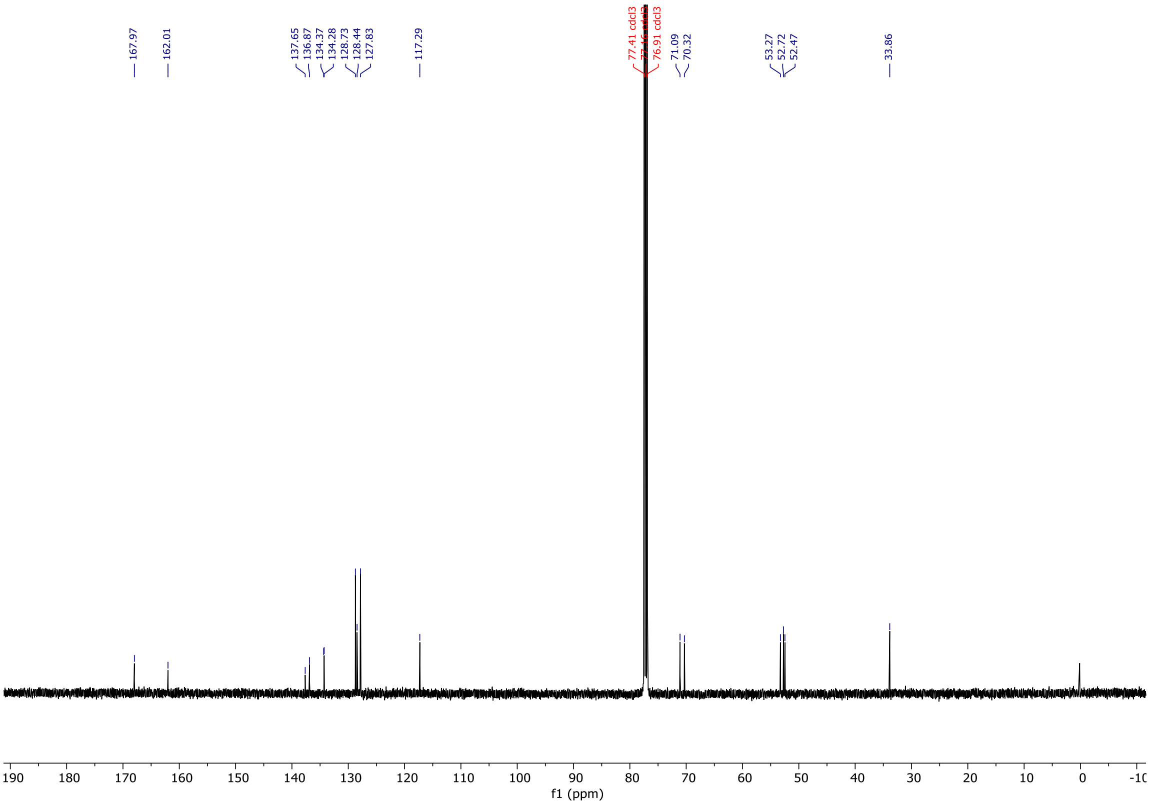

methyl 1-benzyl-5-(1-cyclohexyl-2-methoxy-2-oxoethyl)-1H-1,2,3-triazole-4-carboxylate (21)

^{1}H NMR ($CDCl_3$, 500 MHz)

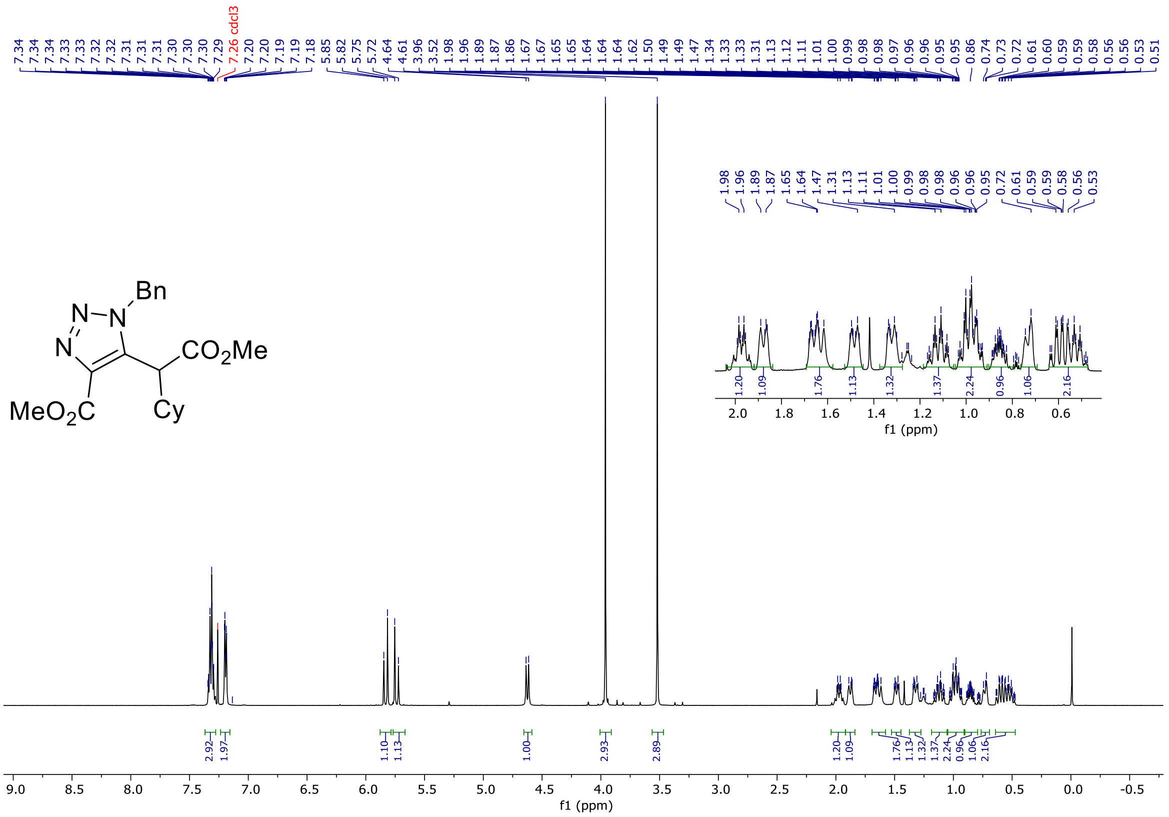

^{13}C NMR ($CDCl_3$, 126 MHz)

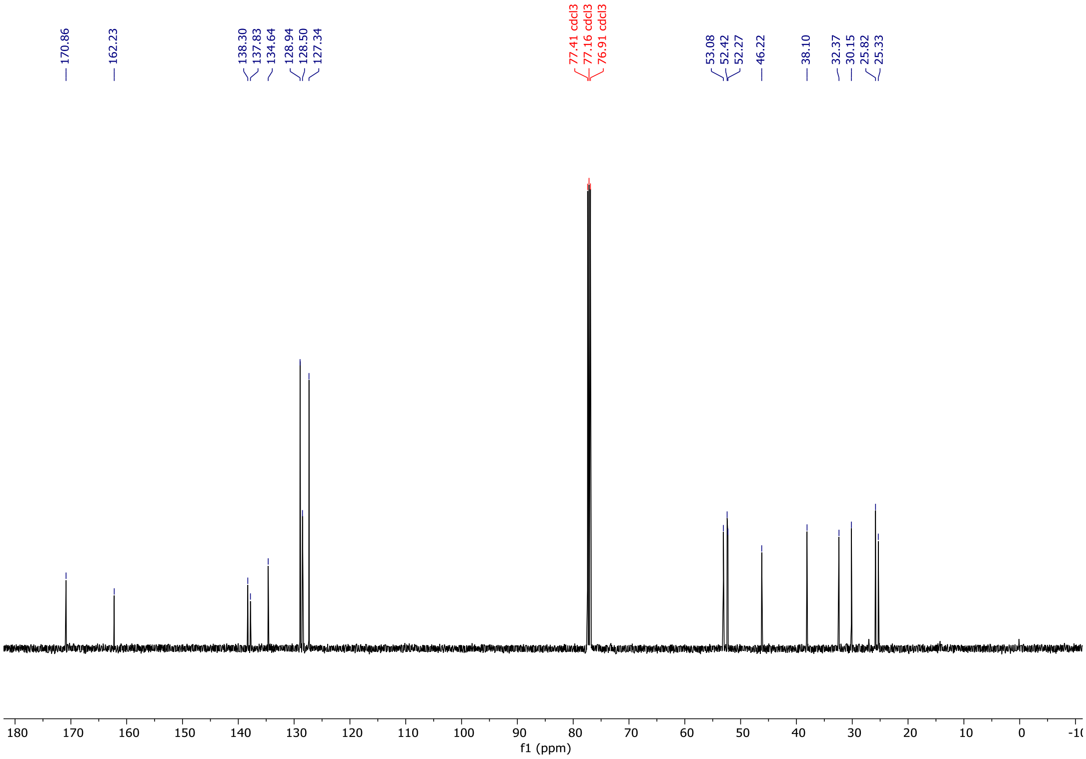

methyl 5-(1-(benzoyloxy)-2-methoxy-2-oxoethyl)-1-benzyl-1H-1,2,3-triazole-4-carboxylate (22a)

^{1}H NMR (CDCl$_3$, 500 MHz)

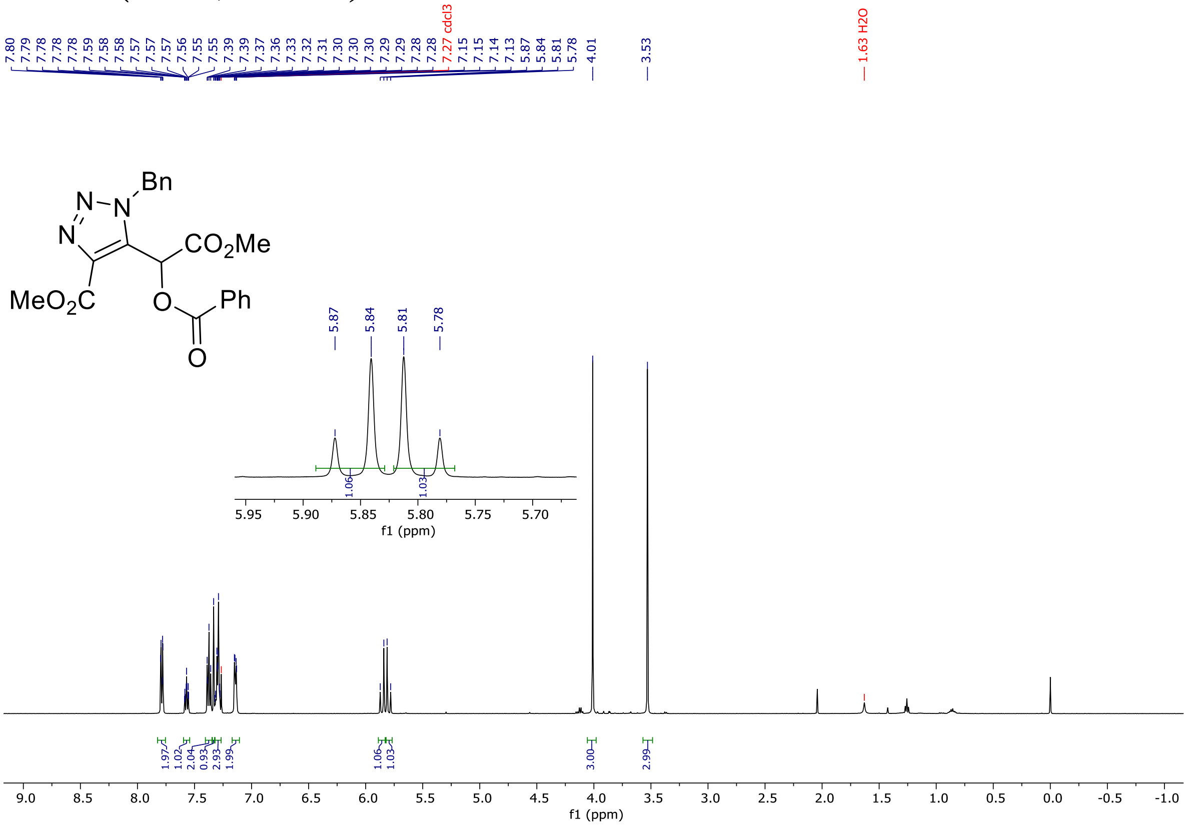

^{13}C NMR (CDCl$_3$, 126 MHz)

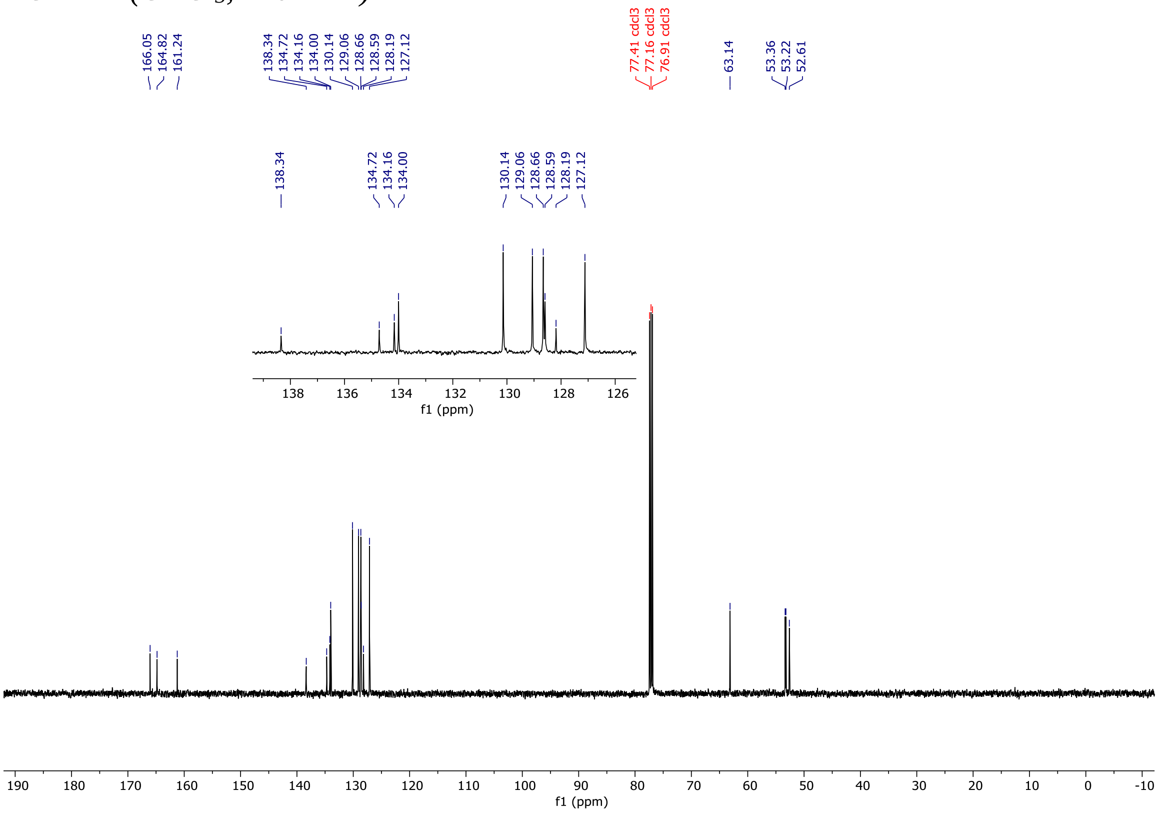

methyl 5-(1-(benzoyloxy)-2-methoxy-2-oxoethyl)-1-phenyl-1H-1,2,3-triazole-4-carboxylate (22b)

^{1}H NMR (CDCl$_3$, 500 MHz)

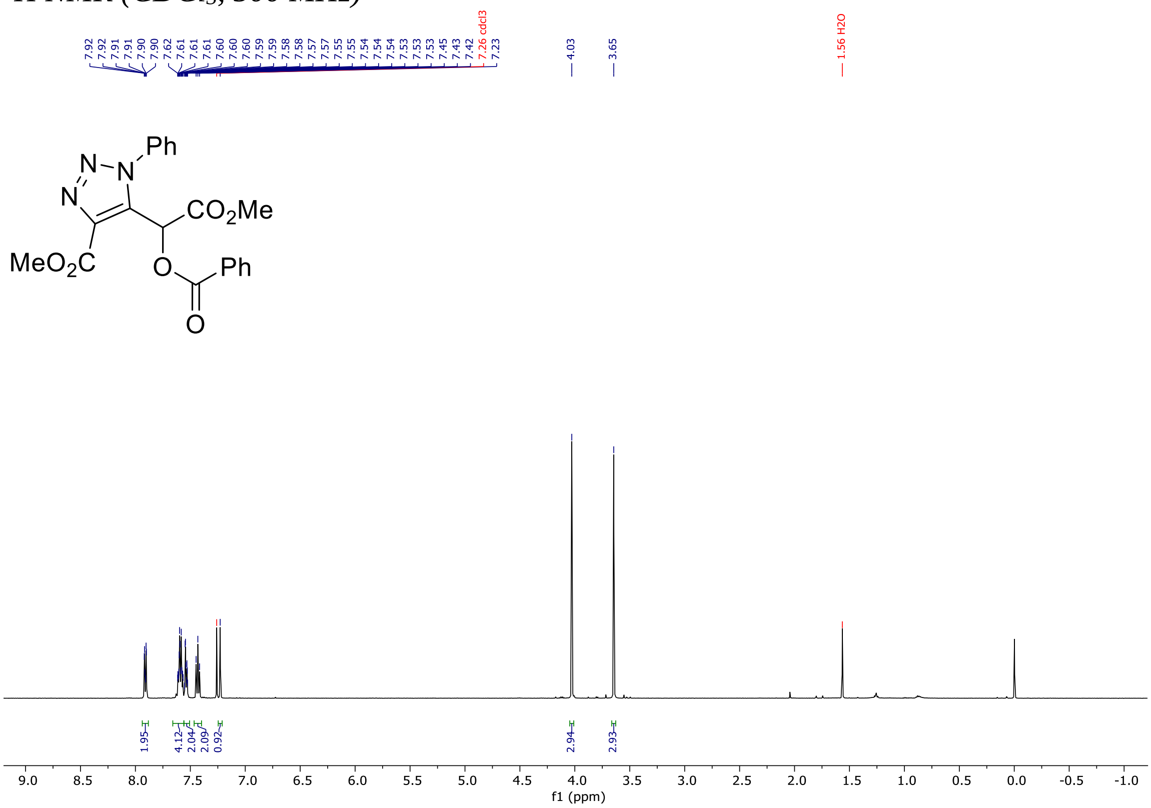

^{13}C NMR (CDCl$_3$, 126 MHz)

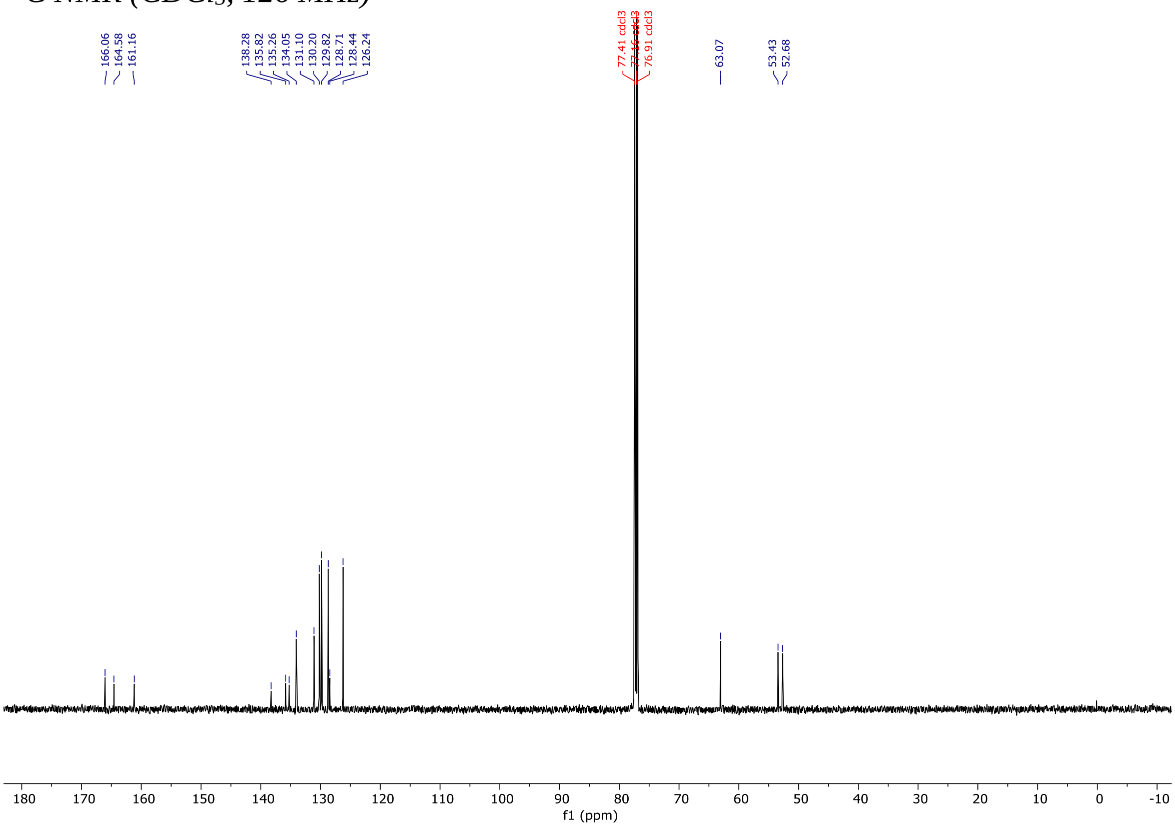

methyl 5-(1-(benzoyloxy)-2-methoxy-2-oxoethyl)-1-cyclopropyl-1H-1,2,3-triazole-4-carboxylate (22c)

^{1}H NMR ($CDCl_3$, 500 MHz)

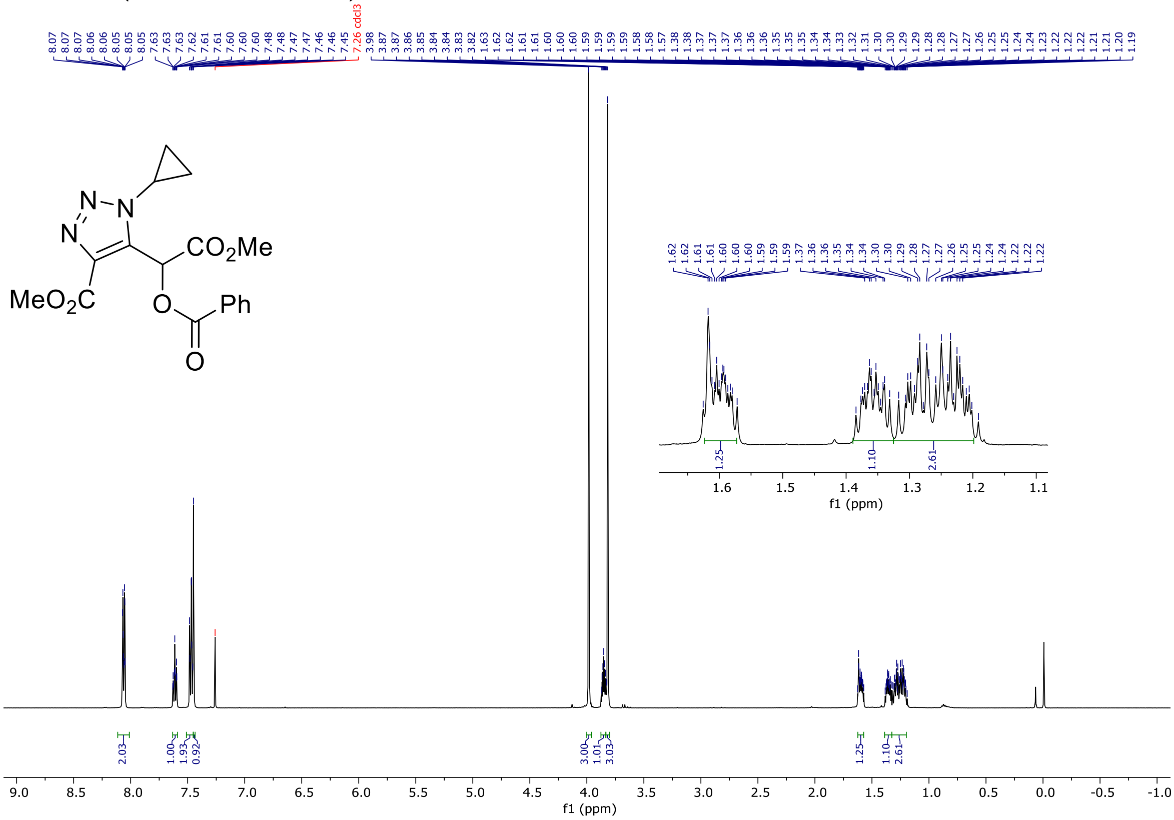

^{13}C NMR ($CDCl_3$, 126 MHz)

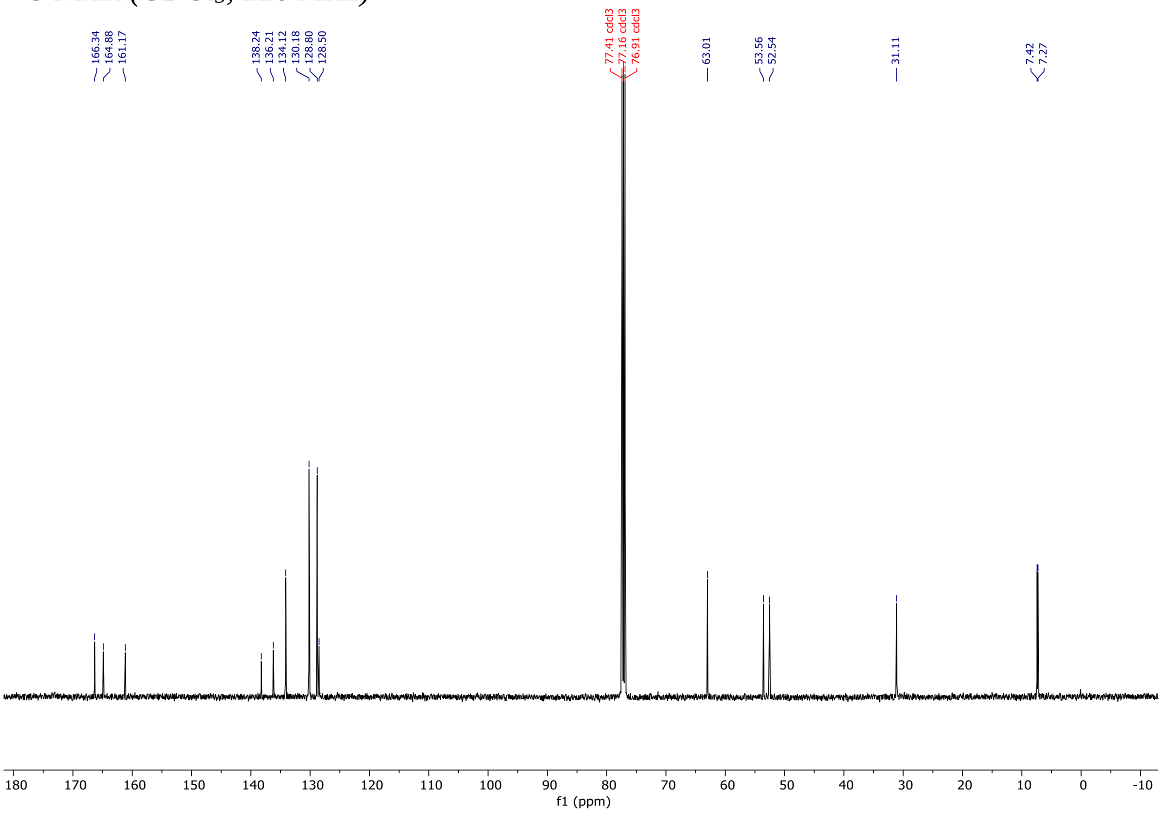

methyl 5-(1-(benzoyloxy)-2-methoxy-2-oxoethyl)-1-propyl-1H-1,2,3-triazole-4-carboxylate (22d)

^{1}H NMR (CDCl$_3$, 400 MHz)

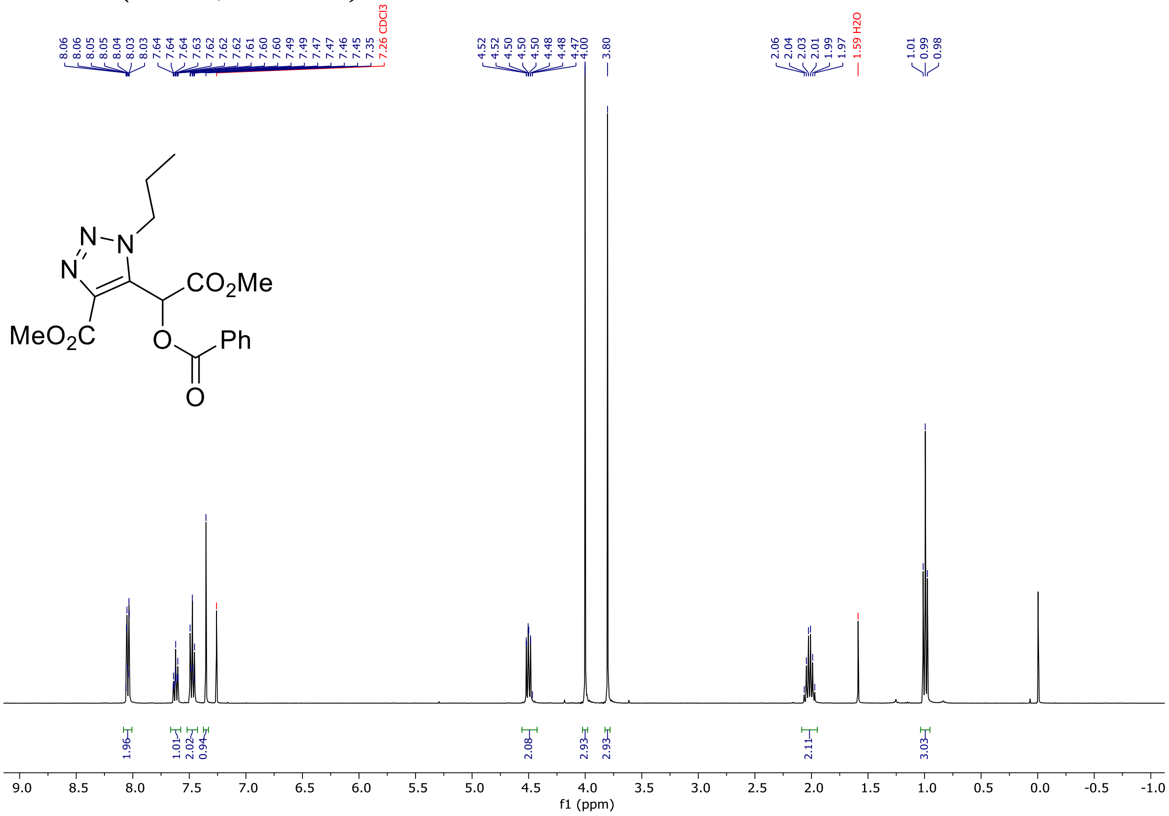

^{13}C NMR (CDCl$_3$, 126 MHz)

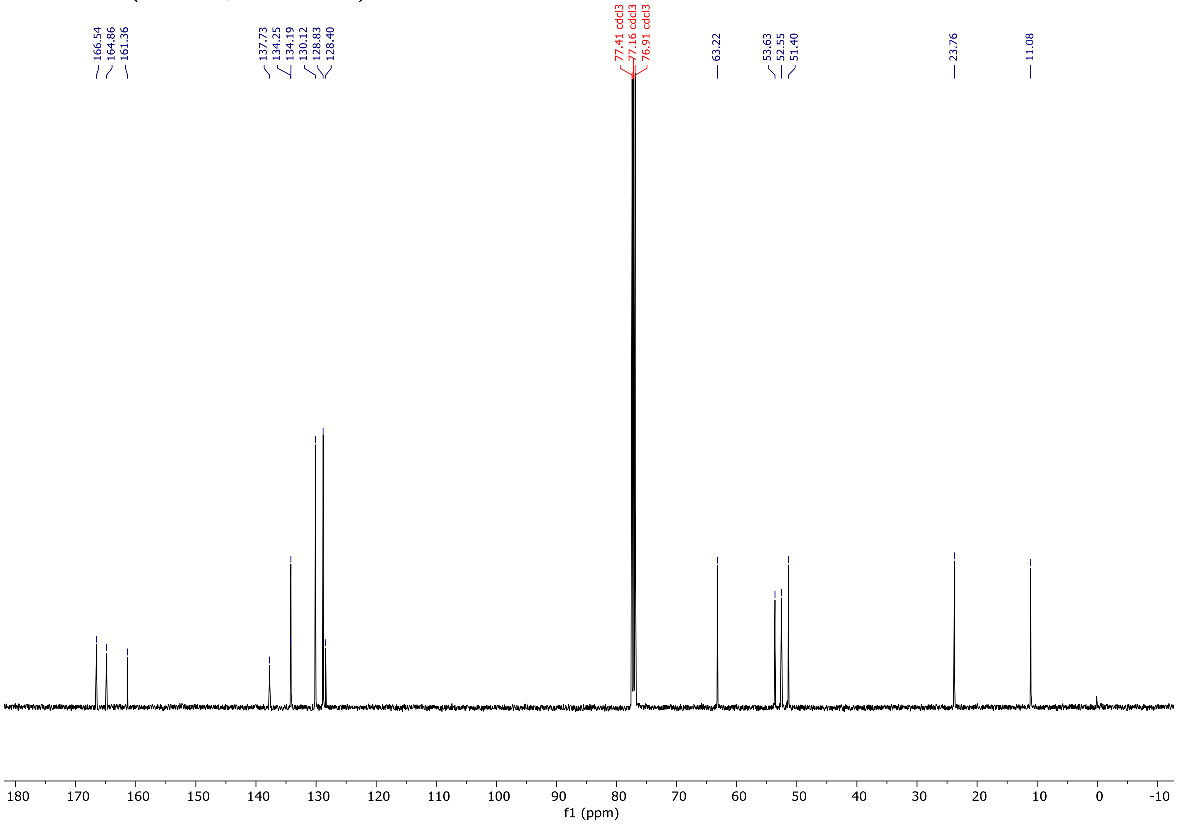

methyl 1-benzyl-5-(2-methoxy-2-oxo-1-(phenylthio)ethyl)-1H-1,2,3-triazole-4-carboxylate (23)

^{1}H NMR (CDCl$_3$, 500 MHz)

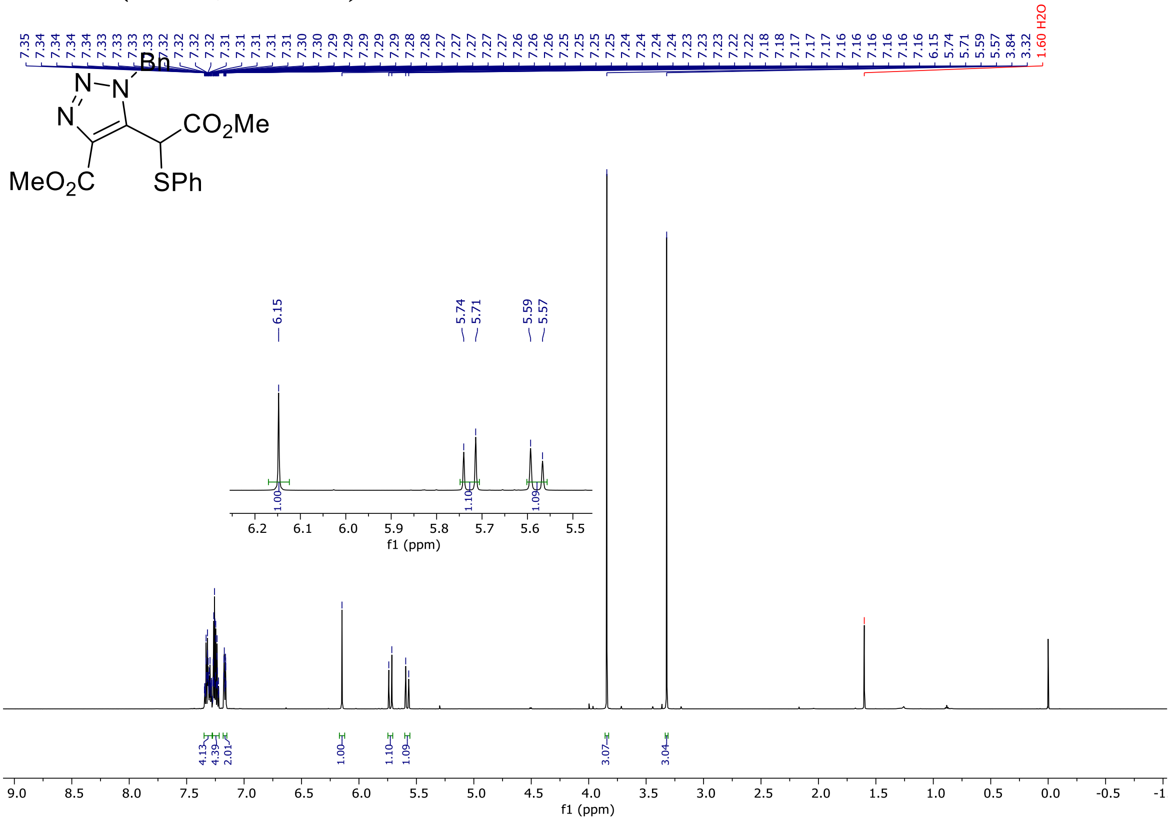

^{13}C NMR (CDCl$_3$, 126 MHz)

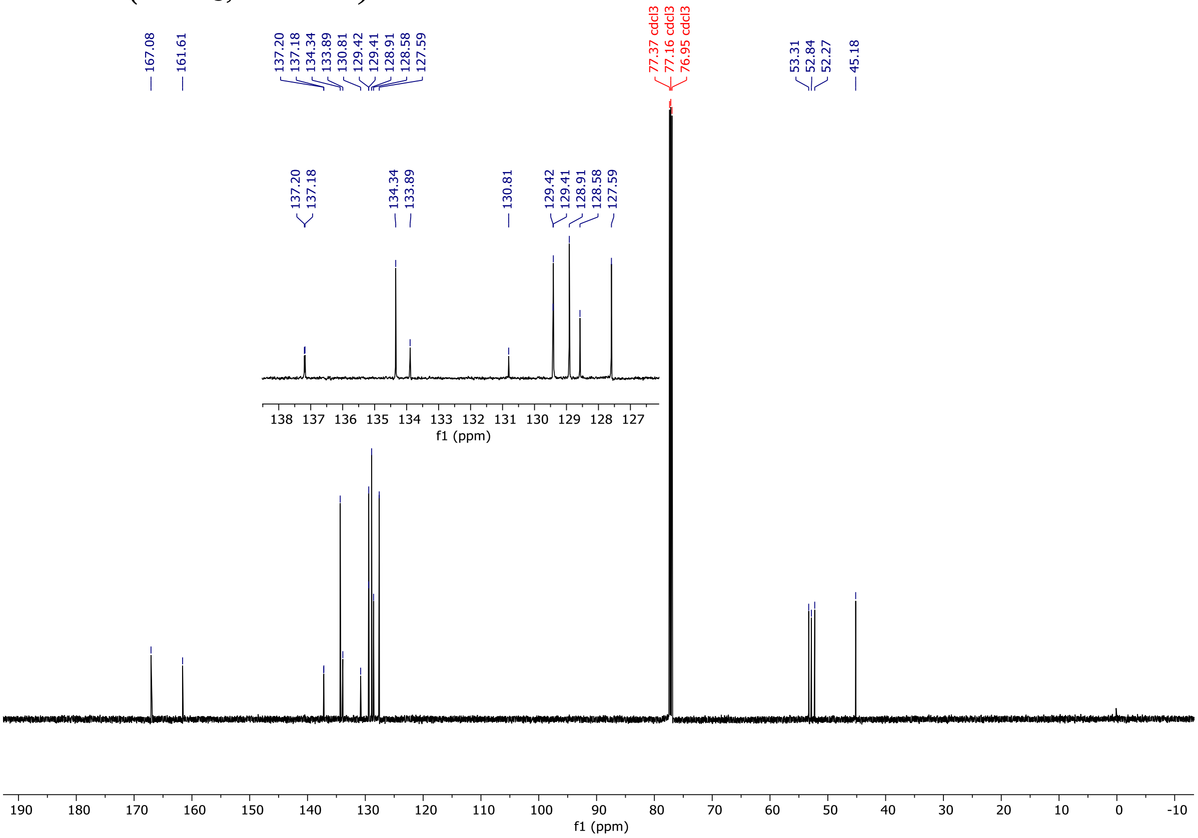

methyl 1-benzyl-5-(1-(benzylamino)-2-methoxy-2-oxoethyl)-1H-1,2,3-triazole-4-carboxylate (24)

^{1}H NMR (CDCl$_3$, 500 MHz)

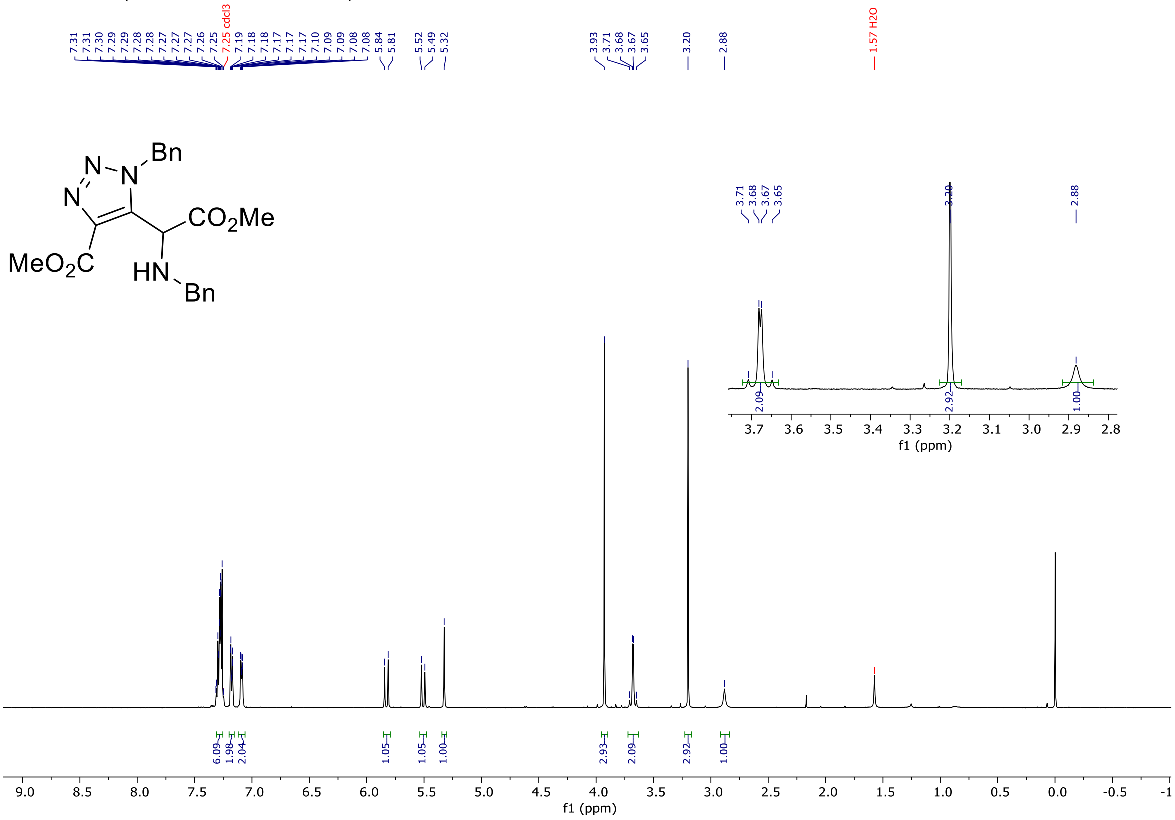

^{13}C NMR (CDCl$_3$, 126 MHz)

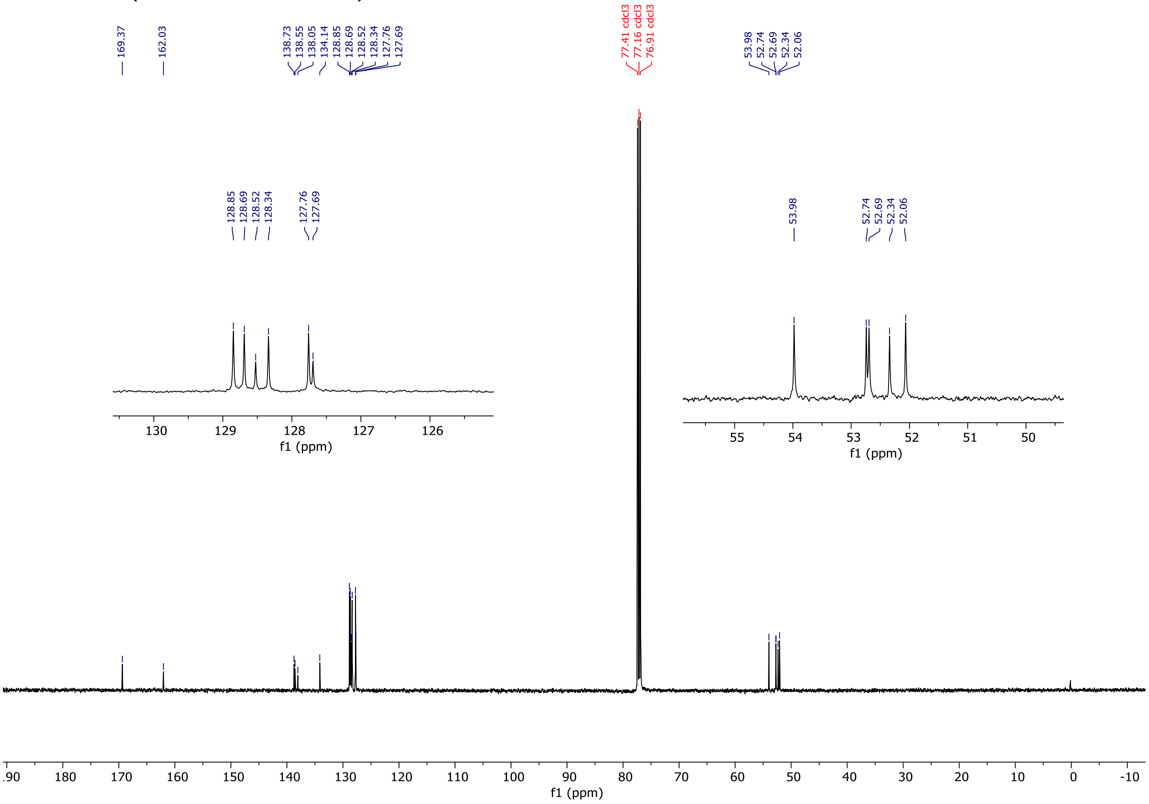

methyl 1-benzyl-5-((2R,3S)-1-(methoxycarbonyl)-2,3-diphenylcyclopropyl)-1H-1,2,3-triazole-4-carboxylate (S3)

^{1}H NMR ($CDCl_3$, 400 MHz)

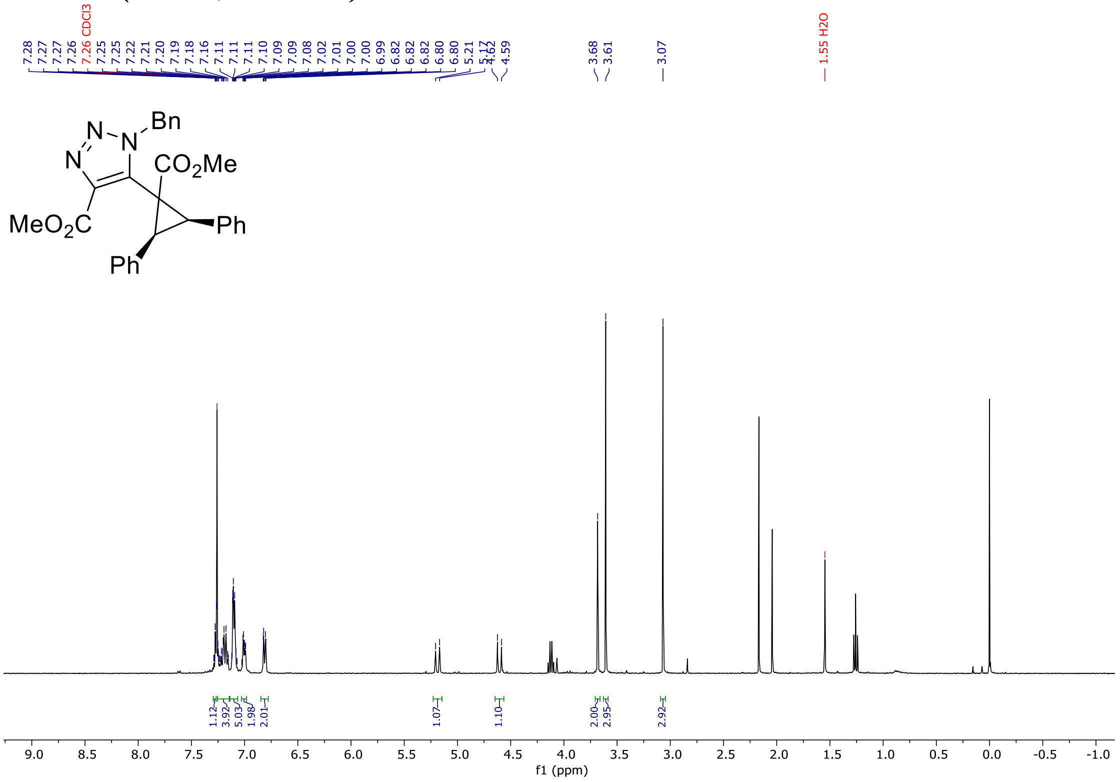

^{13}C NMR ($CDCl_3$, 126 MHz)

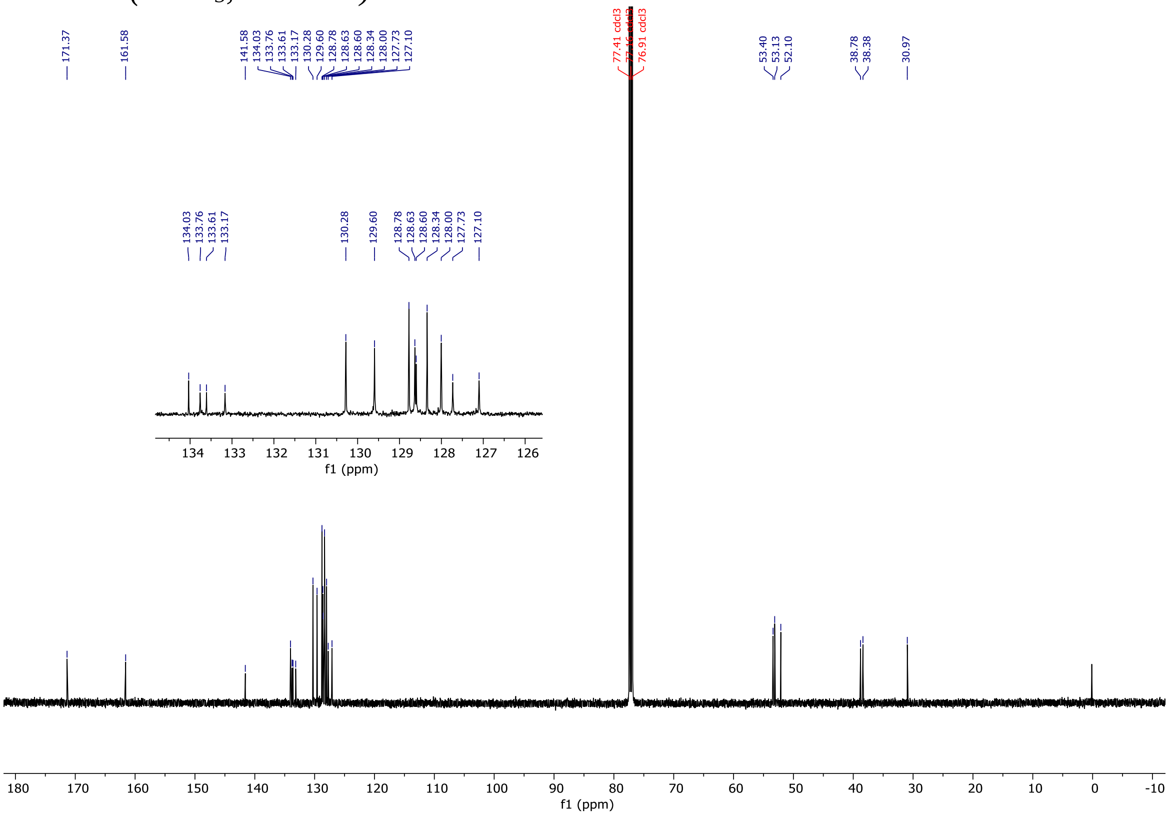

methyl 1-benzyl-5-(2,3-dichloro-1-methoxy-1-oxopropan-2-yl-3,3-d2)-1H-1,2,3-triazole-4-carboxylate (25)

^{1}H NMR (CDCl3, 400 MHz)

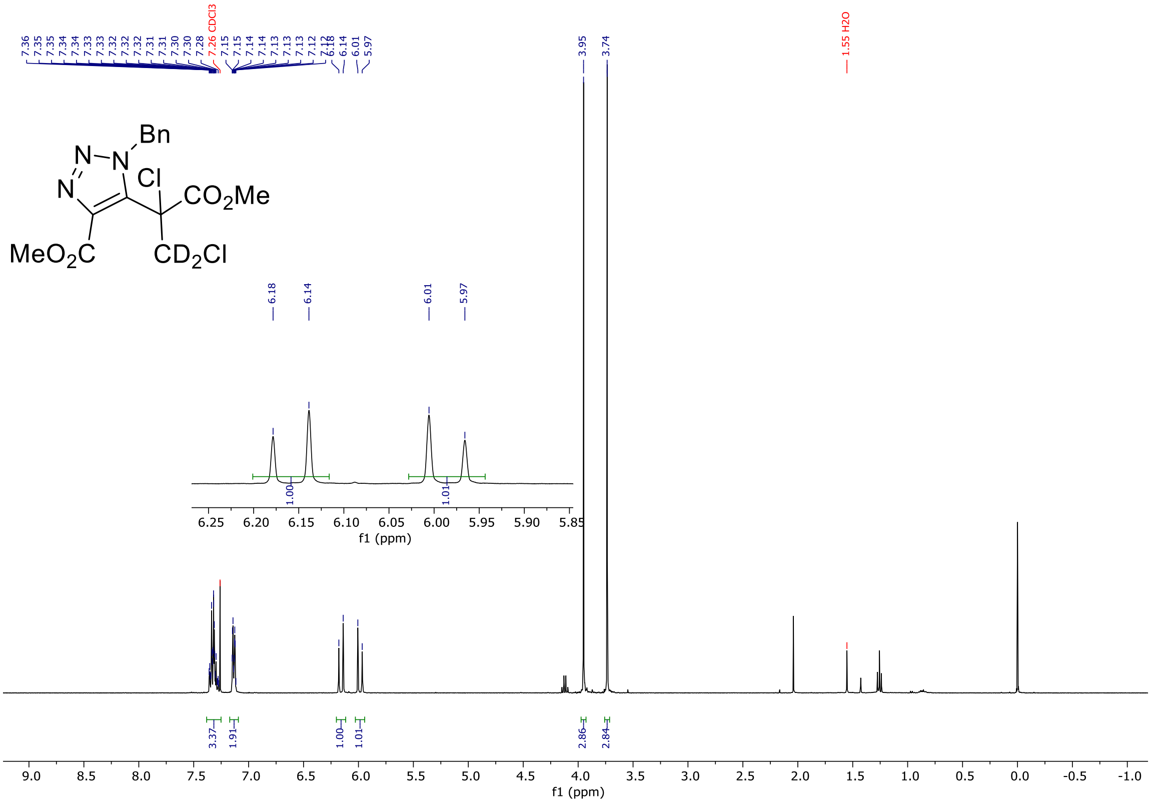

^{13}C NMR (CDCl3, 126 MHz)

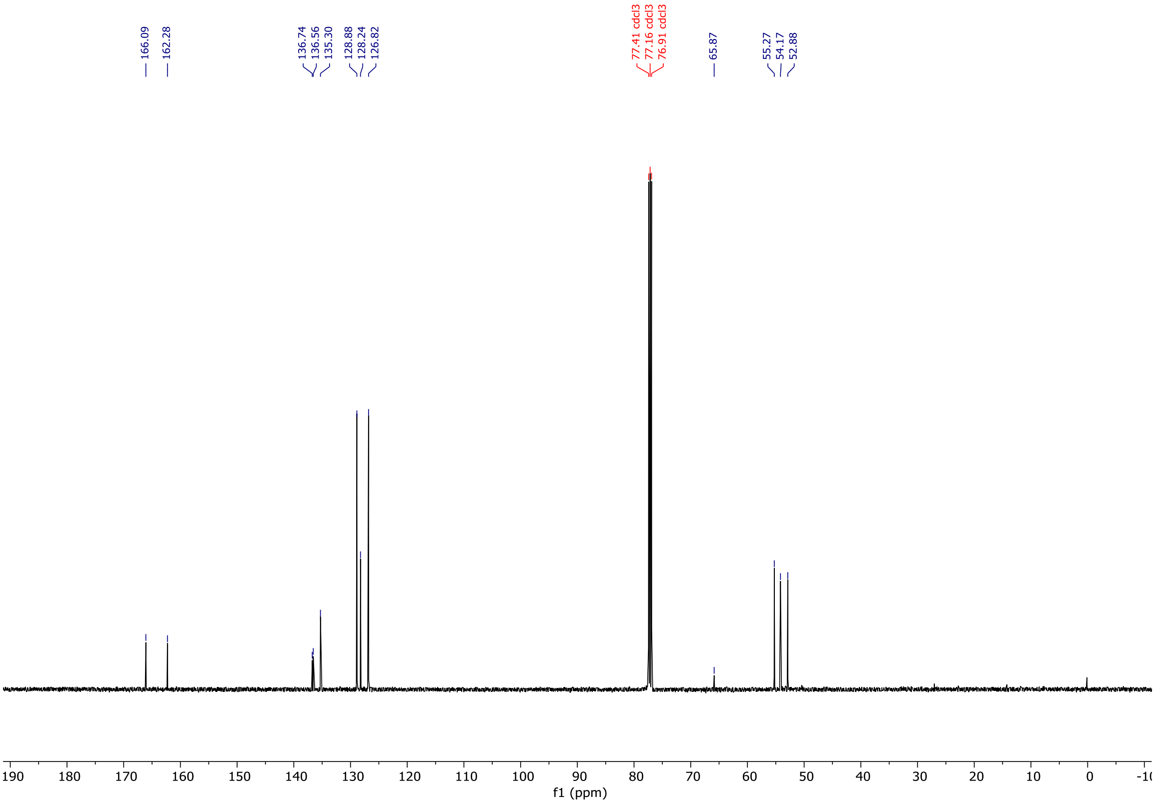

10. References

(1) Dolomanov, O. V.; Bourhis, L. J.; Gildea, R. J.; Howard, J. A. K.; Puschmann, H. *OLEX2* : A Complete Structure Solution, Refinement and Analysis Program. *J. Appl. Crystallogr.* **2009**, *42*, 339–341. https://doi.org/10.1107/S0021889808042726.

(2) Agilent Technologies, CrysAlisPro, Version 1.171.40.84a.

(3) Sheldrick, G. M. Crystal Structure Refinement with SHELXL. Acta Crystallogr., Sect. C: Struct. Chem. 2015, C71, 3-8.

(4) Farrugia, L. J. WinGX Suite for Small-Molecule Single-Crystal Crystallography. J. Appl. Cryst. 1999, 32, 837-838.

(5) Gaussian 16, Revision C.01, M. J. Frisch, G. W. Trucks, H. B. Schlegel, G. E. Scuseria, M. A. Robb, J. R. Cheeseman, G. Scalmani, V. Barone, G. A. Petersson, H. Nakatsuji, X. Li, M. Caricato, A. V. Marenich, J. Bloino, B. G. Janesko, R. Gomperts, B. Mennucci, H. P. Hratchian, J. V. Ortiz, A. F. Izmaylov, J. L. Sonnenberg, D. Williams-Young, F. Ding, F. Lipparini, F. Egidi, J. Goings, B. Peng, A. Petrone, T. Henderson, D. Ranasinghe, V. G. Zakrzewski, J. Gao, N. Rega, G. Zheng, W. Liang, M. Hada, M. Ehara, K. Toyota, R. Fukuda, J. Hasegawa, M. Ishida, T. Nakajima, Y. Honda, O. Kitao, H. Nakai, T. Vreven, K. Throssell, J. A. Montgomery, Jr., J. E. Peralta, F. Ogliaro, M. J. Bearpark, J. J. Heyd, E. N. Brothers, K. N. Kudin, V. N. Staroverov, T. A. Keith, R. Kobayashi, J. Normand, K. Raghavachari, A. P. Rendell, J. C. Burant, S. S. Iyengar, J. Tomasi, M. Cossi, J. M. Millam, M. Klene, C. Adamo, R. Cammi, J. W. Ochterski, R. L. Martin, K. Morokuma, O. Farkas, J. B. Foresman, and D. J. Fox, Gaussian, Inc., Wallingford CT, 2019.

(6) Grimme, S.; Antony, J.; Ehrlich, S.; Krieg, H. A Consistent and Accurate Ab Initio Parametrization of Density Functional Dispersion Correction (DFT-D) for the 94 Elements H-Pu. *J. Chem. Phys*. **2010**, *132*, 154104

(7) Marenich, A. V. ; Cramer, C.J.; Truhlar, D.G. *J. Phys. Chem. B* **2009**, *113*, 6378–6396.

(8) CYLview20; Legault, C. Y., Université de Sherbrooke, 2020 (http://www.cylview.org)
